# Genomic Copy Number Variants in CML Patients With the Philadelphia Chromosome (Ph+): An Update

**DOI:** 10.3389/fgene.2021.697009

**Published:** 2021-08-10

**Authors:** Heyang Zhang, Meng Liu, Xiaoxue Wang, Yuan Ren, Young Mi Kim, Xianfu Wang, Xianglan Lu, Hui Pang, Guangming Liu, Yue Gu, Mingran Sun, Yunpeng Shi, Chuan Zhang, Yaowen Zhang, Jianqin Zhang, Shibo Li, Lijun Zhang

**Affiliations:** ^1^Department of Hematology, The First Hospital of China Medical University, Shenyang, China; ^2^Department of Pediatrics, University of Oklahoma Health Sciences Center, Oklahoma City, OK, United States; ^3^Department of Gastroenterology, The First Hospital of Jilin University, Changchun, China; ^4^Department of Respiratory and Intensive Care Medicine, The First Hospital of Jilin University, Changchun, China; ^5^Department of Hematology and Oncology, Anshan Hospital of First Hospital of China Medical University, Shenyang, Anshan, China; ^6^Department of Hepatobiliary and Pancreatic Surgery, China-Japan Union Hospital of Jilin University, Changchun, China; ^7^Gansu Province Medical Genetics Center, Gansu Provincial Maternal and Child Health Care Hospital, Lanzhou, China; ^8^Department of Neurology, The Second Hospital of Jilin University, Changchun, China; ^9^Department of Pediatric Respiratory, Dalian Children’s Hospital, Dalian, China

**Keywords:** chronic myeloid leukemia, copy number variations, del(9q), *ASS1* gene, additional chromosomal aberrations

## Abstract

**Background:**

Submicroscopic segmental imbalances detected by array-comparative genomic hybridization (array-CGH) were discovered to be common in chronic myeloid leukemia (CML) patients with *t*(9;22) as the sole chromosomal anomaly. To confirm the findings of the previous study and expand the investigation, additional CML patients with *t*(9;22) as the sole chromosomal anomaly were recruited and copy number variants (CNVs) were searched for.

**Methods:**

Karyotyping tests were performed on 106 CML patients during January 2010–September 2019 in our Genetics Laboratory. Eighty-four (79.2%) patients had the Philadelphia (Ph) chromosome as the sole chromosomal anomaly. Only 49(58.3%) of these 84 patients had sufficient marrow or leukemia blood materials to additionally be included in the array-CGH analysis. Fluorescence *in situ* hybridization (FISH) was carried out to confirm the genes covered by the deleted or duplicated regions of the CNVs.

**Results:**

11(22.4%) out of the 49 patients were found to have one to three somatic segmental somatic segmental (CNVs), including fourteen deletions and three duplications. The common region associated with deletions was on 9q33.3-34.12. Identified in five (45.5%) of the 11 positive patients with segmental CNVs, the deletions ranged from 106 kb to 4.1 Mb in size. Two (18.2%) cases had a deletion in the ABL1-BCR fusion gene on der (9), while three (27.3%) cases had a deletion in the ASS1 gene. The remaining CNVs were randomly distributed on different autosomes.

**Conclusion:**

Subtle genomic CNVs are relatively common in CML patients without cytogenetically visible additional chromosomal aberrations (ACAs). Long-term studies investigating the potential impact on patient prognosis and treatment outcome is underway.

## Introduction

Chronic myeloid leukemia (CML) results from the neoplastic transformation of hematopoietic stem cell and it is cytogenetically characterized by the presence of the Philadelphia (Ph) chromosome (Ren, 2005). The Ph chromosome is the consequence of a *t*(9;22)(q34;q11) translocation. This chromosomal translocation generates a fusion gene that encodes BCR-ABL, a constitutively active protein tyrosine kinase (Hochhaus and La Rosee, 2004). Additional chromosomal aberrations (ACAs) in Ph+ cells, such as trisomy 8, a second Ph chromosome, or isochromosome (17)(q10), occur during the course of the disease and is strongly associated with disease progression and treatment outcome (Fabarius et al., 2011; Luatti et al., 2012; Muvarak et al., 2012). Similarly, mutations in the BCR-ABL kinase domain and submicroscopic chromosomal segmental imbalances have been documented to negatively impact on treatment outcomes in CML patients (Kolomietz et al., 2001; Soverini et al., 2009; Nadarajan et al., 2011).

Based on a previous study (Lu et al., 2012), subtle somatic copy number changes detected by array-comparative genomic hybridization (array-CGH) were commonly present in CML patients with a *t*(9;22)(q34;q11) as the sole chromosomal anomaly. Leading to advanced stages of CML and resistance to therapy, such genetic alterations may be manifestations of underlying genomic instability resulting from a compromised DNA damage and repair response (Muvarak et al., 2012). Current evidence also indicates that acquired genetic instability has an inseparable relationship with the continuous acquisition of ACAs (Fabarius et al., 2015; Wang et al., 2016). Furthermore, some of the cryptic genomic changes may be drivers of ACAs and further influence disease progression and therapeutic approaches (Lavrov et al., 2017).

In the current study, additional patient samples with *t*(9;22)(q34;q11) as the sole chromosomal anomaly were investigated. By performing high-density whole genomic array-CGH, similar percentage with cryptic changes were identified.

## Materials and Methods

### Patients

This study was approved by the Institutional Review Board (IRB) of the Oklahoma University (IRB number: 2250). A total of 106 CML patient samples were studied cytogenetically from 2000 to 2019 at the Genetics Laboratory of Oklahoma University Health Sciences Center. DNA samples were obtained from forty-nine of these 106 patients.

### Conventional Cytogenetic Analysis

Short-term cultures of unstimulated bone marrow samples were set up and harvested according to the standard laboratory protocols. Karyotype analysis was performed using giemsa and trypsin technique for G-banding. The cytogenetic abnormalities were described according to the International System for Human Cytogenetic Nomenclature (McGowan-Jordan et al., 2016).

### Array-CGH

Genomic DNA was extracted from the cell pellet of each patient’s bone marrow according to the standard operating procedure using the phenol and chloroform method with a commercially-available DNA extraction kit (Puregene Blood Kit, Qiagen, Valencia, CA, United States) or Nucleic Acid Isolation System (QuickGene-610L, FUJIFILM Corporation, Tokyo, Japan). Two aCGH platforms including NimbleGen and Agilent were used in this study. For the NimbleGen aCGH platform, human reference genomic DNA was purchased from Promega Corporation (Promega Corporation, Madison, WI, United States). The patient’s DNA and the reference DNA were labeled with either Cyanine 3 (Cy-3) or Cyanine 5 (Cy-5) by random priming, and then equal quantity of both labeled products were mixed and loaded onto a 720 K oligonucleotide chip (Roche NimbleGen Inc., Madison, WI, United States) to hybridize at 42°C for 40 h in a MAUI hybridization system (BioMicro Systems, Salt Lake City, UT, United States) according to the manufacturer’s protocols with minor modifications. The slides were washed with washing buffers (Roche NimbleGen Inc., Madison, WI, United States) after hybridization and scanned using Roche Scanner MS200 (Roche NimbleGen Inc., Madison, WI, United States). Images were analyzed using software from NimbleScan software version 2.6 and the SignalMap software version 1.9 (Roche NimbleGen Inc., Madison, WI, United States). The genomic positions were determined using GRCh36/hg18, UCSC Genome Browser. For the Agilent aCGH platform, human reference genomic DNA was purchased from Agilent Corporation (Agilent Corporation, Santa Clara, CA, United States). The patient’s DNA and the purchased reference DNA were labeled with either cyanine 3 (Cy-3) or cyanine 5 (Cy-5) by random priming (Agilent Corporation, Santa Clara, CA, United States). Patient DNA (labeled with Cy-3) was combined with a normal control DNA sample (labeled with Cy-5) of the same sex and hybridized to an Agilent 2 × 400 K oligo microarray chip (Agilent Technologies) by incubating in Agilent’s Microarray Hybridization Ovens (Agilent Technologies). After a 40 h of hybridization at 67°C, the slides were washed and scanned using the NimbleGen MS 200 Microarray Scanner (Roche NimbleGen Inc., Madison, WI, United States). Agilent’s CytoGenomics 2.7 software (Agilent Technologies) was applied for data analysis. The genomic positions were determined using GRCh37/hg19, UCSC Genome Browser.

### Fluorescence *in situ* Hybridization Confirmation Studies

Fluorescence *in situ* hybridization (FISH) analyses were subsequently performed to confirm the copy number changes (CNVs) detected by array-CGH. Commercially available FISH probes (Abbott Molecular, Des Plains, IL, United States) or FISH probes prepared in-house with BAC/PAC clones purchased from the Children’s Hospital Oakland Research Institute (Oakland, CA, United States) were used. All probes were validated before being applied to the patient samples.

## Results

In this study, a total of 49 CML cases with *t*(9;22) as sole anomaly were evaluated by array-CGH assay to check whether those patients who had normal karyotype, may have subtle chromosome changes not visible by routine cytogenetic testing. It turned out that eleven (22.4%) out of those 49 cases had a total of 17 segmental CNVs detected, including both deletions and duplications. Five (29.4%) of those CNVs were clustered on the derivative chromosome 9 and the remaining 12 (70.6%) CNVs were distributed on 2p14, 2p16.2, 4q31.21, 17p11.2, 17p12, 19q13.33, and 22q11.23-q12.1 with loss, and 1q43, 5q13.2, 22q11.23-q12.1 with gain. The size of these CNVs ranged from 74 to 4,064 kb. Five (45.5%) of these eleven cases (case 1, 3, 8, 10, and 11) had a partial deletion of the long arm of chromosome 9, their cryptic deletions of 9q were in variable sizes between 106-4, 064 kb. Three of them (case 3, 8, and 10) had the deletion of the ASS1 gene on the derivative chromosome 9. Cases 3 and 8 had extended deletion of the *ABL1-BCR* fusion gene on the derivative chromosome 9, and case 10 had only deletion of the ASS1 which were confirmed by FISH assay. The breakpoints and molecular sizes of the deletions on both chromosomes 9 and 22 were different in these three cases (Table 1).

**TABLE 1:** Ph+ chronic myeloid leukemia (CML) patients with copy number variants (CNVs).

**Case**	**Gender**	**Age**	**Diagnosis**	**Conventional cytogenetics**	**Chr**	**Cytoband**	**Size (kb)**		**Start (bp)**	**Stop (bp)**
							**Loss**	**gain**		
1	M	80	BC	46,XX,*t*(9;22)(q34;q11.2)[20].nuc ish (ABL1,BCR)x3(ABL1 con BCRx2)[195/200]	9	q34.12	105.69		133576051	133681919
2	F	71	CP	46,XX,*t*(9;22)(q34;q11.2)[20]	19	q13.33	73.946		48212252	48286197
3	M	69	CP	46,XX,*t*(9;22)(q34;q11.2)[20]	9	q33.3–q34.12	3,368.571		130211514	133580084
					22	q11.23–q12.1	3,345.445		23634972	26980416
4	M	67	CP	46,XX,*t*(9;22)(q34;q11.2)[17].nuc ish (ABL1,BCR)x3(ABL1 con BCRx2)[198/200]	2	p16.2	222.463		54564146	54786608
					4	q31.21	2097.826		142143472	144241297
5	M	36	CP	46,XX,*t*(9;22)(q34;q11.2)[20].nuc ish (ABL1,BCR)x3(ABL1 con BCRx2)[196/200]	5	q13.2		2127.377	68728731	70856107
6	F	–	CP	46,XX,*t*(9;22)(q34;q11.2)[20]	17	p11.2	613		16581147	17194128
7	F	21	CP	46,XX,*t*(9;22)(q34;q11.2)[22]	1	q43		405	239954040	240359460
8	M	–	CP	46,XX,*t*(9;22)(q34;q11.2)[20]	2	p14	840.474		65033335	65873808
					9	q33.3–q34.12	4064.166		129644449	133708614
					22	q11.23	1126.469		23634972	24761440
9	M	35	CP	46,XX,*t*(9;22)(q34;q11.2)[19].nuc ish (ABL1,BCR)x3(ABL1 con BCRx2)[196/200]	17	p12	1342		14100118	15442206
10	F	26	CP	46,XX,*t*(9;22)(q34;q11.2)[20].nuc ish (ABL1,BCR)x3(ABL1 con BCRx2)[200/200]	9	q34.11–q34.12	692		132870231	133562475
11	M	65	BC	46,XX,*t*(9;22)(q34;q11.2)[20]	9	q34.12	119.087		133595219	133714305
					22	q11.23	1073.861		23633252	24707112
					22	q11.23–q12.1		101.297	25809371	25910667

**FIGURE 1:**
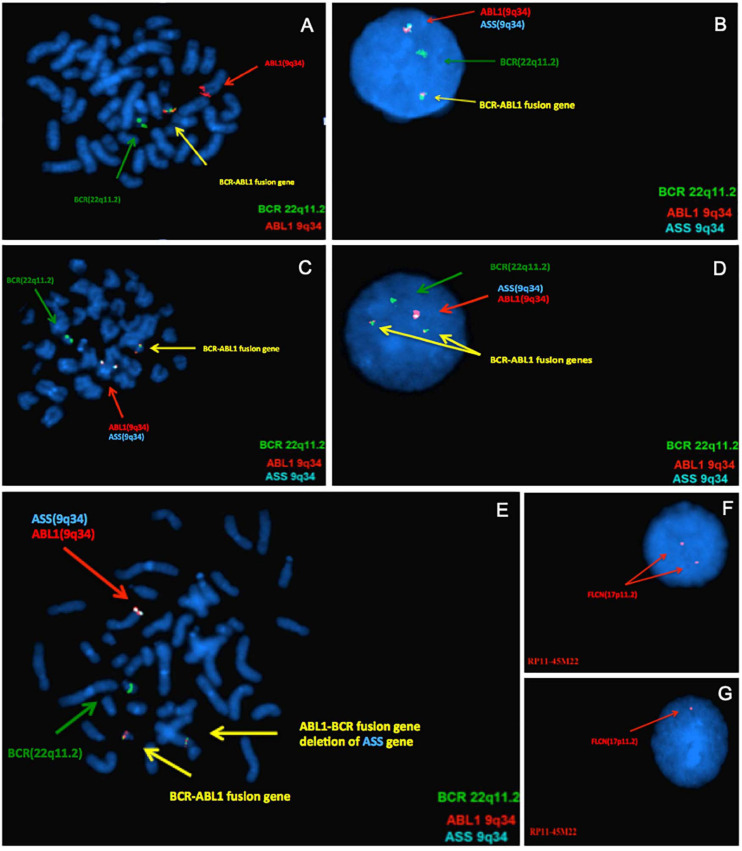
Results of confirmatory Fluorescence *in situ* hybridization (FISH) analyses. From panels **(A–E)**, the green signal represents *BCR* gene on 22q11.2, the red and aqua signals on 9q34 represent *ABL1* and *ASS* gene, respectively. The yellow signal represents *BCR-ABL* fusion gene. **(A)** FISH analysis using metaphase of case 3 confirmed the array-CGH finding, it had only one yellow fusion signal on der(22) and deletion of *ABL-BCR* fusion gene on der(9). **(B)** FISH test using DNA probes specific for *BCR-ABL* fusion gene of Case 8, indicate that one of the fusion gene *ABL-BCR* on der(9) was deleted. Only one *ASS* signal (aqua) can be seen, indicating *ASS1* gene deleted. **(C)** Metaphase of Case 8. **(D)** This result confirms the deletion of 9q34 on the *ASS* gene of Case 10, which is associated with the disease. **(E)** Metaphase of Case 10. As for panels **(F,G)**, the *FLCN* gene located at chromosome 17p11.2. In Case 6, the FISH analysis showed normal hybridization signal patterns in the interphase **(F)** and one *FLCN* gene deletion in the interphase **(G)**.

To confirm these CNVs, FISH assay was performed using either commercial FISH probes or home brewed FISH probes derived from clones purchased from Children’s Hospital Oakland Research Institute, involving 9q33.3-q34.12, 9q34.11-q34.12, 17p11.2, 19q13.41, and 22q11.23-q12.1 (Table 1). Case 1 had a 9q deletion region at band q34.12. Case 3, 8, and 10 had an imbalance in the genomic regions that was associated with the breakpoints of the *t*(9;22) at bands q33.3–q34.1. Subsequent FISH analysis showed *ABL1-BCR* fusion gene deletion on der (9) in Case 3 and Case 8 (Figures 1A–C). All of these three cases were found the ASS1 gene deletion on der(9) (Figures 1D,E). Case 4 had a loss in the short arm of chromosome 2 at band p16.2 and also in the long arm of chromosome 4 at bands q31.21. Case 5 had a duplication segment of chromosome 5q at band q13.2. Case 7 was also found a gain region in chromosome 1q43. Case 6 had a loss in the short arm of chromosome 17 at bands p11.2, which was confirmed by FISH with BAC clone RP11-45M22 (Figures 1F,G). Case 9 had a loss in the short arm of chromosome 17 at bands p12, this large region did not need to confirm. Case 11 had a 101 kb gain of chromosome 22 and two loss regions related to 9q34.12 and 22q11.23. Detail information of 11 patients who revealed CNVs can be found in Supplementary Material. Basic information of the 49 patients who received array-CGH tests can be found in Supplementary Table 1.

## Discussion

Additional chromosomal aberrations at the cytogenetic [+der(22), +8, i(17q), and +19] levels, along with a Ph+ chromosome, occurs during the course of CML and is strongly associated with disease progression and poor treatment outcomes (Alpar et al., 2012; van der Sligte et al., 2014). Generally speaking, patients presenting with only *t*(9;22)(q34;q11) anomalies may have subtle genomic segmental alterations that conventional cytogenetic analysis can’t detect.

In a previous pilot study using whole genomic oligonucleotide array-CGH analysis in 19 CML patients with the Ph chromosome as the sole aberration, it was demonstrated that subtle genomic copy changes are relatively common (Lu et al., 2012).

Here, an additional 49 CML patient samples positive for *t*(9;22), but negative for secondary chromosomal changes according to G-banded chromosomal analysis, were collected. Array-CGH was performed to identify subtly acquired genomic CNVs. Regional alterations involving 2p16, 5q13, 17p12, 19q13, 9q34, and 22q11 were observed in this study. Although the span of the aberrations were not completely identical, this finding is similar to those reported previously (Nowak et al., 2010; Nadarajan et al., 2011). Conversely, many alterations were also identified showing little resemblance to other array-CGH analyses in CML patients (Hosoya et al., 2006). The lack of specific recurring lesions might indicate the marked genomic heterogeneity of CML cells and its inherent genomic instability as the disease progresses. The overlapping region of imbalance among the five patients with 9q34 deletions was localized. A recurrent deletion of the ABL1-BCR fusion gene and ASS1 gene, which were located in the 9q33.3-q34.12 region, could be observed in two and three cases, respectively. Other CNVs were randomly distributed on different autosomes.

Loss of 9q34 and 22q11 were the most common alterations and were considered as lesions acquired during the initial genesis of the Ph chromosome. These usually derive from the sequence proximal to ABL1 but distal to BCR in the derivative chromosome 9 [der(9)]. Seen in 10% of CML patients, these variants are associated with poor prognosis (Egan and Radich, 2016). The extent of copy number loss on der(9) varies substantially. A study conducted by Fourouclas et al. (2006) showed that larger deletions (>1.4 Mb) might lead to poorer overall survival because more tumor suppressor genes might be affected. Previous reports have indicated that adverse prognostic effects of ABL1-BCR deletions on derivative chromosome 9 [der(9)] are present in CML patients (Loncarevic et al., 2002). A recent study conducted by Chandran et al. (2018) performed FISH analysis in 489 patients at different stages of CML. Their findings suggest that CML patients in the accelerated phase or in blast crisis (AP/BC) with der(9) deletions show poor response to IM therapy. However, for CML patients in the chronic phase (CP), deletion of ABL1-BCR did not result in poor response to imatinib or lower rates of either complete cytogenetic or major molecular responses (Quintas-Cardama et al., 2011; Svabek et al., 2018). While it suggested that clonal copy number aberrations remain a hallmark of disease progression (Shao et al., 2015), the mechanism of the disease progression has not been fully illustrated. Due to the relatively small sample size in this study, the relationship between CML phase and CNVs cannot be further studied. Further investigations may be needed to determine whether specific CNVs can correctly distinguish CML-CP from CML-AP/BC patients. While an association between deletions in chromosome 22 and prognosis has not been established, it was shown that copy number gain of *GSTT1* in 22q11.23 was frequently observed in patients who did not respond well to the escalated imatinib dose. The gain of *GSTT1* was therefore found to be an independent predictive factor for short, progression-free survival in these patients (Banerjee, 2013).

Fluorescence *in situ* hybridization analysis showed that the *ASS1* gene deletion could be detected in three cases (cases 3, 8, and 10). Expressed in most body tissue, the *ASS1* gene encodes argininosuccinate synthetase-1. Several studies focused on various tumors have shown that *ASS1* may have a tumor suppressor function. Notably, *ASS1* deficiency has been associated with clinically aggressive states of nasopharyngeal carcinoma and pancreatic ductal adenocarcinoma (Lan et al., 2014; Liu et al., 2017). Similarly, patients with urinary bladder high-grade neuroendocrine carcinomas (HGNECs) have shown an absence of *ASS1* expression. Such individuals may therefore be candidates for arginine deprivation therapy using drugs such as ADI-PEG 20 (Gupta et al., 2018). However, *ASS1* also has oncogenic potential in gastric cancer and hepatocellular adenoma (Henriet et al., 2017; Tsai et al., 2018). Rabinovich et al. (2015) demonstrated that the decreased activity of *ASS1* in cancer increases cytosolic aspartate levels. As a result, this increases carbamoyl-phosphate synthase 2, aspartate transcarbamylase, and dihydroorotase complex (CAD) activation by upregulating the availability of its substrate and by increasing its phosphorylation by S6K1 through the mammalian target of rapamycin (mTOR) pathway. Therefore, by blocking citrin and decreasing CAD activity, mTOR signaling or pyrimidine synthesis decreases proliferation. This may serve as a therapeutic strategy in multiple cancers where *ASS1* is downregulated. Collectively, the effect of the *ASS1* deletion in CML should be further considered.

Another related gene involved in the deletion region of the above three cases is *PRDM1*2 which encodes a member of a family of transcriptional regulators that participate in the control of vertebrate neurogenesis (Mzoughi et al., 2016). Reid and Nacheva (2004) identified a patient with an aggressive form of CML harboring a submicroscopic insertion of sequences from chromosome 9q34.1 into chromosome 7q35. The *PRDM12* and *FUBP3* genes were included in this insertion. It was therefore hypothesized that loss of *PRDM12* expression could be responsible for the aggressive state of the disease in this patient.

In case 9, the size of the 17p11.2 deletion was 0.61 Mb and affected five genes (*FLCN, TNFRSF13B, MPRIP, COPS3*, and *PLD6*). Birt-Hogg-Dube disease is caused by mutations in the folliculin (*FLCN*) gene through which 30–45% of cases develop renal cell carcinoma (RCC). While the tumor suppressor activity of *FLCN* was confirmed by nude mouse xenograft assays of two human RCC cell lines with either diminished or re-expressed *FLCN* (Hudon et al., 2010), a recent report showed a 14-year-old patient with early-onset bulky RCC harboring a deletion in *FLCN* exon 5 (Schneider et al., 2018). Furthermore, somatic frameshift mutations in the *FLCN* exon 11 C(8) mononucleotide tract were detected in more than 20% of colorectal cancers, suggesting that *FLCN* inactivation might contribute to colorectal tumorigenesis (Nahorski et al., 2010). However, while the molecular functions of *FLCN* are poorly understood, indirect interactions between *FLCN* and 5′ AMP-activated protein kinase (AMPK) in the mTOR complex 1 (mTORC1) signaling networks have been firmly established. These interactions are known to be mediated by the novel *FLCN* -interacting proteins 1 and 2 (*FNIP1* and *FNIP2*; Baba et al., 2006). Although, several kinds of tyrosine kinase inhibitors are widely used in the treatment of CML, a proportion of patients may still develop into BCR-ABL-independent resistance. Potentially contributing to chemotherapy resistance, constitutive activation of the phosphatidylinositol 3-kinase/protein kinase B/mammalian target of rapamycin (PI3K/Akt/mTOR) signaling pathway has been observed in CML (Dinner and Platanias, 2016). A recent study showed that combined mTOR and autophagy inhibition may provide an attractive approach to target a BCR-ABL-independent mechanism of resistance (Mitchell et al., 2018). It would be an interesting point for further investigation.

In case 8, the loss of 9q33.3 was observed and affected the RALFPS1 and ANGPTL2 genes. ANGPTL2 contributes to proliferation and invasion of gastric cancer cells (Sheng et al., 2016). Since serum ANGPTL2 levels were significantly increased in patients with non-small cell lung cancer, it could serve as a novel potential diagnostic and prognostic biomarker (Chen et al., 2017). Angptl2 fosters the polarization of tumor-associated macrophages (TAMs) through the p65 nuclear factor-kappa B (NF-κB) pathway and functional M2 phenotype (Wei et al., 2017). Deng et al. (2014) revealed that Angptl2 binds to leukocyte immunoglobulin (Ig)-like receptor B2 (LILRB2) and activates subsequent downstream signaling. This has enabled the development of a new approach for *ex vivo* expansion of human HSC and leukemia development, which might have great value for future therapy.

## Conclusion

In summary, the findings demonstrate that CNVs are relatively common in CML patients with a pure Ph+ chromosome. These cryptic genomic changes were genetically identified by array-CGH and harbored tumor-related genes which may be drivers of disease progression in CML for individual patients. This, followed up with targeted gene studies, should be further evaluated for clinical significance and influence on therapeutic approaches which may open many avenues for research into clinical studies and the behavior of CML.

## Data Availability Statement

The data that support the findings of this study are available from the corresponding author upon reasonable request.

## Ethics Statement

The studies involving human participants were reviewed and approved by the Institutional Review Board (IRB) of the Oklahoma University (IRB number: 2250). The patients/participants provided their written informed consent to participate in this study.

## Author Contributions

SL and LZ conceived and supervised the study. XWx obtained the financial support. HZ, XXW, ML, and YR involved in analysis and interpretation of the data, and drafting the manuscript. YK and XFW performed the array-CGH. XL, HP, GL, YG, MS, YS, CZ, YZ, and JZ carried out the FISH analysis. SL, LZ, XXW, and HZ revised the manuscript. All authors read and approved the final manuscript.

## Conflict of Interest

The authors declare that the research was conducted in the absence of any commercial or financial relationships that could be construed as a potential conflict of interest.

## Publisher’s Note

All claims expressed in this article are solely those of the authors and do not necessarily represent those of their affiliated organizations, or those of the publisher, the editors and the reviewers. Any product that may be evaluated in this article, or claim that may be made by its manufacturer, is not guaranteed or endorsed by the publisher.
