# Supplementary material for: Genomic Copy Number Variants in CML Patients With the Philadelphia Chromosome (Ph+): An Update
Source: Front Genet. 2021 Aug 10;12:697009. doi: 10.3389/fgene.2021.697009 (PMC8383316; doi:10.3389/fgene.2021.697009)
Supplement: Supplementary file 1 [file Data_Sheet_1.PDF]

Sample Information

Array ID	:	252185023391_1_2
Global Display Name	:	13-1264-FM-252185023391_1_2
Green Sample	:	
Red Sample	:	
Polarity	:	1
DerivativeOfLogRatioSD	:	0.128289
Intermediate Report by	:	OUHSC\xwang3

This is an intermediate report and not a final signed off report

Genome View (Amp/Del)

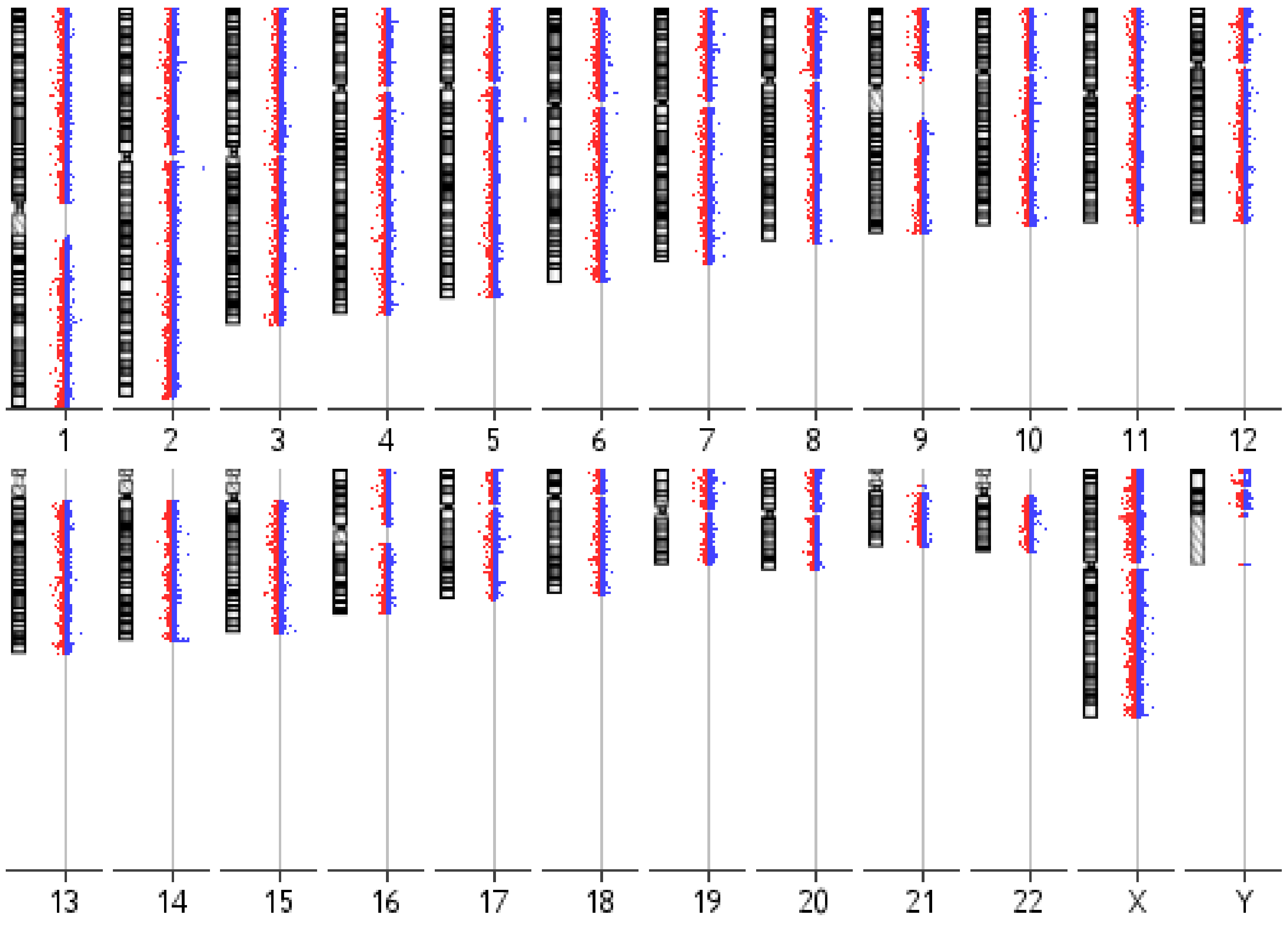

This is an intermediate report and not a final signed off report

Chromosome Views (Amp/Del)

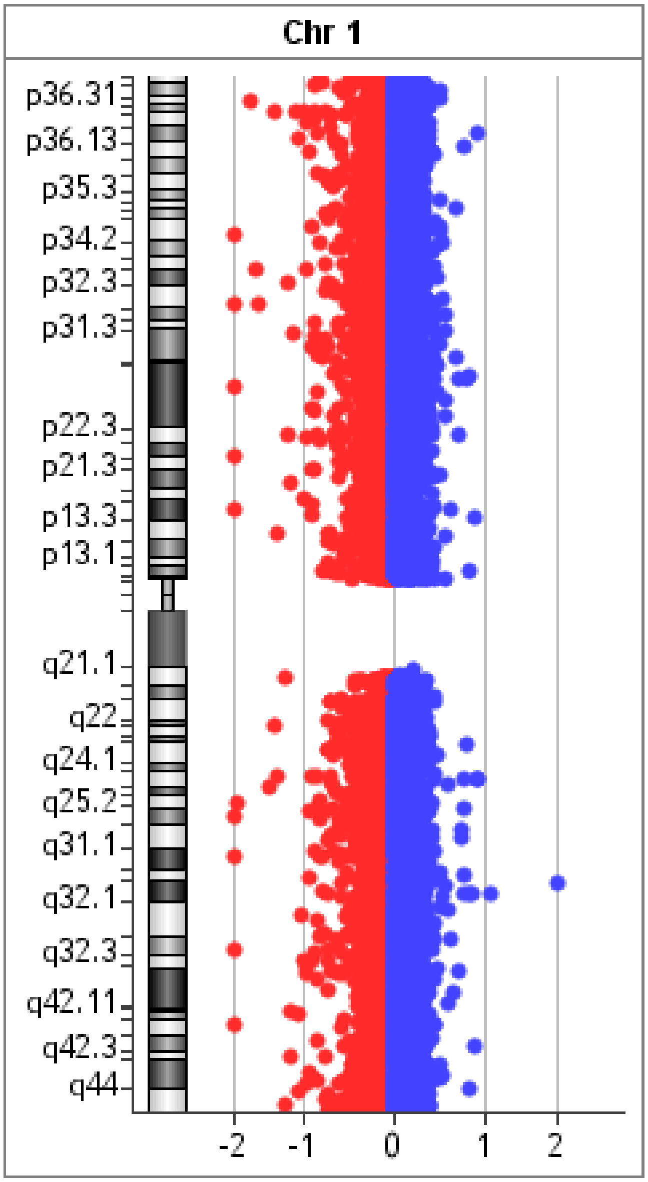

This is an intermediate report and not a final signed off report

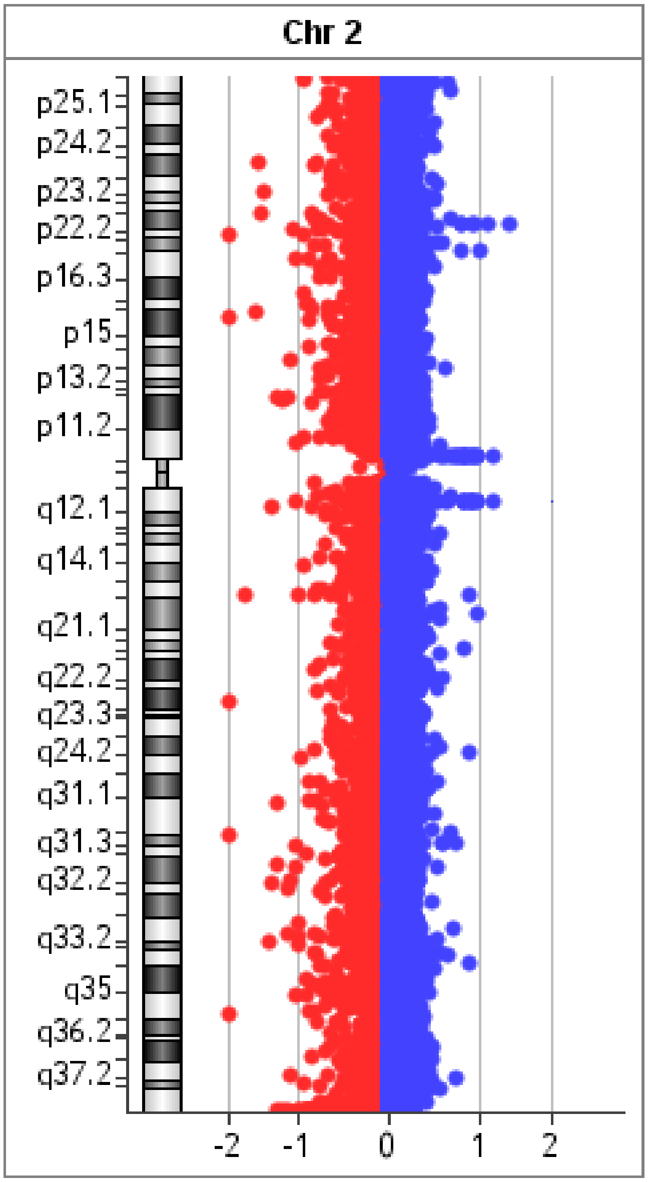

This is an intermediate report and not a final signed off report

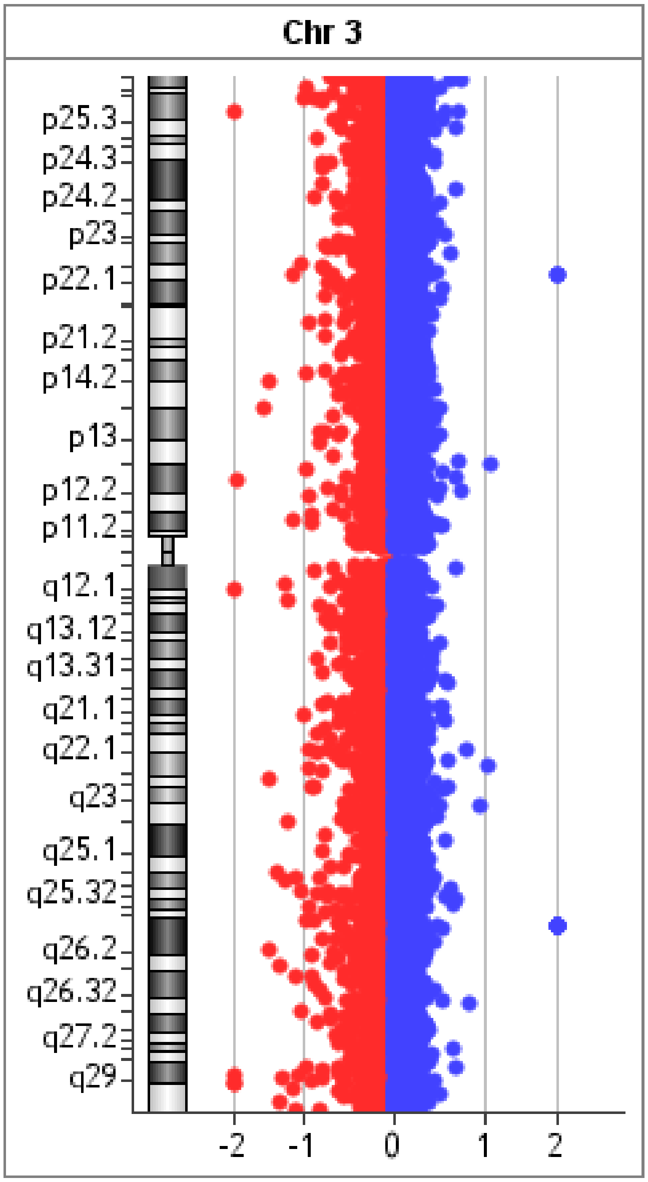

This is an intermediate report and not a final signed off report

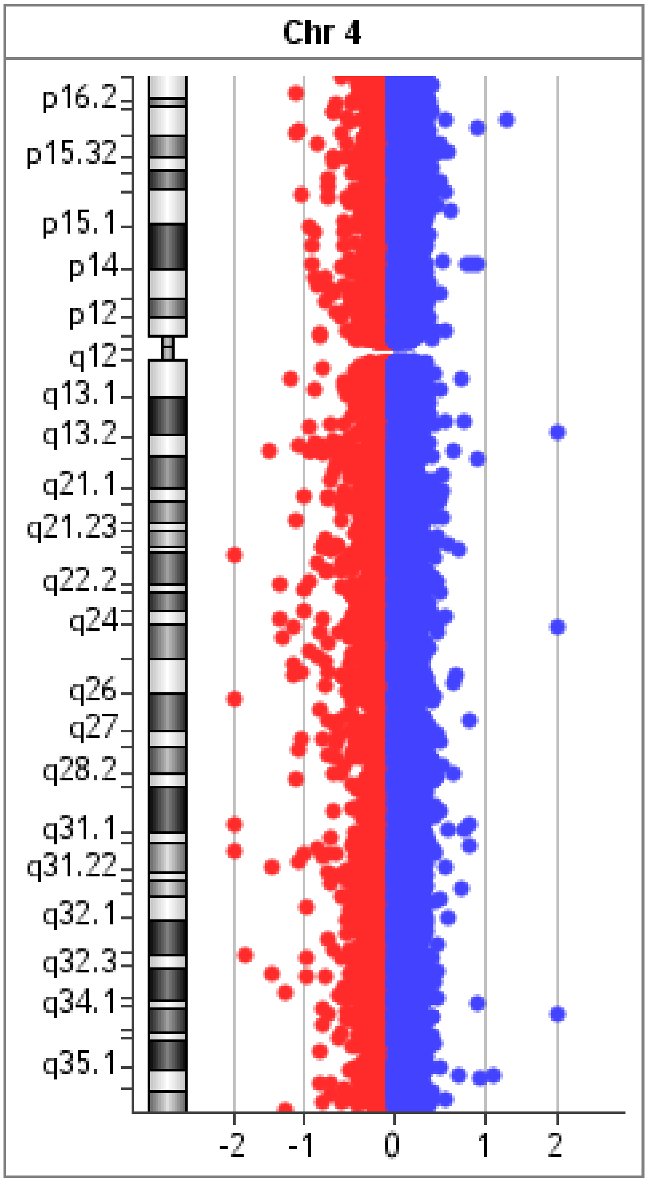

This is an intermediate report and not a final signed off report

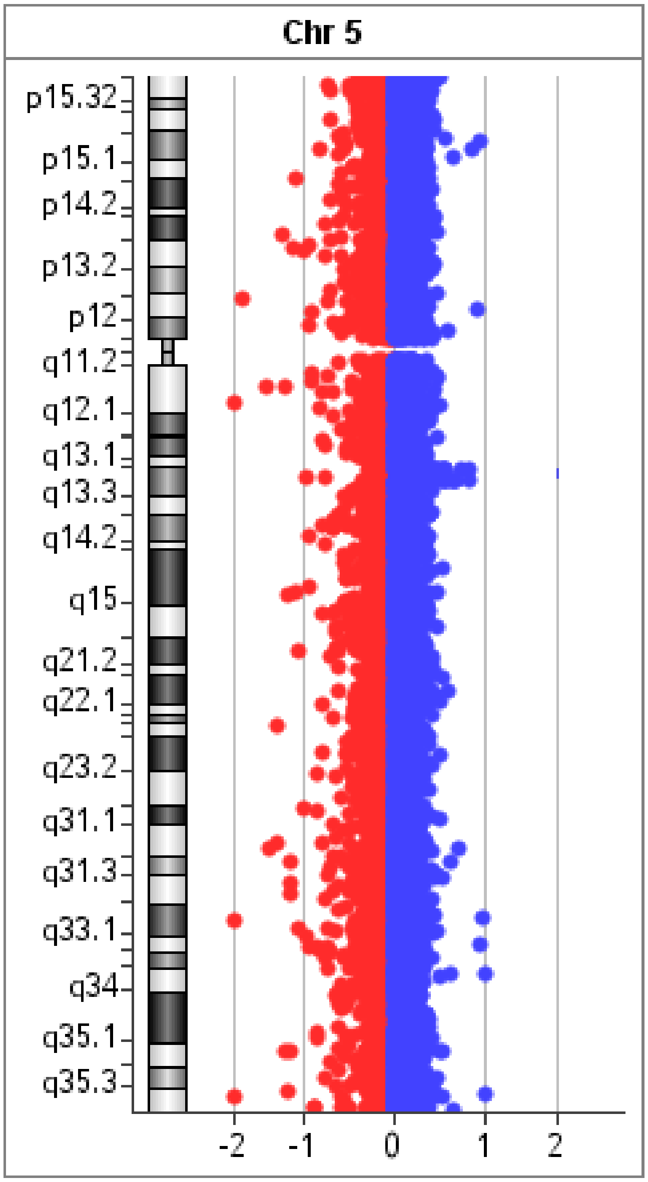

This is an intermediate report and not a final signed off report

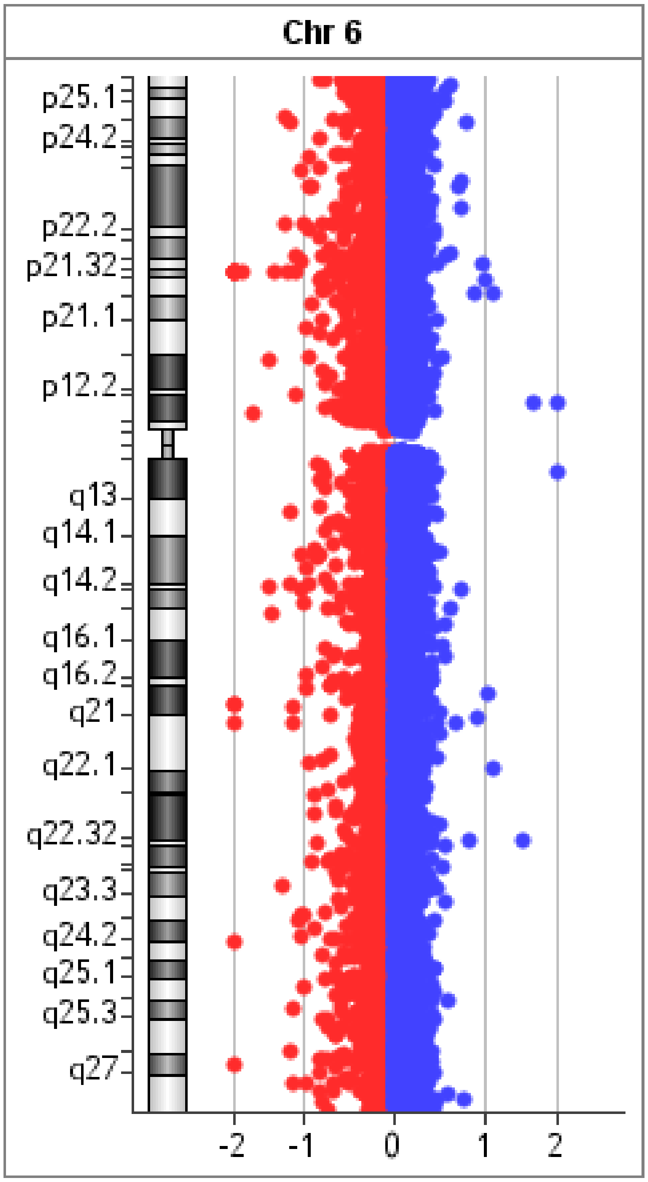

This is an intermediate report and not a final signed off report

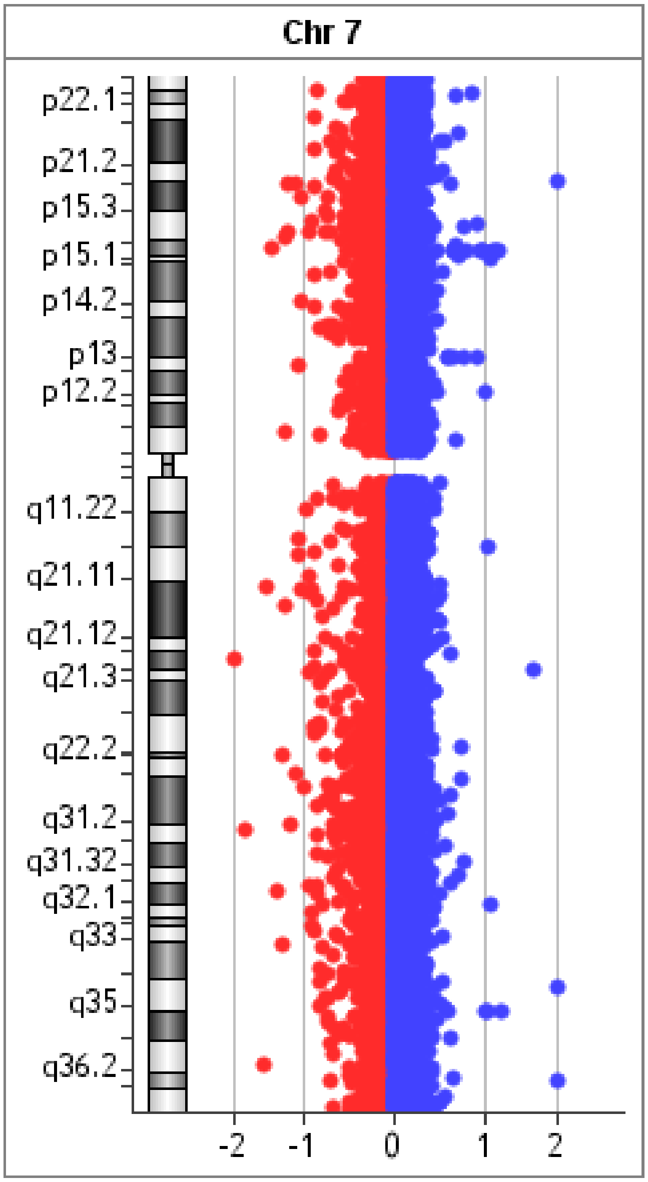

This is an intermediate report and not a final signed off report

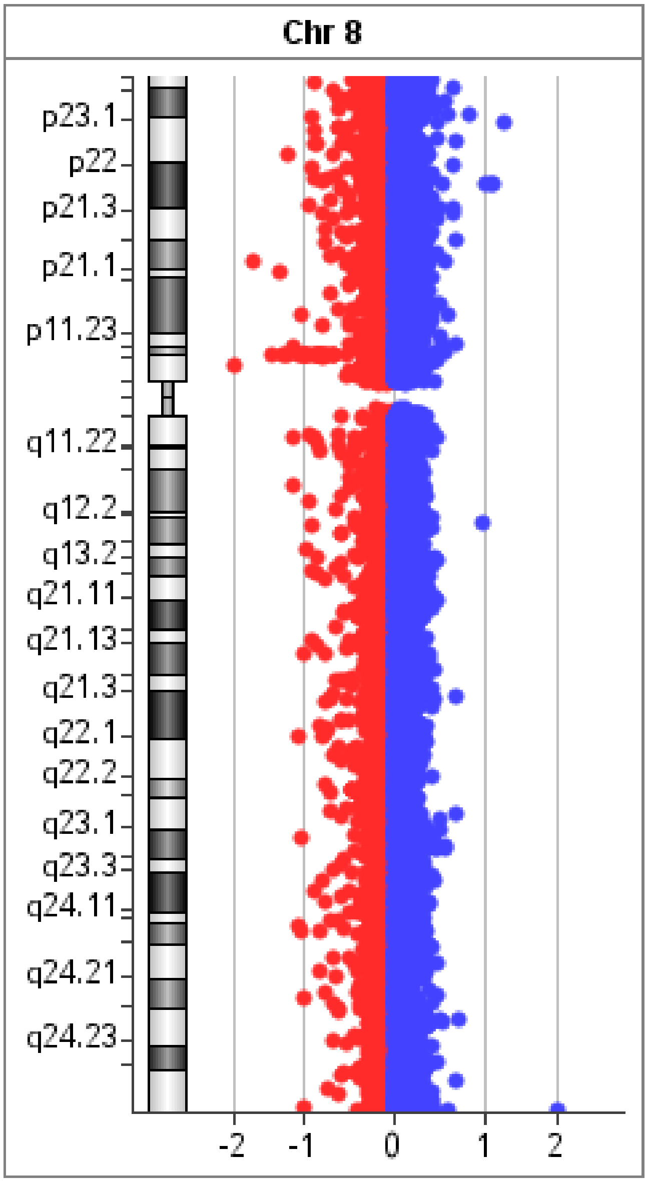

This is an intermediate report and not a final signed off report

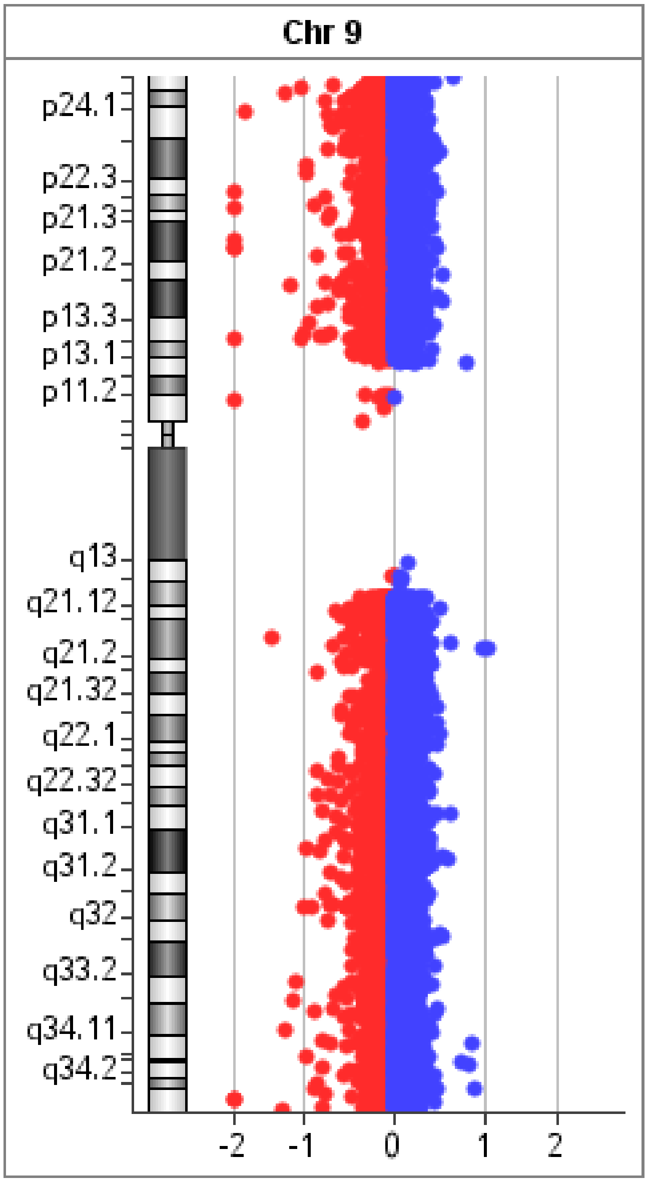

This is an intermediate report and not a final signed off report

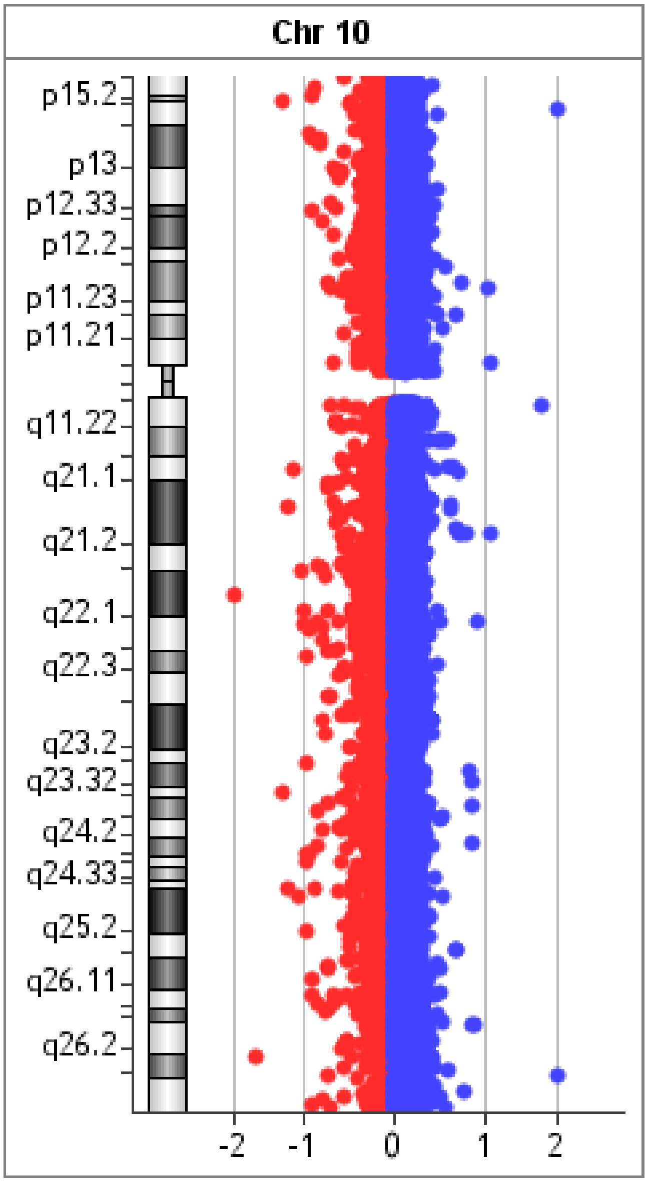

This is an intermediate report and not a final signed off report

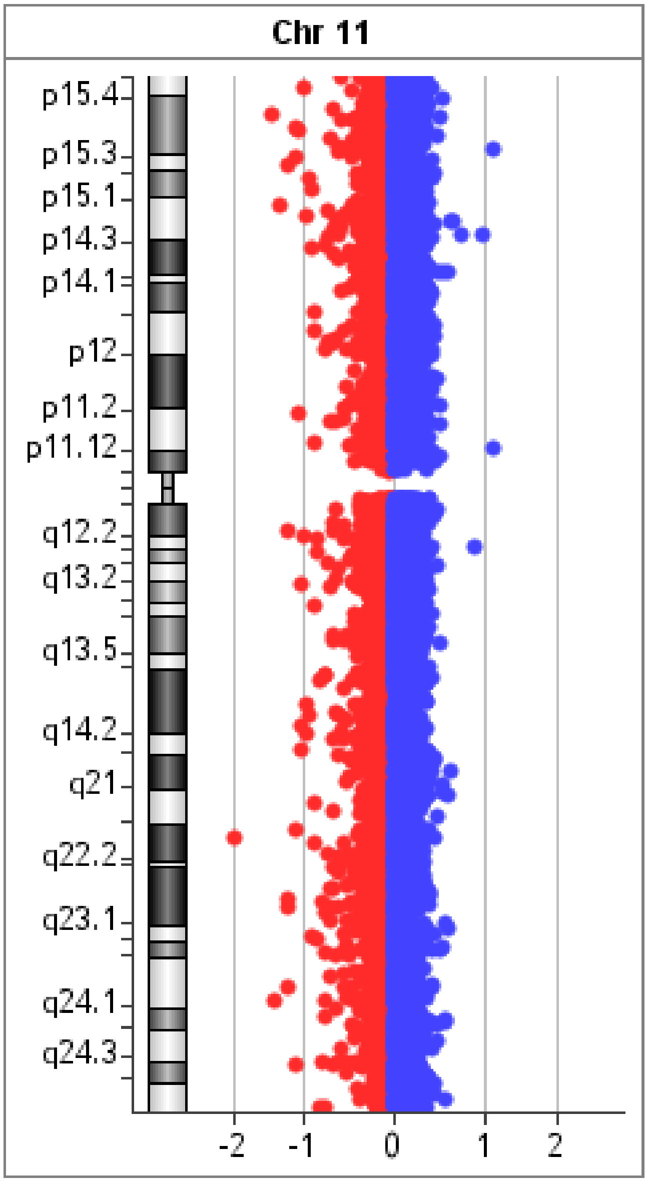

This is an intermediate report and not a final signed off report

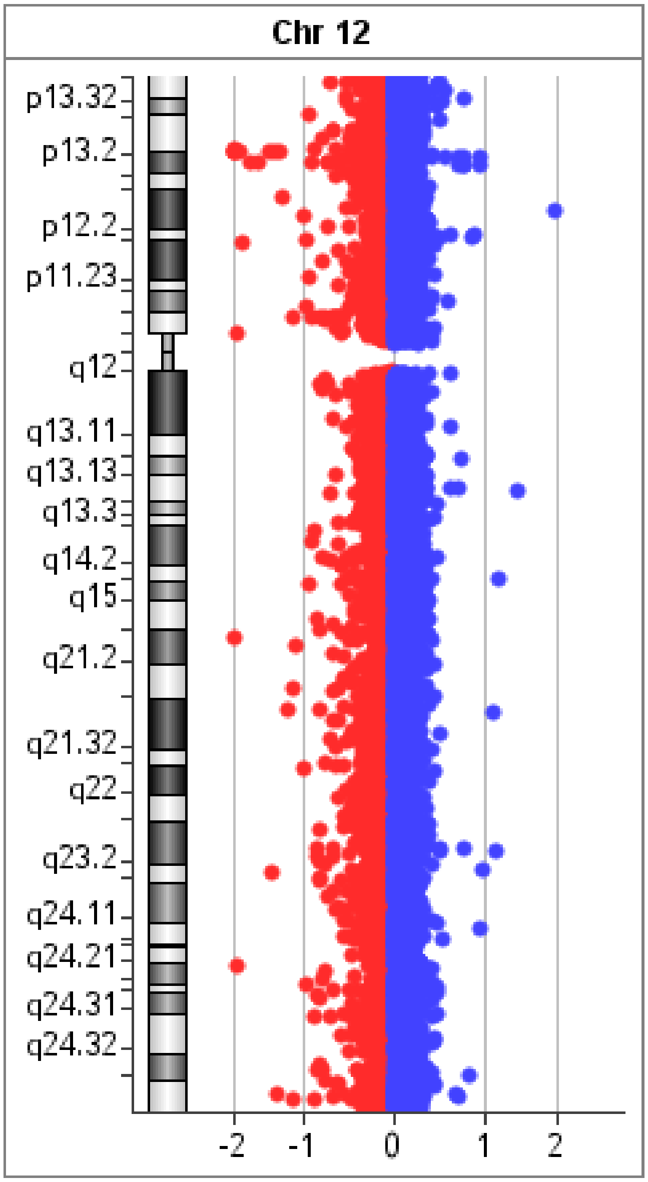

This is an intermediate report and not a final signed off report

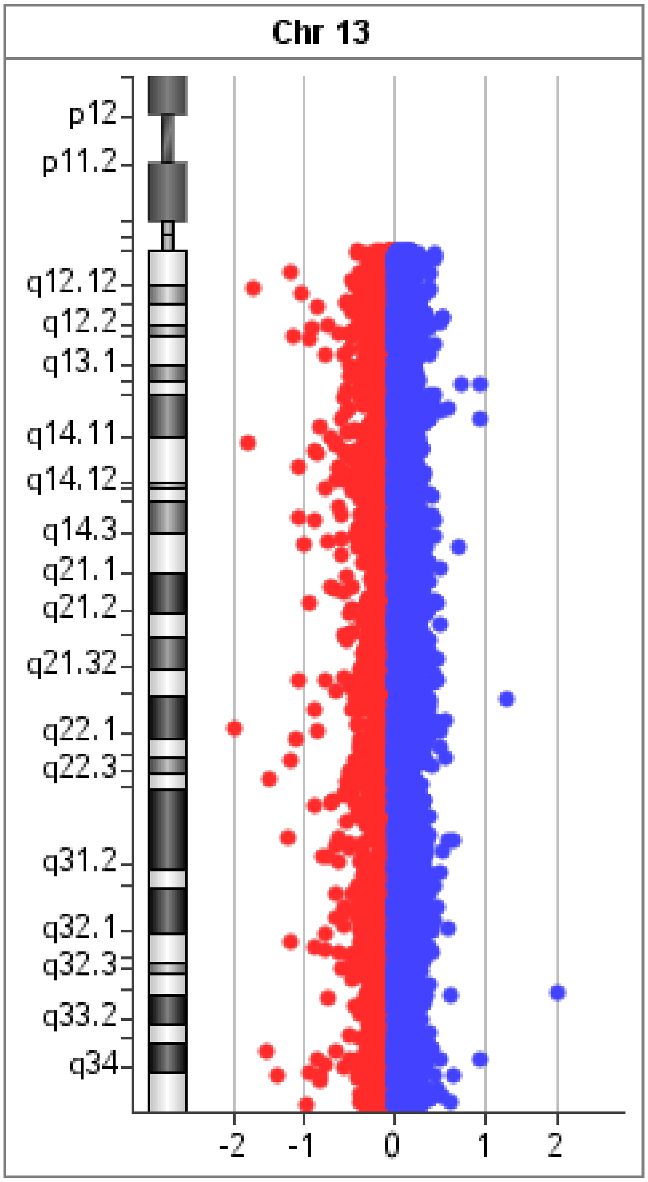

This is an intermediate report and not a final signed off report

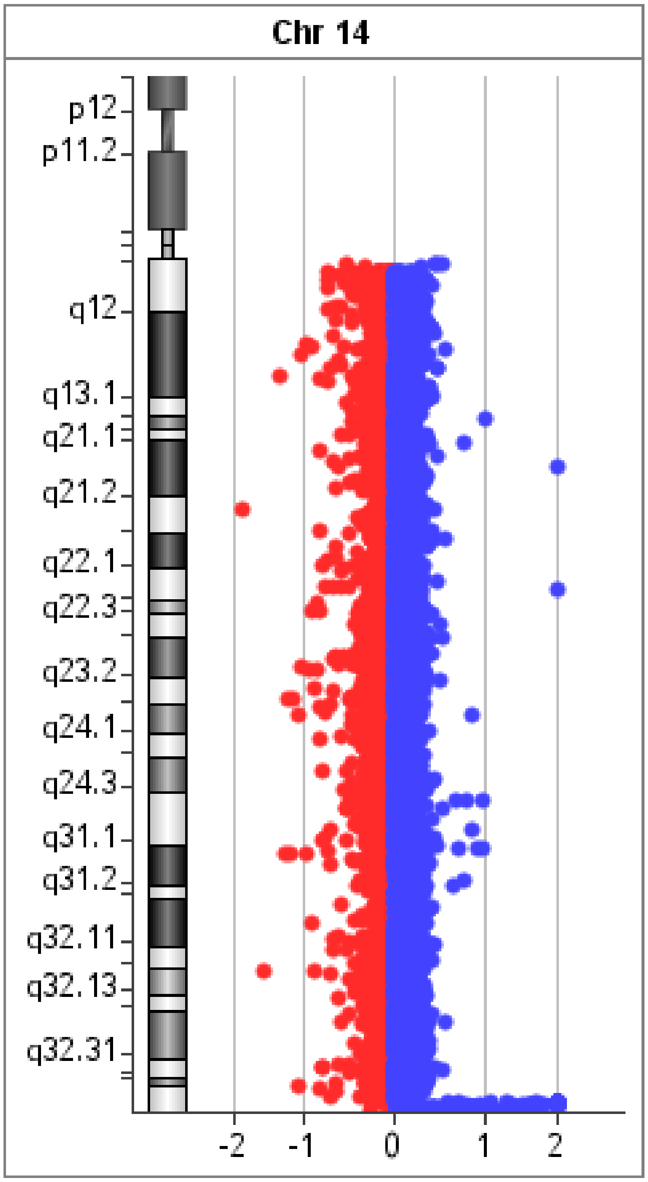

This is an intermediate report and not a final signed off report

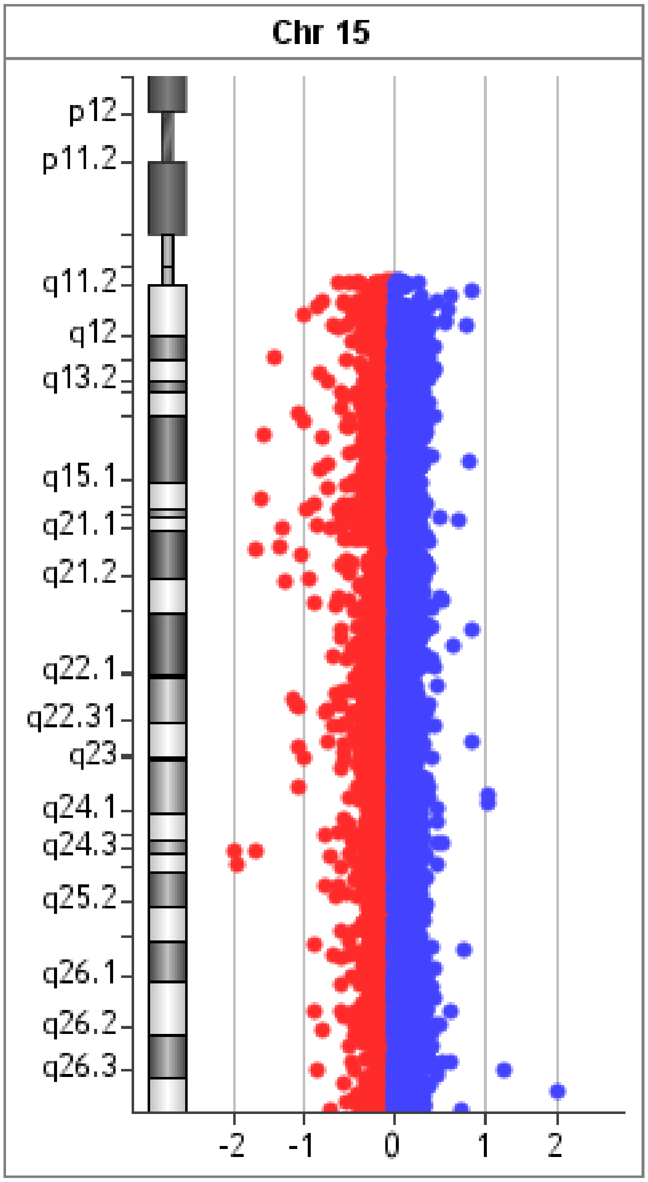

This is an intermediate report and not a final signed off report

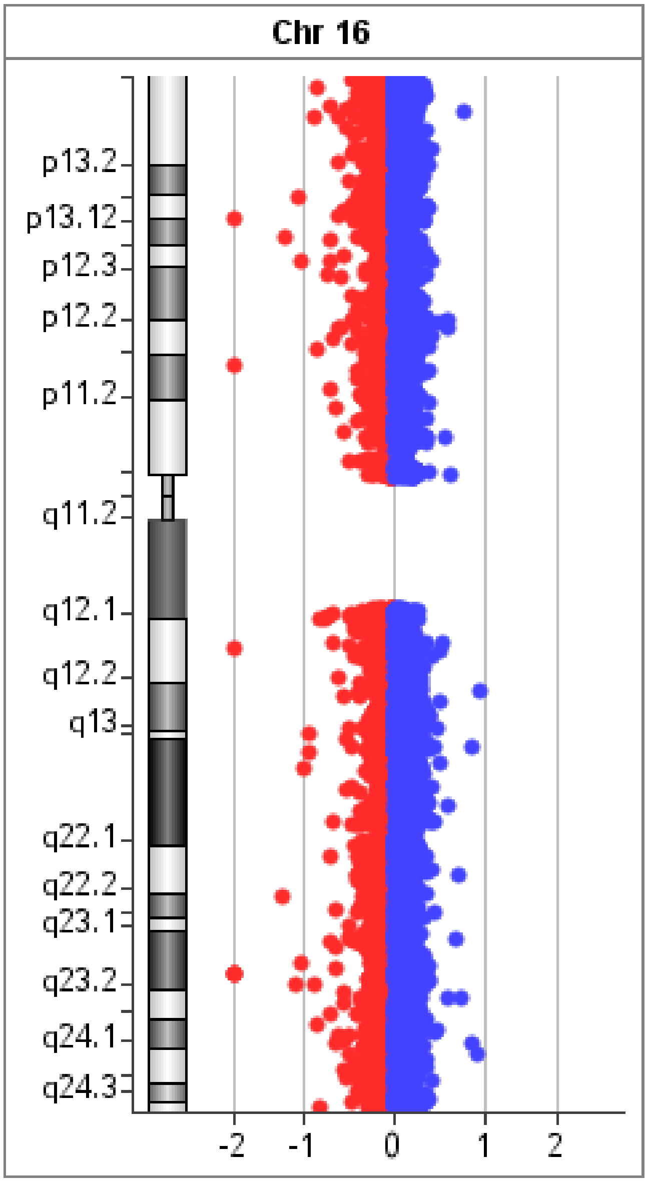

This is an intermediate report and not a final signed off report

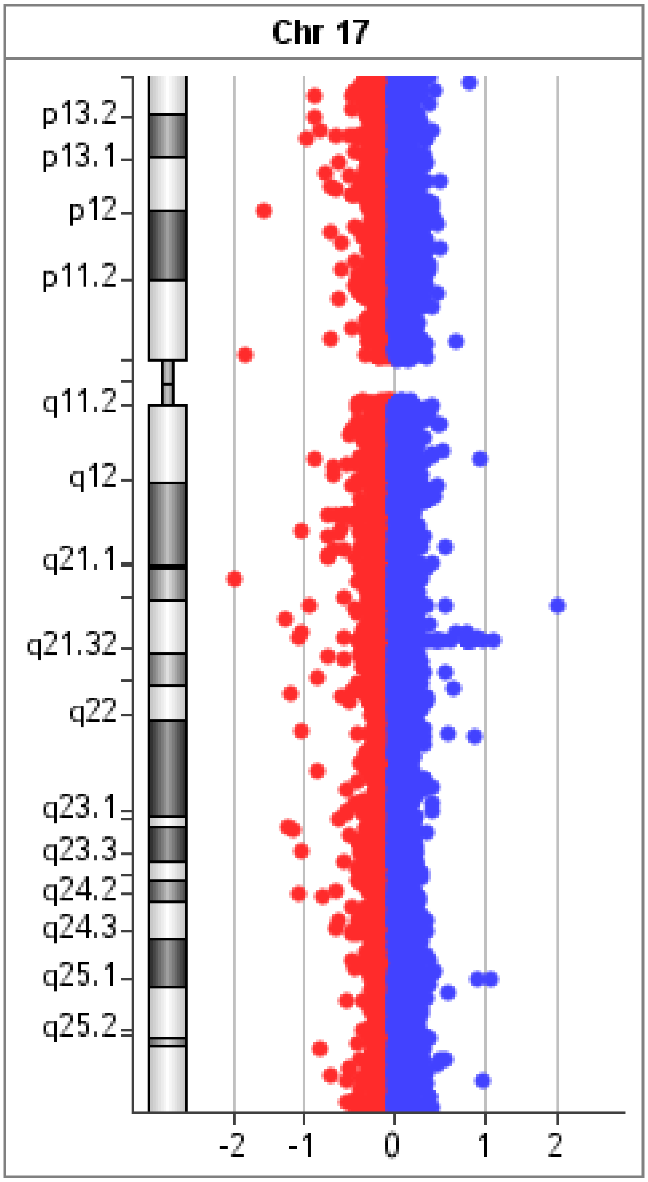

This is an intermediate report and not a final signed off report

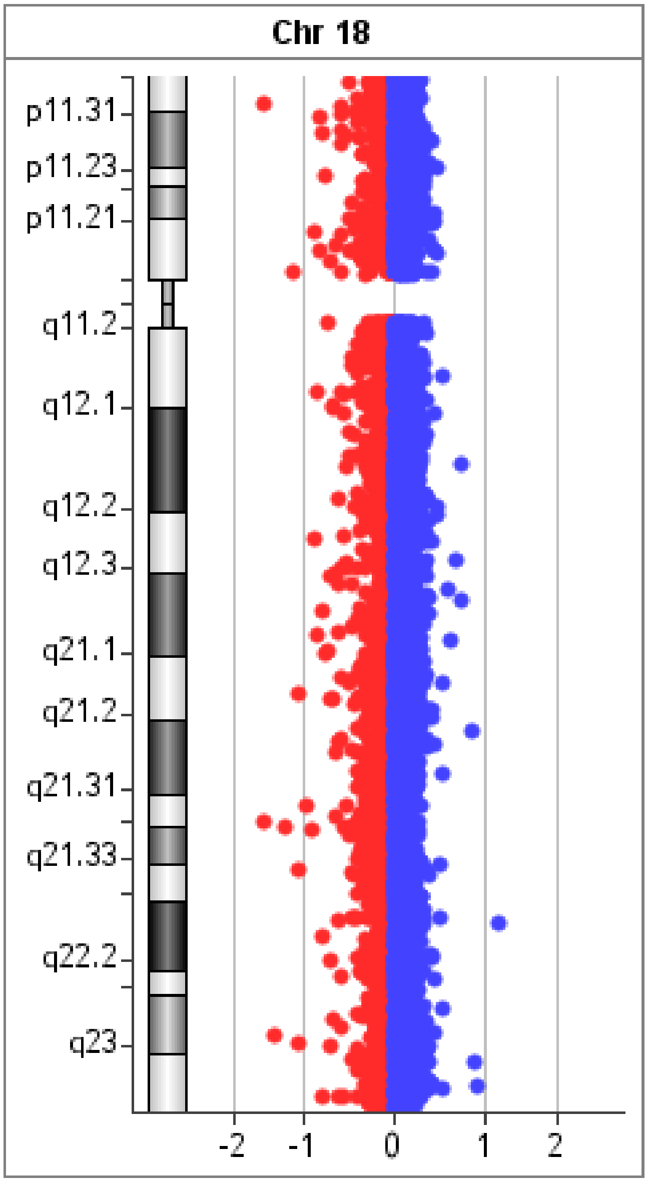

This is an intermediate report and not a final signed off report

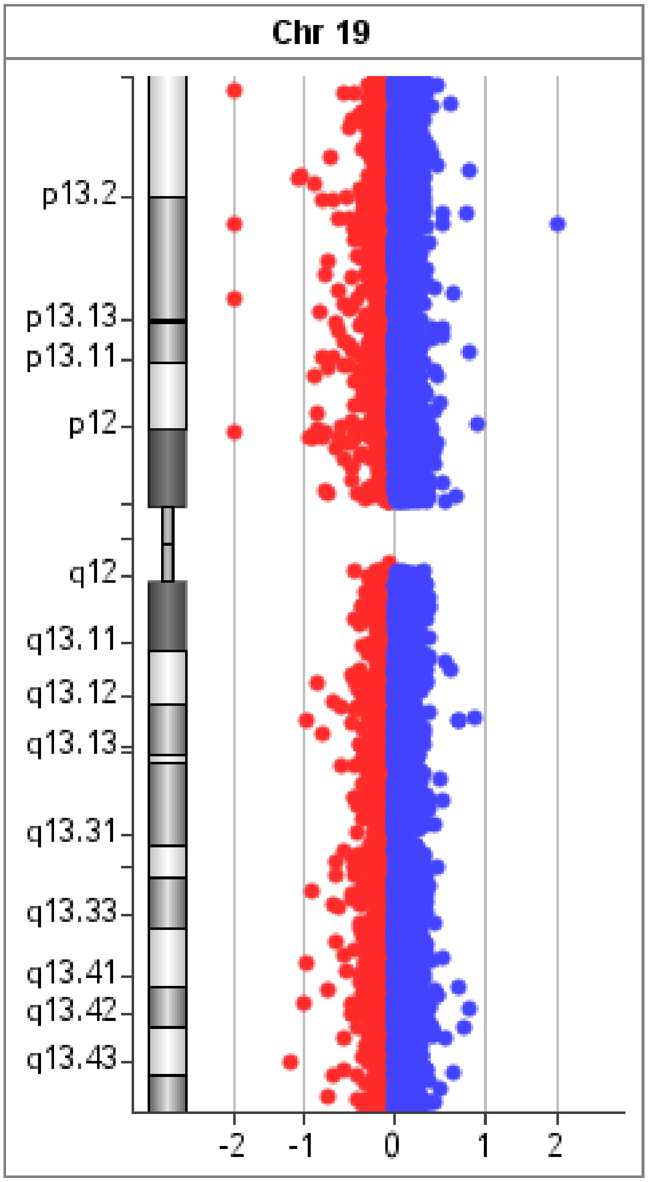

This is an intermediate report and not a final signed off report

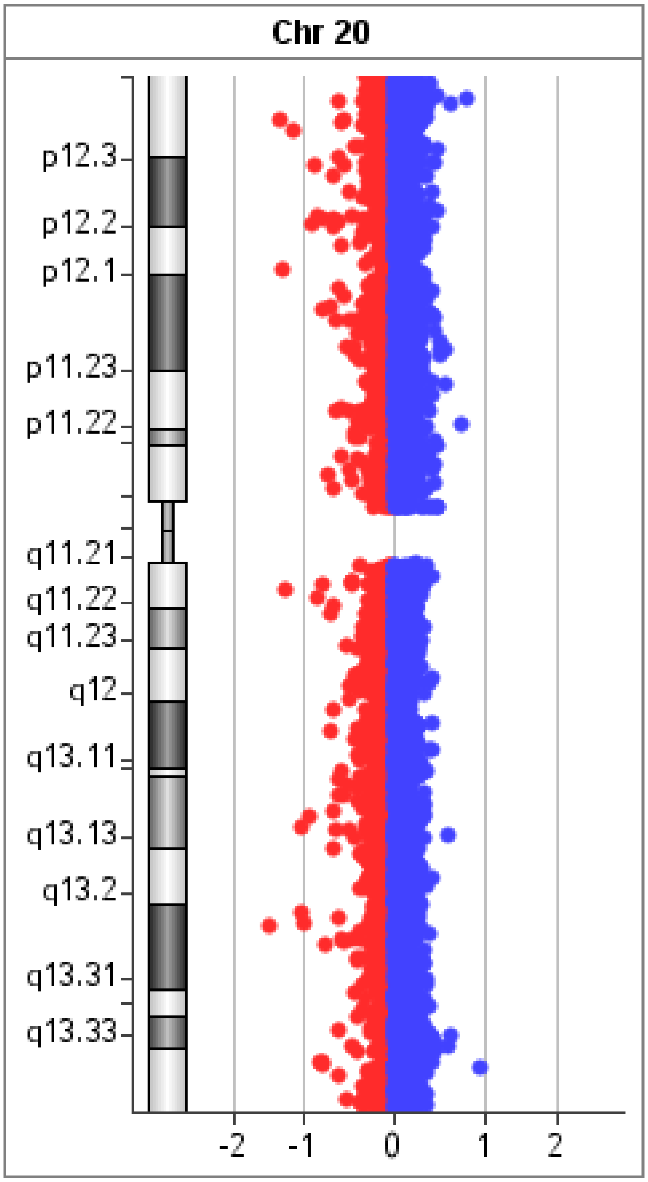

This is an intermediate report and not a final signed off report

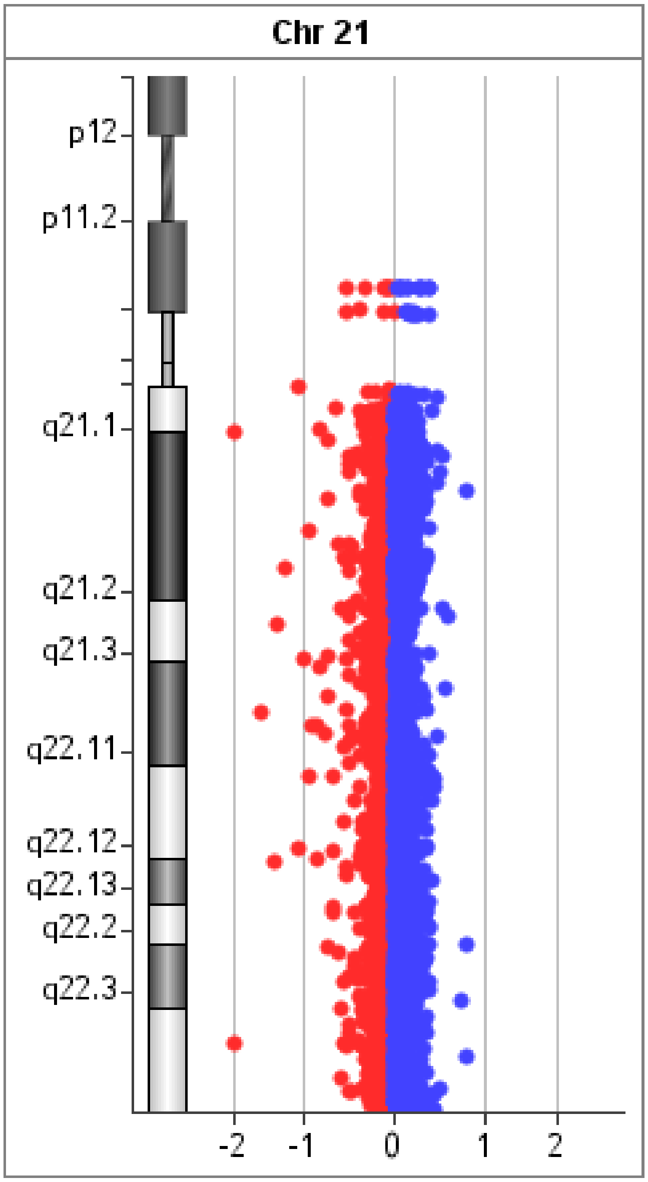

This is an intermediate report and not a final signed off report

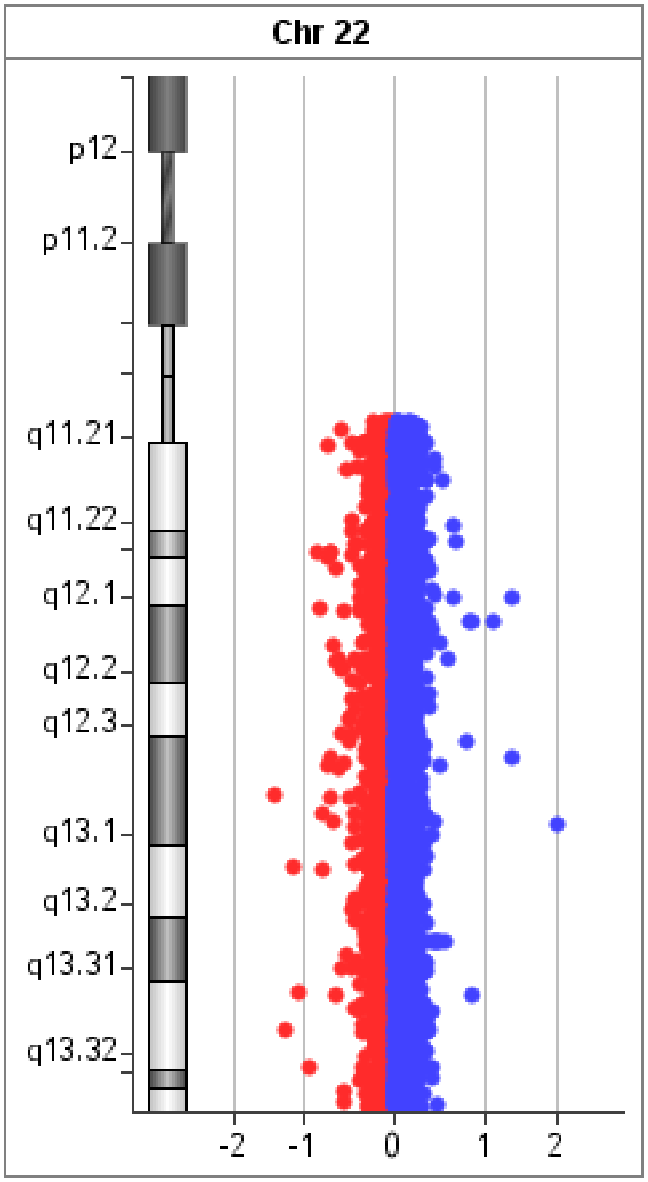

This is an intermediate report and not a final signed off report

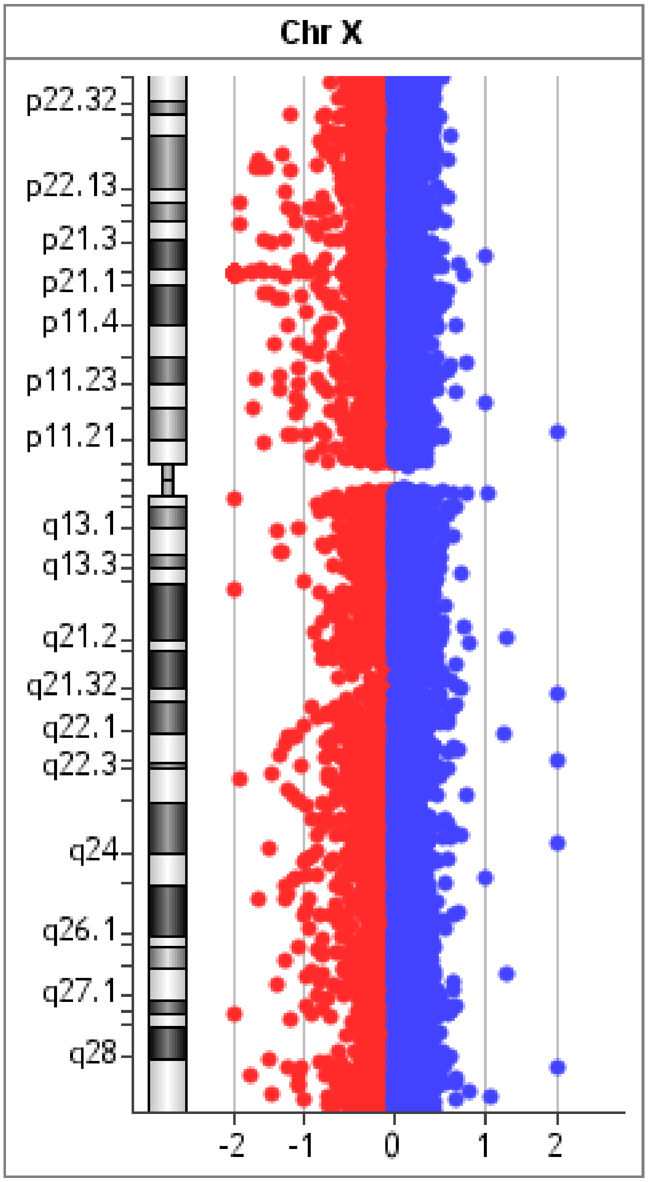

This is an intermediate report and not a final signed off report

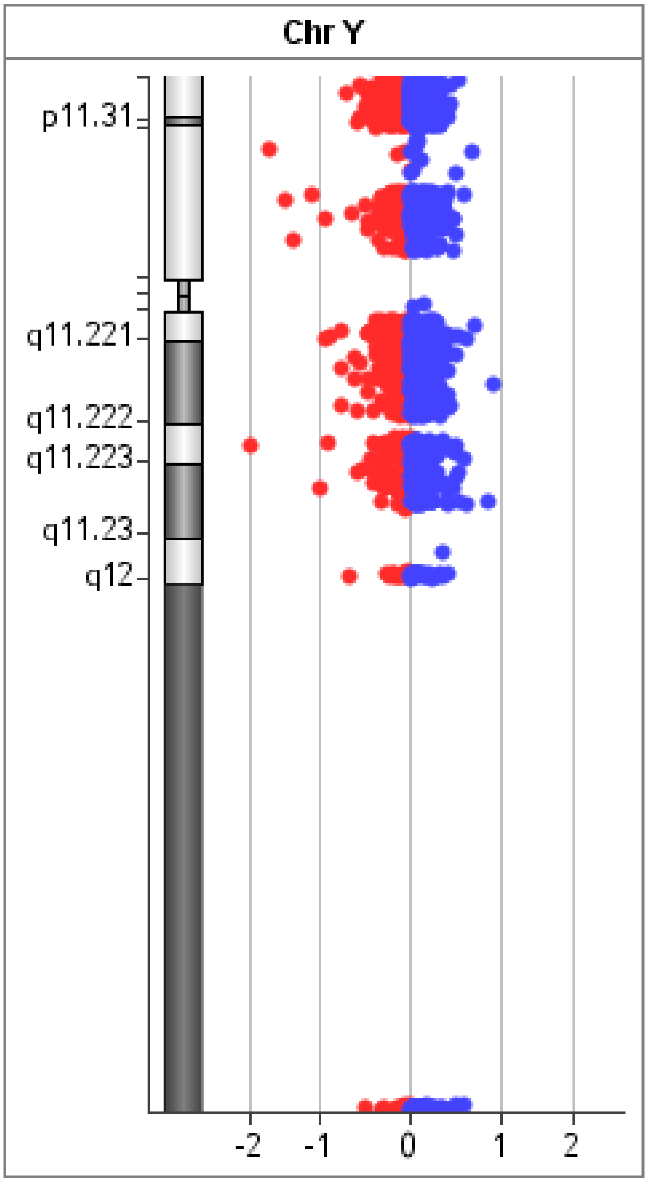

This is an intermediate report and not a final signed off report

Amp/Gain/Loss/Del Intervals Table

Chr	Start-Stop(bp)	Cytoband	Size(kb)	#Probes	Amp/Gain/Loss/Del	Annotations	Classifications
chr2	99833970-99915014	q11.2	81.045	16	0.823	LYG2, LYG1, nsv874698...	
chr5	68728731-70856107	q13.2	2,127.377	49	0.321	MARVELD2, LOC101928924, OCLN...	
chrX	29538933-29645698	p21.2	106.766	22	-2.077	IL1RAPL1, MIR4666B, nsv523313...	

Amp=Amplification Del=Deletion

Total Amp/Gain/Loss/Del Intervals: 3

This is an intermediate report and not a final signed off report

ISCN Nomenclature

arr[GRCh37] 2q11.2(99833970_99915014)x4
arr[GRCh37] 5q13.2(68728731_70856107)x3

This is an intermediate report and not a final signed off report

Analysis Settings

Design	021850_20150623	Sample Name	13-1264-FM-25218502339 1_1_2
Genome	hg19	Aberration Algorithm	ADM-2
Threshold	6.0	Fuzzy Zero	OFF
GC Correction	ON	Window Size	2Kb
Centralization (legacy)	OFF	Diploid Peak Centralization	ON
SNP Copy Number	OFF	LOH	OFF
Combine Replicates (Intra Array)	ON	Array Level Filter	NONE
Metric Set Filter	NONE	Aberration Filter Name	Default Aberration Filter
Aberration Filter	Minimum Number of Probes for Amplification >= 3 AND Nesting Level <= 100 AND Minimum Avg. Absolute Log Ratio for Amplification >= 0.25 AND Minimum Size (Kb) of Region for Amplification >= 0.0 AND Minimum Size (Kb) of Region for Deletion >= 0.0 AND Minimum Number of Probes for Deletion >= 3 AND Minimum Avg. Absolute Log Ratio for Deletion >= 0.25 AND Minimum Number of Probes for Gain >= 3 AND Minimum Number of Probes for Loss >= 3 AND Minimum Avg. Absolute Log Ratio for Gain >= 0.25 AND Minimum Avg. Absolute Log Ratio for Loss >= 0.25 AND Minimum Size (Kb) of Region for Gain >= 0.0 AND Minimum Size (Kb) of Region for Loss >= 0.0	Feature Level Filter	gIsSaturated = true OR rIsSaturated = true OR gIsFeatNonUnifOL = true OR rIsFeatNonUnifOL = true OR LogRatio = 0; Include matching values=false
Design Level Filter	Homology = 0 OR IsPseudoautosomal = 1	LOH Filter	NONE
Genomic Boundary	OFF	Show Flat Intervals	false
Template Name	ouhsc-cgh		

Genomic region filter for Aberration: OFF

Genomic region filter for LOH: OFF

Notes

Sample Notes

No notes available.

This is an intermediate report and not a final signed off report

Amp/Gain/Loss/Del Interval Notes

No notes available.

This is an intermediate report and not a final signed off report
