# Supplementary material for: Genomic Copy Number Variants in CML Patients With the Philadelphia Chromosome (Ph+): An Update
Source: Front Genet. 2021 Aug 10;12:697009. doi: 10.3389/fgene.2021.697009 (PMC8383316; doi:10.3389/fgene.2021.697009)
Supplement: Supplementary file 2 [file Data_Sheet_2.PDF]

Sample Information

DerivativeOfLogRatioSD	:	0.143907
Red Sample	:	
Polarity	:	1
Global Display Name	:	252185022956_1_2
Array ID	:	252185022956_1_2
Green Sample	:	Agilent Euro Male
Intermediate Report by	:	OUHSC\xwang3

This is an intermediate report and not a final signed off report

Genome View (Amp/Del)

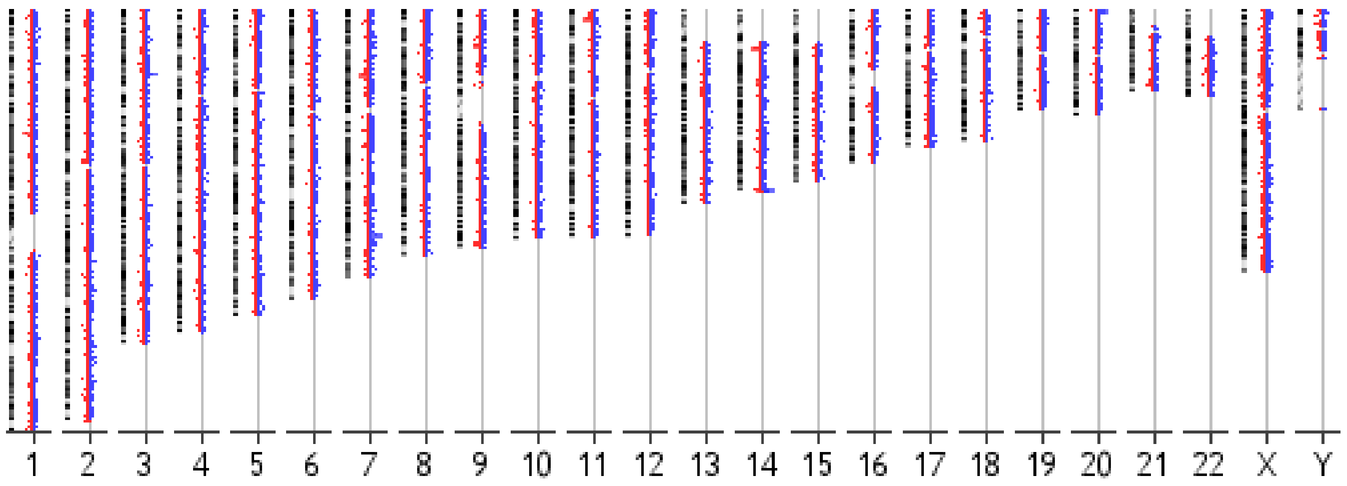

This is an intermediate report and not a final signed off report

Chromosome Views (Amp/Del)

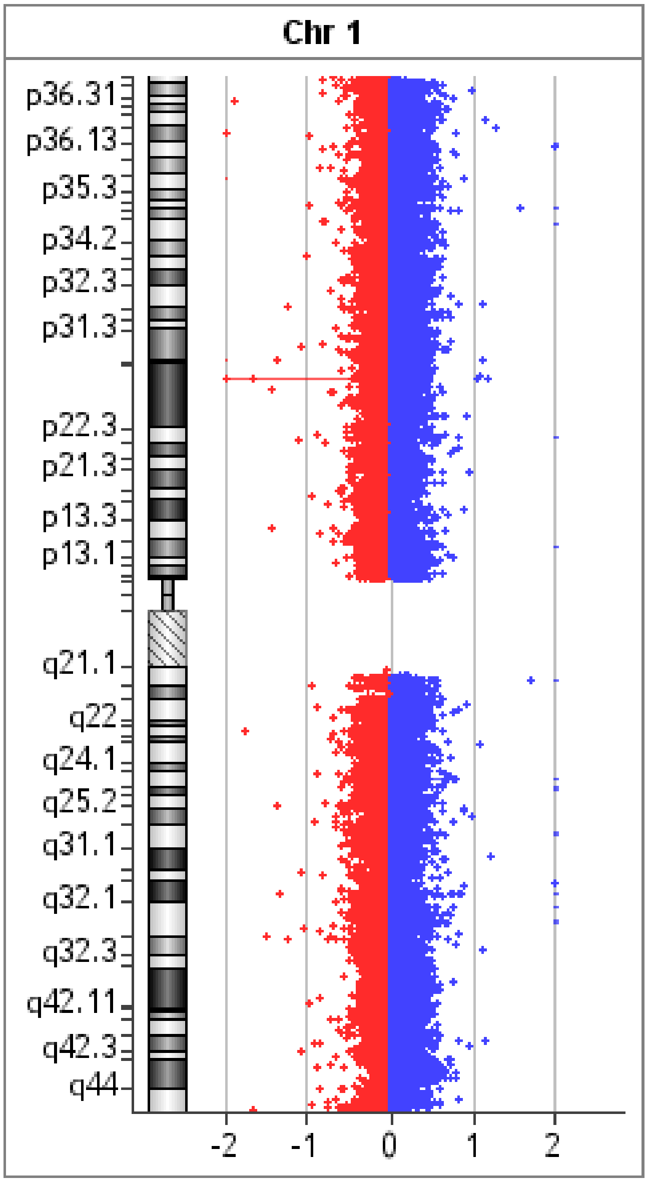

This is an intermediate report and not a final signed off report

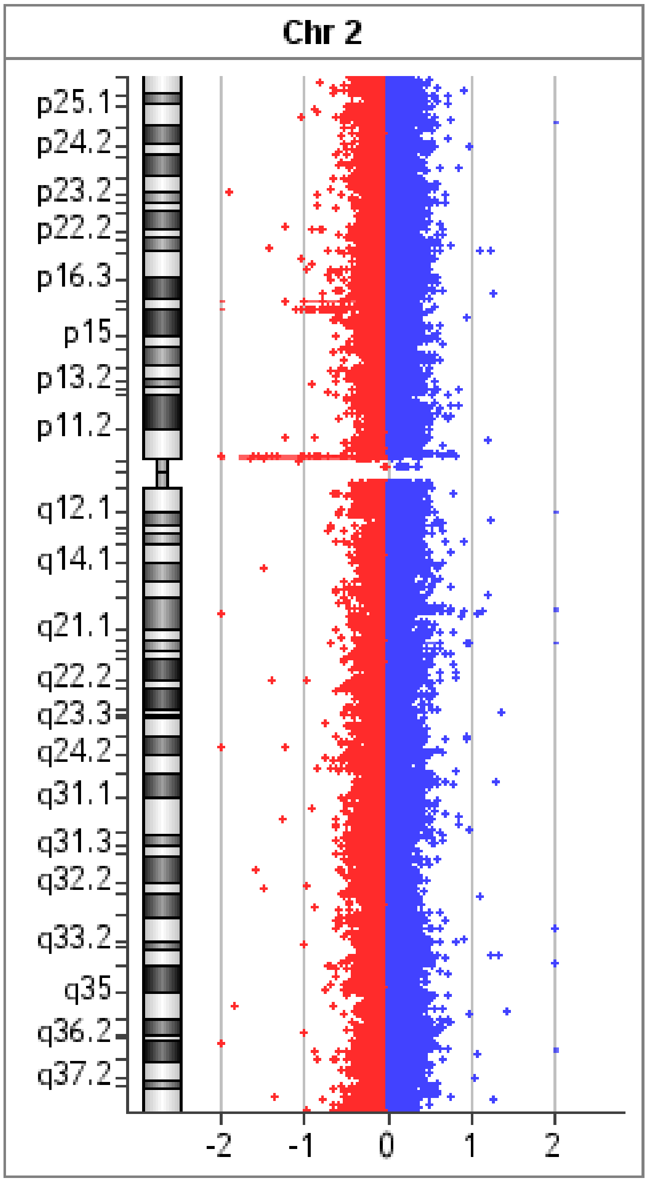

This is an intermediate report and not a final signed off report

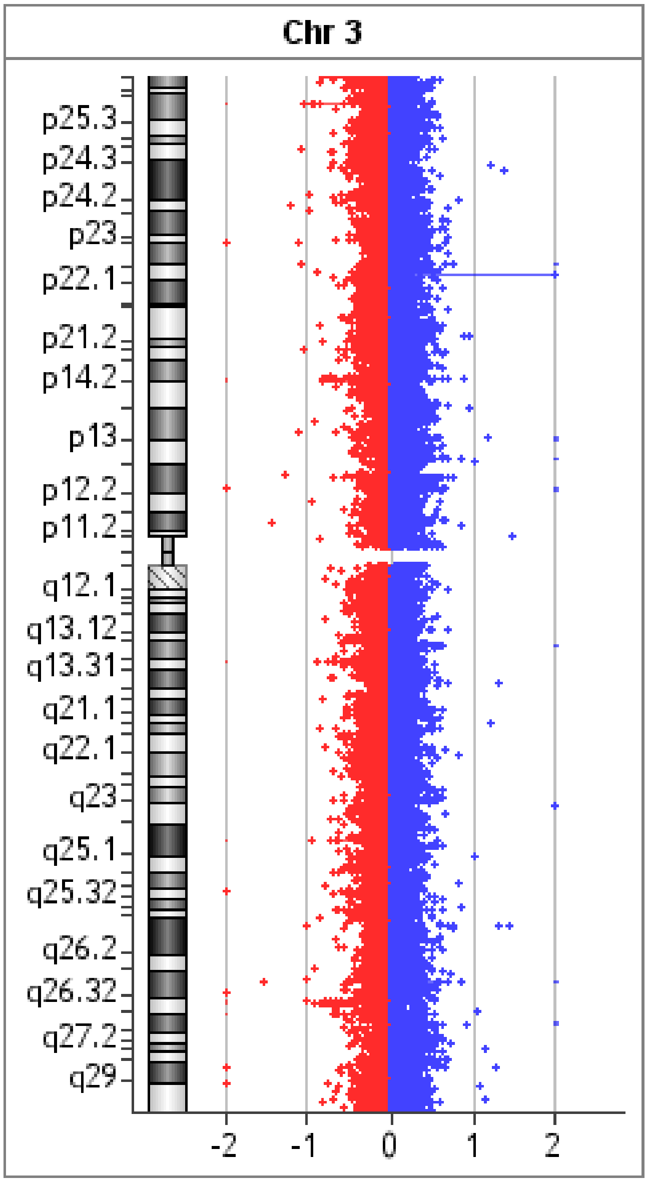

This is an intermediate report and not a final signed off report

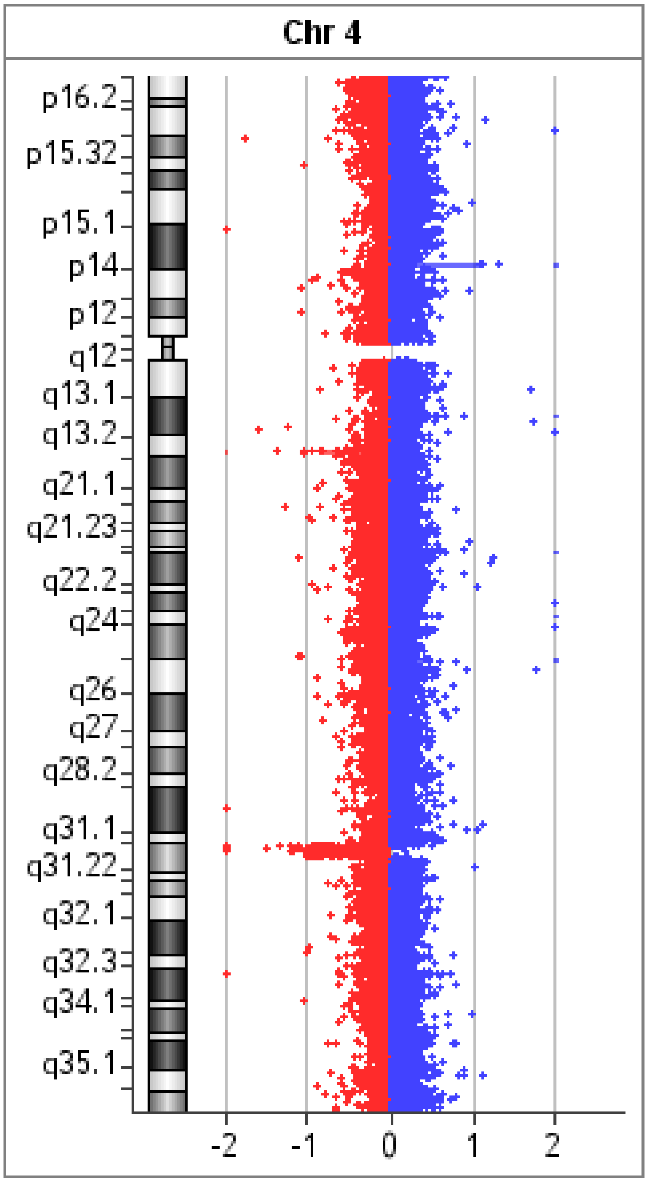

This is an intermediate report and not a final signed off report

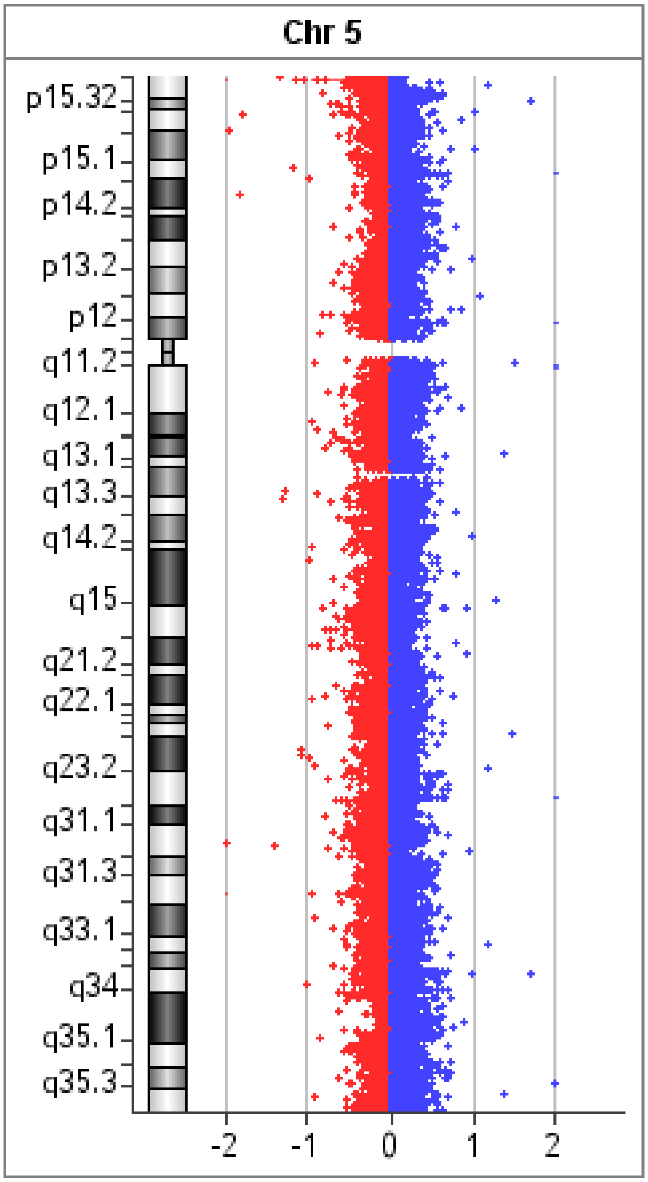

This is an intermediate report and not a final signed off report

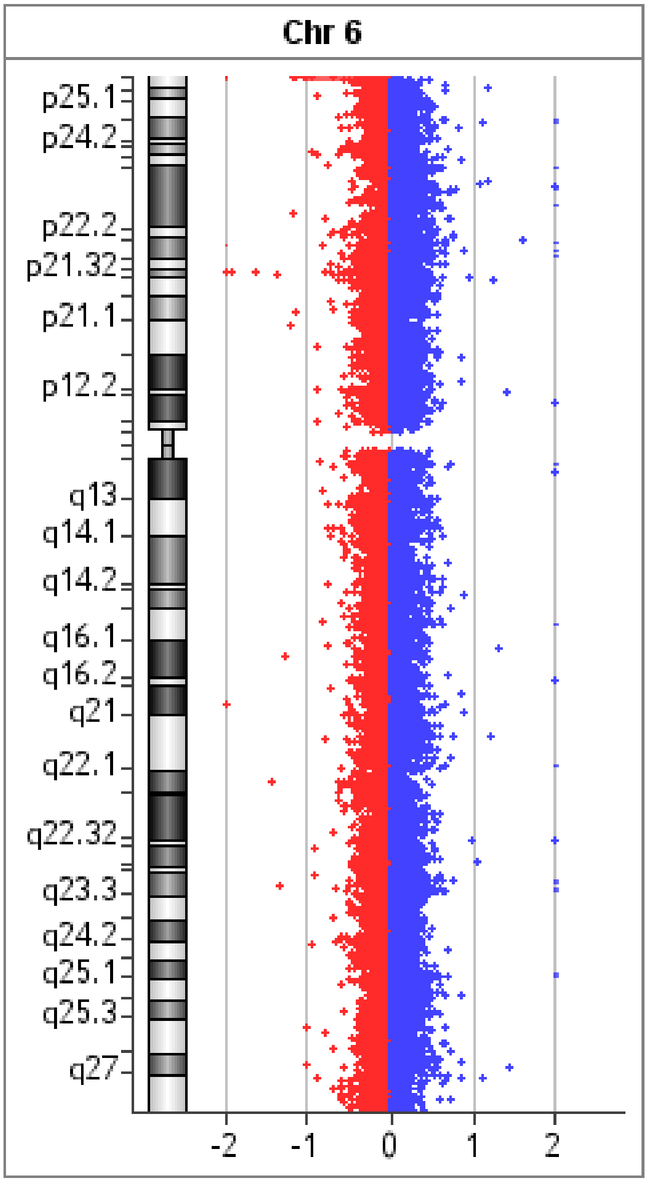

This is an intermediate report and not a final signed off report

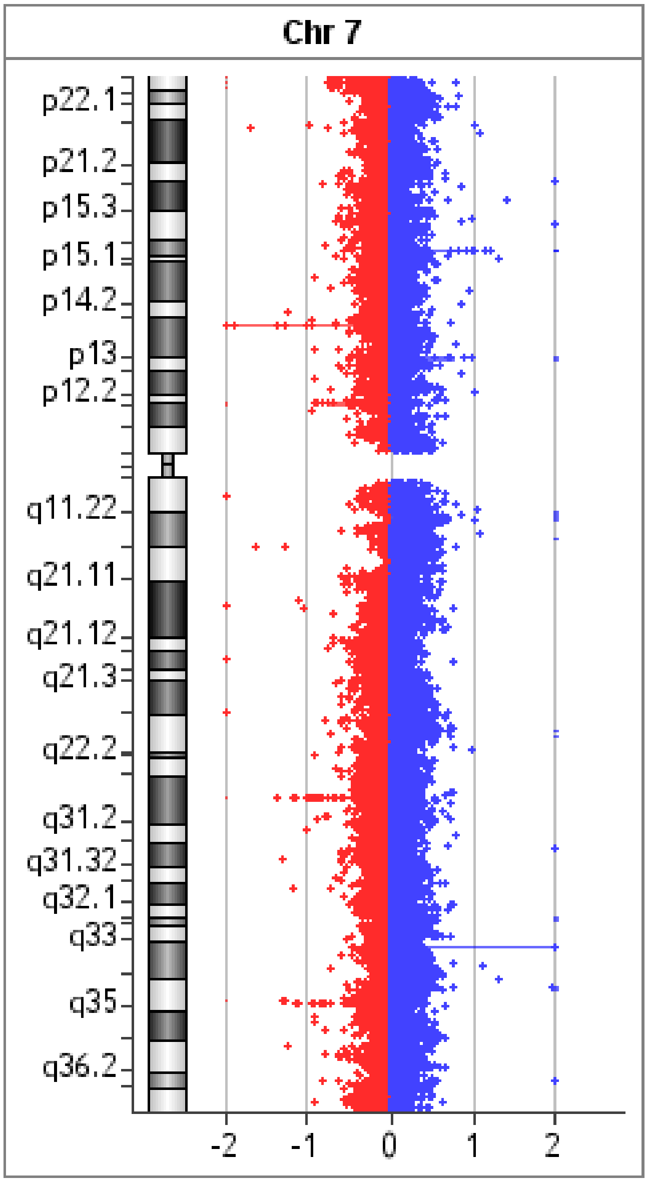

This is an intermediate report and not a final signed off report

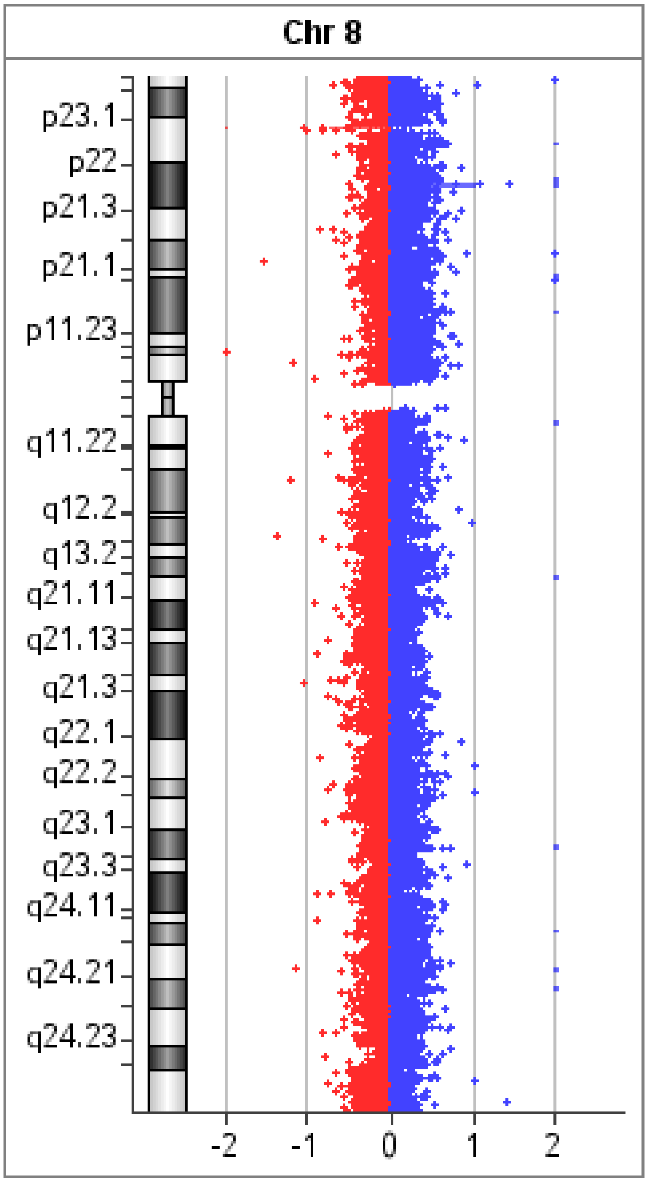

This is an intermediate report and not a final signed off report

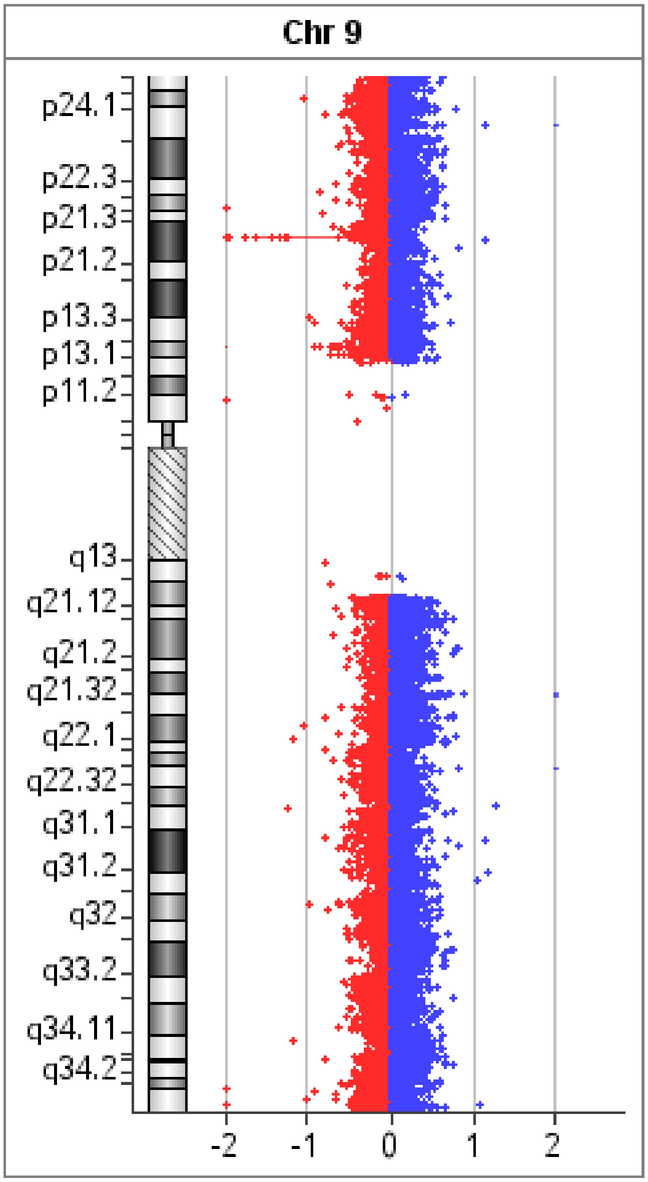

This is an intermediate report and not a final signed off report

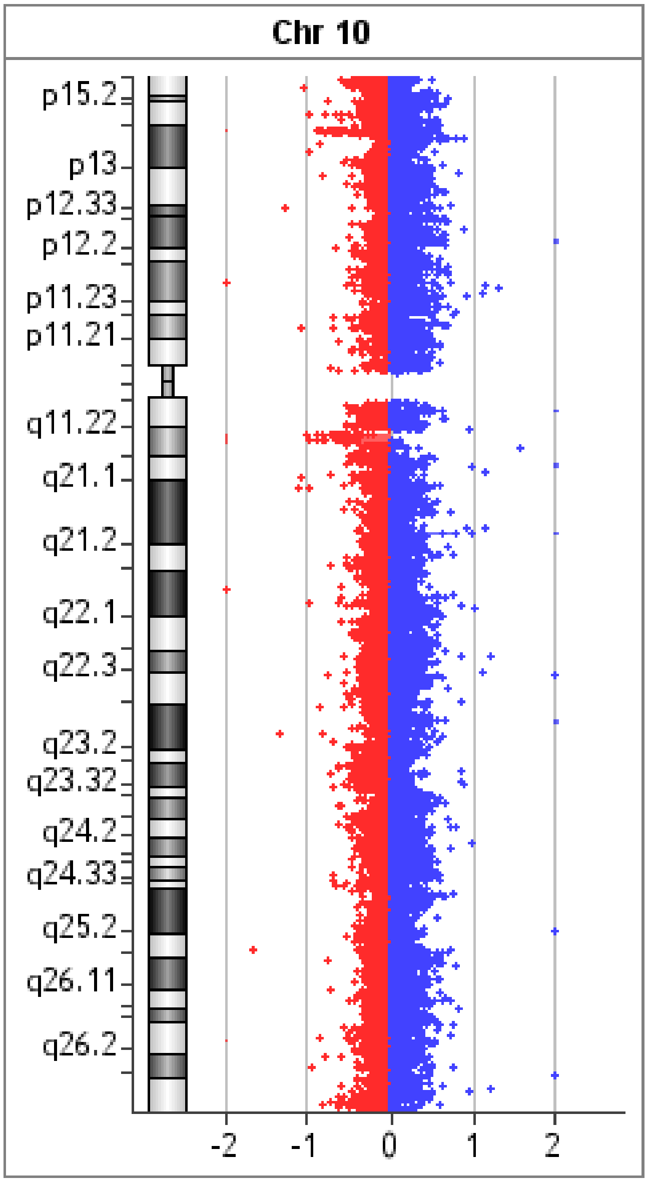

This is an intermediate report and not a final signed off report

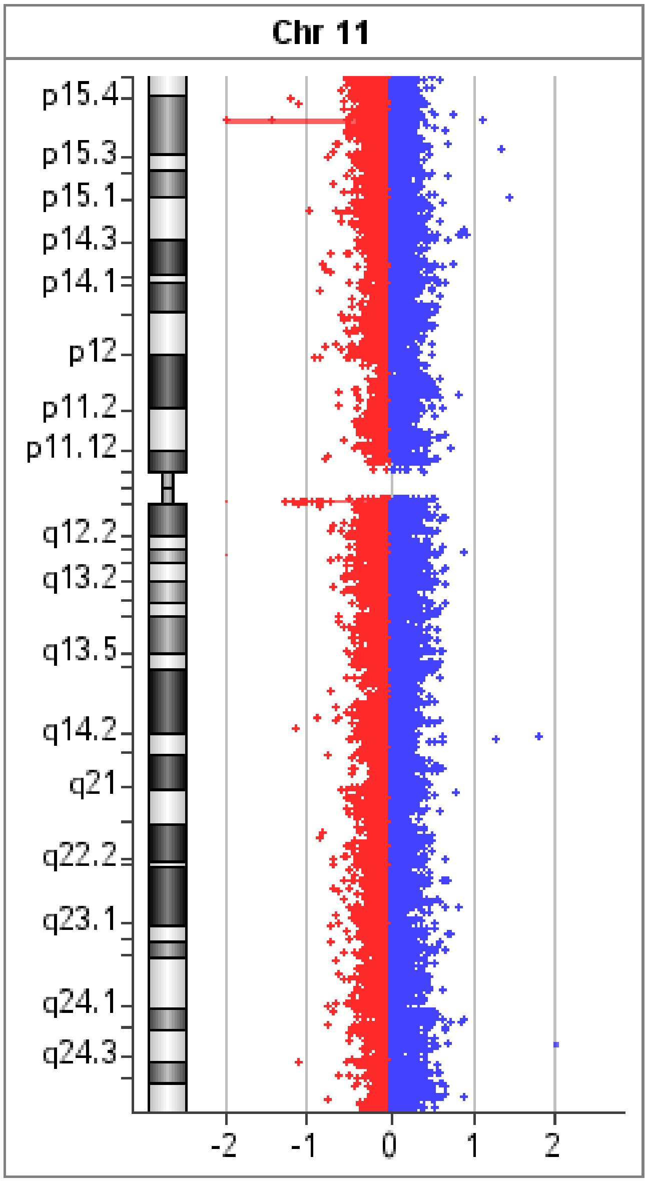

This is an intermediate report and not a final signed off report

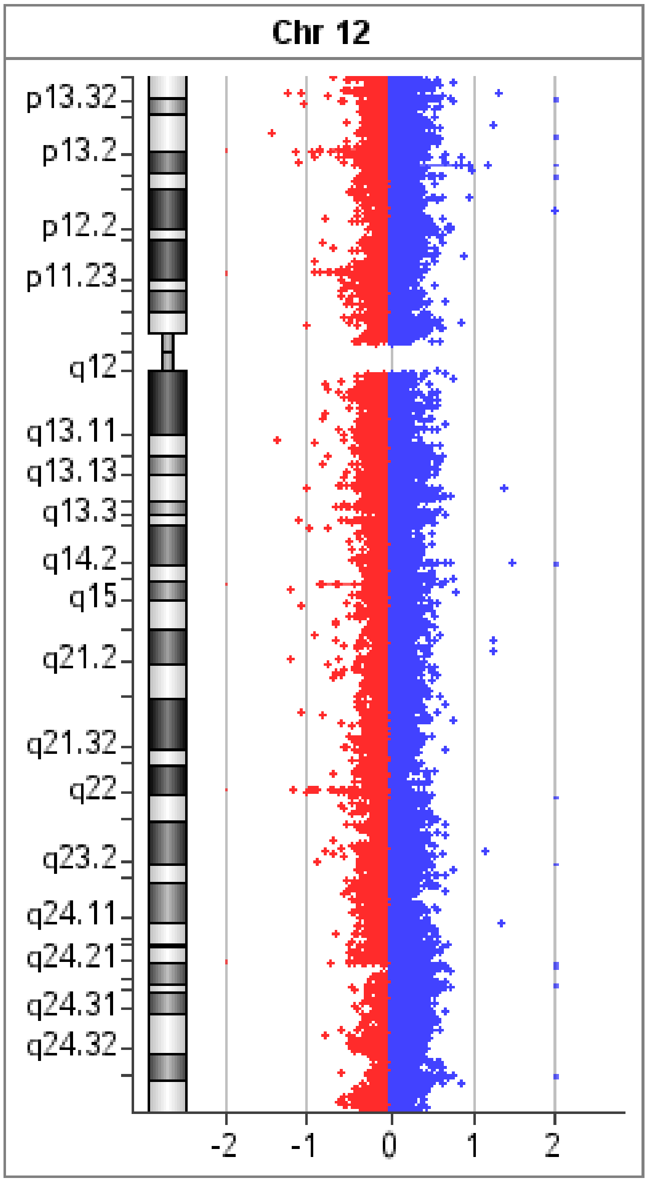

This is an intermediate report and not a final signed off report

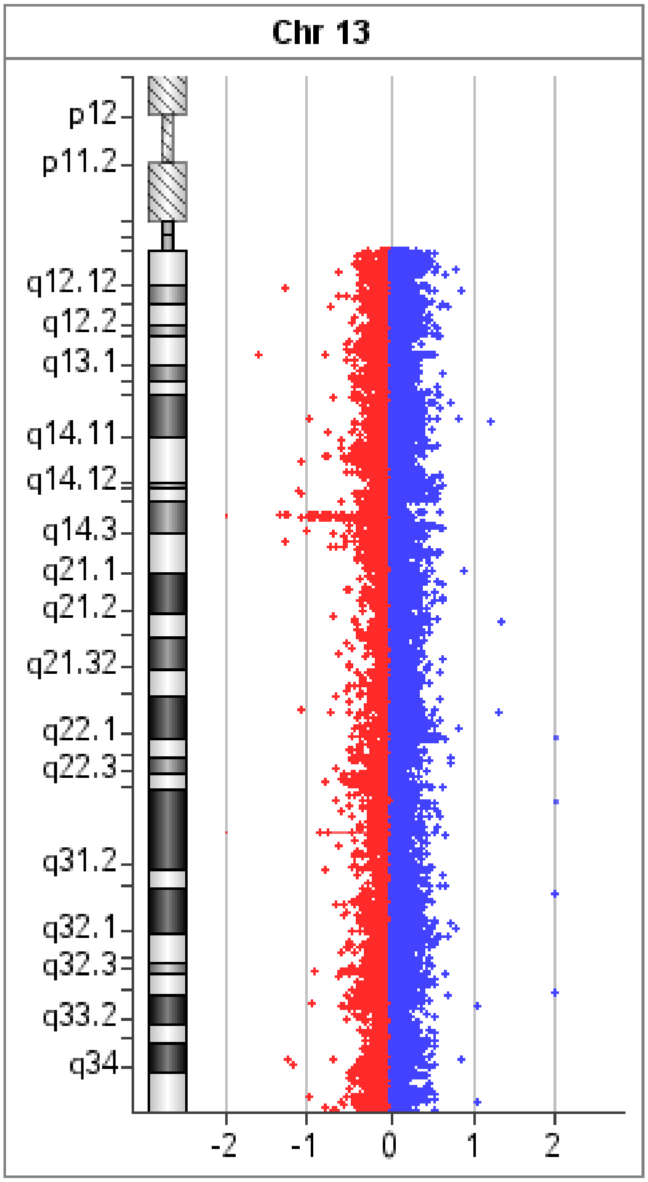

This is an intermediate report and not a final signed off report

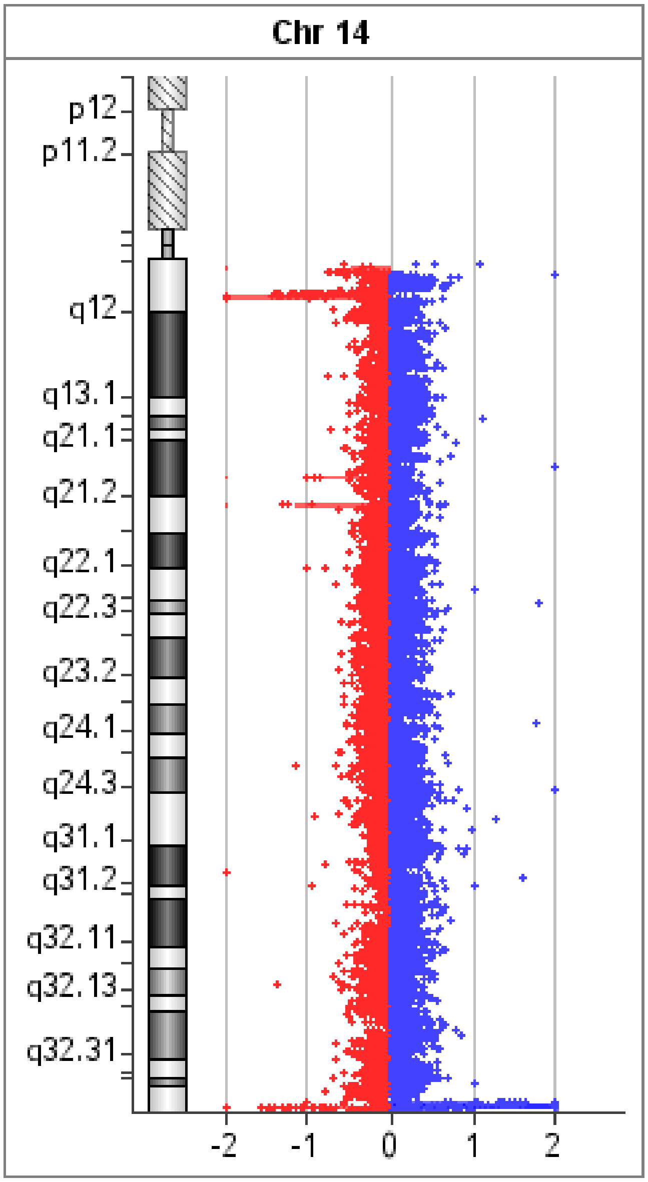

This is an intermediate report and not a final signed off report

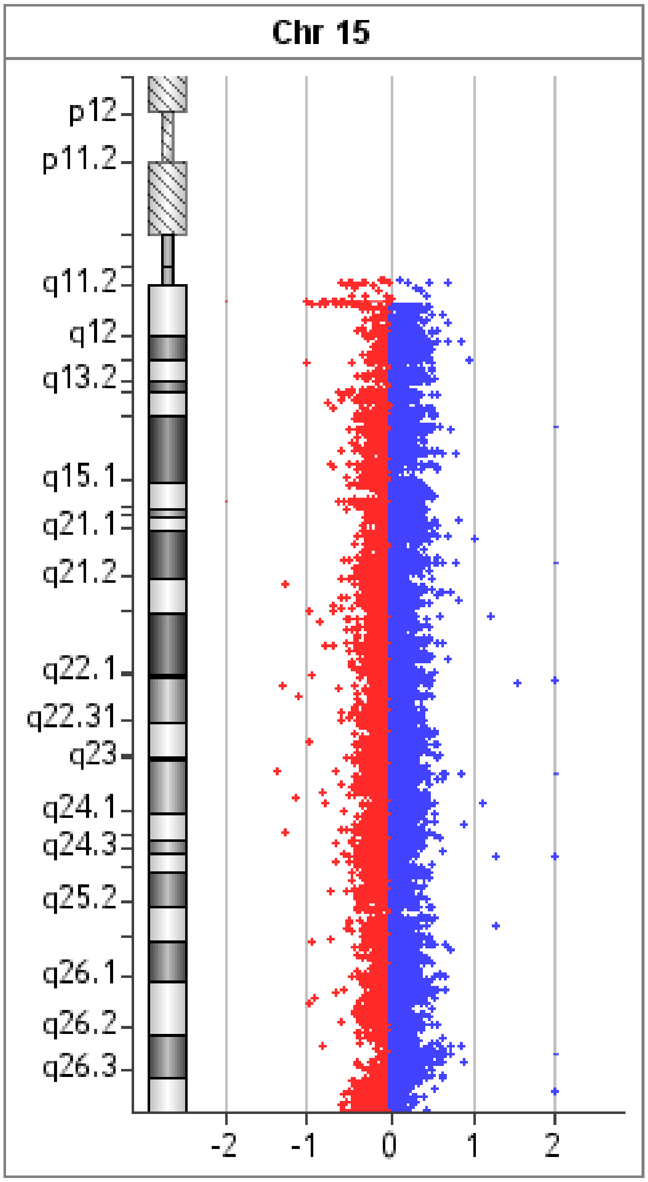

This is an intermediate report and not a final signed off report

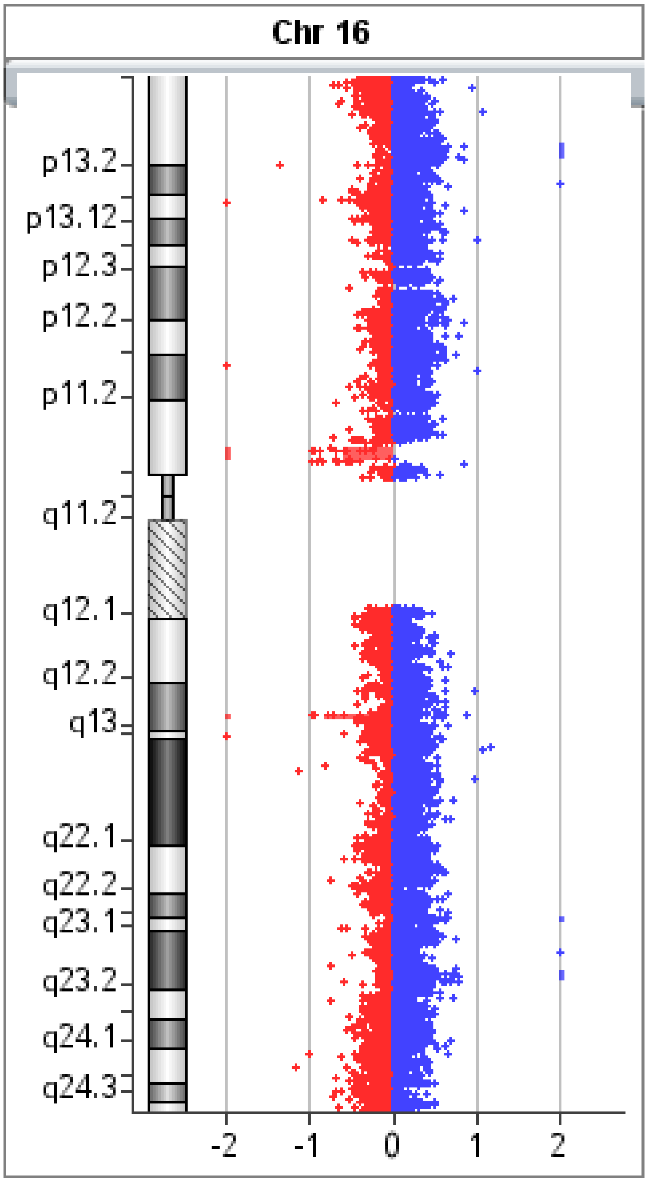

This is an intermediate report and not a final signed off report

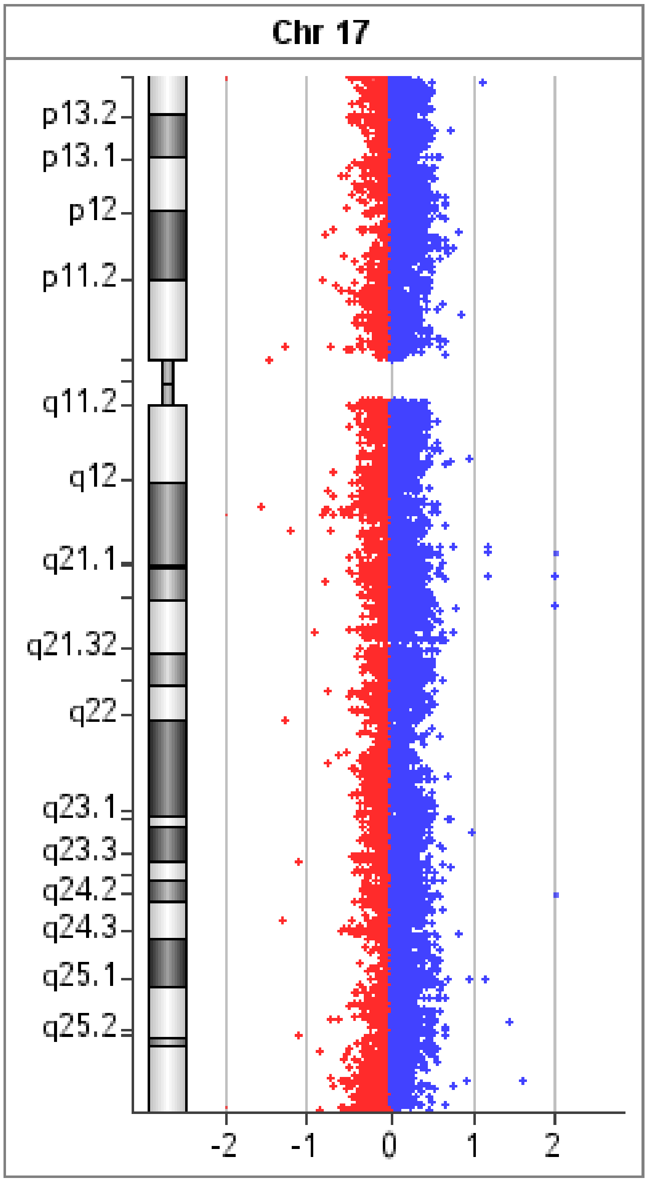

This is an intermediate report and not a final signed off report

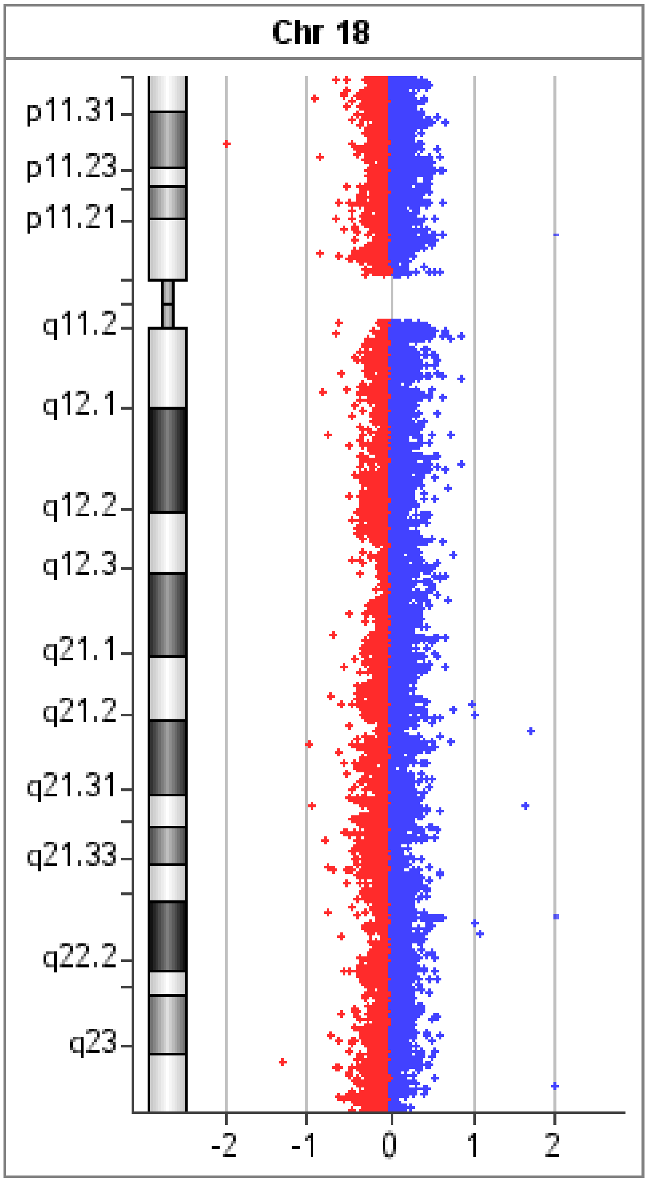

This is an intermediate report and not a final signed off report

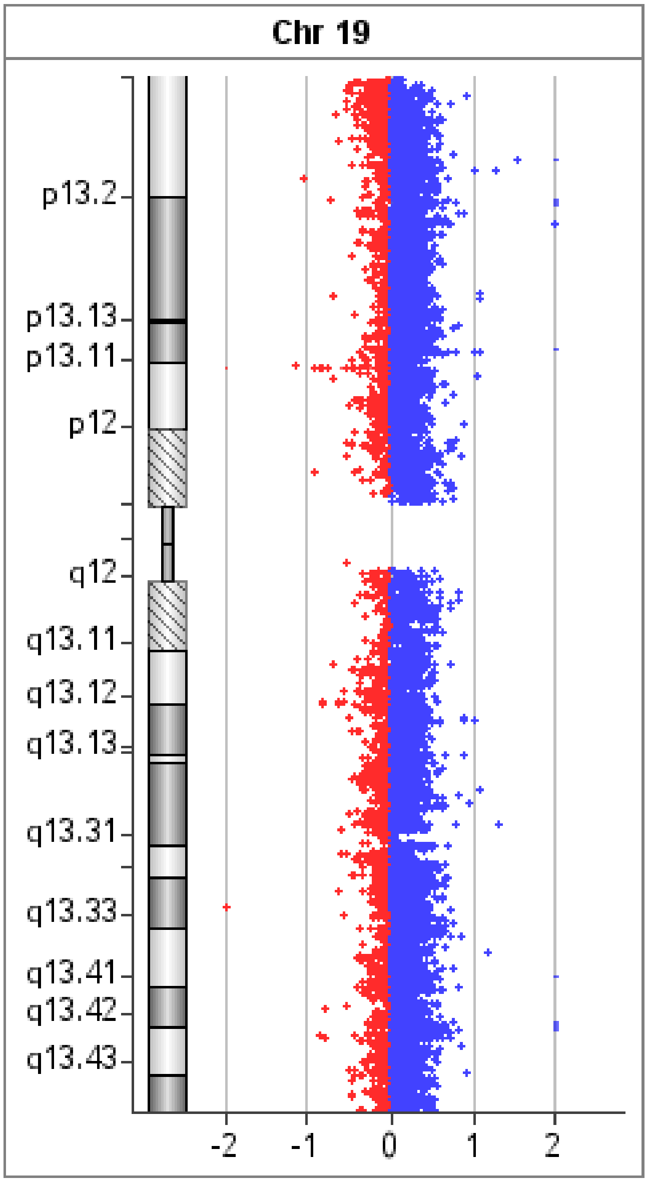

This is an intermediate report and not a final signed off report

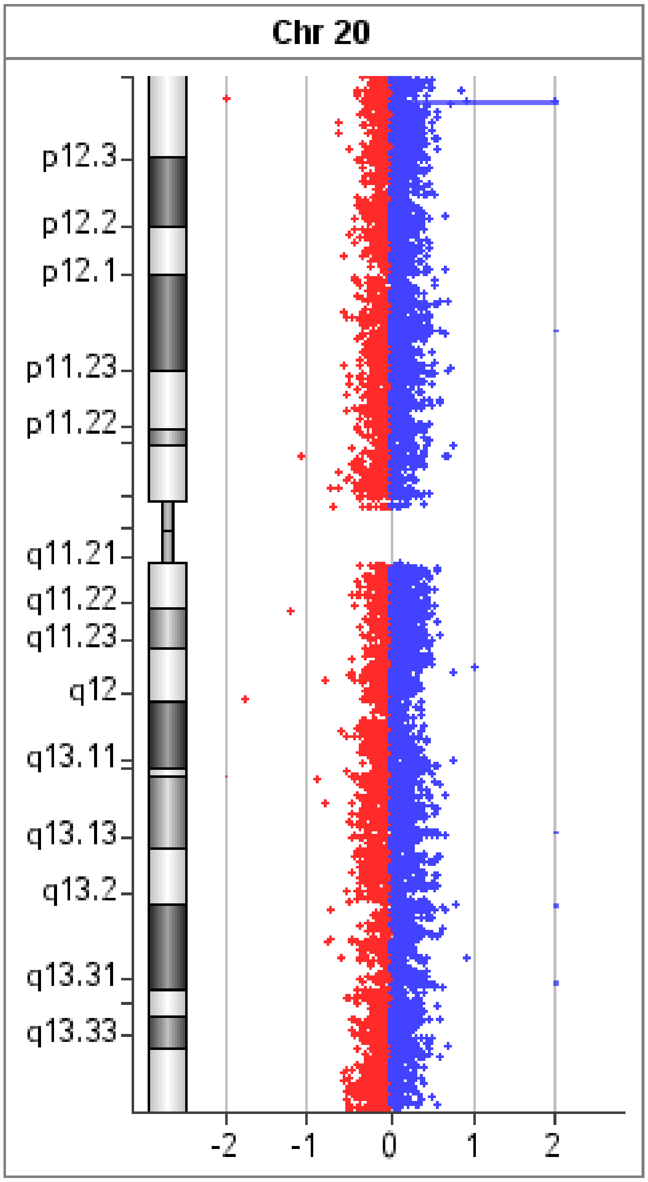

This is an intermediate report and not a final signed off report

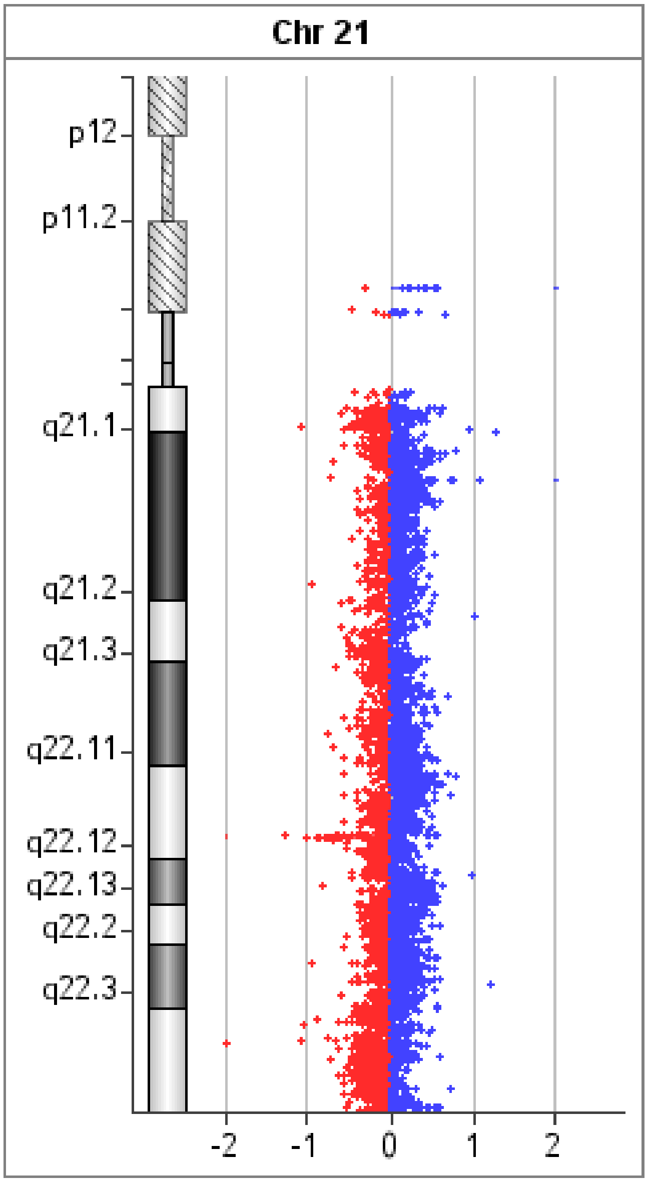

This is an intermediate report and not a final signed off report

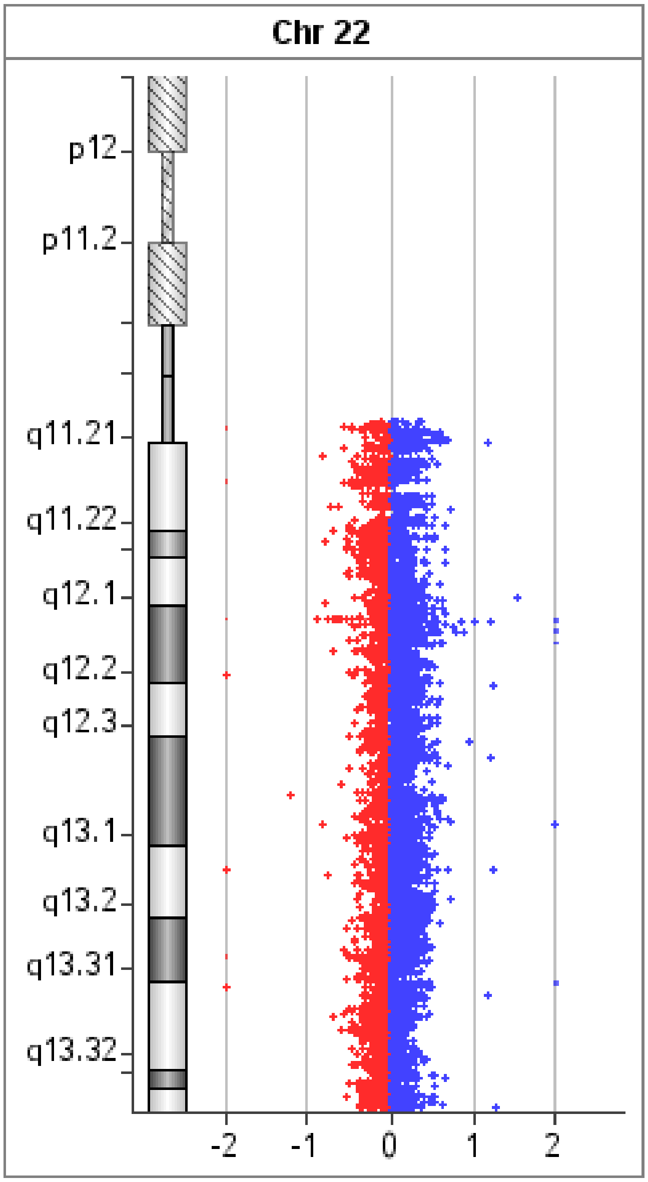

This is an intermediate report and not a final signed off report

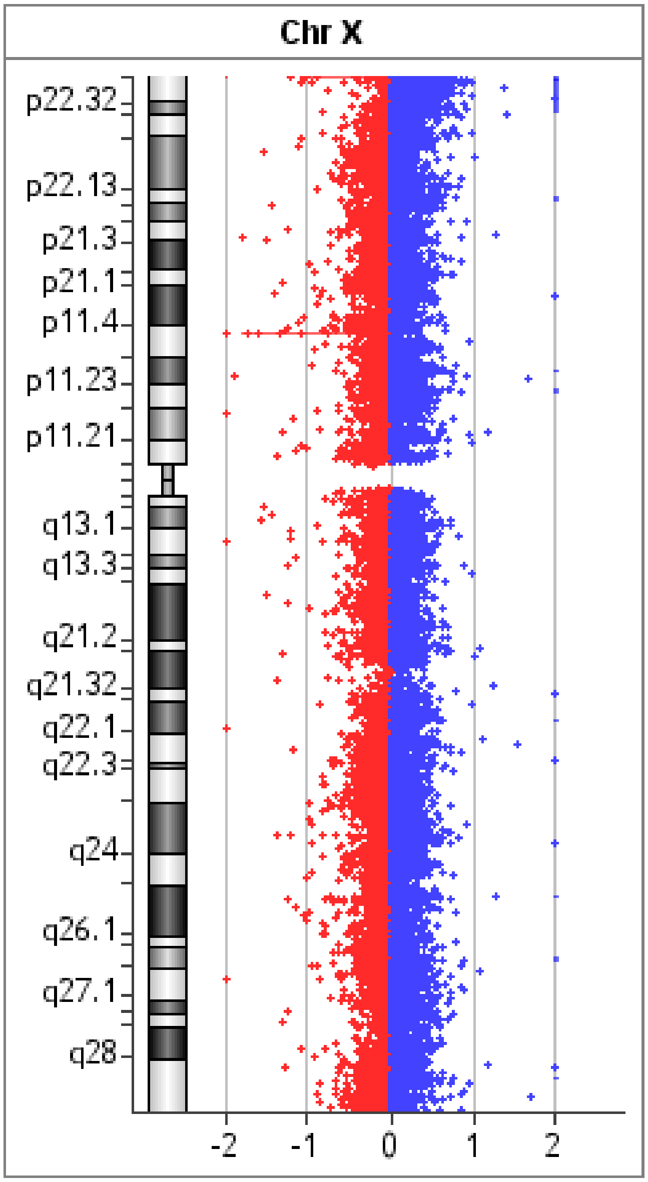

This is an intermediate report and not a final signed off report

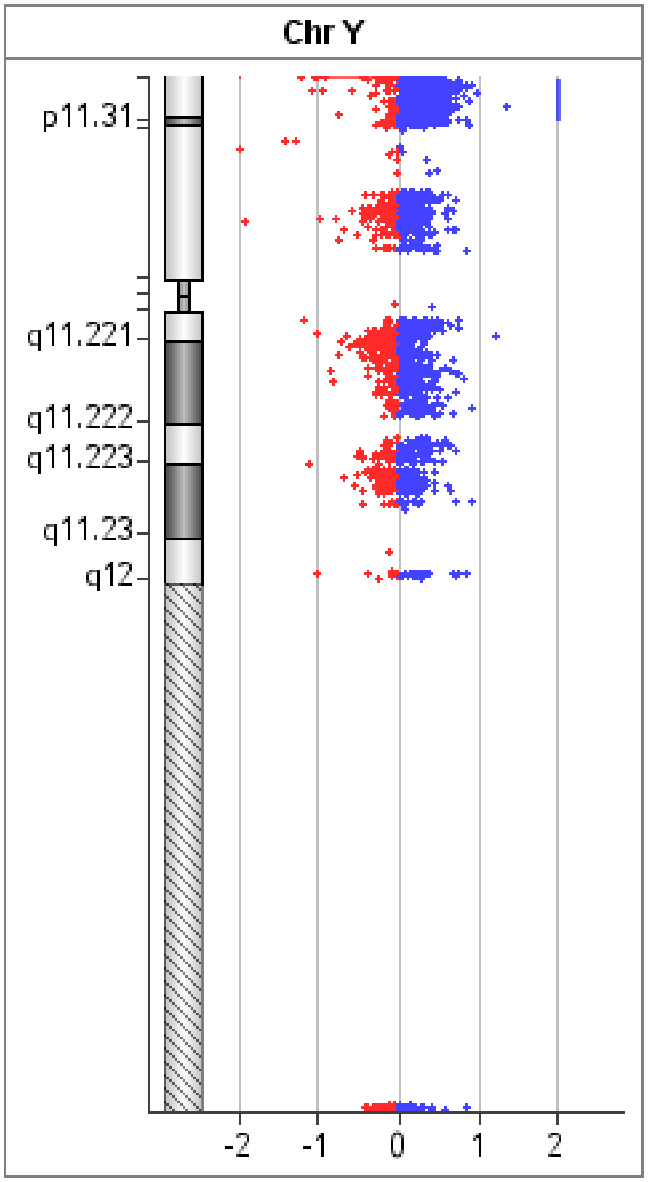

This is an intermediate report and not a final signed off report

Amp/Del Intervals Table

Chr	Start-Stop(bp)	Size(kb)	Cytoband	#Probes	Amp/Gain/ Loss/Del	Annotations	Classifications
chr2	54564146-54786608	222	p16.2	42	-0.699826	C2orf73, SPTBN1, RPL23AP32	Unknown CNV
chr3	57998731-58114058	115	p14.3	22	-0.545014	FLNB, FLNB, p14.3...	Pathogenic CNV
chr4	142143472-144241297	2,098	q31.21	281	-0.630231	ZNF330, IL15, INPP4B...	Pathogenic CNV
chr7	38304417-38352444	48	p14.1	11	-2.320761	TARP, TARP, p14.1...	
chr9	21973728-22008226	34	p21.3	8	-1.553871	CDKN2A, CDKN2B-AS1, CDKN2B	Pathogenic CNV
chr9	36936679-37025239	89	p13.2	19	-0.627054	PAX5, PAX5, p13.2...	Pathogenic CNV
chr10	7166693-7452242	286	p14	56	-0.534684	SFMBT2, SFMBT2, p14...	Pathogenic CNV
chr13	48806530-48874253	68	q14.2	17	-0.869130	ITM2B, ITM2B, q14.2...	Pathogenic CNV
chr13	48998774-49118064	119	q14.2	28	-0.742045	RB1, LPAR6, RCBTB2	Pathogenic CNV
chr22	26872961-26931831	59	q12.1	19	-0.555123	HPS4, SRRD, TFIP11...	Pathogenic CNV

Amp=Amplification
Del=Deletion

Total Amp/Del Intervals: 10

This is an intermediate report and not a final signed off report

ISCN Nomenclature

arr 2p16.2(54,564,146-54,786,608)x1,3p14.3(57,998,731-58,114,058)x1,4q31.21(142,143,472-144,241,297)x1,7p14.1(38,304,417-38,352,444)x1
,9p21.3(21,973,728-22,008,226)x1,9p13.2(36,936,679-37,025,239)x1,10p14(7,166,693-7,452,242)x1,13q14.2(48,806,530-48,874,253)x1
,13q14.2(48,998,774-49,118,064)x1,22q12.1(26,872,961-26,931,831)x1

This is an intermediate report and not a final signed off report

Analysis Settings

Design	:	021850_20111015
Genome	:	hg19
Threshold	:	6.0
GC Correction	:	ON
Centralization (legacy)	:	OFF
SNP Copy Number	:	OFF
Combine Replicates (Intra Array)	:	ON
Metric Set Filter	:	NONE
Feature Level Filter	:	glsSaturated = true OR rIsSaturated = true OR glsFeatNonUnifOL = true OR rIsFeatNonUnifOL = true OR LogRatio = 0; Include matching values=false
LOH Filter	:	NONE
Show Flat Intervals	:	false

Sample Name	:	252185022956_1_2
Aberration Algorithm	:	ADM-2
Fuzzy Zero	:	OFF
Window Size	:	2Kb
Diploid Peak Centralization	:	ON
LOH	:	OFF
Array Level Filter	:	NONE
Aberration Filter	:	Minimum Number of Probes for Amplification >= 3 AND Nesting Level <= 100 AND Minimum Avg. Absolute Log Ratio for Amplification >= 0.25 AND Minimum Size (Kb) of Region for Amplification >= 0.0 AND Minimum Size (Kb) of Region for Deletion >= 0.0 AND Minimum Number of Probes for Deletion >= 3 AND Minimum Avg. Absolute Log Ratio for Deletion >= 0.25
Design Level Filter	:	Homology = 0 OR IsPseudoautosomal = 1
Genomic Boundary	:	OFF
Template Name	:	OUHSC CGH report

This is an intermediate report and not a final signed off report

Notes

Sample Notes

No notes available.

Amp/Del Interval Notes

No notes available.

This is an intermediate report and not a final signed off report
