# Supplementary material for: Genomic Copy Number Variants in CML Patients With the Philadelphia Chromosome (Ph+): An Update
Source: Front Genet. 2021 Aug 10;12:697009. doi: 10.3389/fgene.2021.697009 (PMC8383316; doi:10.3389/fgene.2021.697009)
Supplement: Supplementary file 3 [file Data_Sheet_3.PDF]

Sample Information

DerivativeOfLogRatioSD	:	0.120395
Red Sample	:	
Polarity	:	1
Global Display Name	:	14-0045GM-0039D-Wel,C-252185022597_1_1
Array ID	:	252185022597_1_1
Green Sample	:	Agilent Euro Female
Intermediate Report by	:	OUHSC\xwang3

This is an intermediate report and not a final signed off report

Genome View (Amp/Del)

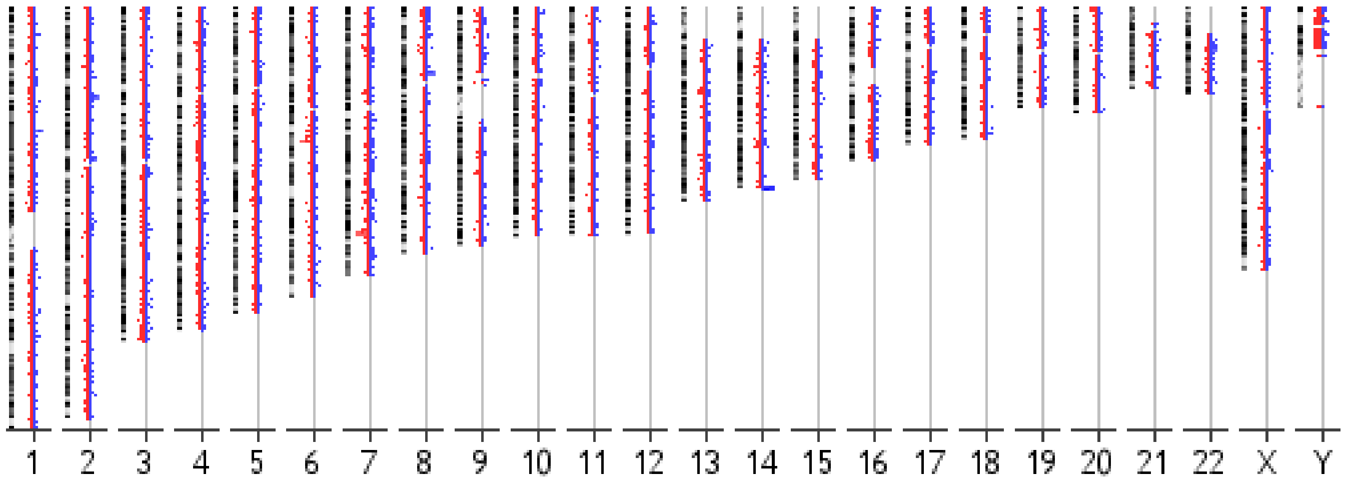

This is an intermediate report and not a final signed off report

Chromosome Views (Amp/Del)

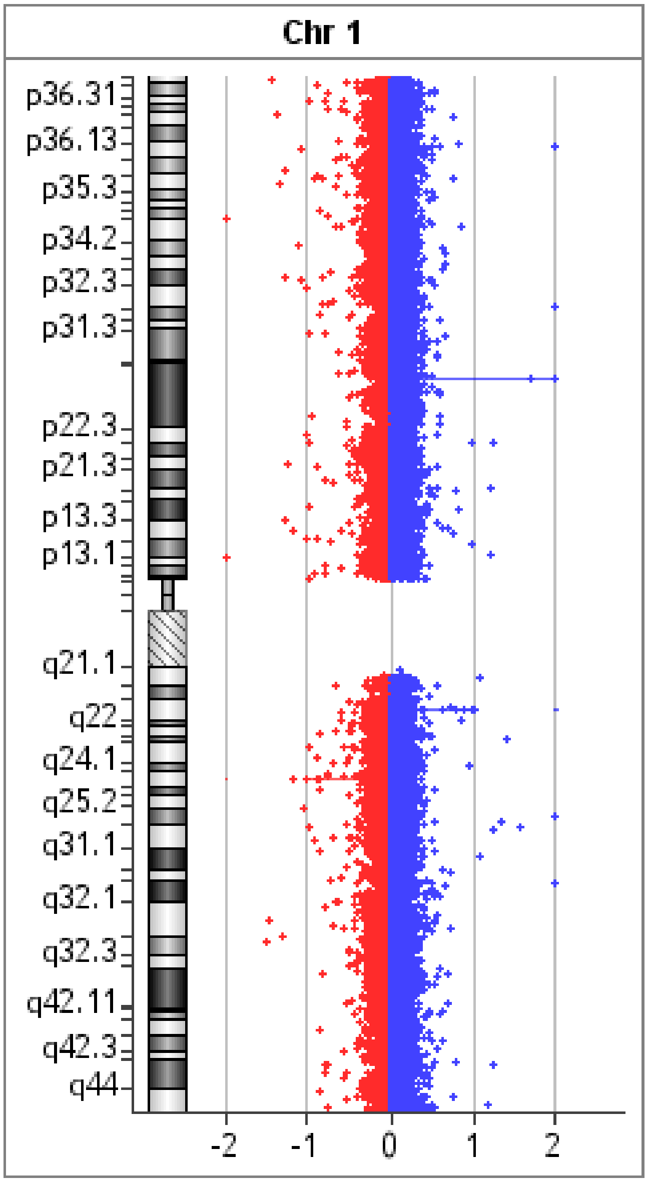

This is an intermediate report and not a final signed off report

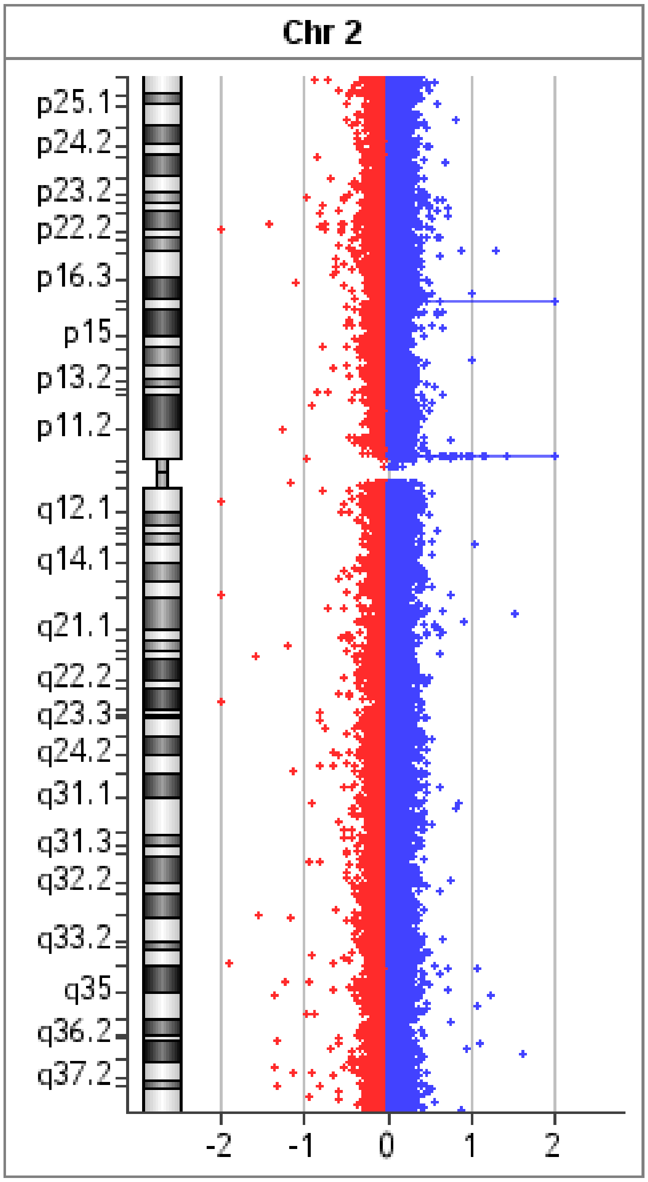

This is an intermediate report and not a final signed off report

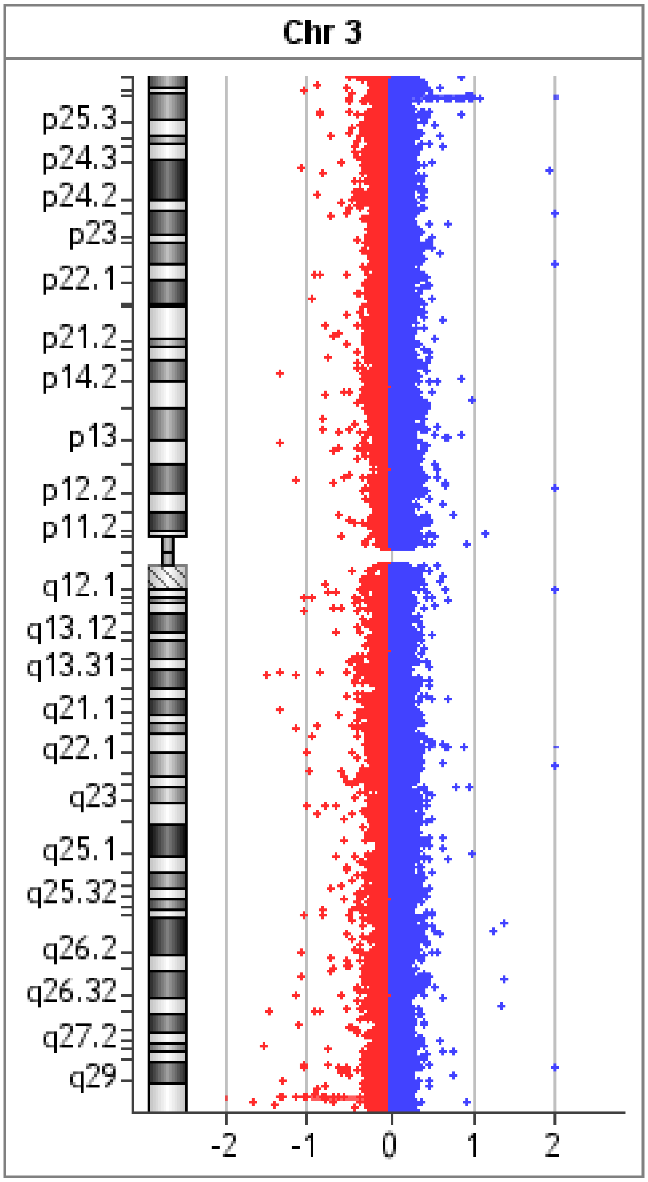

This is an intermediate report and not a final signed off report

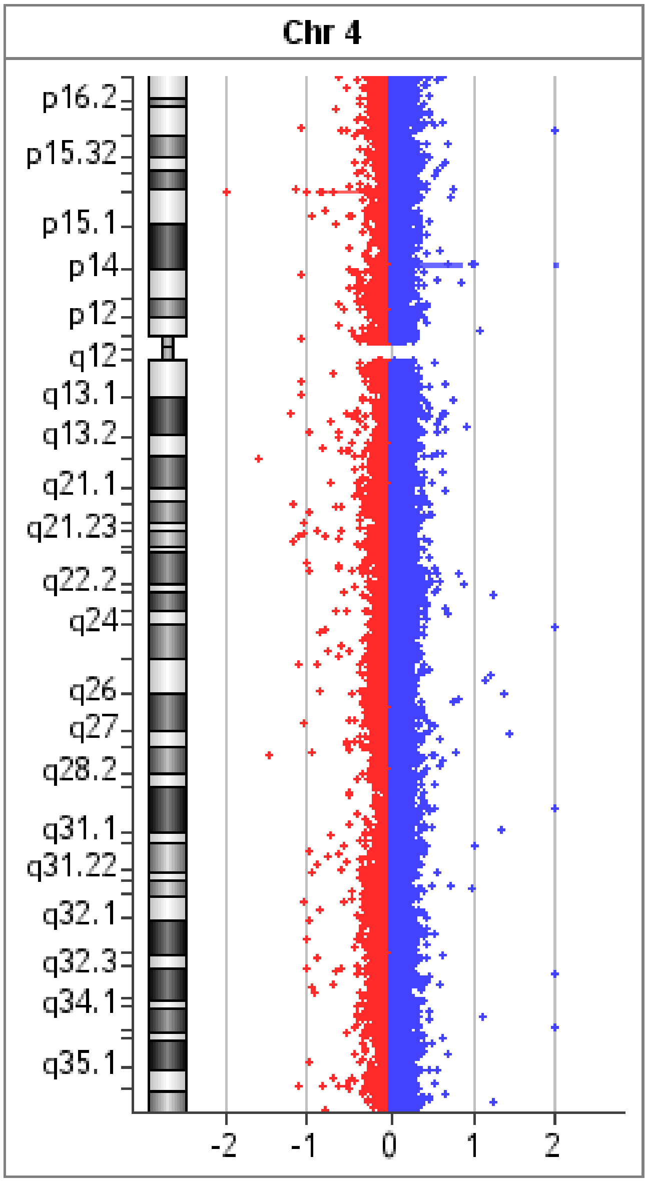

This is an intermediate report and not a final signed off report

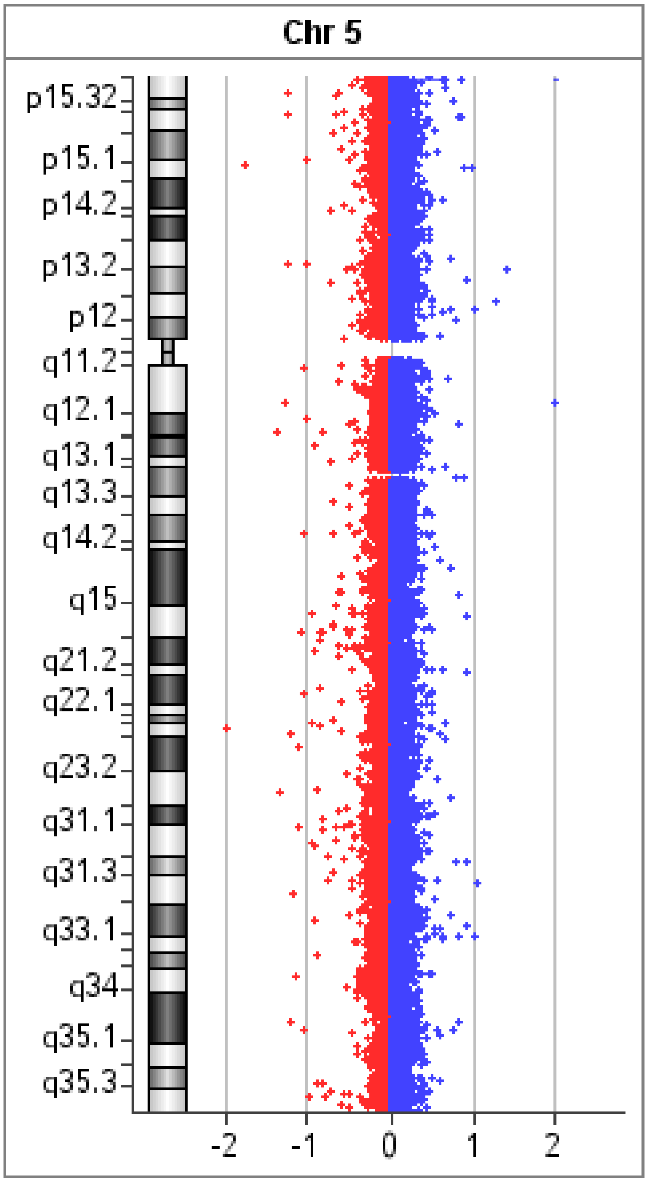

This is an intermediate report and not a final signed off report

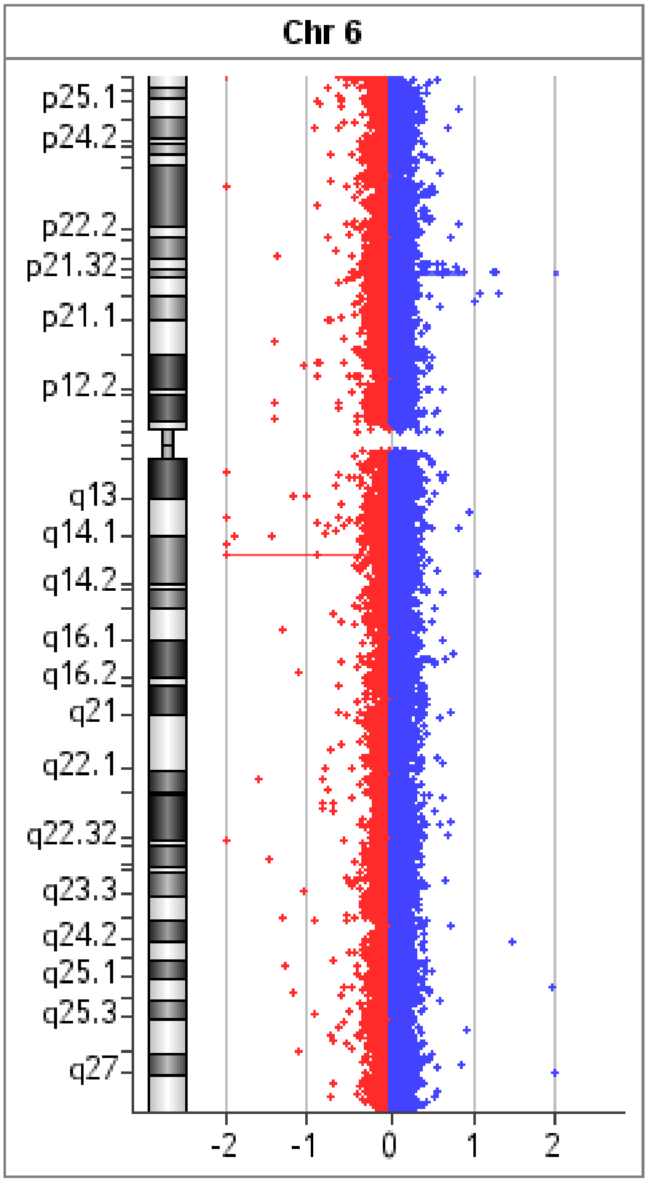

This is an intermediate report and not a final signed off report

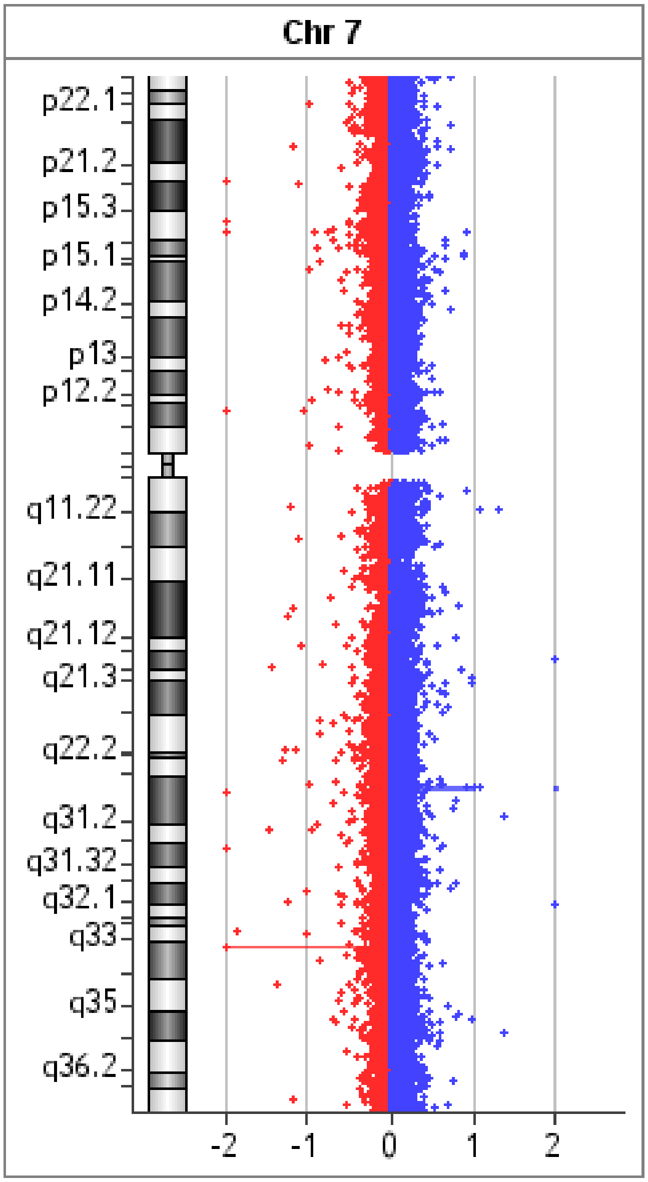

This is an intermediate report and not a final signed off report

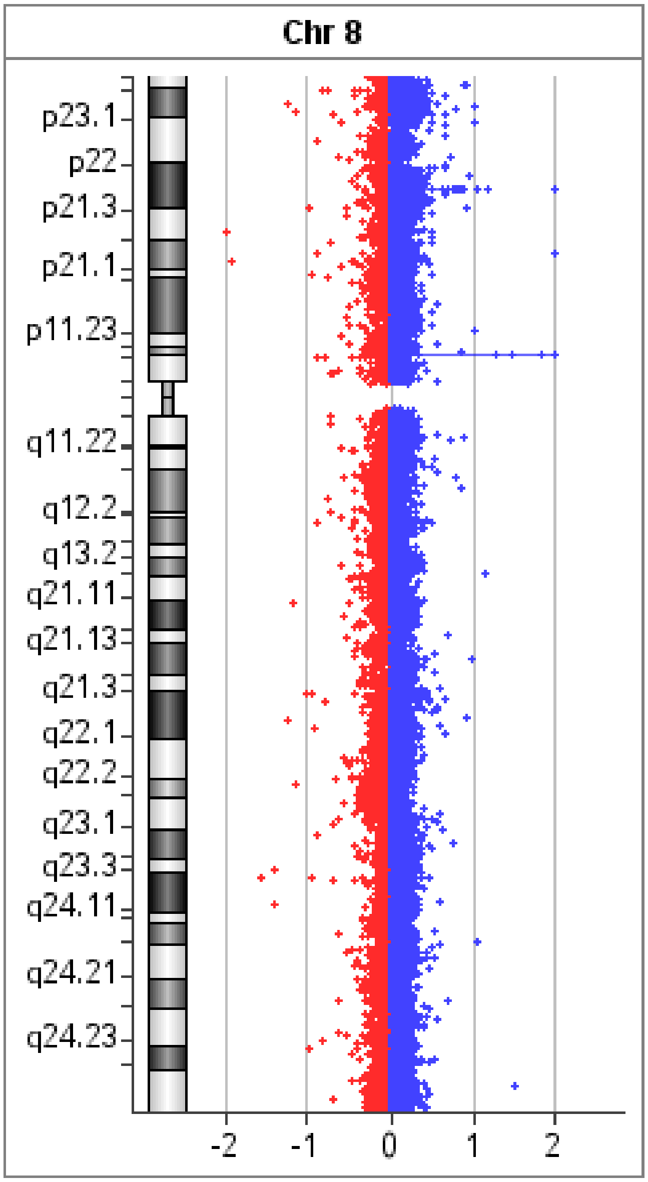

This is an intermediate report and not a final signed off report

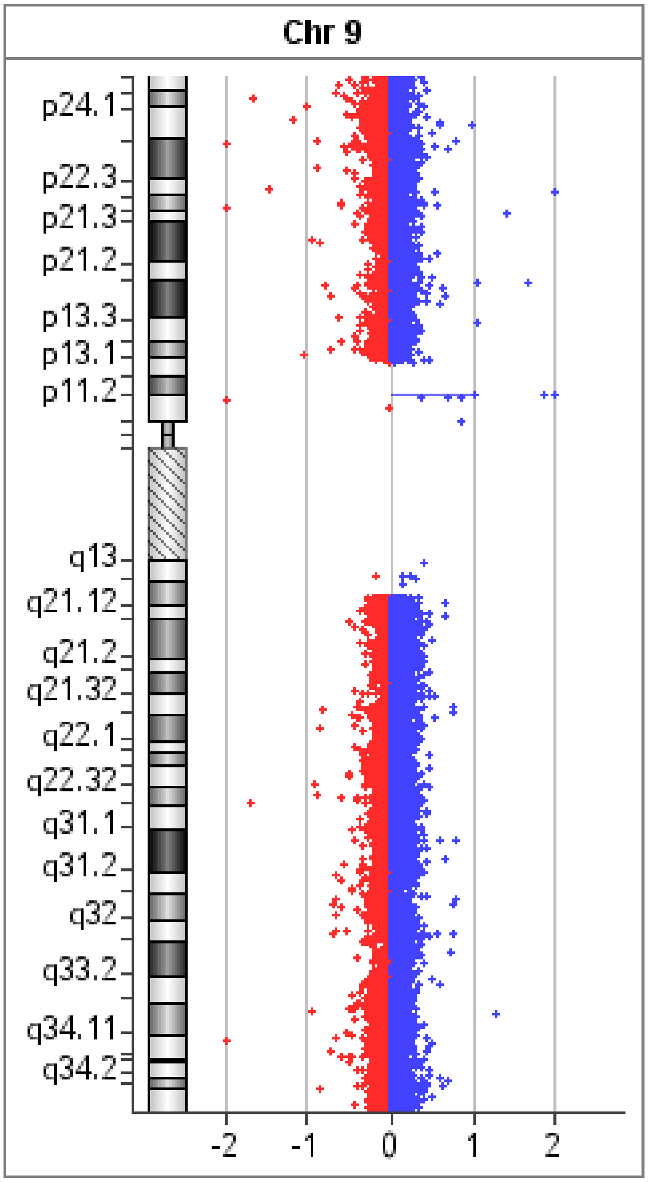

This is an intermediate report and not a final signed off report

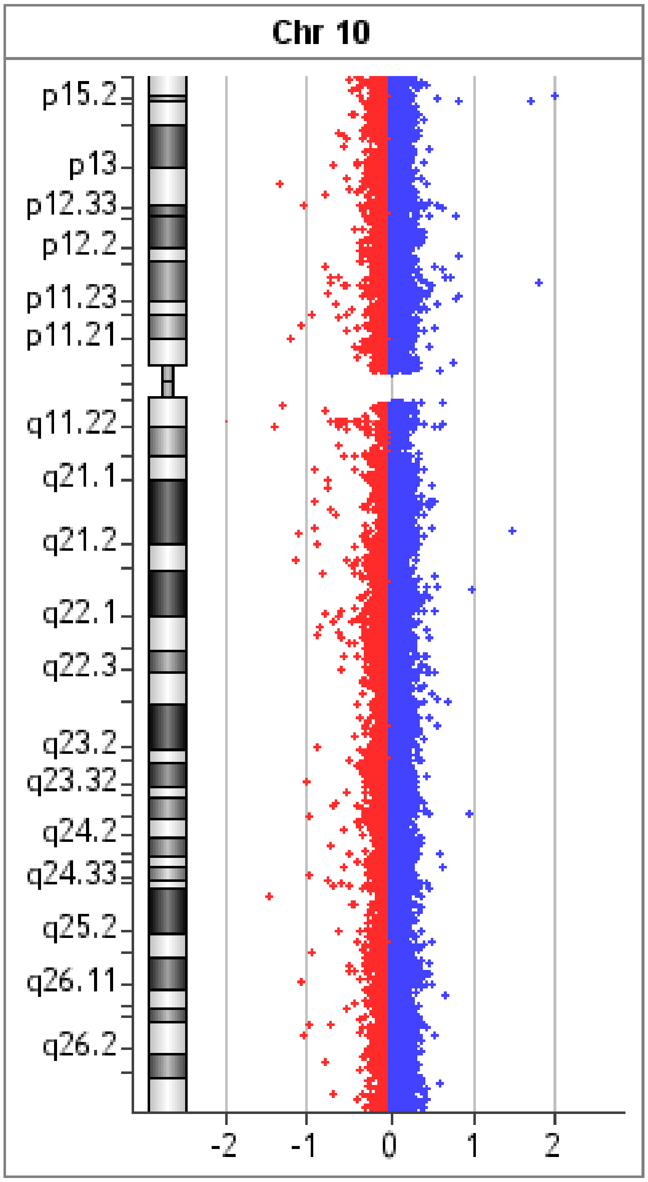

This is an intermediate report and not a final signed off report

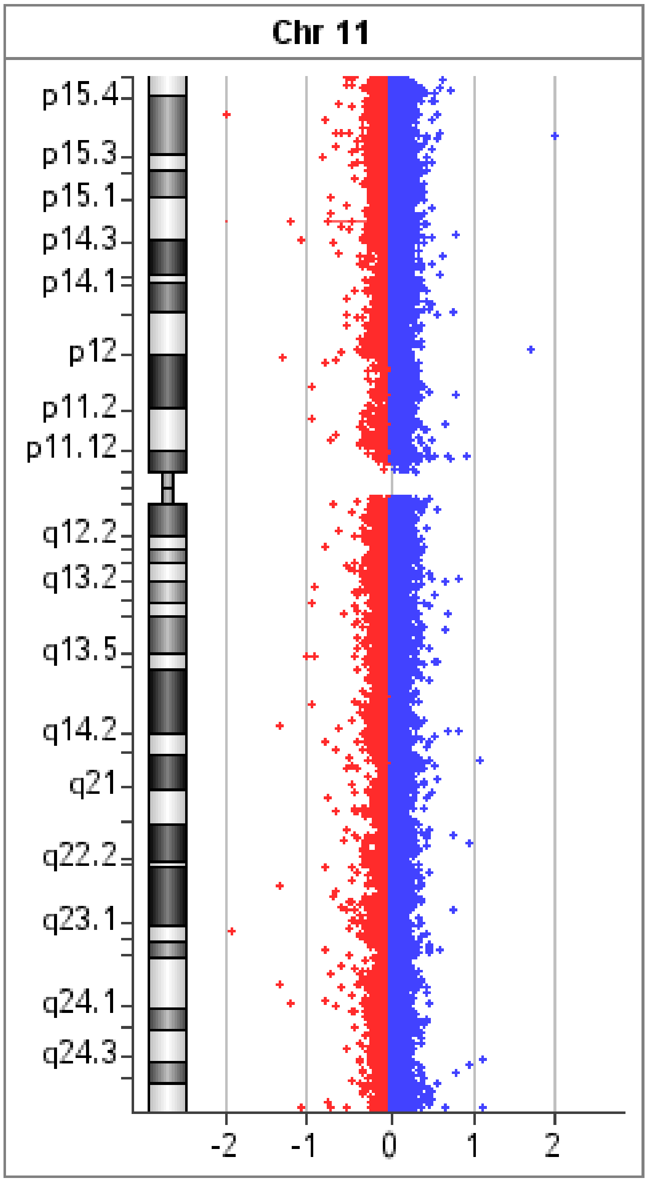

This is an intermediate report and not a final signed off report

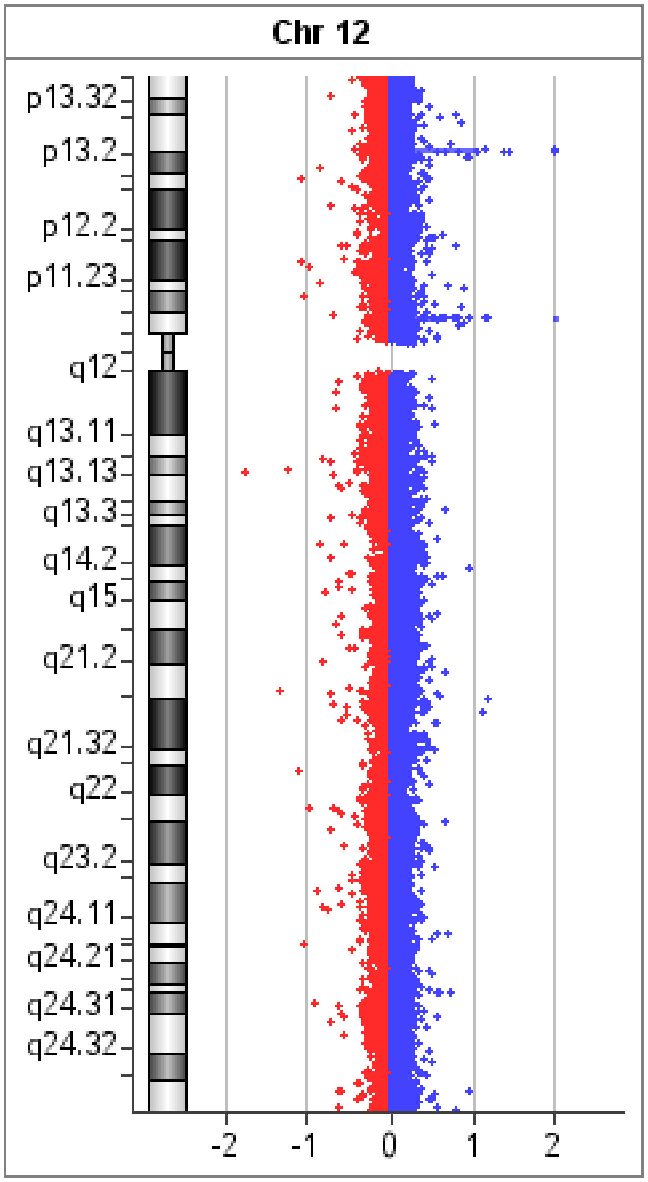

This is an intermediate report and not a final signed off report

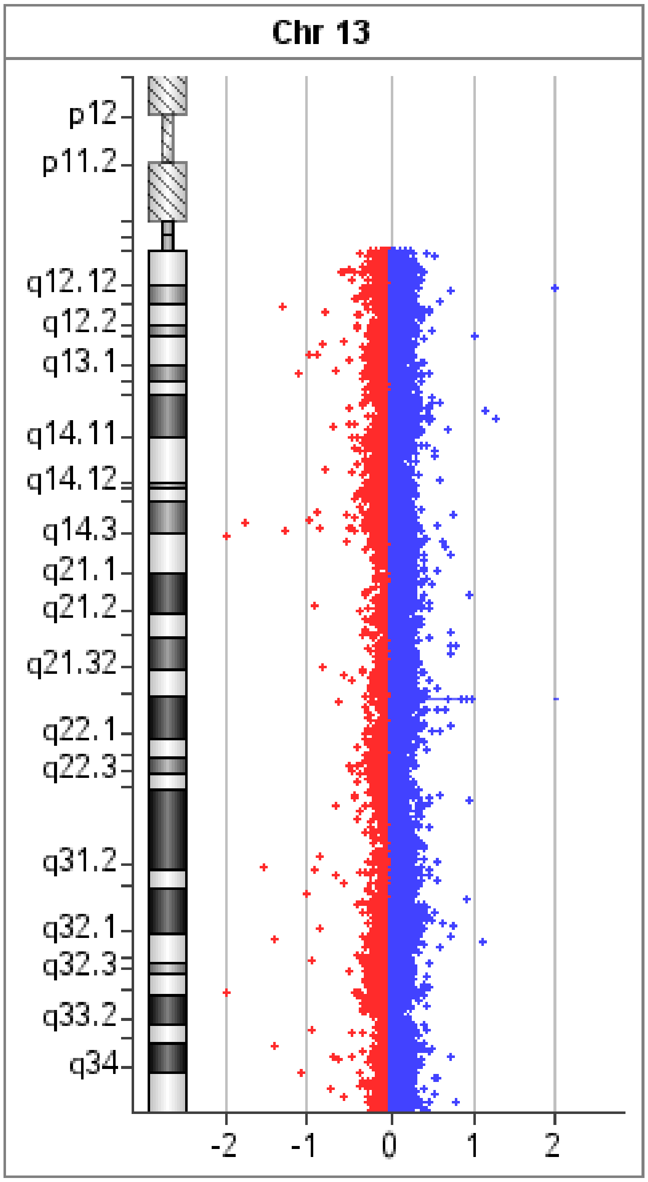

This is an intermediate report and not a final signed off report

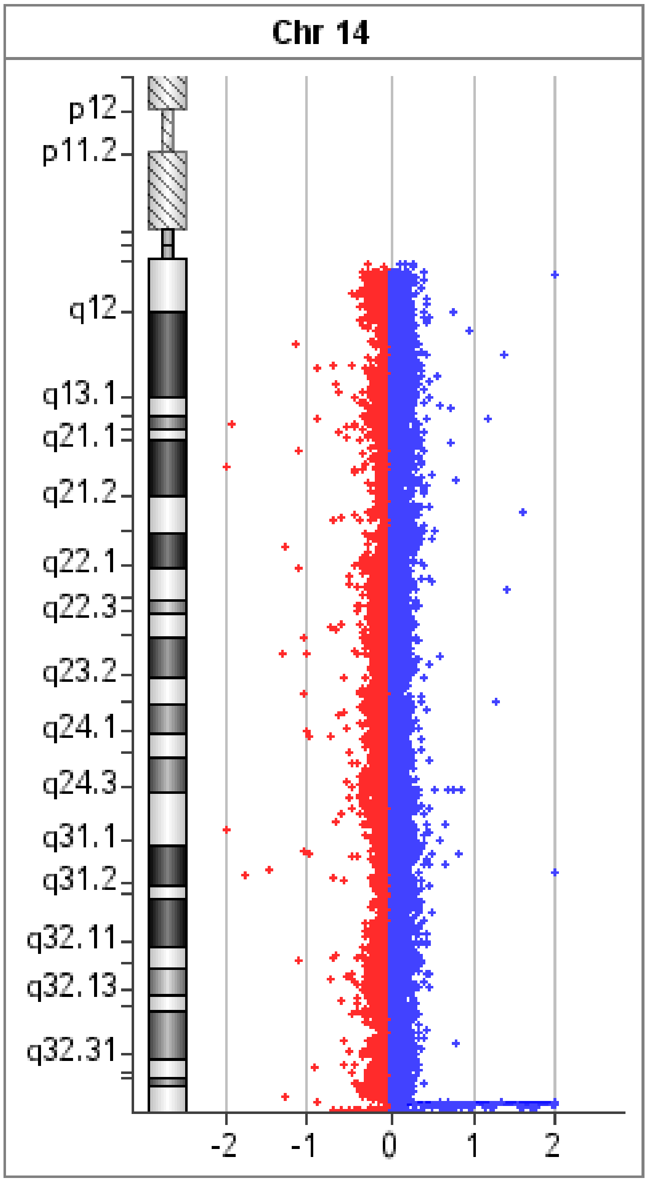

This is an intermediate report and not a final signed off report

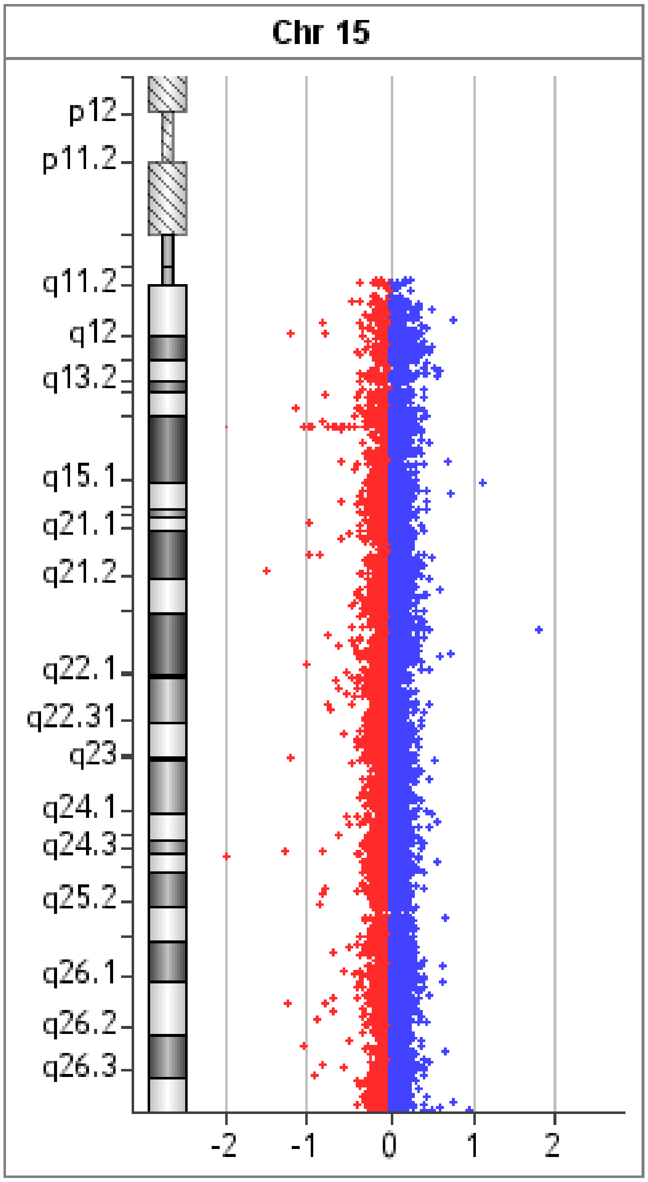

This is an intermediate report and not a final signed off report

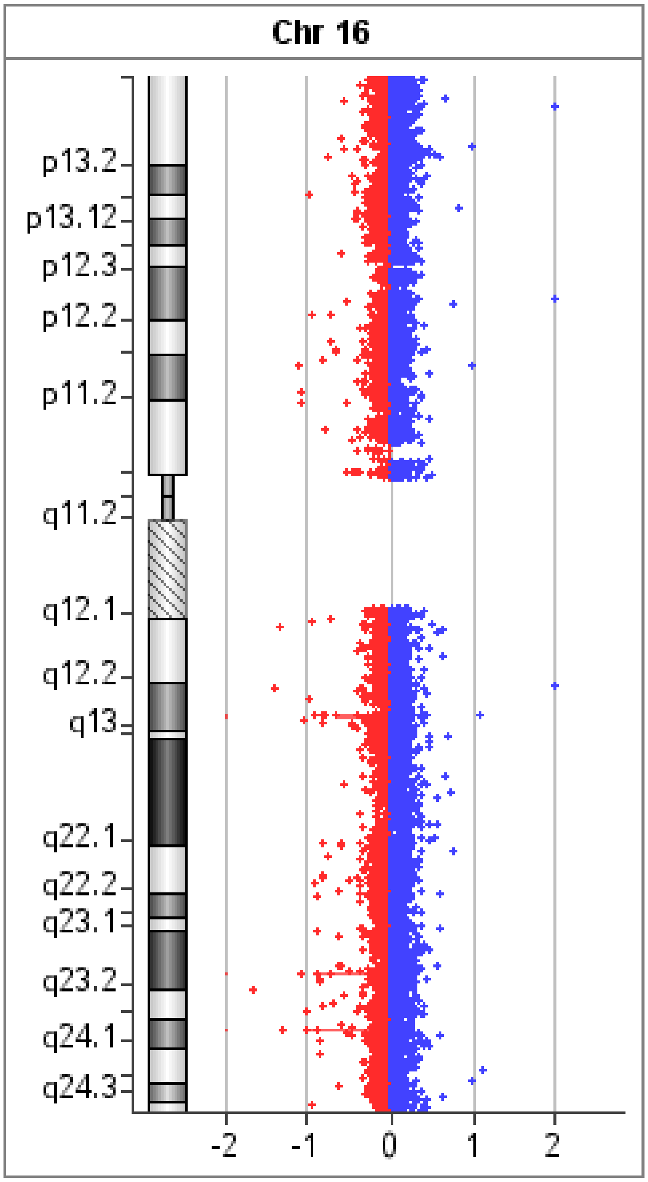

This is an intermediate report and not a final signed off report

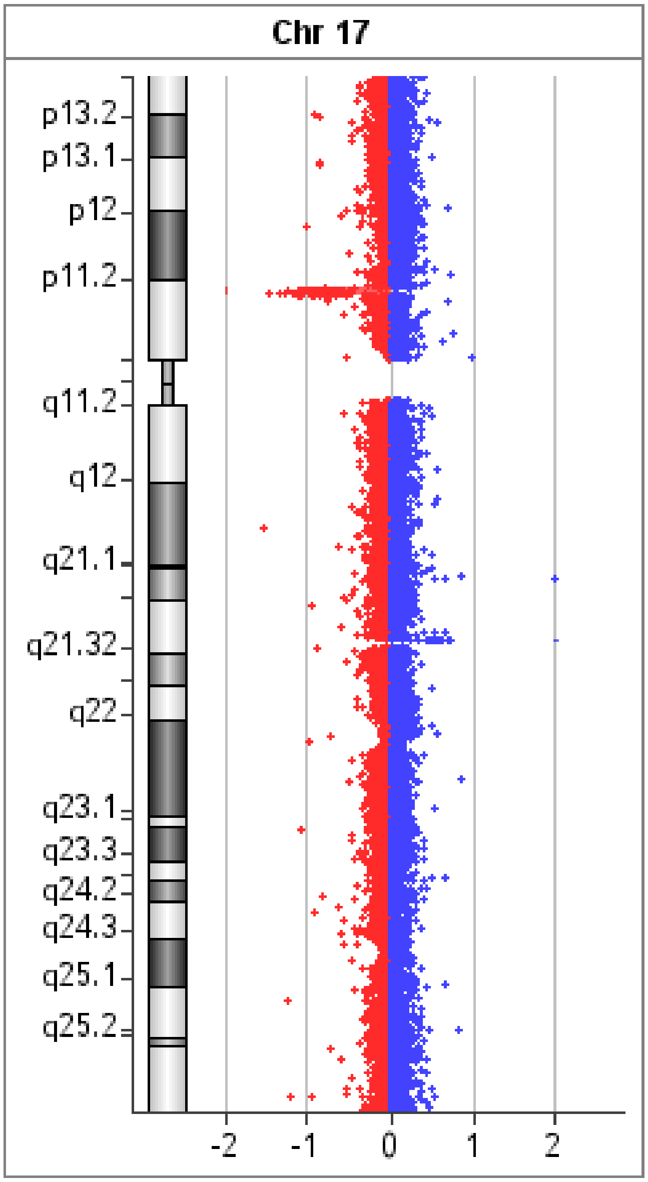

This is an intermediate report and not a final signed off report

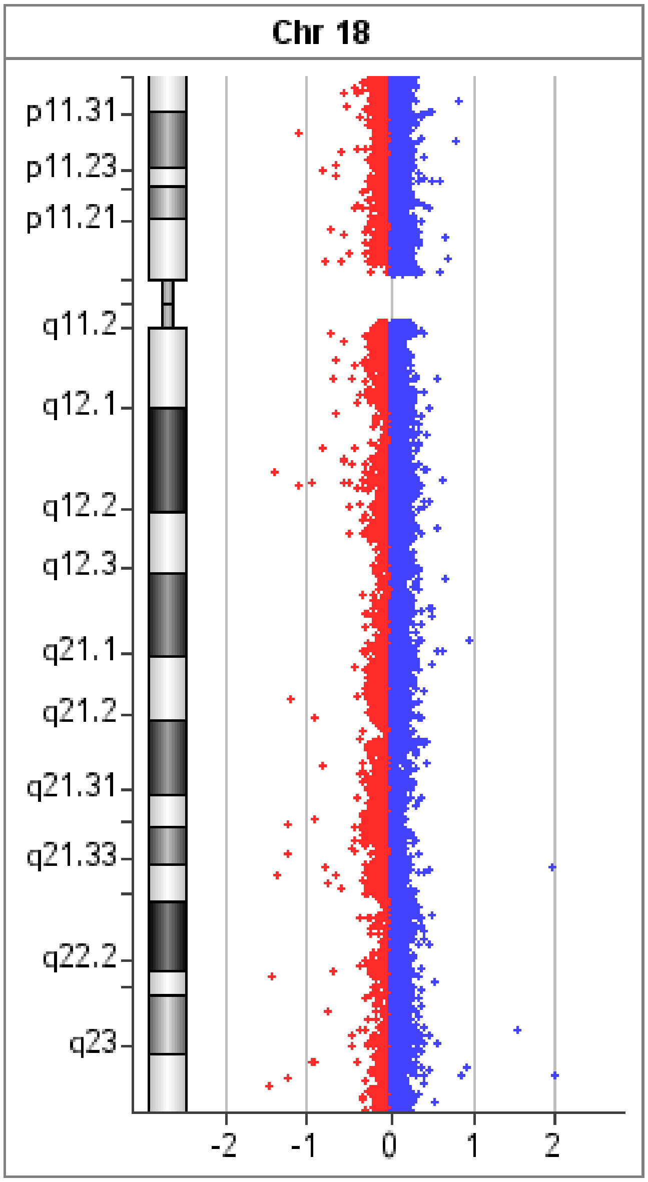

This is an intermediate report and not a final signed off report

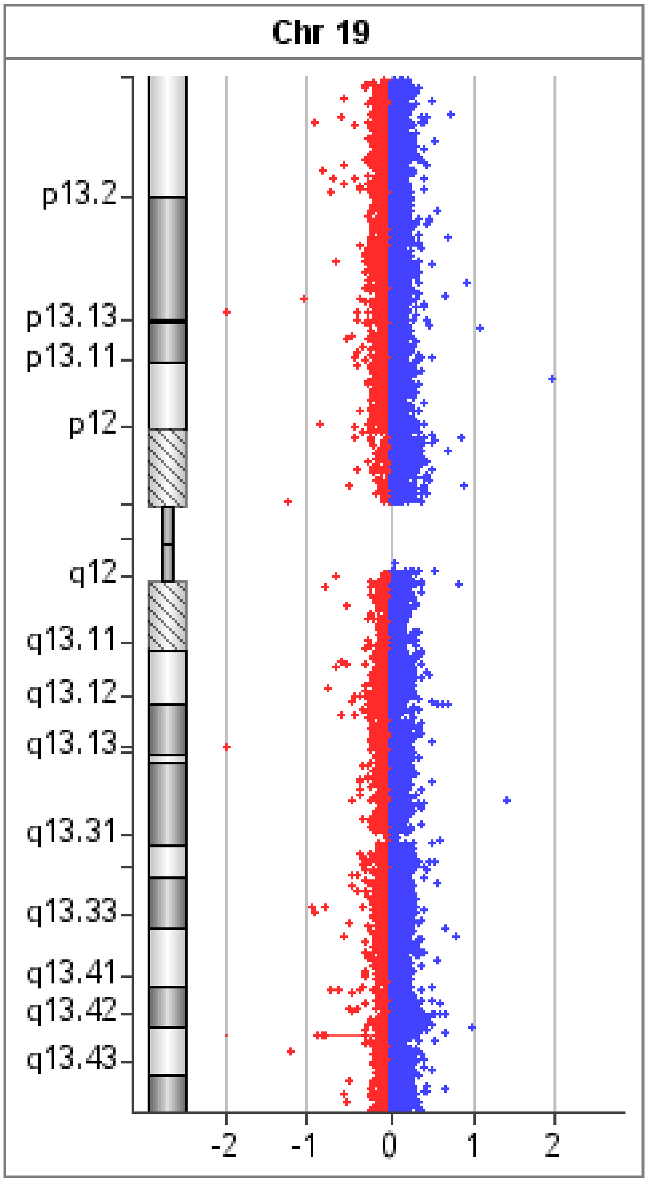

This is an intermediate report and not a final signed off report

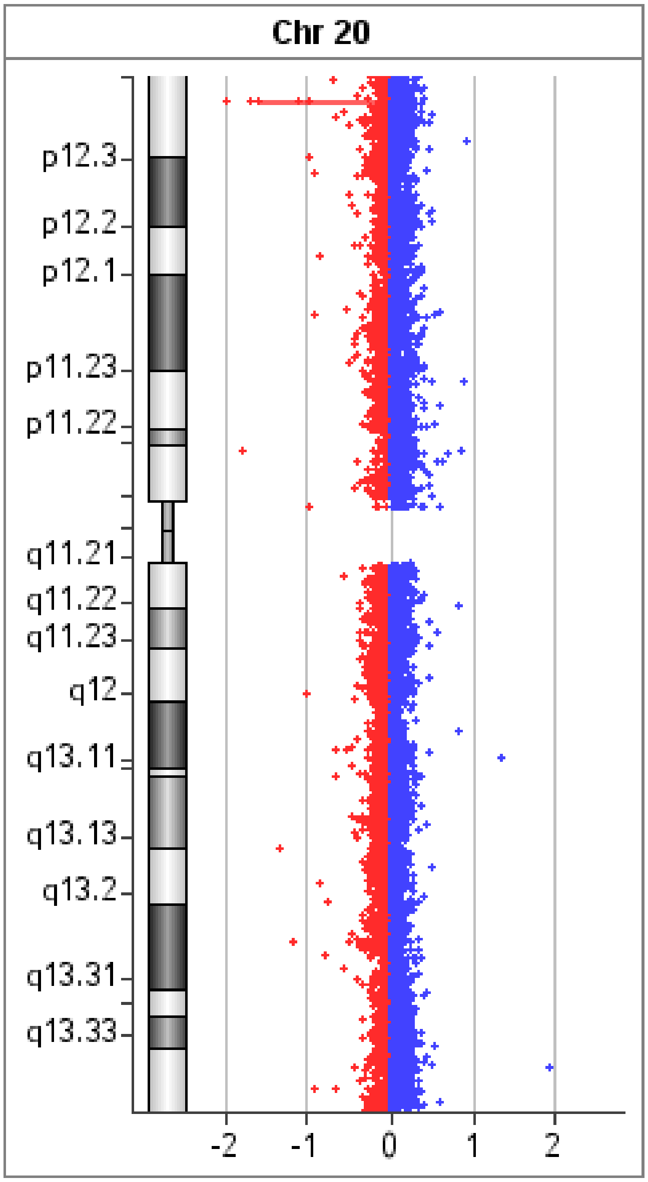

This is an intermediate report and not a final signed off report

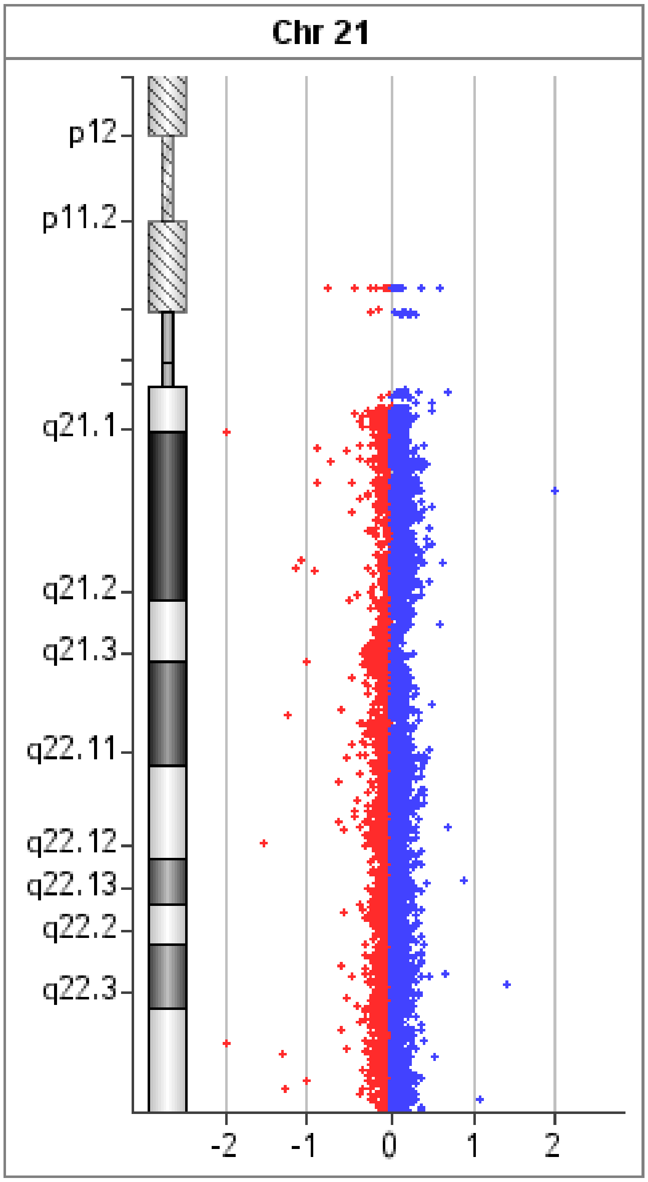

This is an intermediate report and not a final signed off report

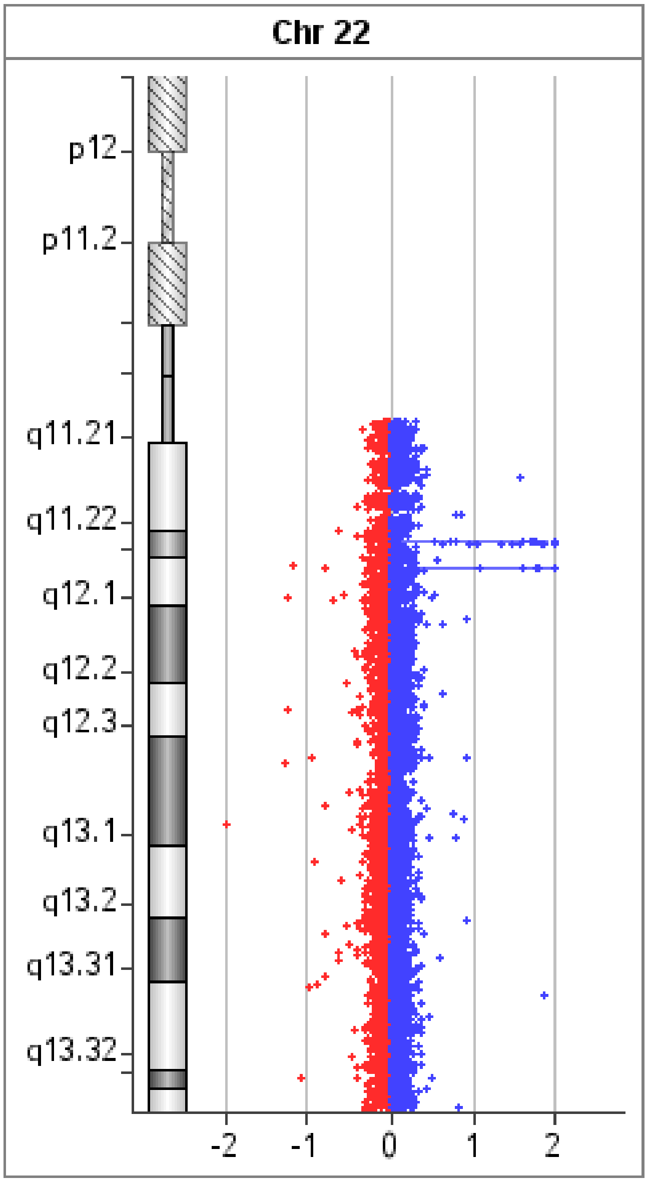

This is an intermediate report and not a final signed off report

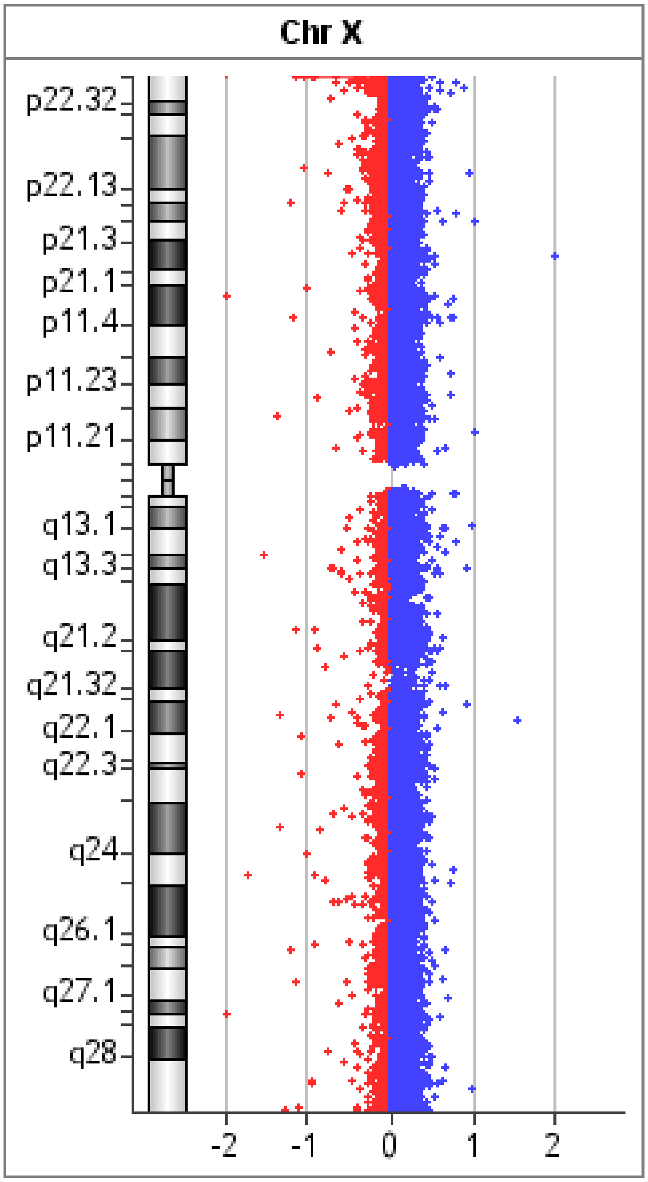

This is an intermediate report and not a final signed off report

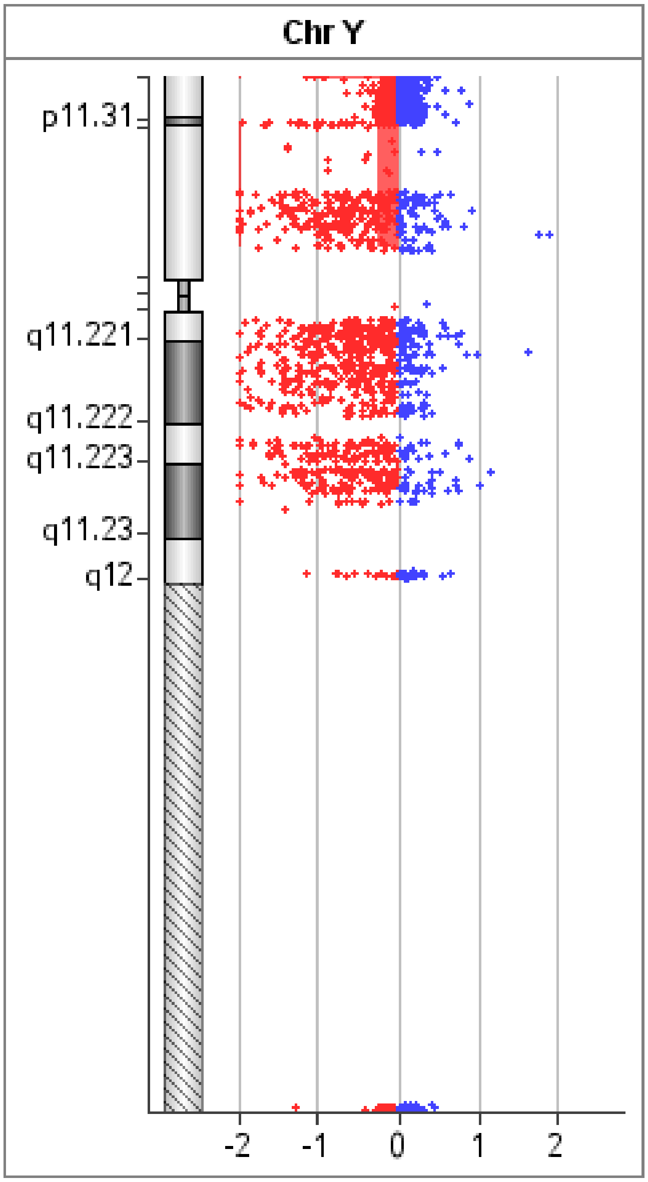

This is an intermediate report and not a final signed off report

Amp/Del Intervals Table

Chr	Start-Stop(bp)	Size(kb)	Cytoband	#Probes	Amp/Gain/ Loss/Del	Annotations	Classifications
chr17	16581147-17194128	613	p11.2	82	-0.820469	CCDC144A, LOC162632, FAM106C...	Pathogenic CNV

Amp=Amplification
Del=Deletion

Total Amp/Del Intervals: 1

This is an intermediate report and not a final signed off report

ISCN Nomenclature

arr 17p11.2(16,581,147-17,194,128)x1

This is an intermediate report and not a final signed off report

Analysis Settings

Design	021850_20111015
Genome	hg19
Threshold	6.0
GC Correction	ON
Centralization (legacy)	OFF
SNP Copy Number	OFF
Combine Replicates (Intra Array)	ON
Metric Set Filter	NONE
Feature Level Filter	gIsSaturated = true OR rIsSaturated = true OR gIsFeatNonUnifOL = true OR rIsFeatNonUnifOL = true OR LogRatio = 0; Include matching values=false
LOH Filter	NONE
Show Flat Intervals	false

Sample Name	14-0045GM-0039D-Wel,C-252185022597_1_1
Aberration Algorithm	ADM-2
Fuzzy Zero	OFF
Window Size	2Kb
Diploid Peak Centralization	ON
LOH	OFF
Array Level Filter	NONE
Aberration Filter	Minimum Number of Probes for Amplification >= 3 AND Nesting Level <= 100 AND Minimum Avg. Absolute Log Ratio for Amplification >= 0.25 AND Minimum Size (Kb) of Region for Amplification >= 0.0 AND Minimum Size (Kb) of Region for Deletion >= 0.0 AND Minimum Number of Probes for Deletion >= 3 AND Minimum Avg. Absolute Log Ratio for Deletion >= 0.25
Design Level Filter	Homology = 0 OR IsPseudoautosomal = 1
Genomic Boundary	OFF
Template Name	OUHSC CGH report

This is an intermediate report and not a final signed off report

Notes

Sample Notes

No notes available.

Amp/Del Interval Notes

No notes available.

This is an intermediate report and not a final signed off report
