# Supplementary material for: Genomic Copy Number Variants in CML Patients With the Philadelphia Chromosome (Ph+): An Update
Source: Front Genet. 2021 Aug 10;12:697009. doi: 10.3389/fgene.2021.697009 (PMC8383316; doi:10.3389/fgene.2021.697009)
Supplement: Supplementary file 4 [file Data_Sheet_4.PDF]

Sample Information

DerivativeOfLogRatioSD	:	0.236696
Red Sample	:	
Polarity	:	1
Global Display Name	:	252185022602_1_2
Array ID	:	252185022602_1_2
Green Sample	:	Agilent Euro Female
Intermediate Report by	:	OUHSC\xwang3

This is an intermediate report and not a final signed off report

Genome View (Amp/Del)

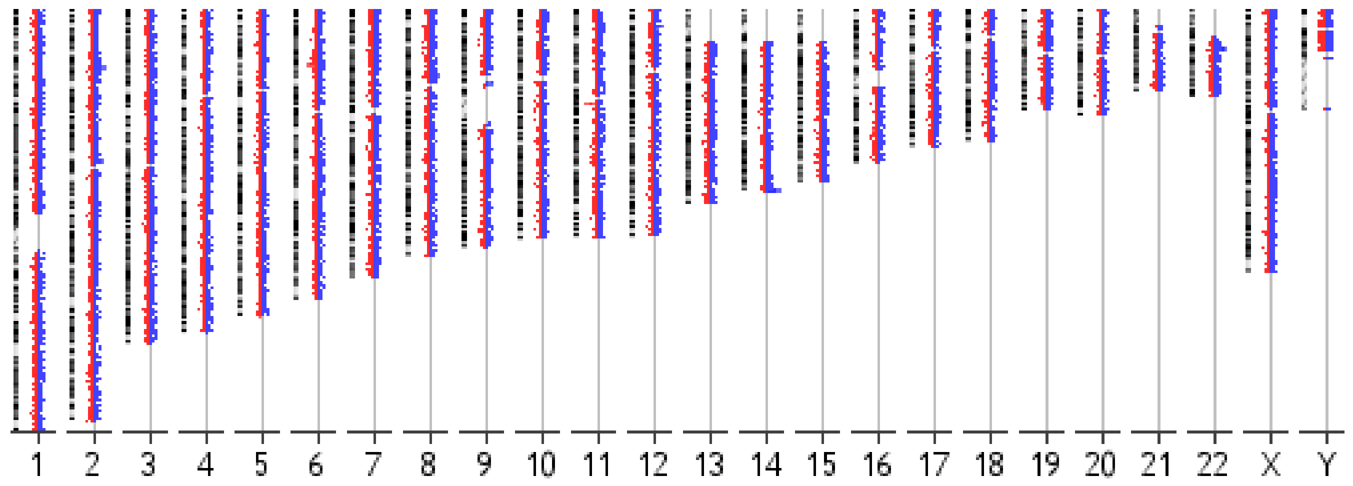

This is an intermediate report and not a final signed off report

Chromosome Views (Amp/Del)

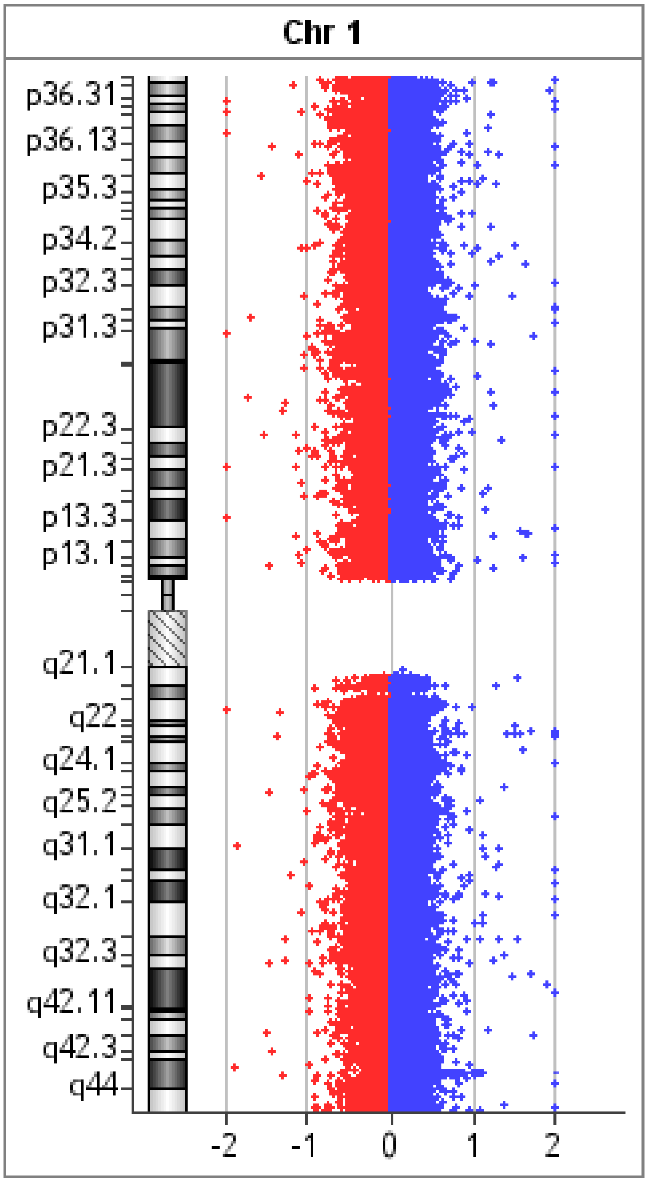

This is an intermediate report and not a final signed off report

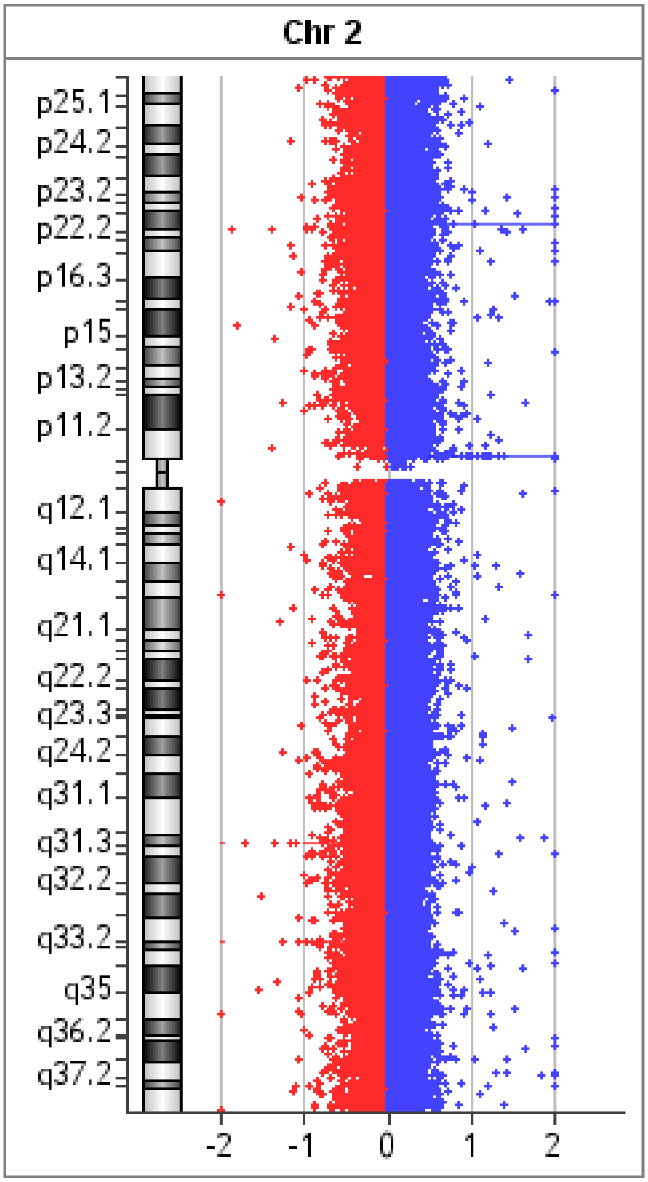

This is an intermediate report and not a final signed off report

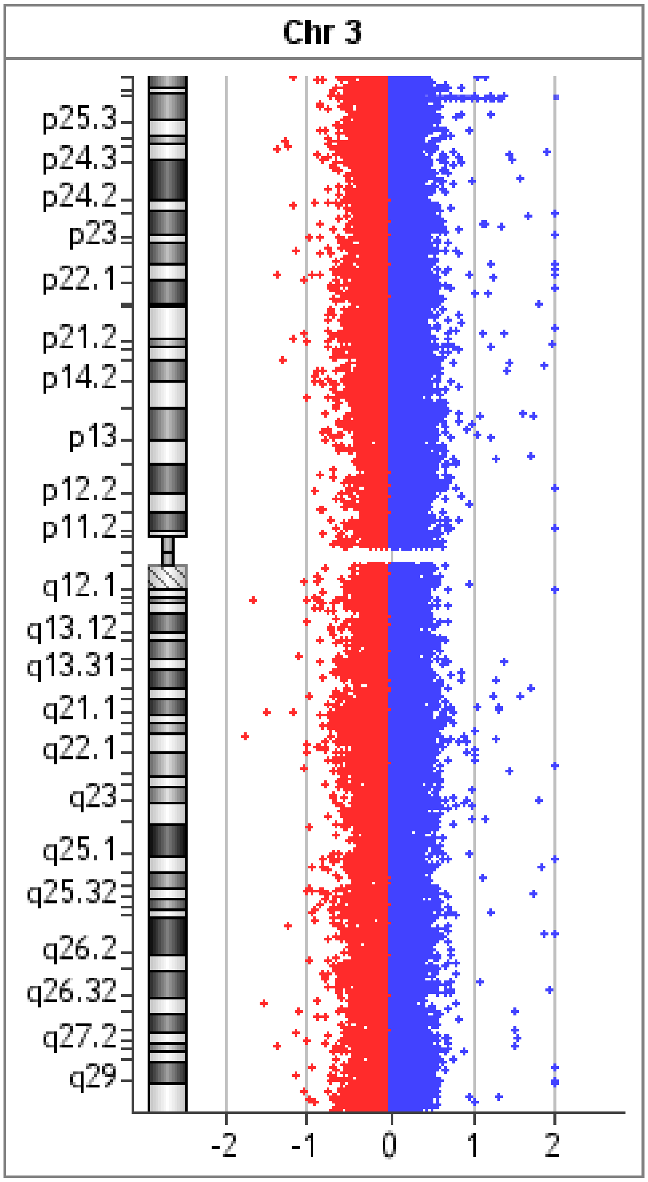

This is an intermediate report and not a final signed off report

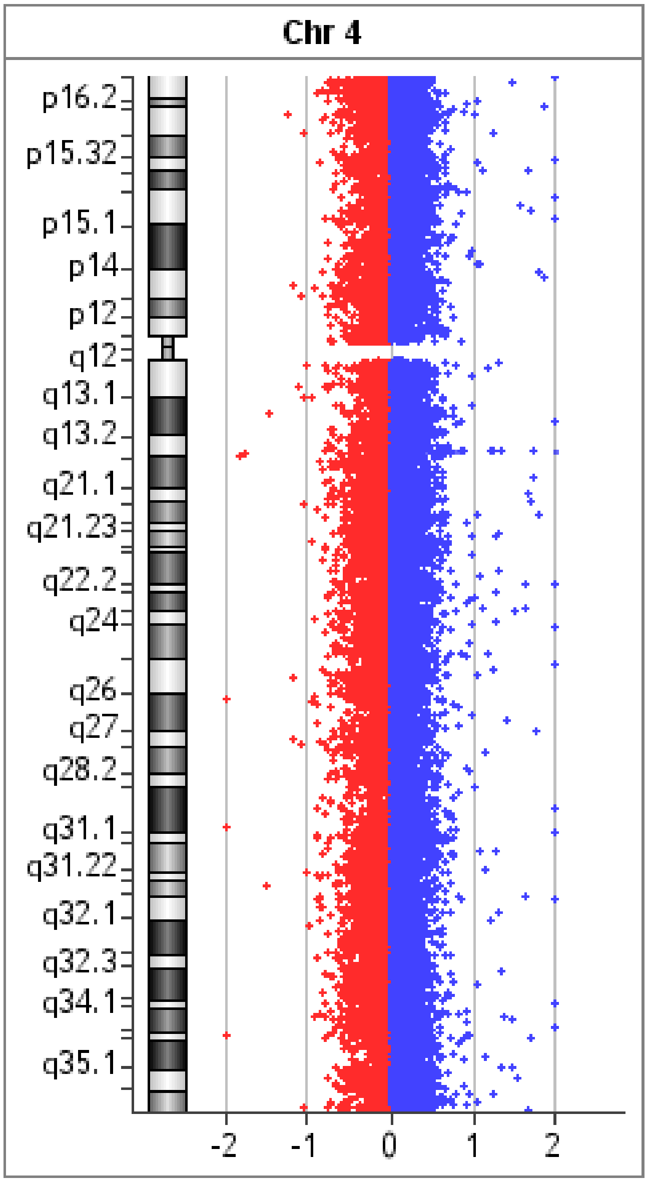

This is an intermediate report and not a final signed off report

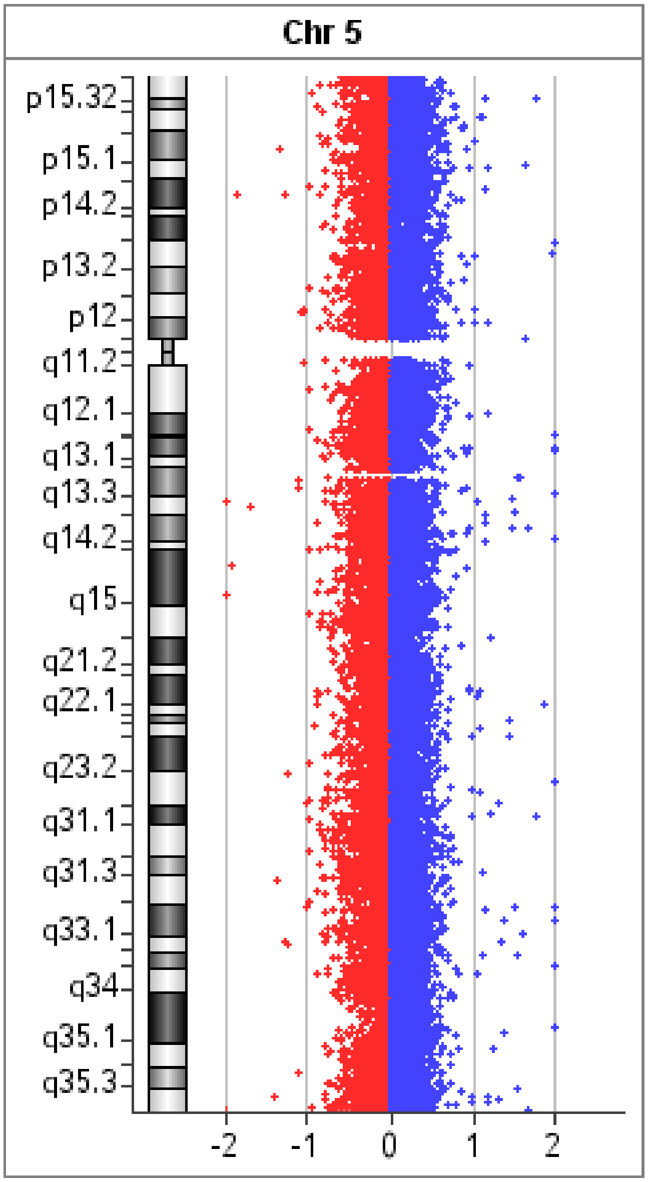

This is an intermediate report and not a final signed off report

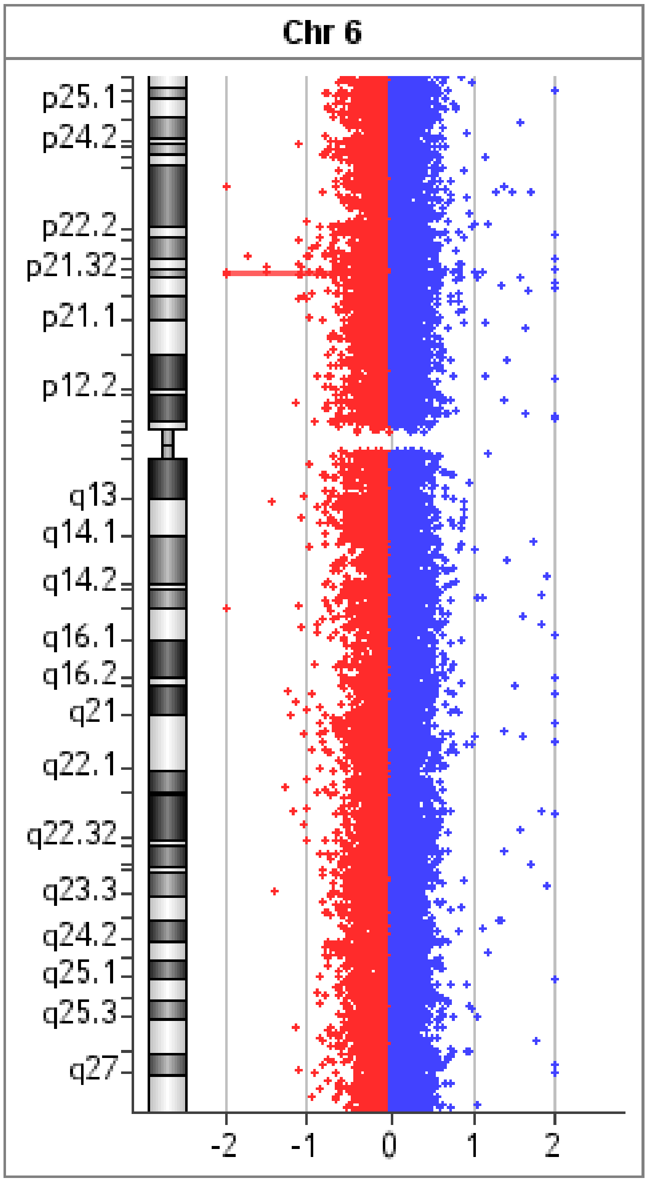

This is an intermediate report and not a final signed off report

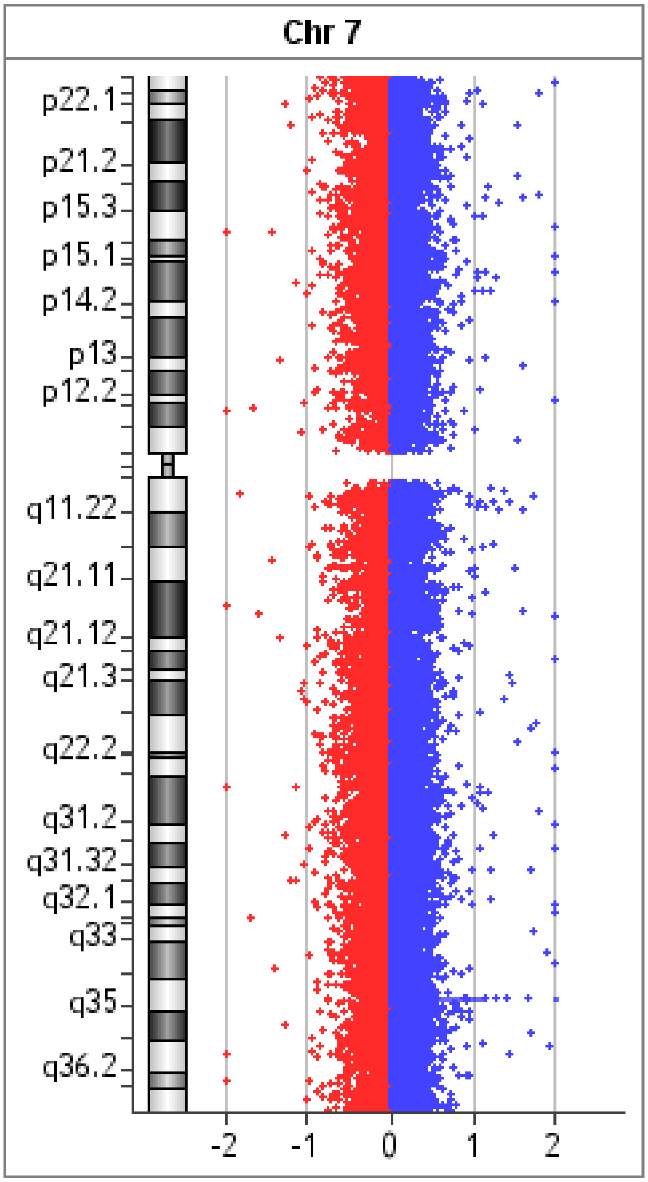

This is an intermediate report and not a final signed off report

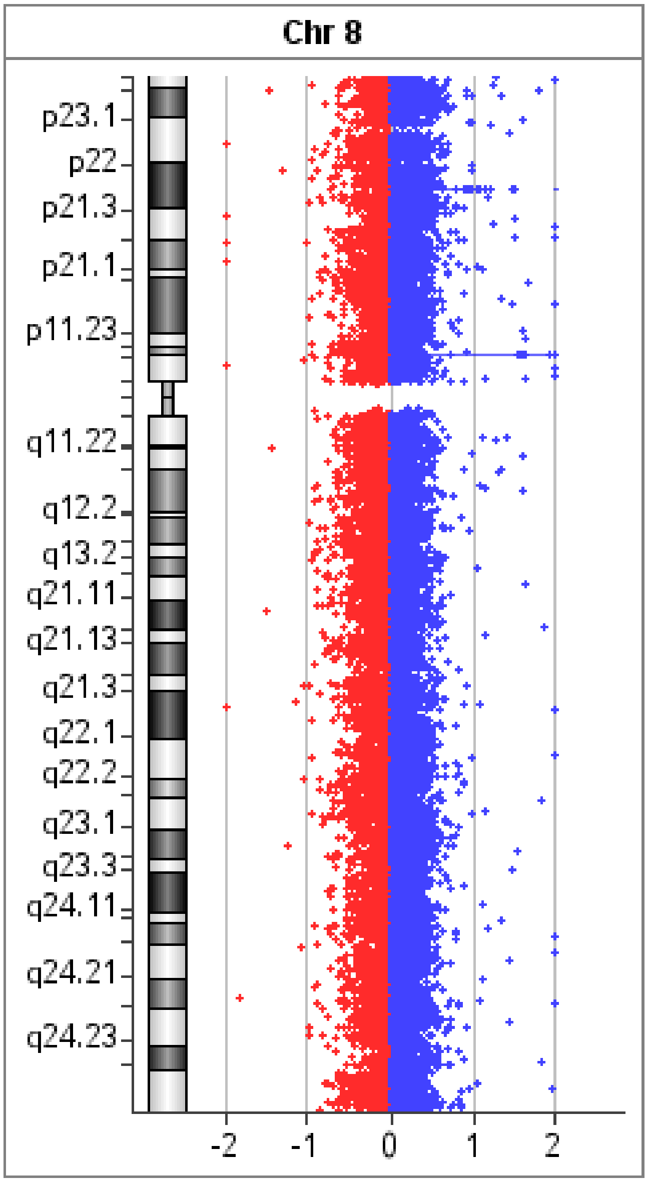

This is an intermediate report and not a final signed off report

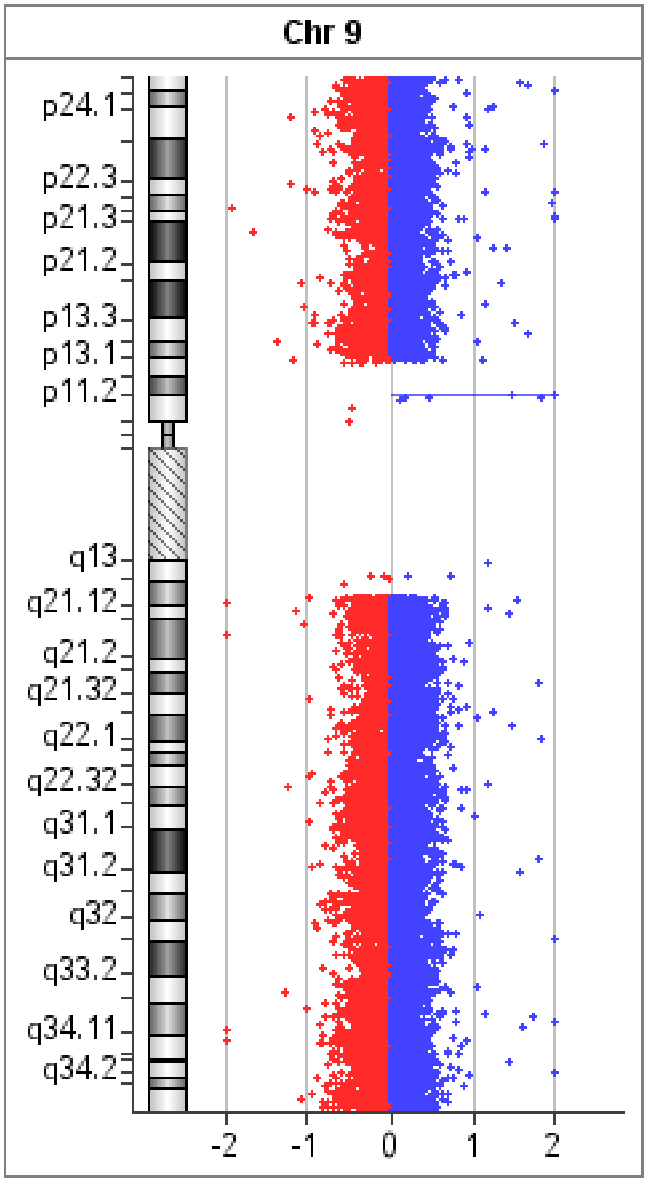

This is an intermediate report and not a final signed off report

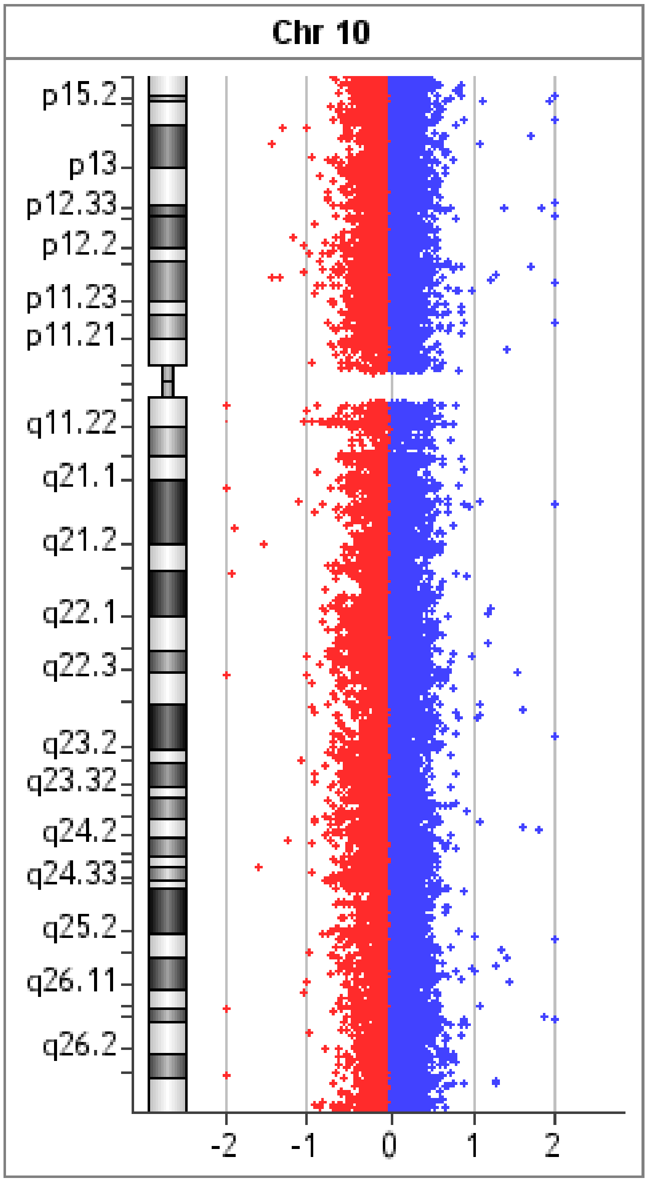

This is an intermediate report and not a final signed off report

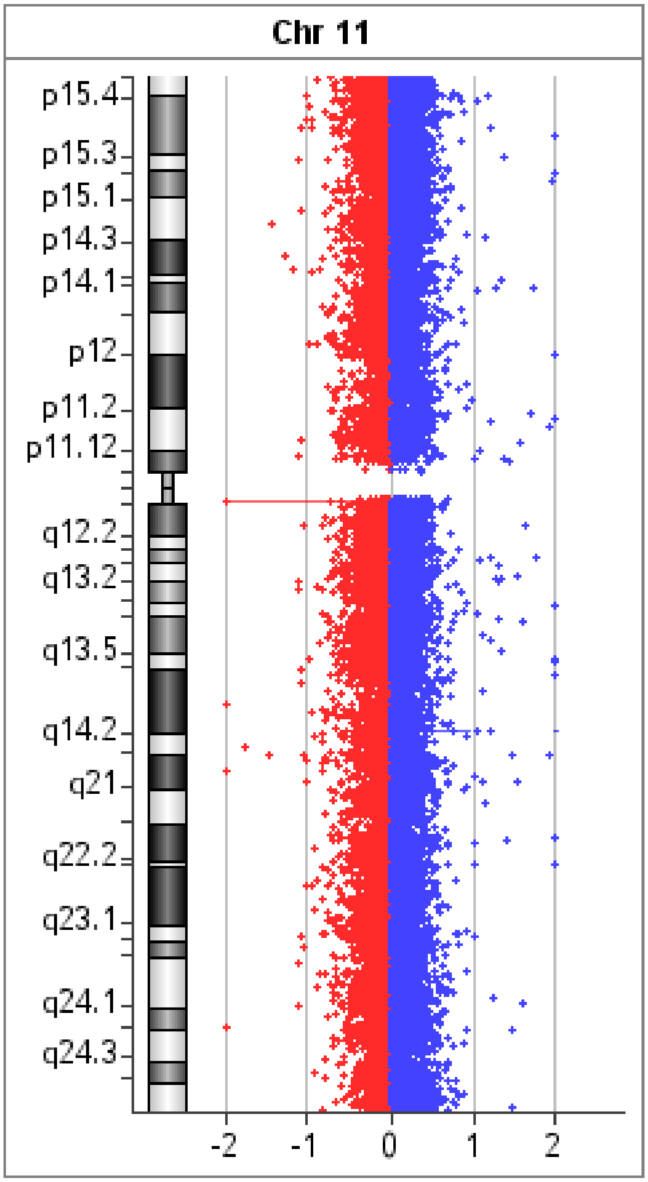

This is an intermediate report and not a final signed off report

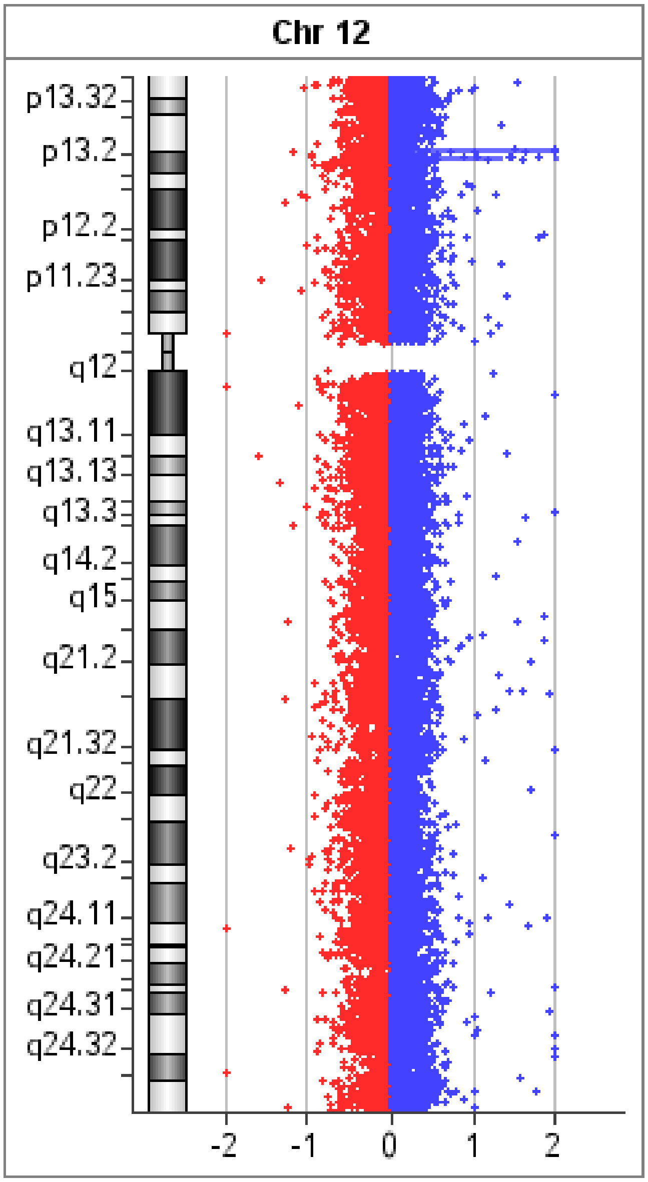

This is an intermediate report and not a final signed off report

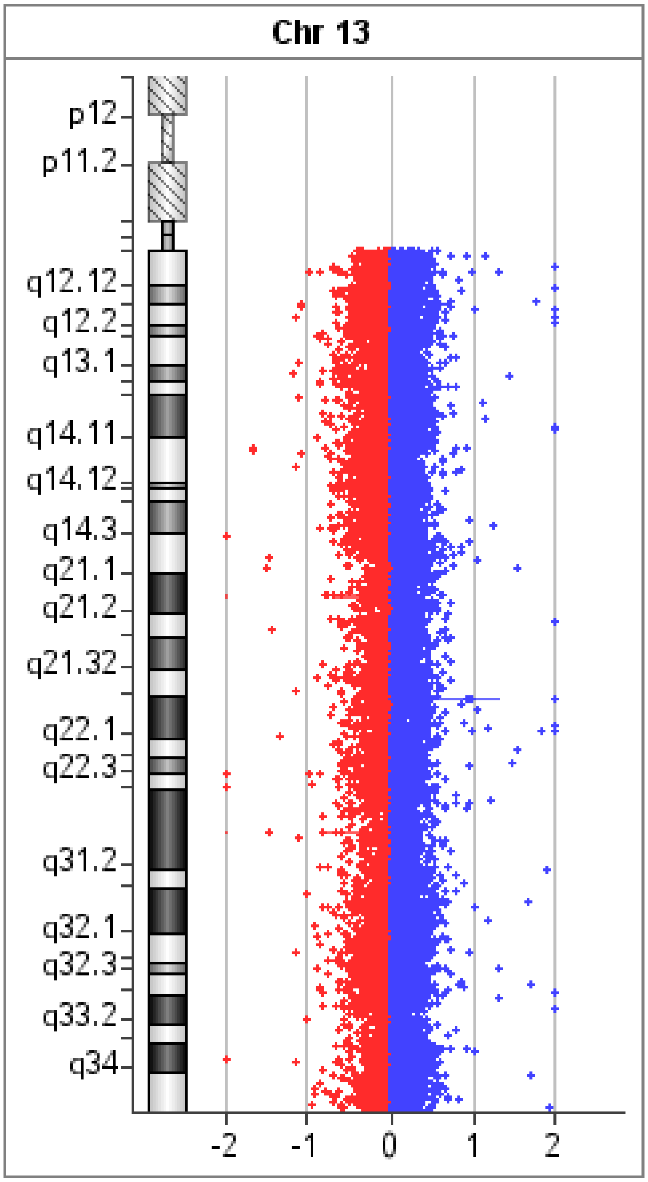

This is an intermediate report and not a final signed off report

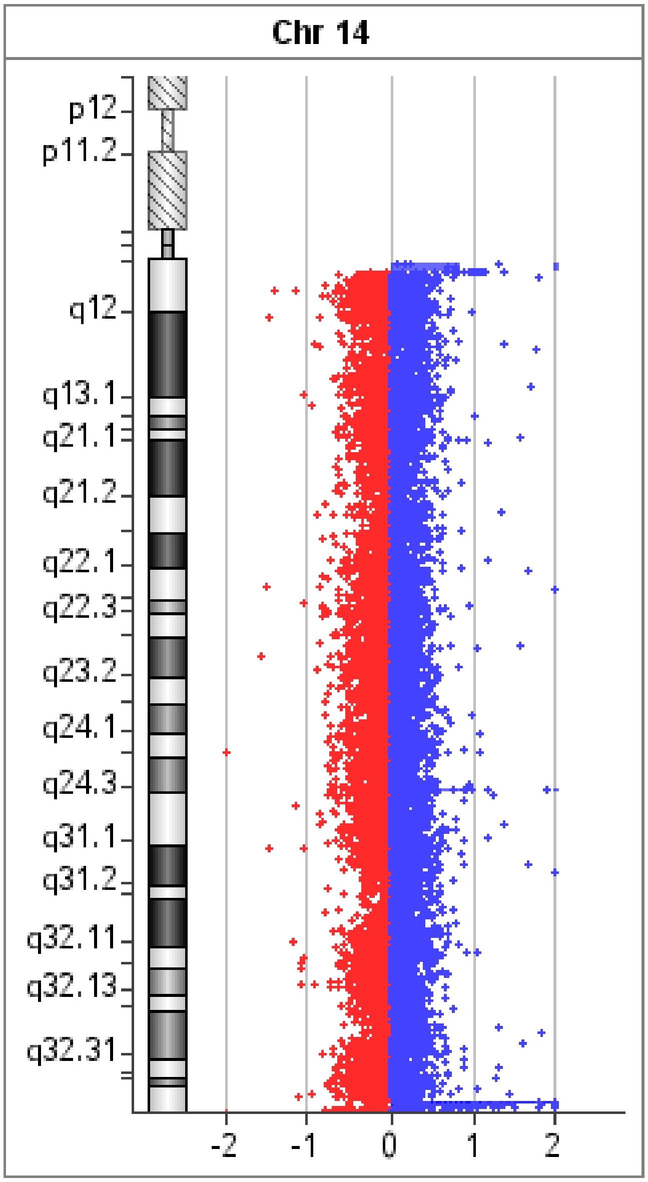

This is an intermediate report and not a final signed off report

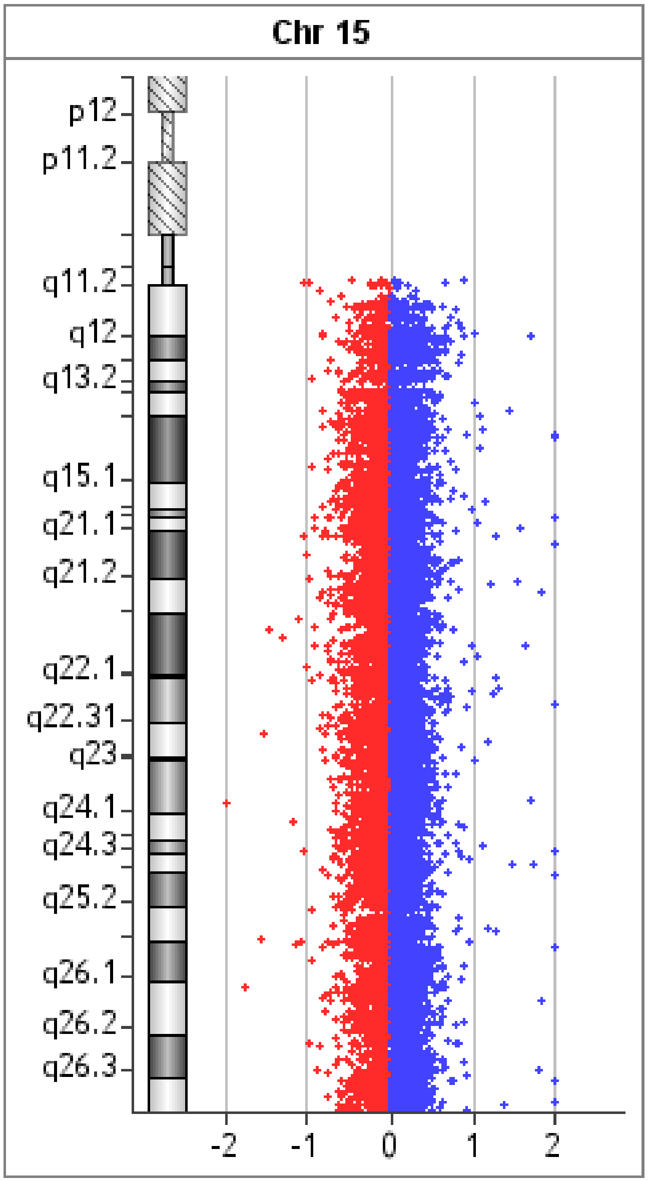

This is an intermediate report and not a final signed off report

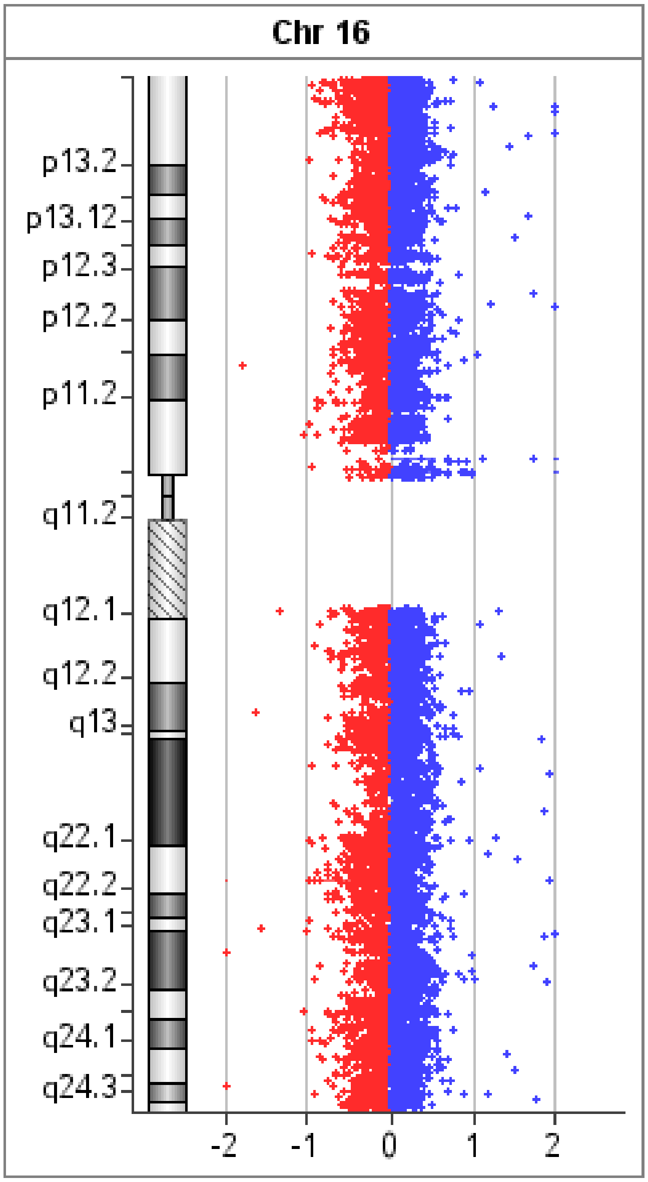

This is an intermediate report and not a final signed off report

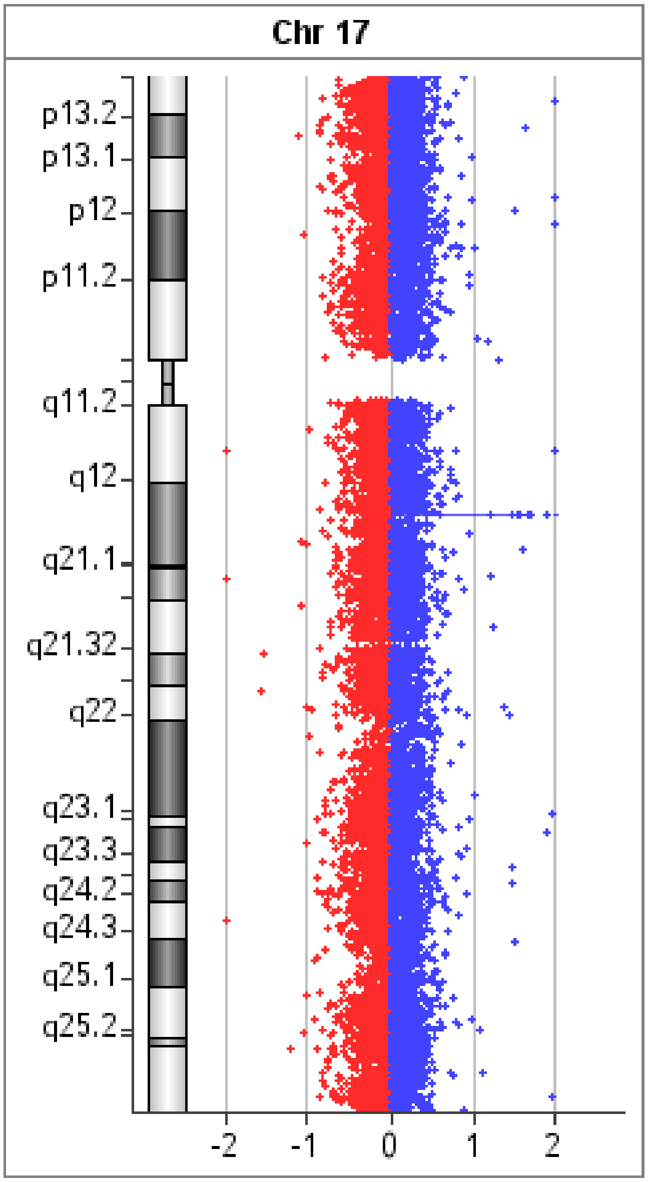

This is an intermediate report and not a final signed off report

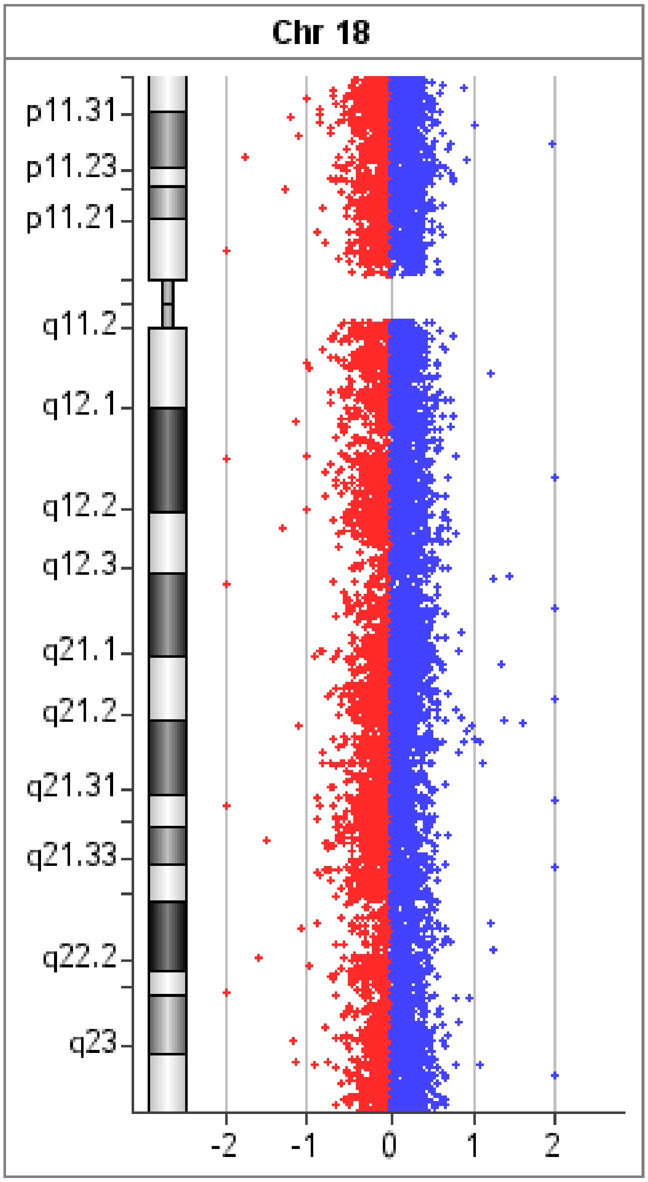

This is an intermediate report and not a final signed off report

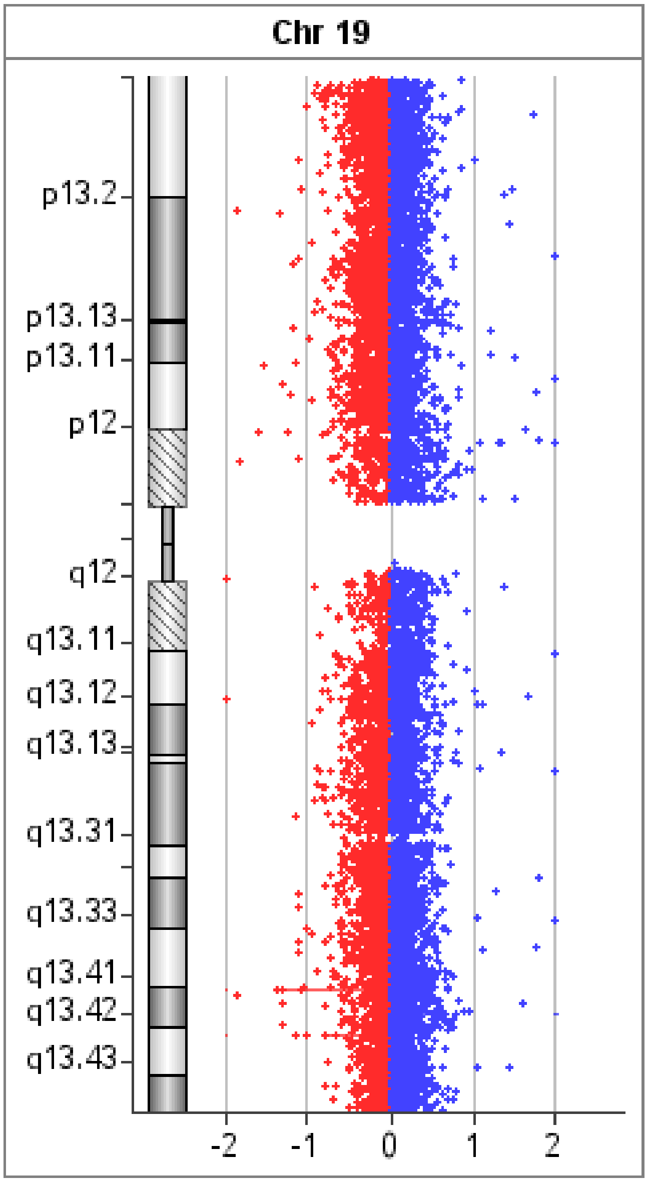

This is an intermediate report and not a final signed off report

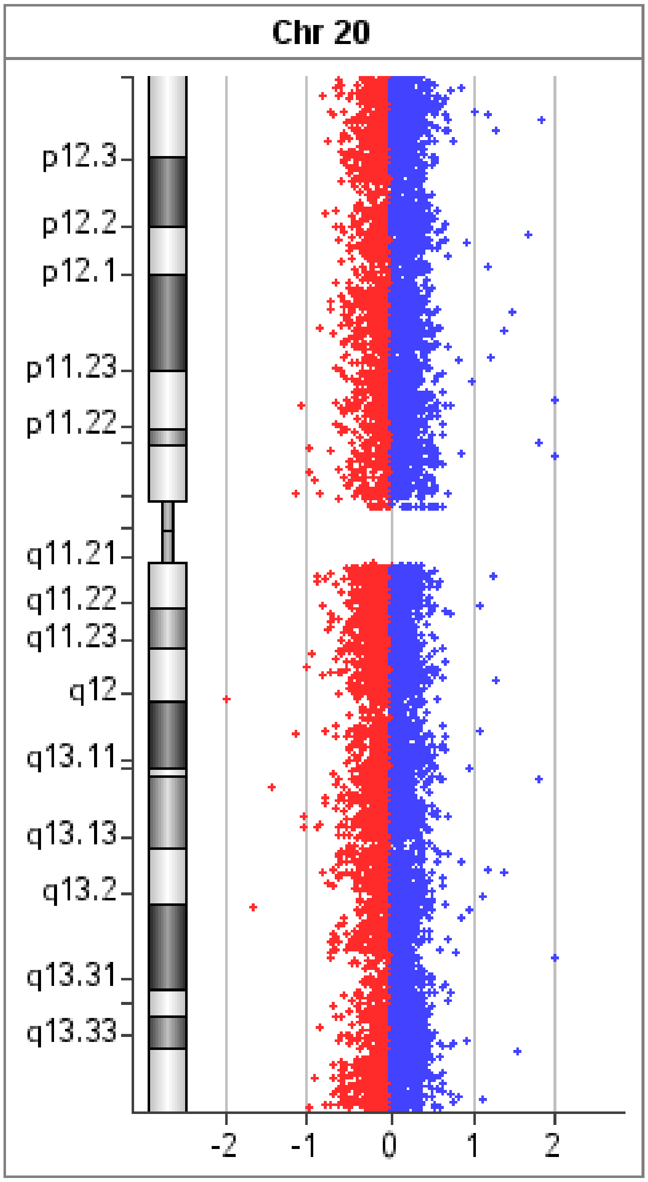

This is an intermediate report and not a final signed off report

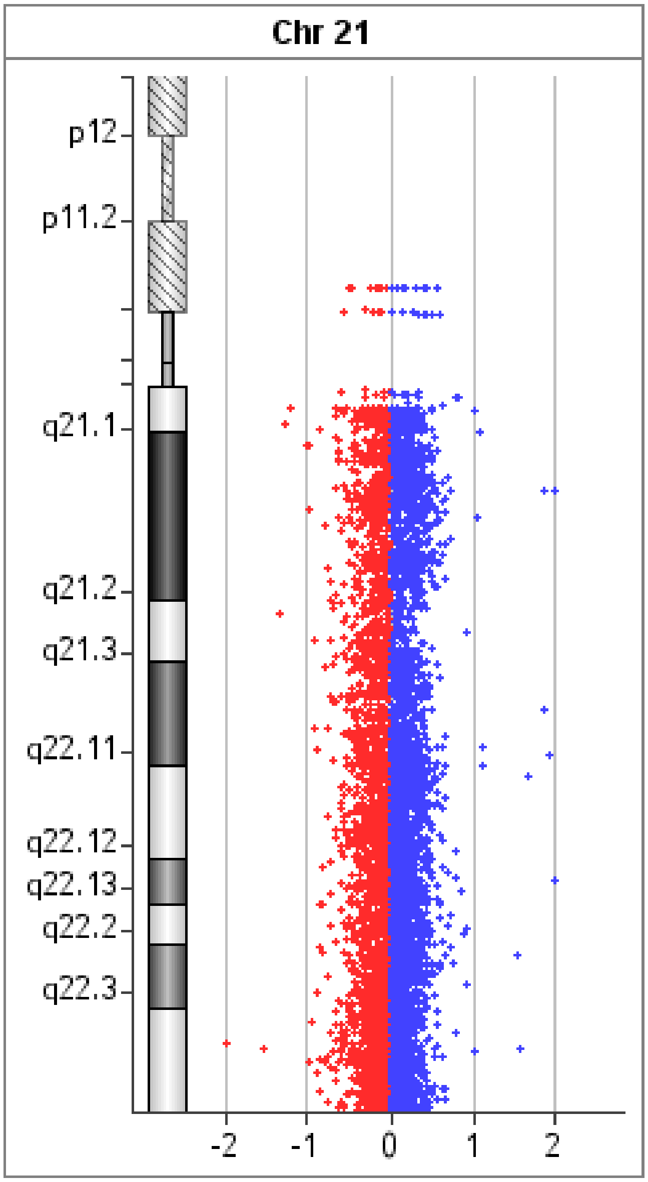

This is an intermediate report and not a final signed off report

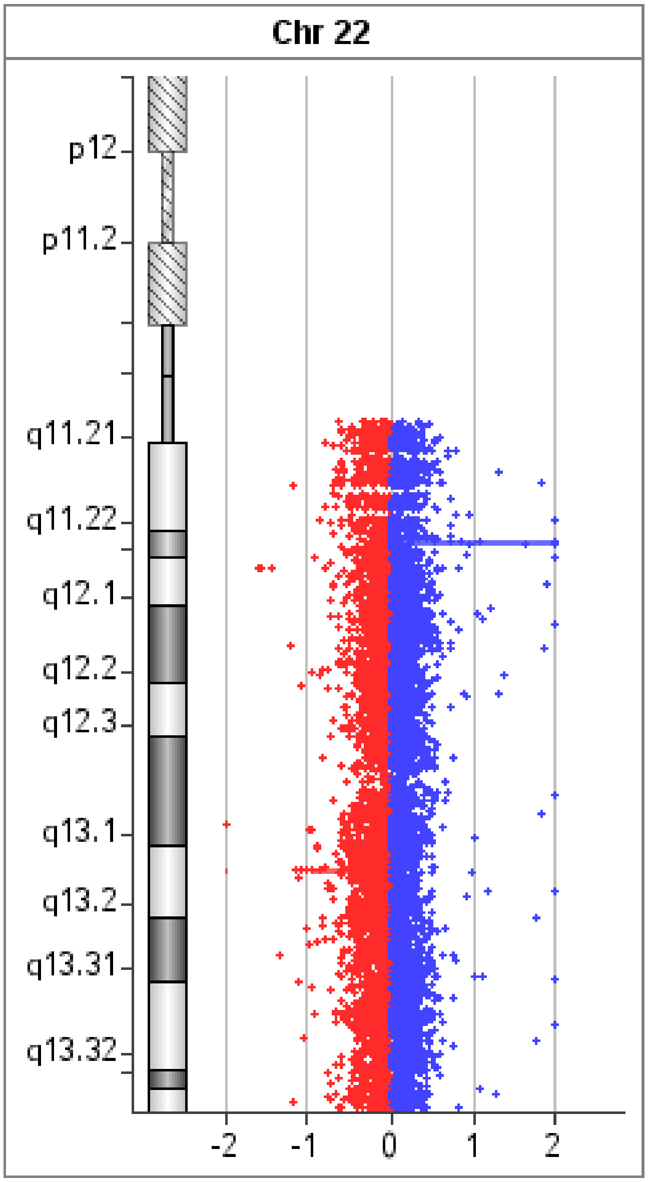

This is an intermediate report and not a final signed off report

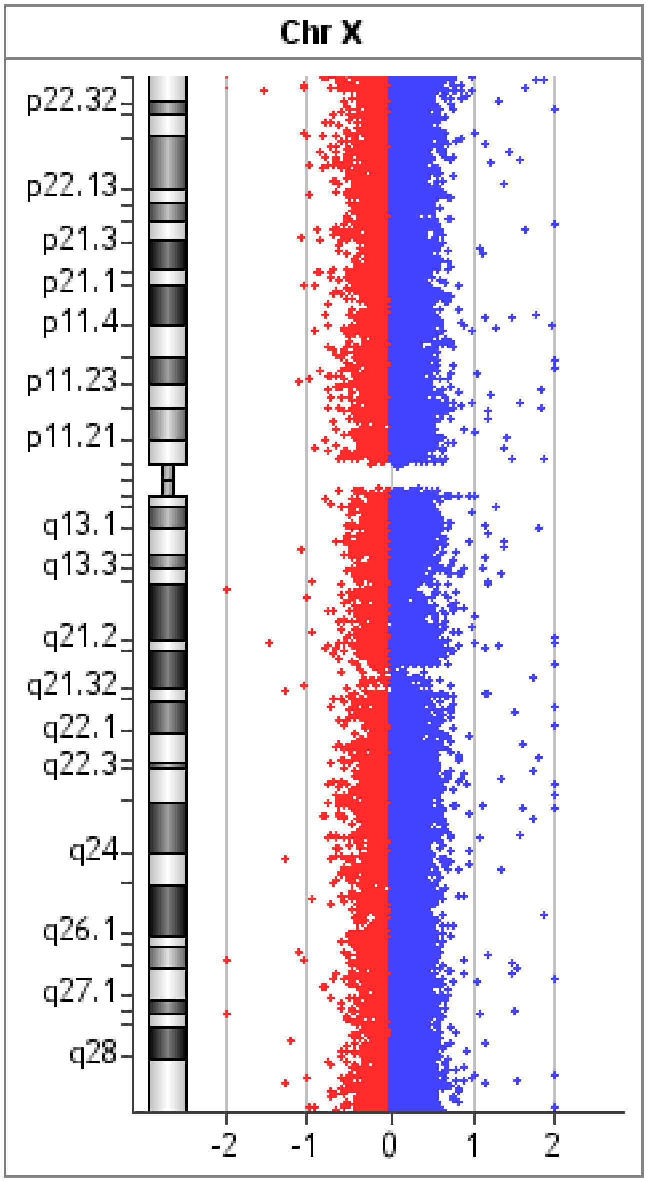

This is an intermediate report and not a final signed off report

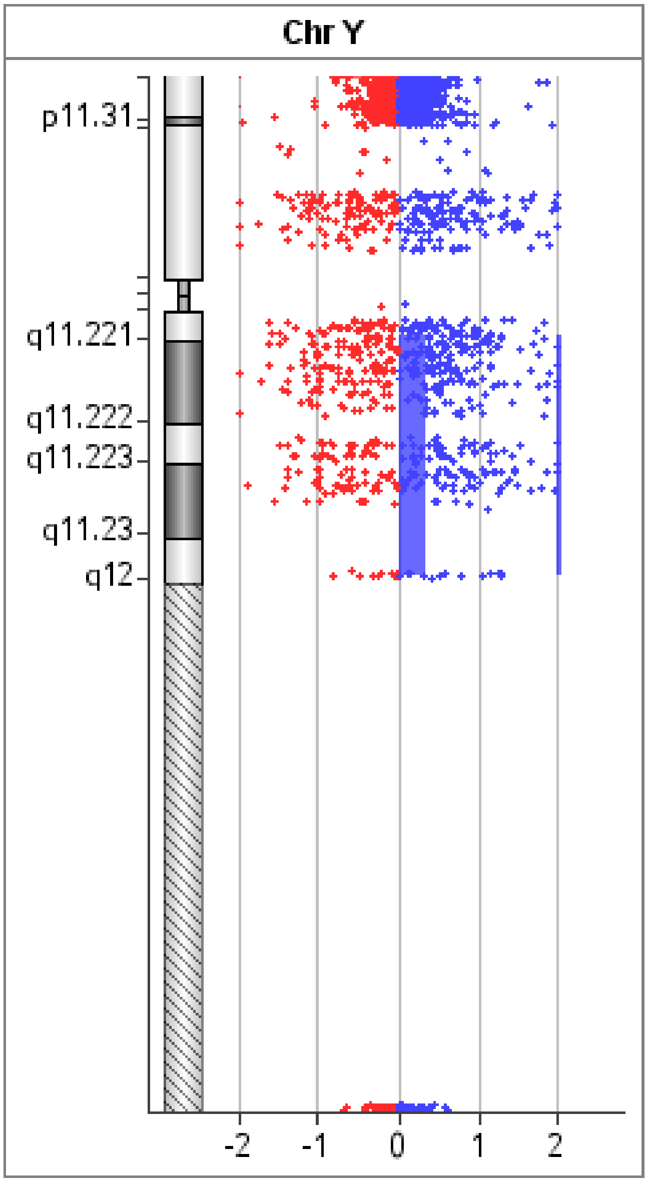

This is an intermediate report and not a final signed off report

Amp/Del Intervals Table

Chr	Start-Stop(bp)	Size(kb)	Cytoband	#Probes	Amp/Gain/ Loss/Del	Annotations	Classifications
chr1	239954040-240359460	405	q43	68	0.690510	CHRM3, RPS7P5, FMN2	Unknown CNV

Amp=Amplification
Del=Deletion

Total Amp/Del Intervals: 1

This is an intermediate report and not a final signed off report

ISCN Nomenclature

arr 1q43(239,954,040-240,359,460)x3

This is an intermediate report and not a final signed off report

Analysis Settings

Design	:	021850_20111015	Sample Name	:	252185022602_1_2
Genome	:	hg19	Aberration Algorithm	:	ADM-2
Threshold	:	6.0	Fuzzy Zero	:	OFF
GC Correction	:	ON	Window Size	:	2Kb
Centralization (legacy)	:	OFF	Diploid Peak Centralization	:	ON
SNP Copy Number	:	OFF	LOH	:	OFF
Combine Replicates (Intra Array)	:	ON	Array Level Filter	:	NONE
Metric Set Filter	:	NONE	Aberration Filter	:	Minimum Number of Probes for Amplification >= 3 AND Nesting Level <= 100 AND Minimum Avg. Absolute Log Ratio for Amplification >= 0.25 AND Minimum Size (Kb) of Region for Amplification >= 0.0 AND Minimum Size (Kb) of Region for Deletion >= 0.0 AND Minimum Number of Probes for Deletion >= 3 AND Minimum Avg. Absolute Log Ratio for Deletion >= 0.25
Feature Level Filter	:	glsSaturated = true OR rIsSaturated = true OR glsFeatNonUnifOL = true OR rIsFeatNonUnifOL = true OR LogRatio = 0; Include matching values=false	Design Level Filter	:	Homology = 0 OR IsPseudoautosomal = 1
LOH Filter	:	NONE	Genomic Boundary	:	OFF
Show Flat Intervals	:	false	Template Name	:	OUHSC CGH report

This is an intermediate report and not a final signed off report

Notes

Sample Notes

No notes available.

Amp/Del Interval Notes

No notes available.

This is an intermediate report and not a final signed off report
