# Supplementary material for: Genomic Copy Number Variants in CML Patients With the Philadelphia Chromosome (Ph+): An Update
Source: Front Genet. 2021 Aug 10;12:697009. doi: 10.3389/fgene.2021.697009 (PMC8383316; doi:10.3389/fgene.2021.697009)
Supplement: Supplementary file 5 [file Data_Sheet_5.PDF]

Sample Information

Green Sample	:	Agilent Euro Male
Array ID	:	252185022565_1_1
Global Display Name	:	12-0350D-252185022565_1_1
Polarity	:	1
Red Sample	:	
DerivativeOfLogRatioSD	:	0.164066
Intermediate Report by	:	OUHSC\xwang3

Genome View (Amp/Del)

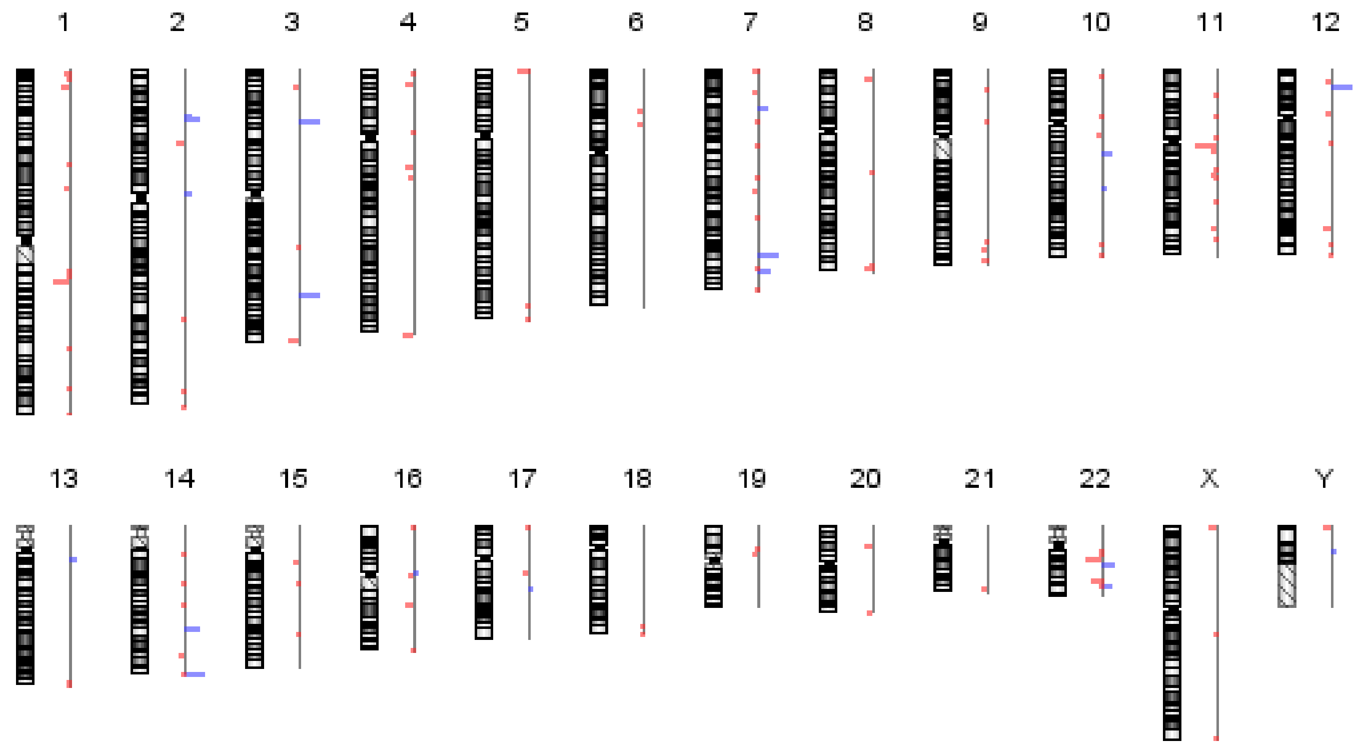

This is an intermediate report and not a final signed off report

Chromosome Views (Amp/Del)

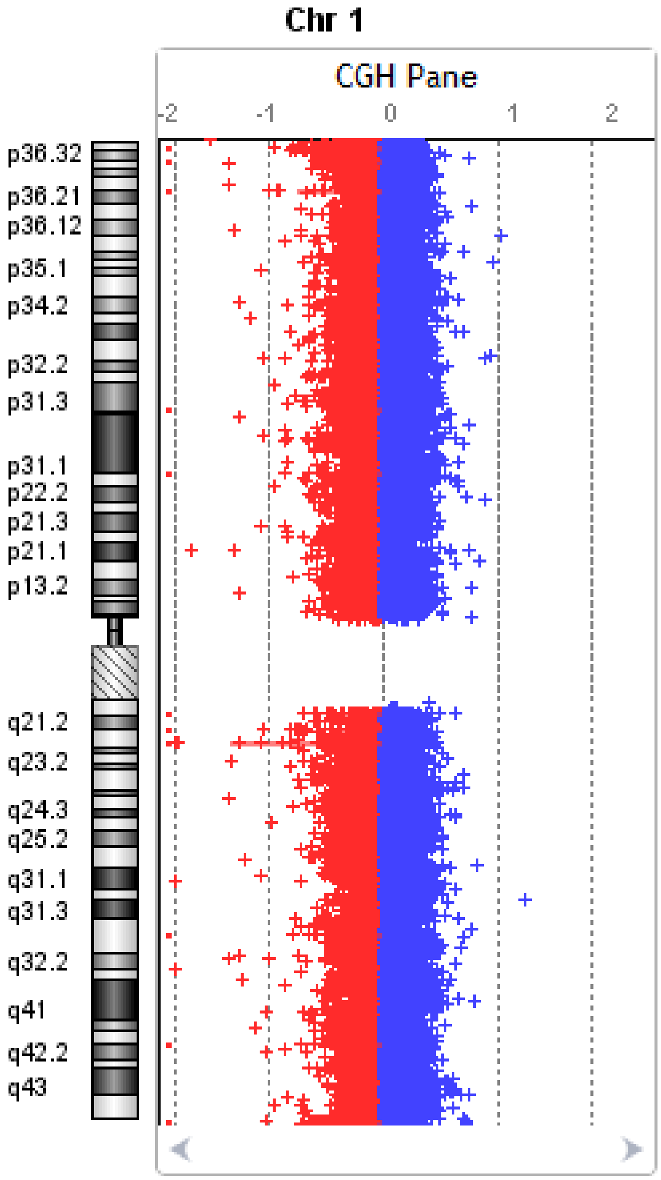

This is an intermediate report and not a final signed off report

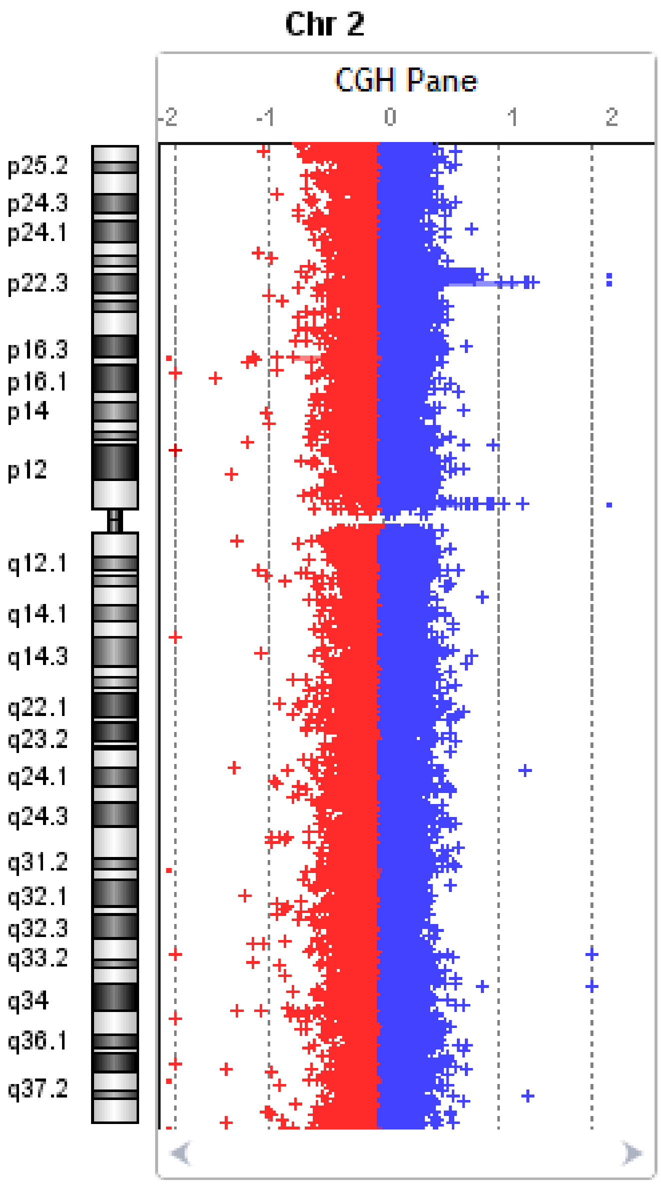

This is an intermediate report and not a final signed off report

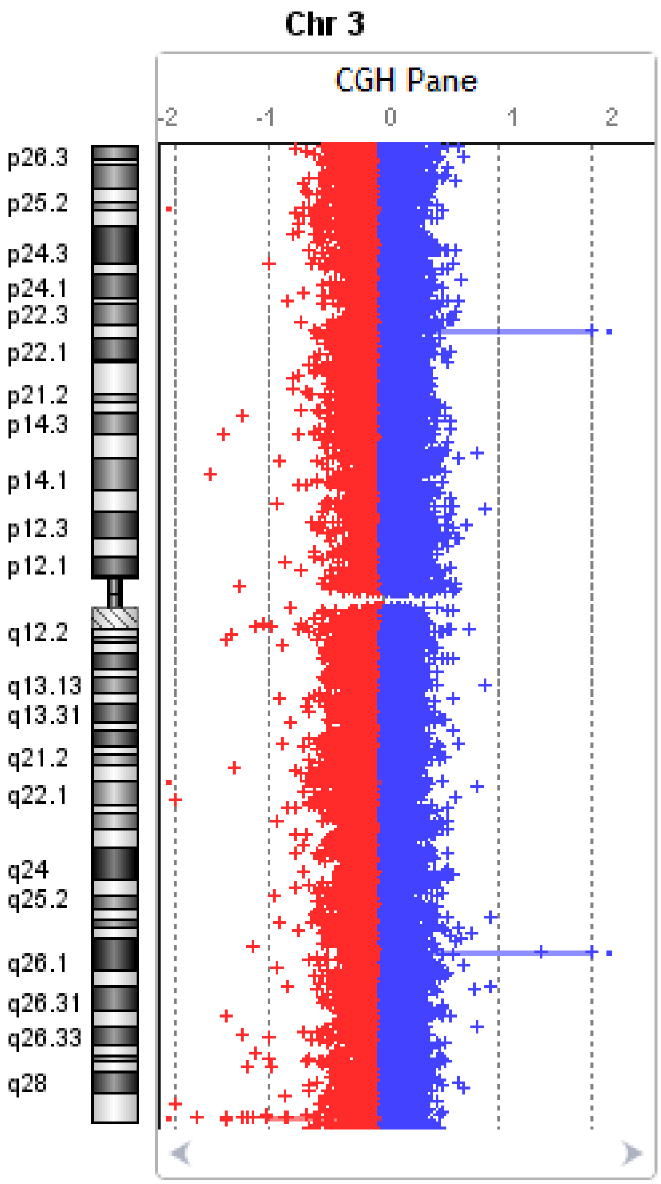

This is an intermediate report and not a final signed off report

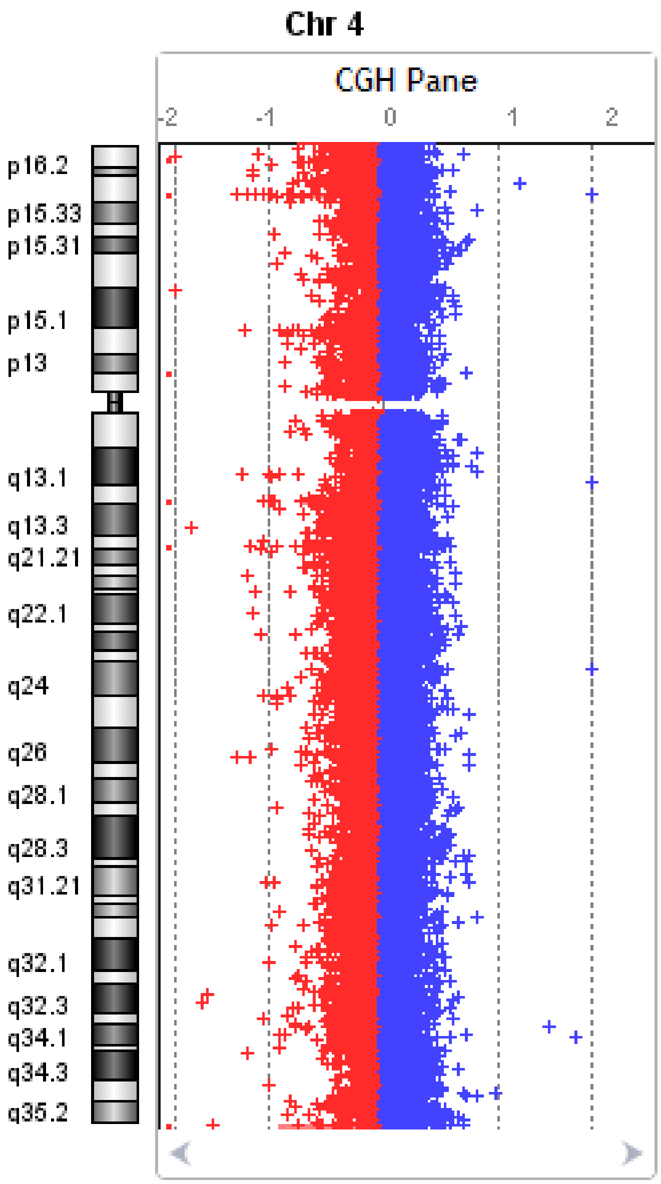

This is an intermediate report and not a final signed off report

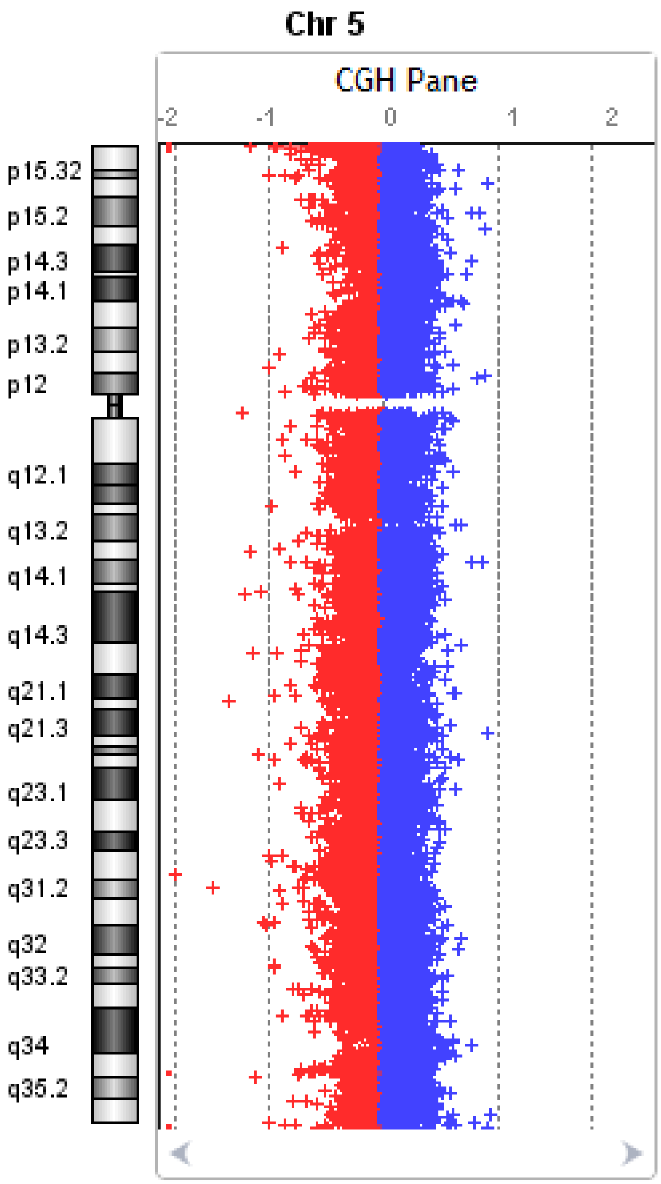

This is an intermediate report and not a final signed off report

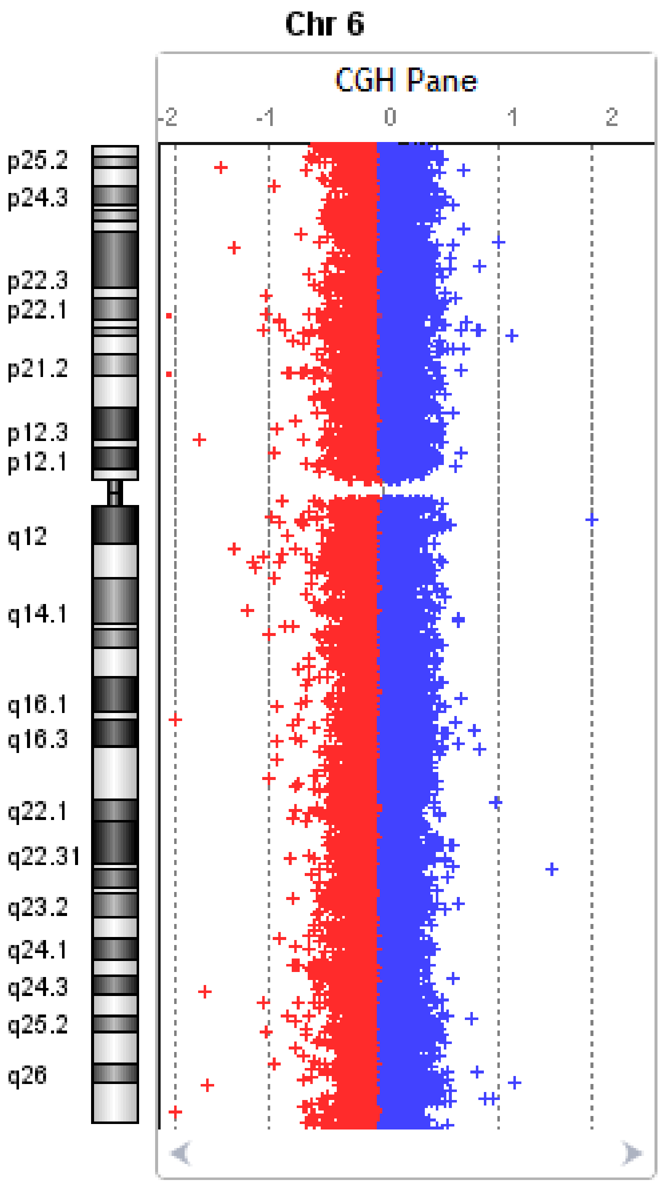

This is an intermediate report and not a final signed off report

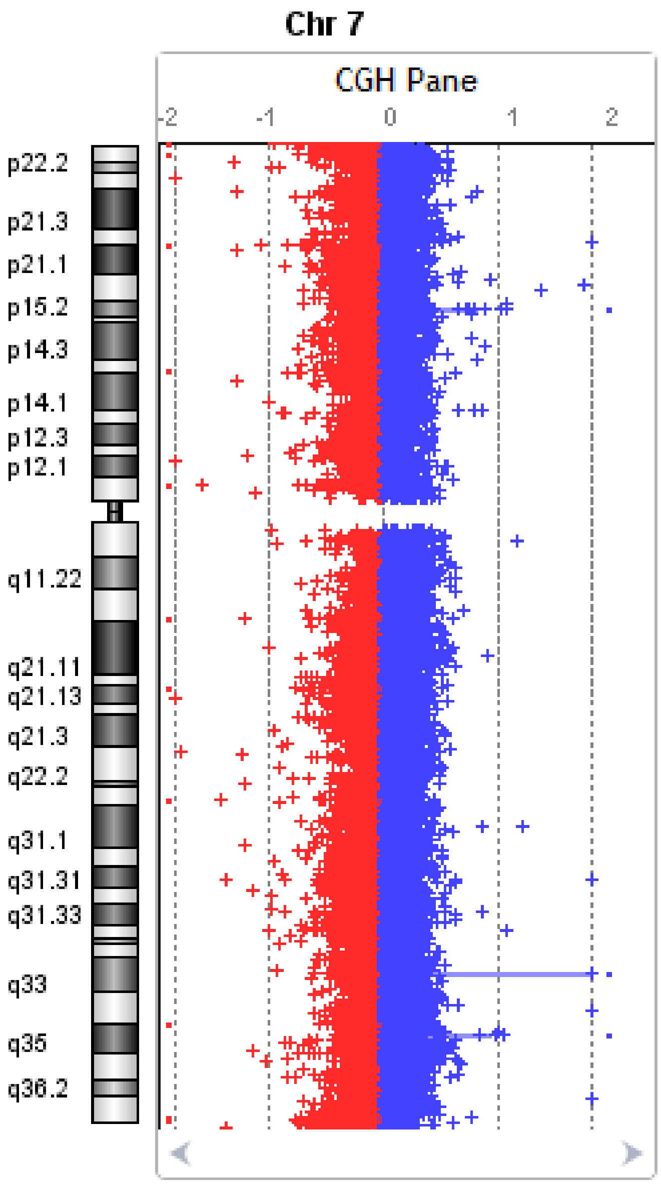

This is an intermediate report and not a final signed off report

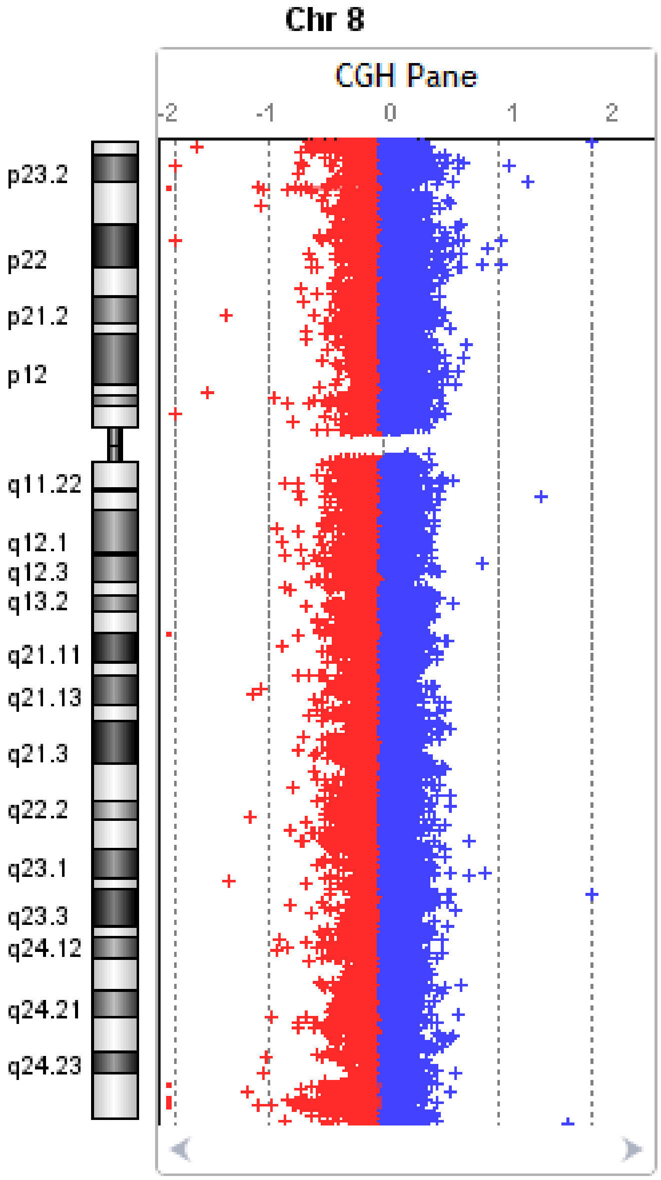

This is an intermediate report and not a final signed off report

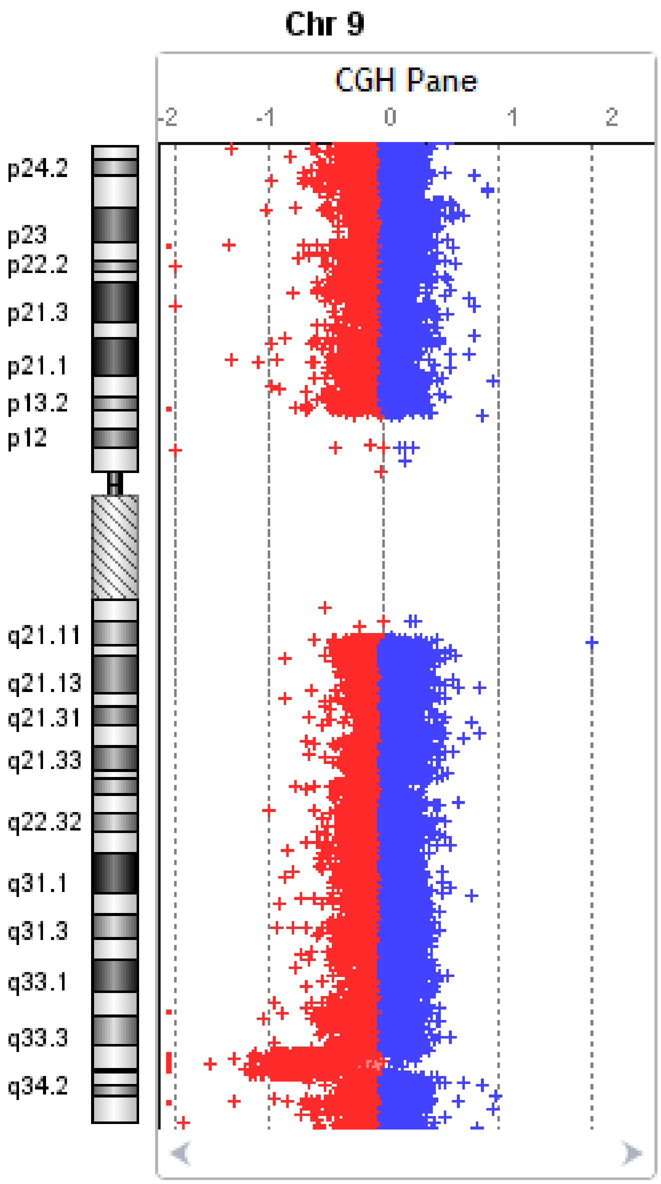

This is an intermediate report and not a final signed off report

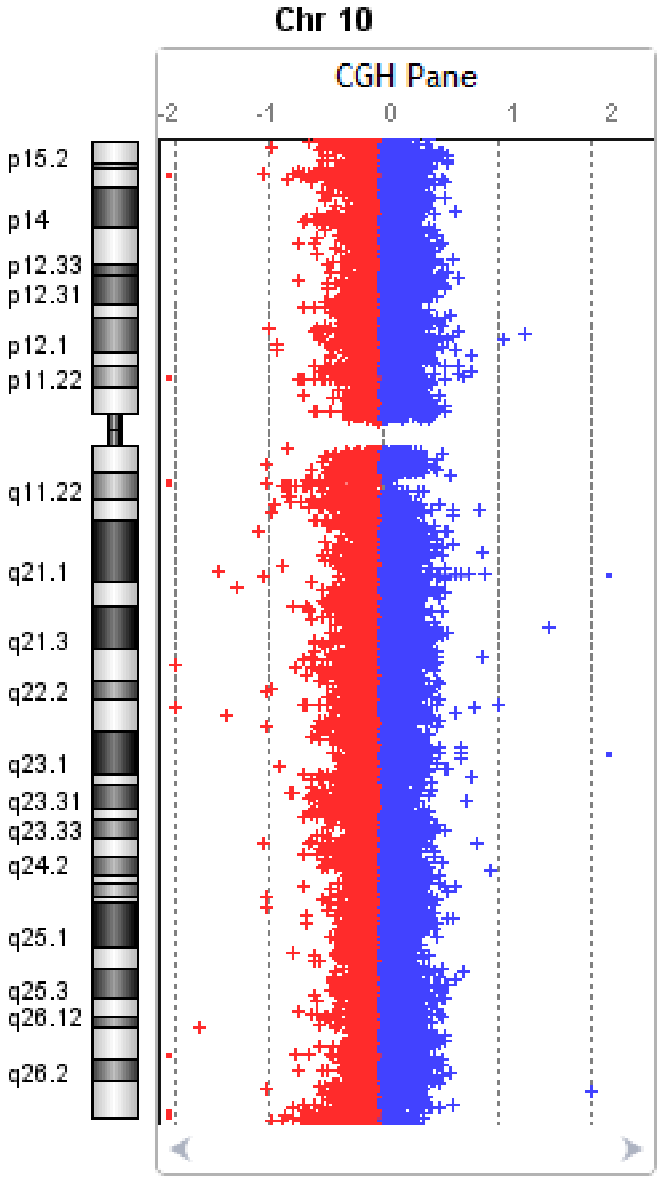

This is an intermediate report and not a final signed off report

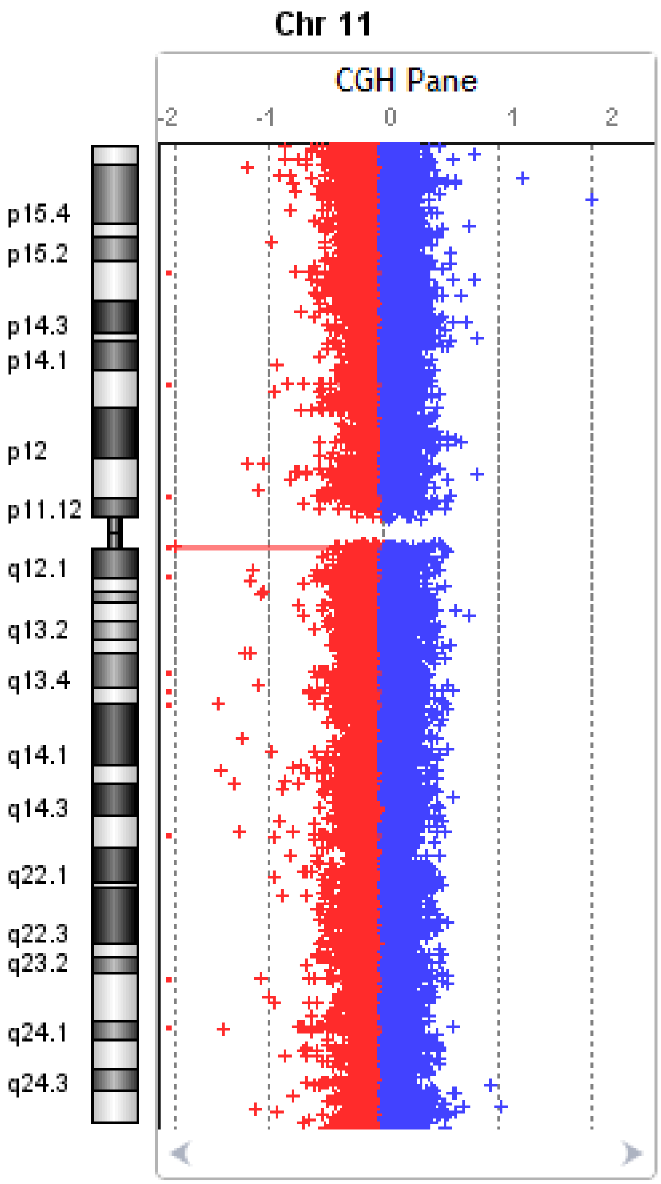

This is an intermediate report and not a final signed off report

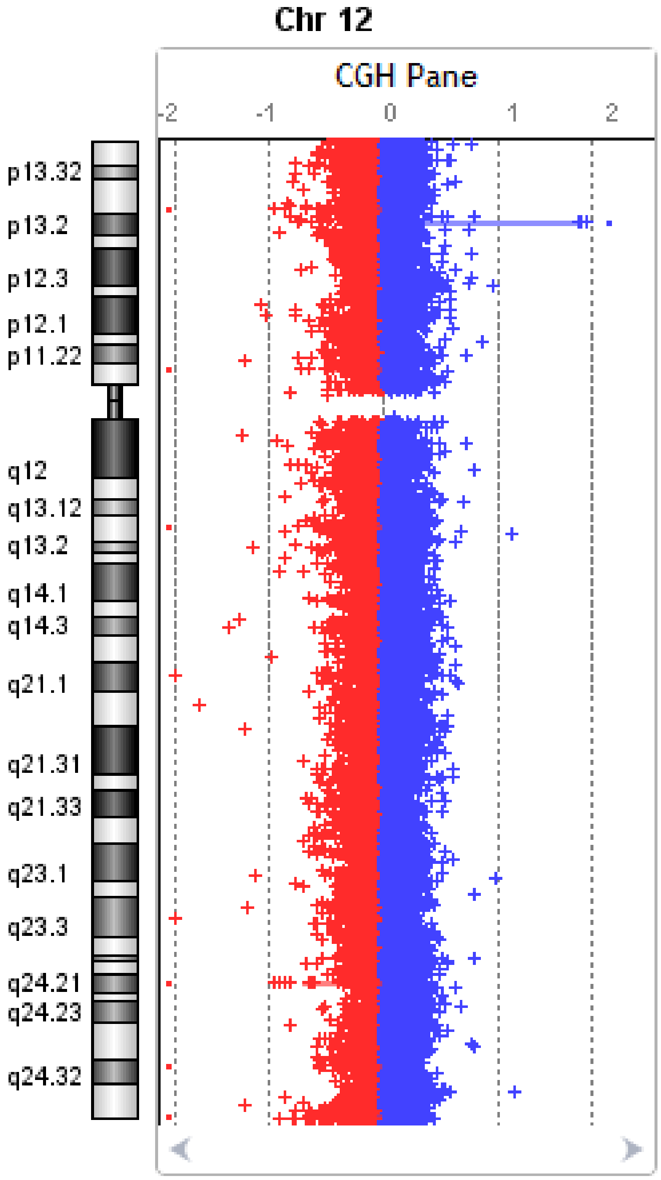

This is an intermediate report and not a final signed off report

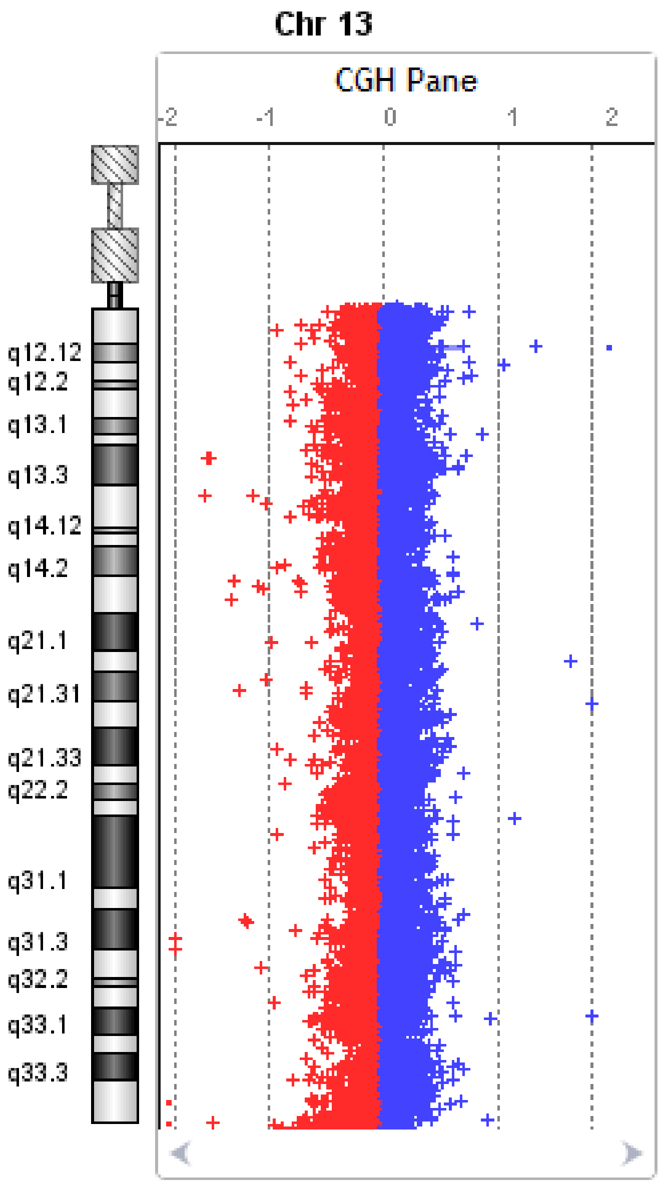

This is an intermediate report and not a final signed off report

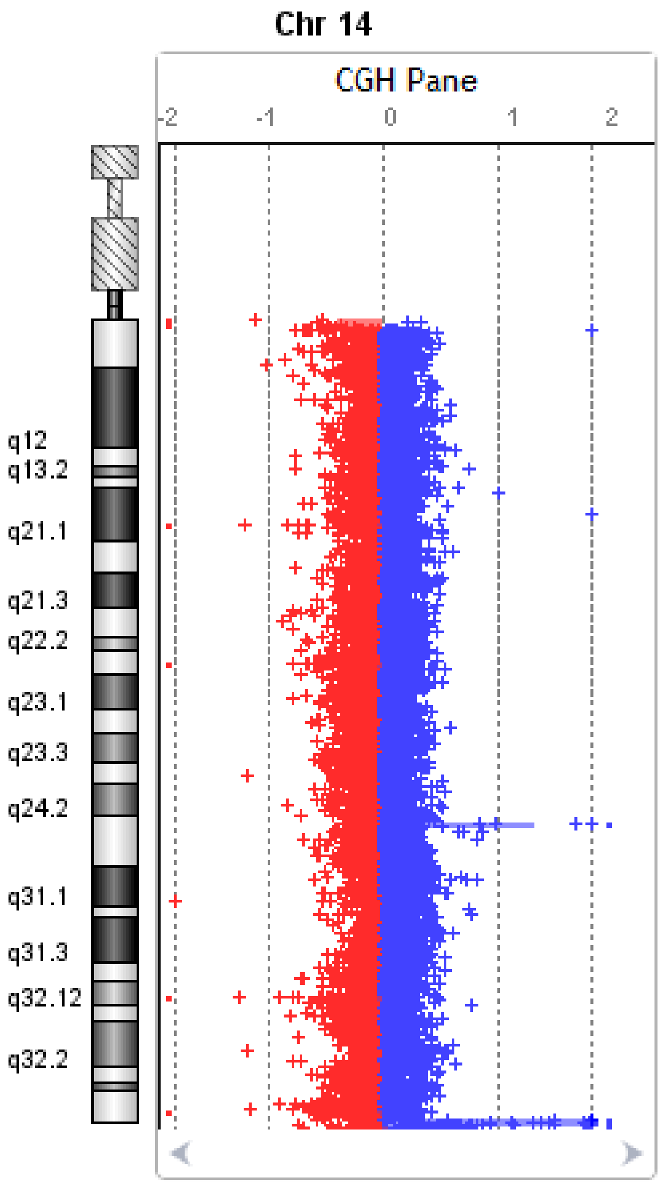

This is an intermediate report and not a final signed off report

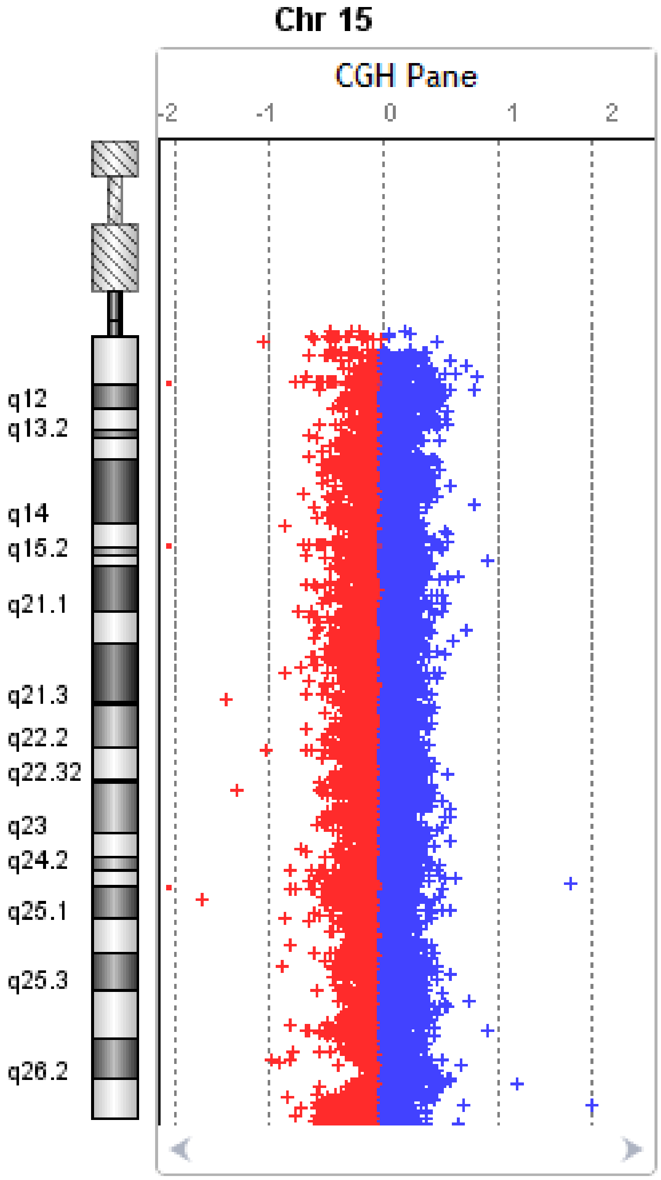

This is an intermediate report and not a final signed off report

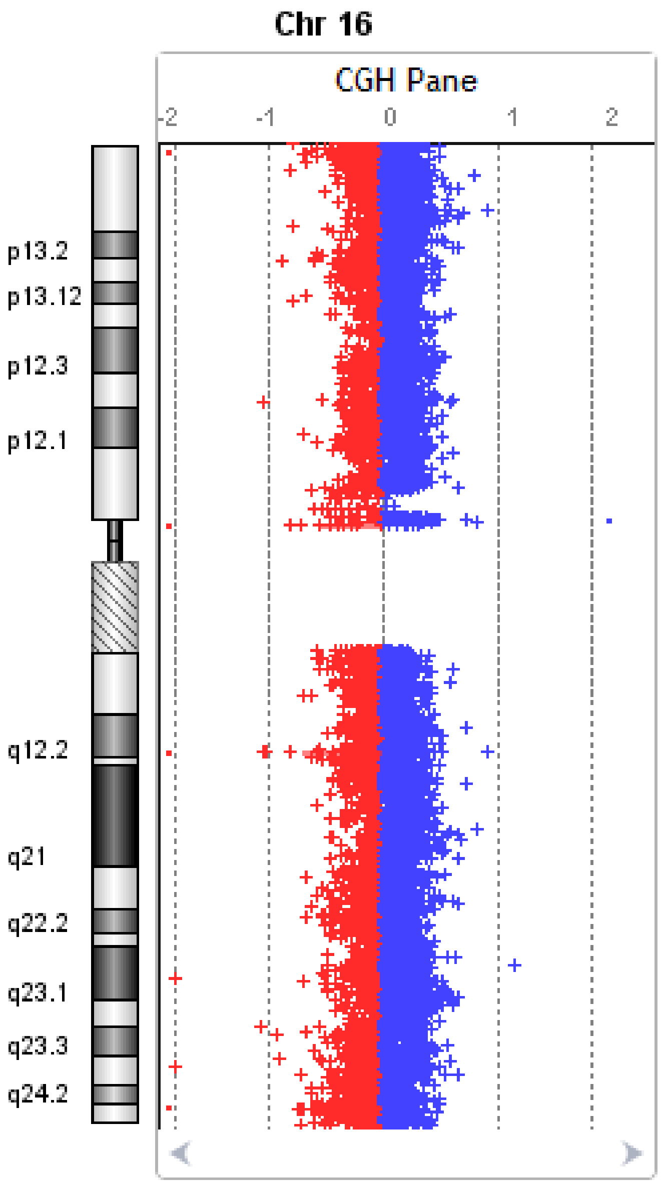

This is an intermediate report and not a final signed off report

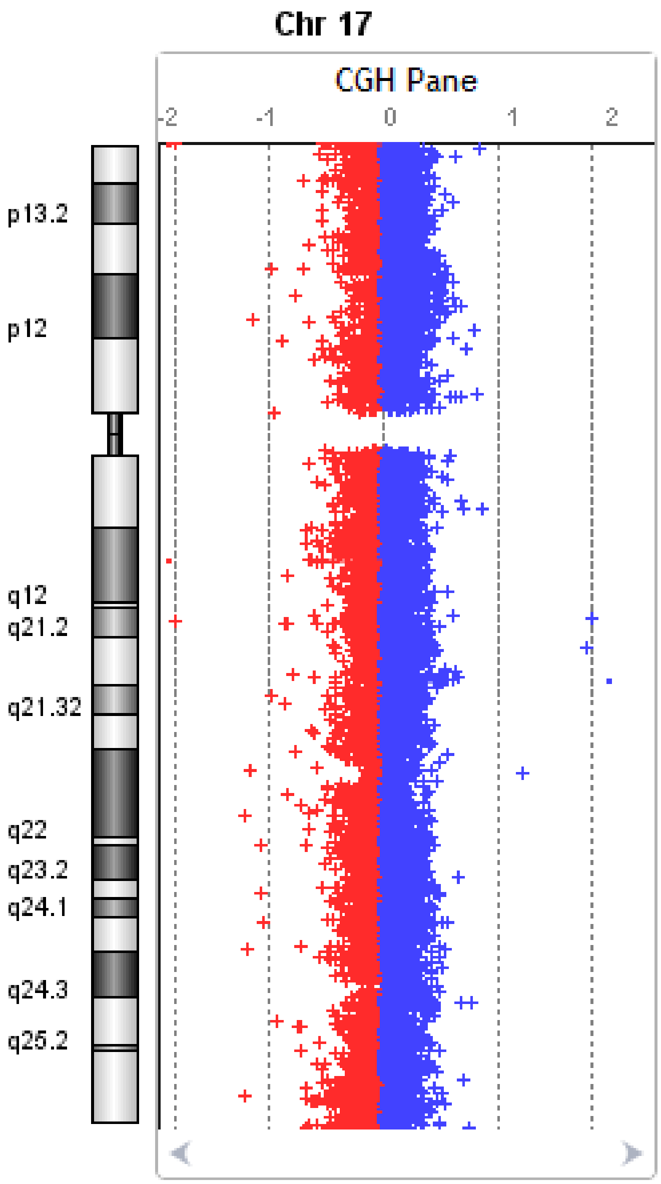

This is an intermediate report and not a final signed off report

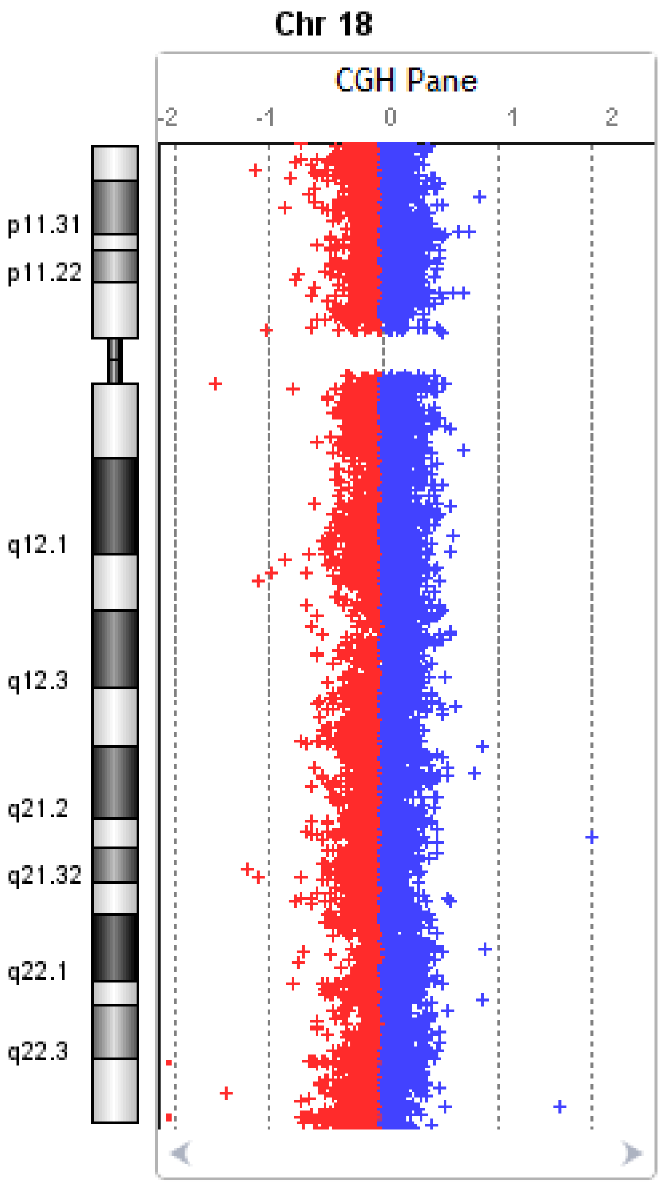

This is an intermediate report and not a final signed off report

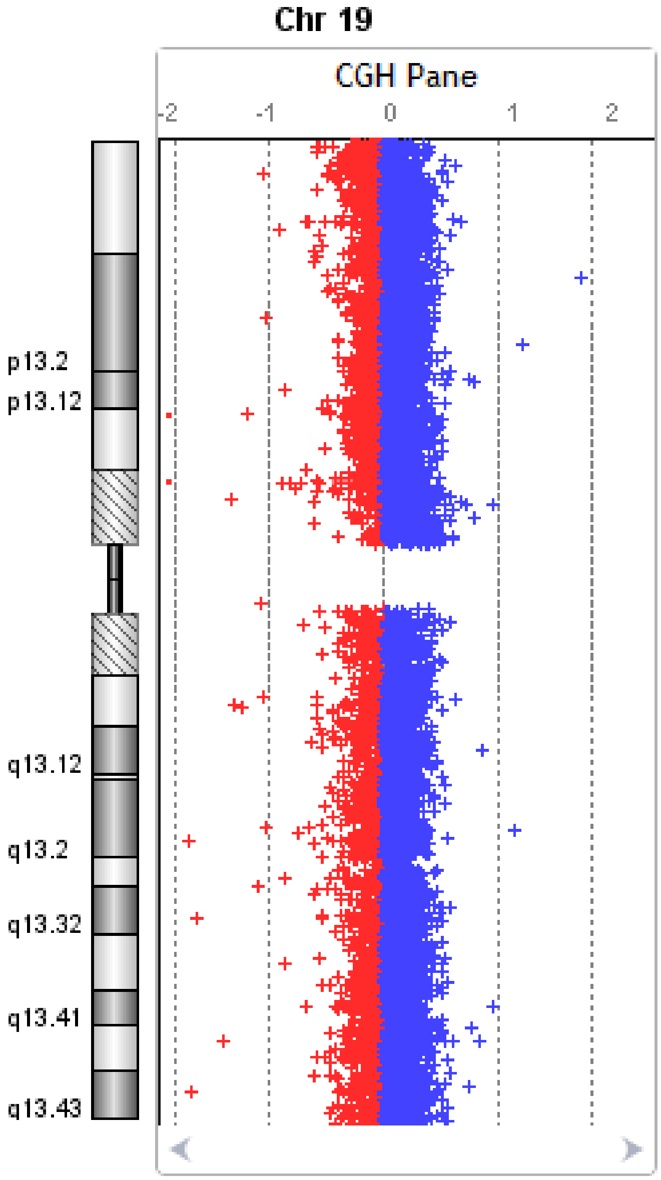

This is an intermediate report and not a final signed off report

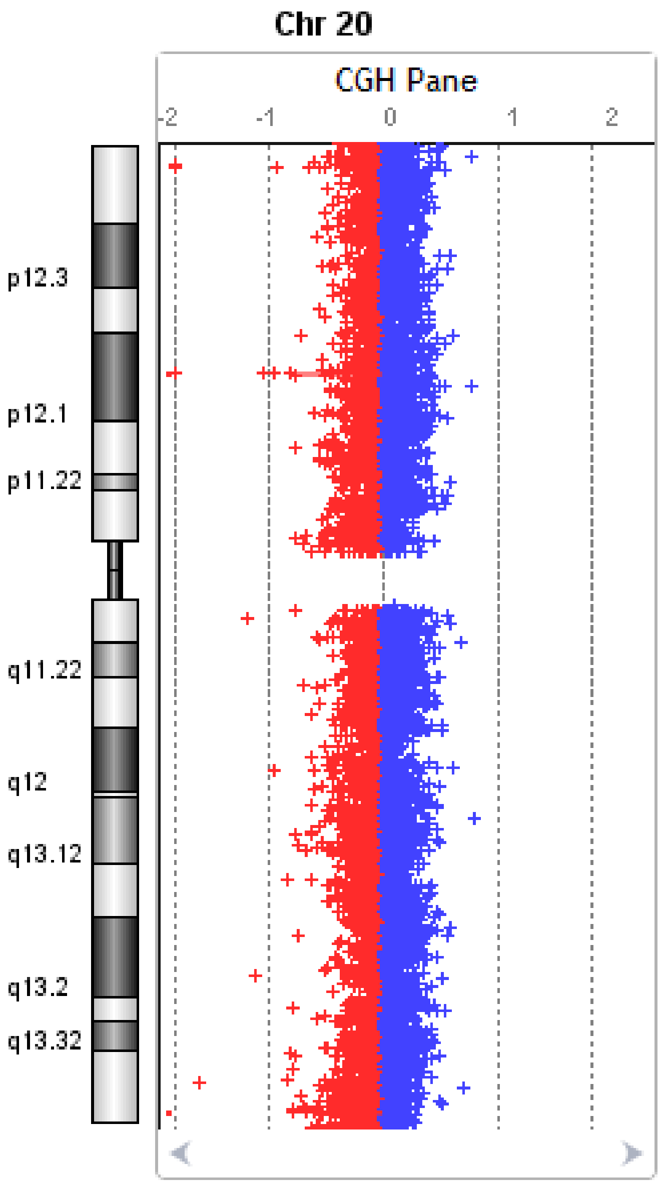

This is an intermediate report and not a final signed off report

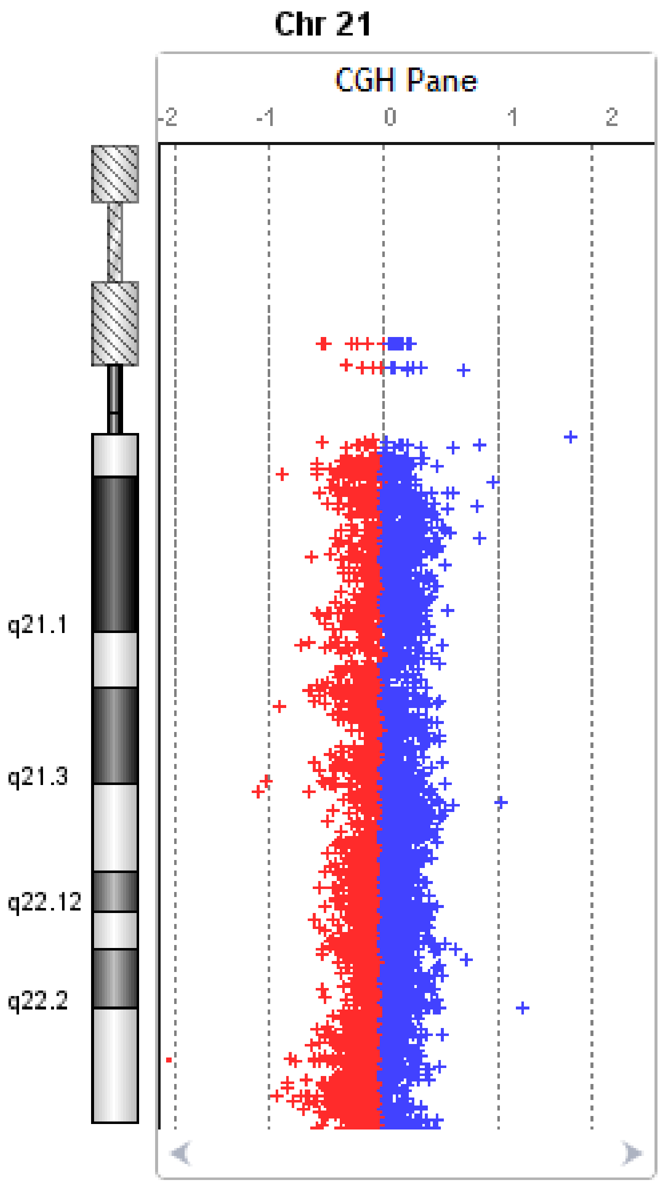

This is an intermediate report and not a final signed off report

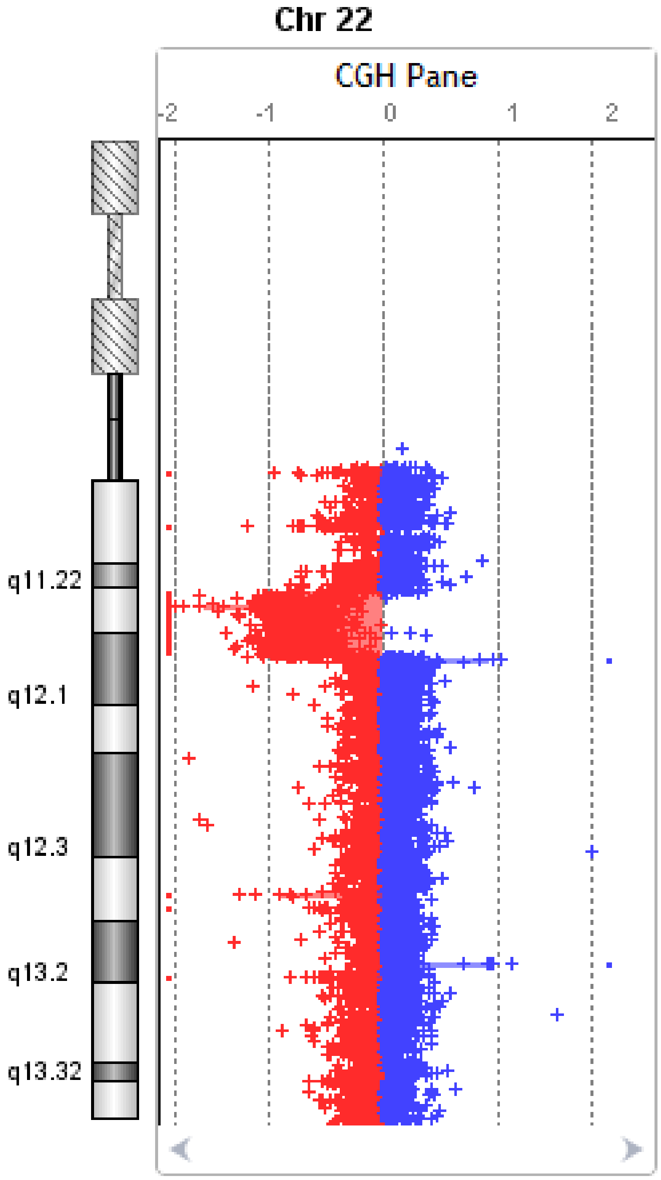

This is an intermediate report and not a final signed off report

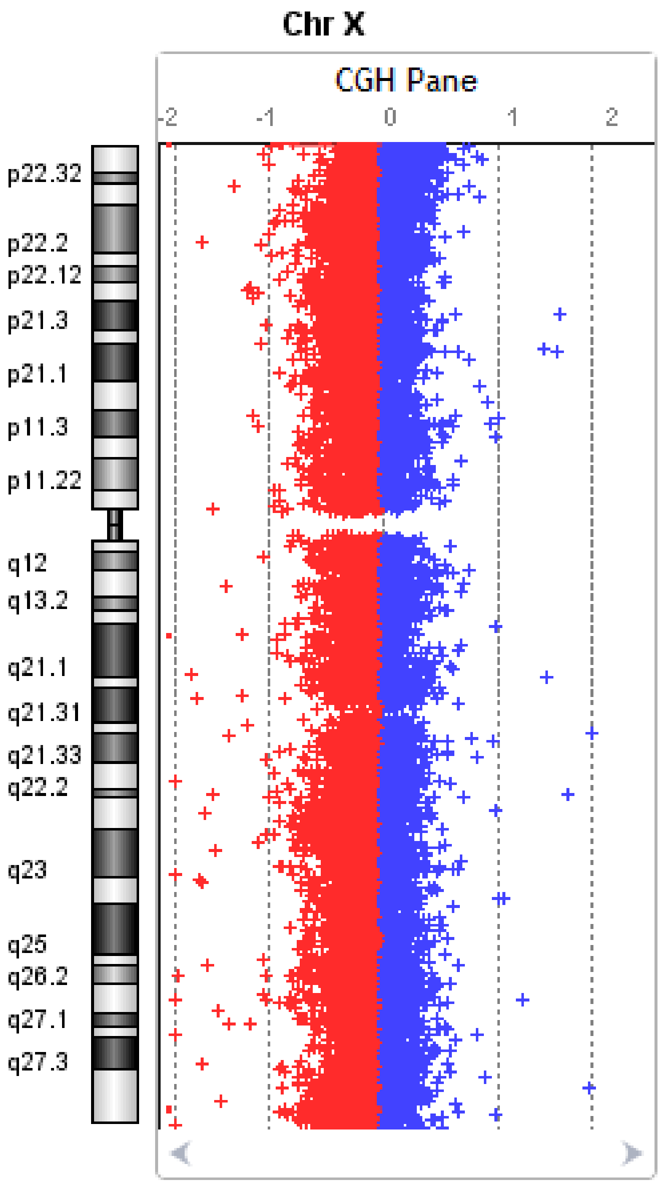

This is an intermediate report and not a final signed off report

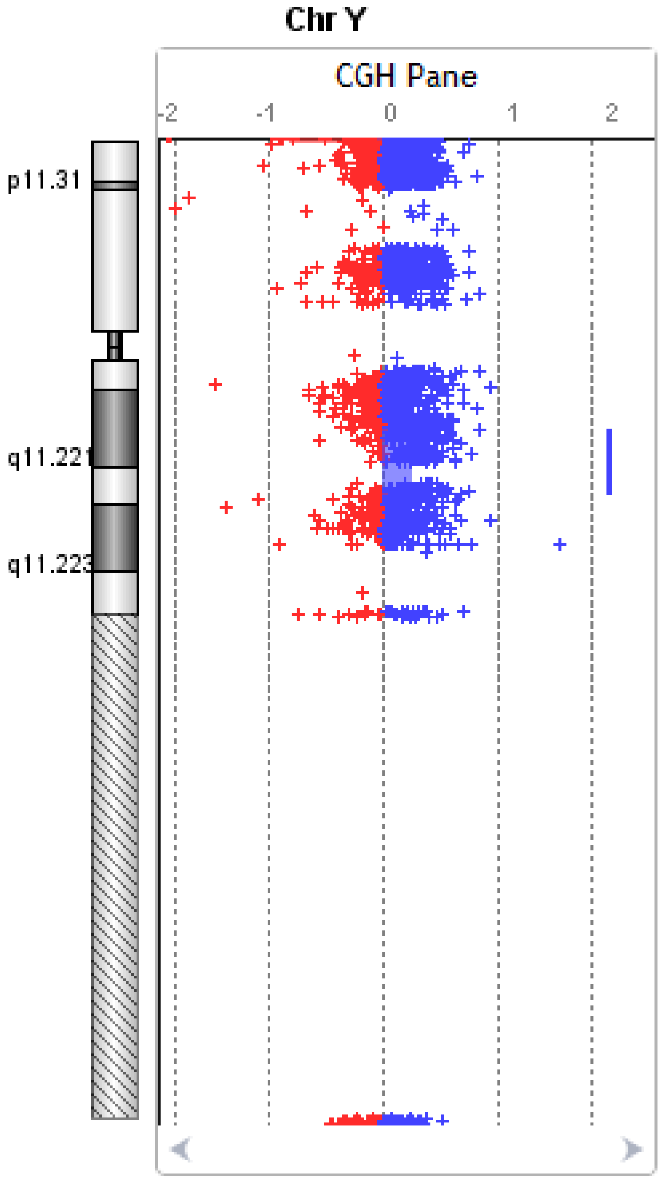

This is an intermediate report and not a final signed off report

Amp/Del Intervals Table

Chr	Start-Stop(bp)	Size(bp)	Cytoband	#Probes	Amp/Del	P-value	Annotations
chr1	12846934-12917461	70,528	p36.21	6	-0.731167	1.70E-16	PRAMEF1, PRAMEF11, LOC649330...
chr2	32632139-33332063	699,925	p22.3	150	0.491710	NA	BIRC6, MIR558, TTC27...
chr7	129757-229852	100,096	p22.3	21	-0.597667	1.43E-20	FAM20C, nssv579569_unk, nssv582028_unk...
chr8	143501226-143586335	85,110	q24.3	17	-0.688473	2.67E-15	BAI1, nssv582242_unk, nssv583478_unk_F...
chr9	130211514-133580084	3,368,571	q33.3 - q34.12	615	-0.648459	NA	RPL12, LRSAM1, FAM129B...
chr14	92858456-92936822	78,367	q32.12	22	-0.529464	1.26E-31	SLC24A4, nssv578675_unk, nssv706219_unk...
chr20	61919662-61995842	76,181	q13.33	15	-0.531264	5.61E-14	ARFGAP1, COL20A1, CHRNA4
chr22	23634972-26980416	3,345,445	q11.23 - q12.1	700	-0.702461	NA	BCR, CES5AP1, ZDHHC8P1...

Amp=Amplification
Del=Deletion

Total Amp/Del Intervals: 8

This is an intermediate report and not a final signed off report

Analysis Settings

Design	:	021850_20111015	Sample Name	:	12-0350D-252185022565_1_1
Genome	:	hg19	Aberration Algorithm	:	ADM-2
Threshold	:	6.0	Fuzzy Zero	:	OFF
GC Correction	:	ON	Window Size	:	2Kb
Centralization (legacy)	:	OFF	Diploid Peak Centralization	:	ON
SNP Copy Number	:	OFF	LOH	:	OFF
Combine Replicates (Intra Array)	:	ON	Array Level Filter	:	NONE
Metric Set Filter	:	NONE	Aberration Filter	:	Minimum Number of Probes for Amplification >= 3 AND Nesting Level <= 100 AND Minimum Avg. Absolute Log Ratio for Amplification >= 0.25 AND Minimum Size (Kb) of Region for Amplification >= 0.0 AND Minimum Size (Kb) of Region for Deletion >= 0.0 AND Minimum Number of Probes for Deletion >= 3 AND Minimum Avg. Absolute Log Ratio for Deletion >= 0.25
Feature Level Filter	:	glsSaturated = true OR rIsSaturated = true OR glsFeatNonUnifOL = true OR rIsFeatNonUnifOL = true OR LogRatio = 0; Include matching values=false	Design Level Filter	:	Homology = 0 OR IsPseudoautosomal = 1
LOH Filter	:	NONE	Genomic Boundary	:	OFF
Show Flat Intervals	:	false	Template Name	:	Default Cyto Report Template - CGH

This is an intermediate report and not a final signed off report

Notes

Sample Notes

No notes available.

Amp/Del Interval Notes

No notes available.

Classifications

Amp/Del Interval Classifications

Interval	Classification
chr1:12846934-12917461	benign
chr2:32632139-33332063	benign
chr2:32632139-33332063	unknown
chr7:129757-229852	benign
chr8:143501226-143586335	unknown
chr9:130211514-133580084	pathogenic
chr14:92858456-92936822	unknown
chr20:61919662-61995842	unknown
chr22:23634972-26980416	pathogenic

This is an intermediate report and not a final signed off report
