# Supplementary material for: Genomic Copy Number Variants in CML Patients With the Philadelphia Chromosome (Ph+): An Update
Source: Front Genet. 2021 Aug 10;12:697009. doi: 10.3389/fgene.2021.697009 (PMC8383316; doi:10.3389/fgene.2021.697009)
Supplement: Supplementary file 7 [file Data_Sheet_7.PDF]

Sample Information

Array ID	:	252185022557_1_1
Global Display Name	:	10-0206-FM-252185022557_1_1
Green Sample	:	
Red Sample	:	
Polarity	:	1
DerivativeOfLogRatioSD	:	0.182482
Intermediate Report by	:	OUHSC\xwang3

This is an intermediate report and not a final signed off report

Genome View (Amp/Del)

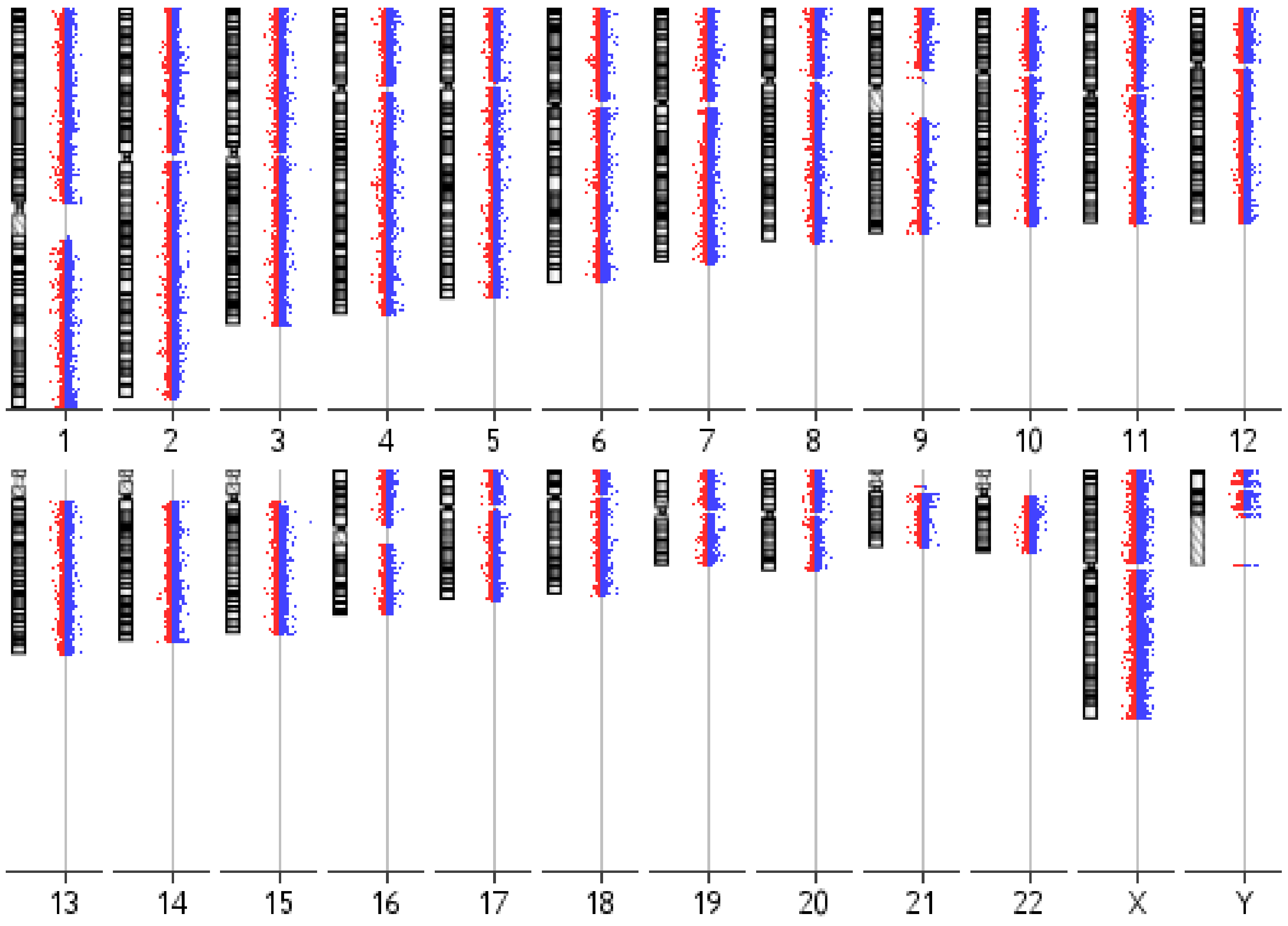

This is an intermediate report and not a final signed off report

Chromosome Views (Amp/Del)

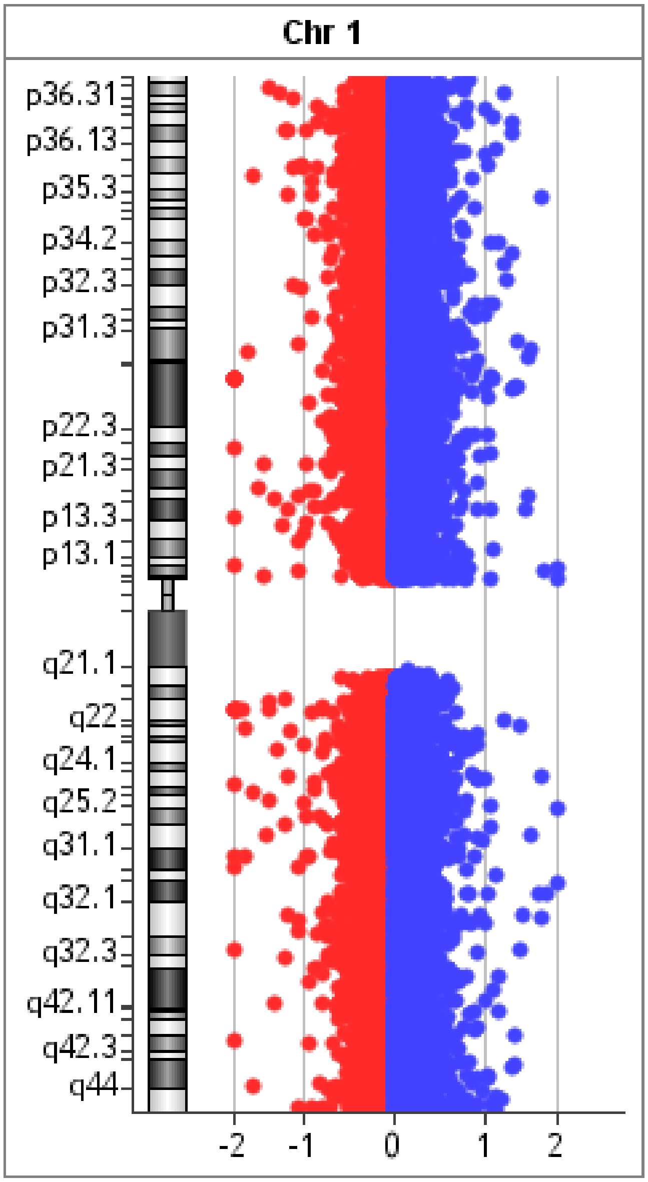

This is an intermediate report and not a final signed off report

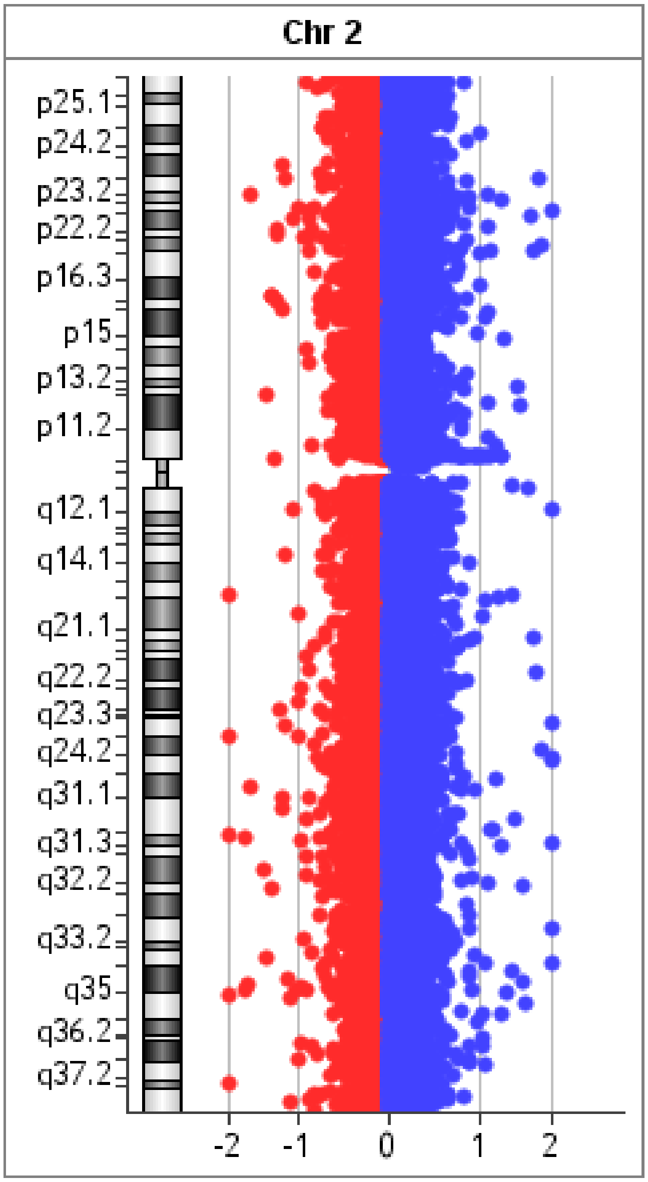

This is an intermediate report and not a final signed off report

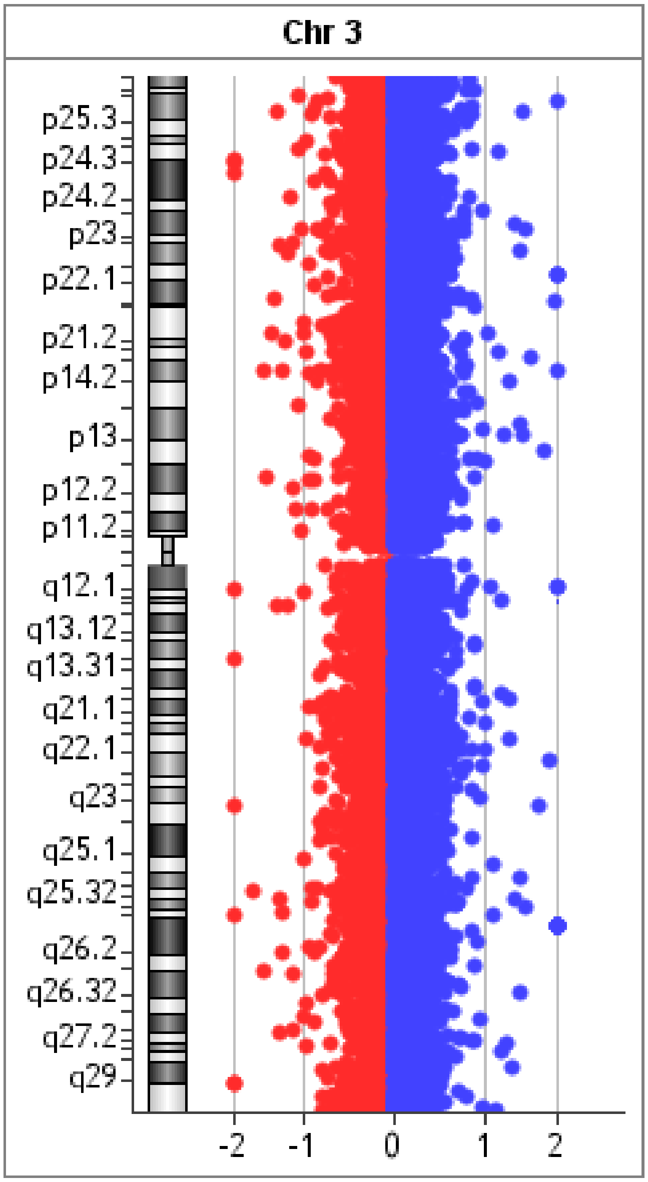

This is an intermediate report and not a final signed off report

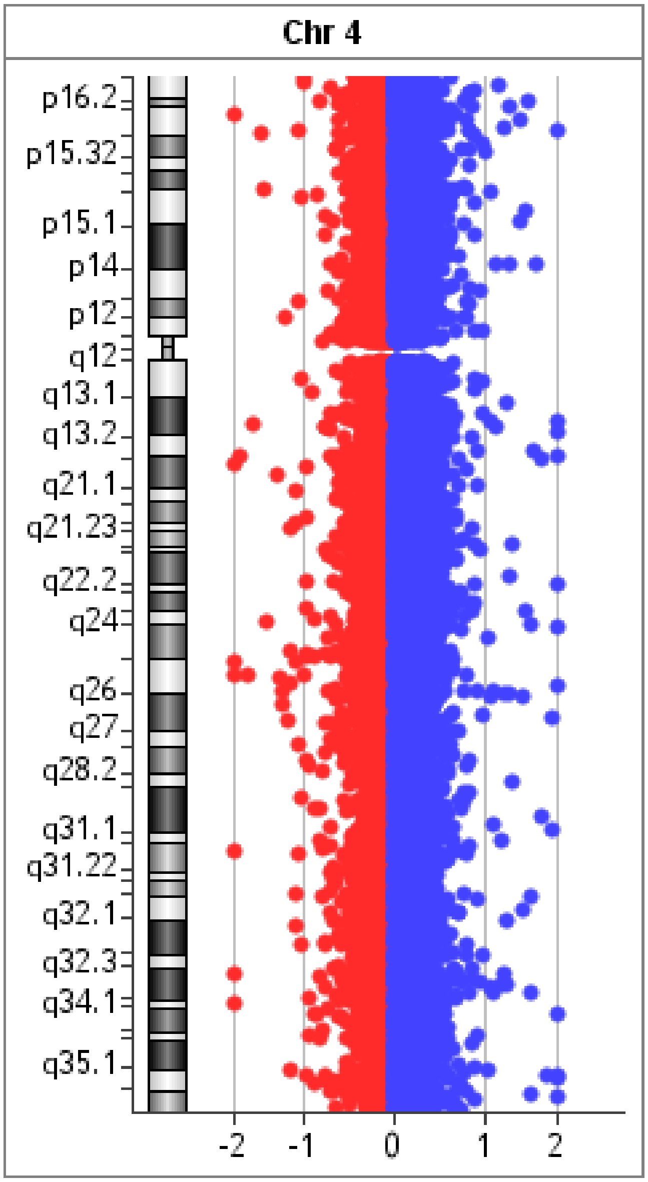

This is an intermediate report and not a final signed off report

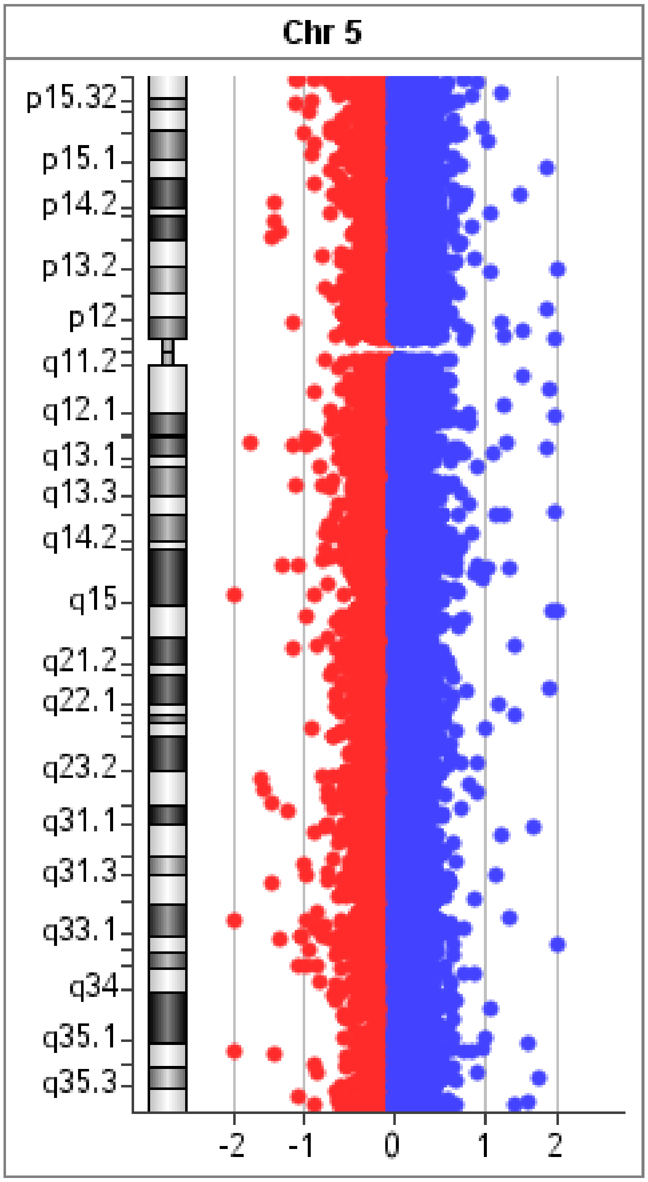

This is an intermediate report and not a final signed off report

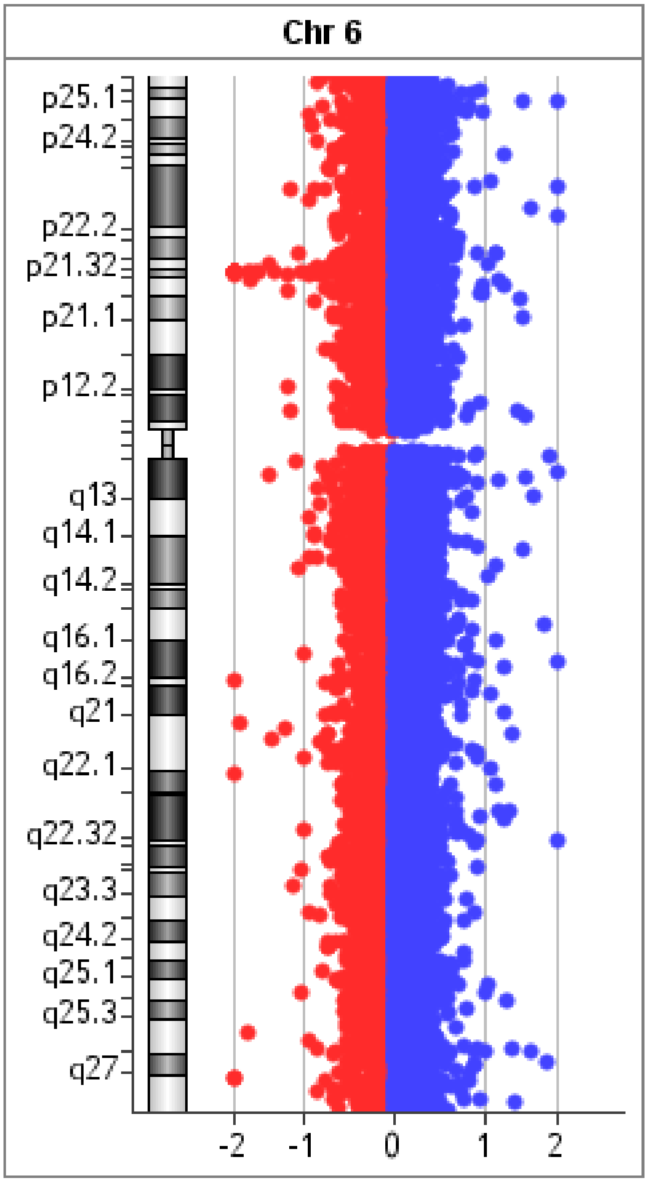

This is an intermediate report and not a final signed off report

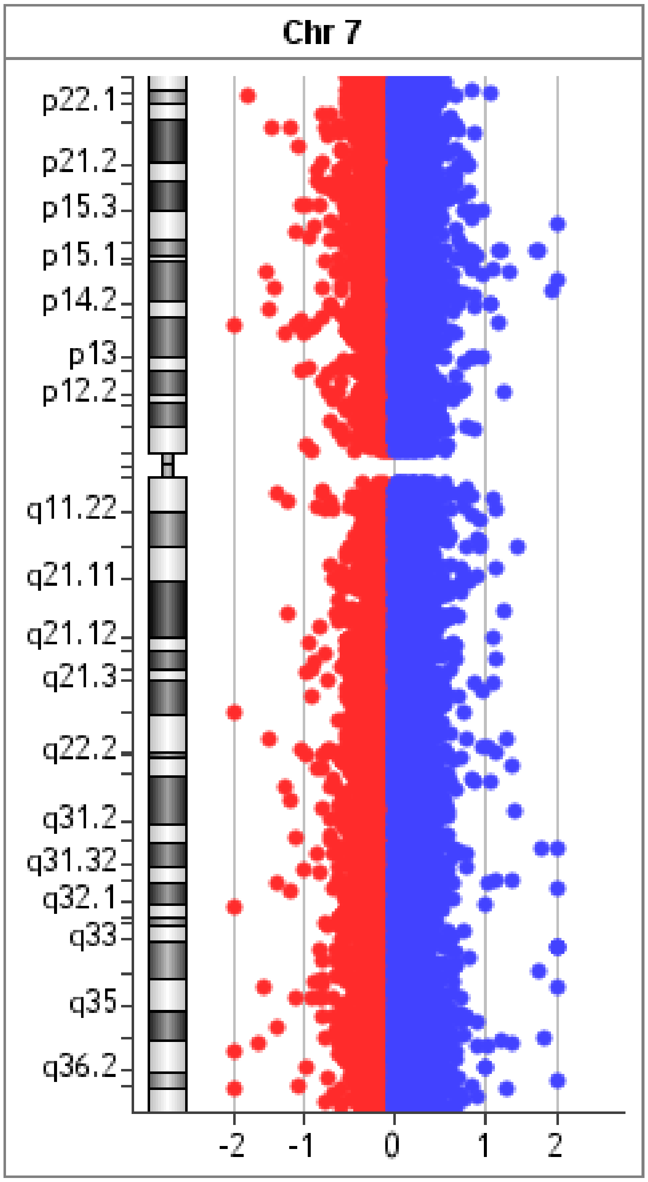

This is an intermediate report and not a final signed off report

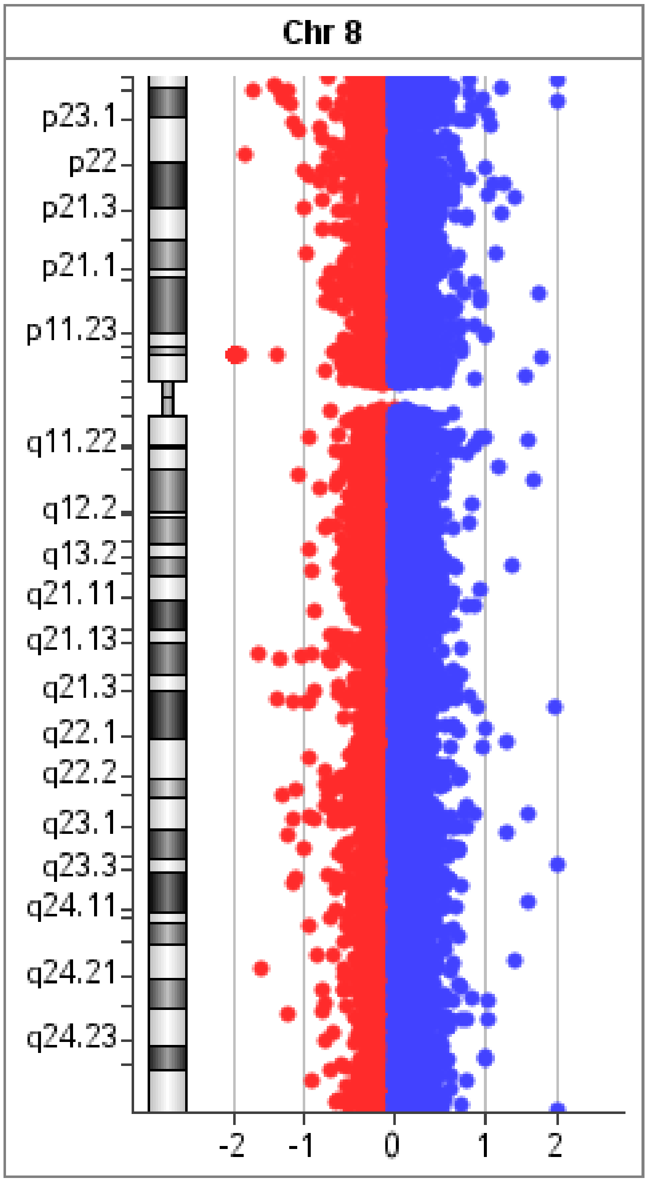

This is an intermediate report and not a final signed off report

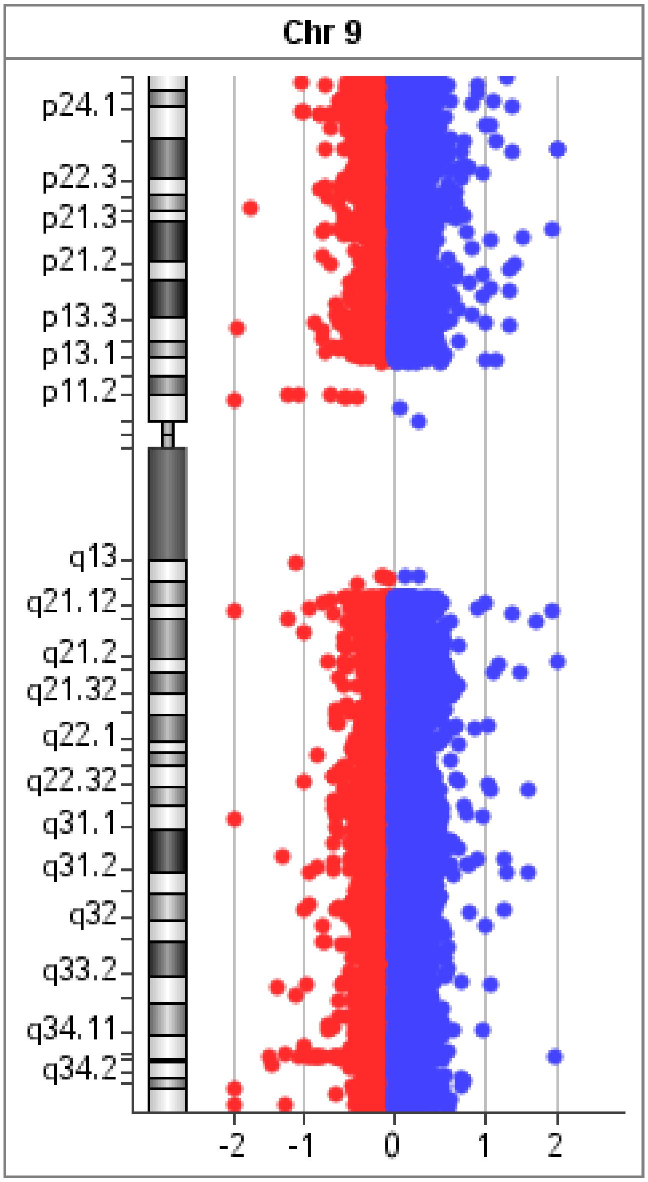

This is an intermediate report and not a final signed off report

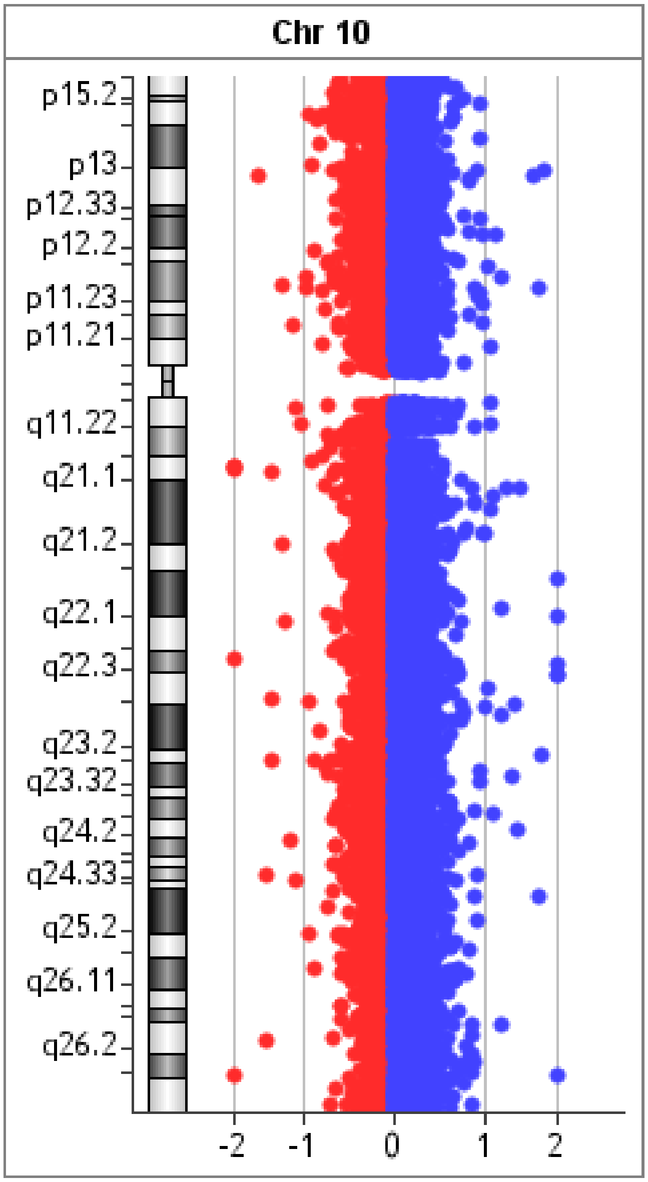

This is an intermediate report and not a final signed off report

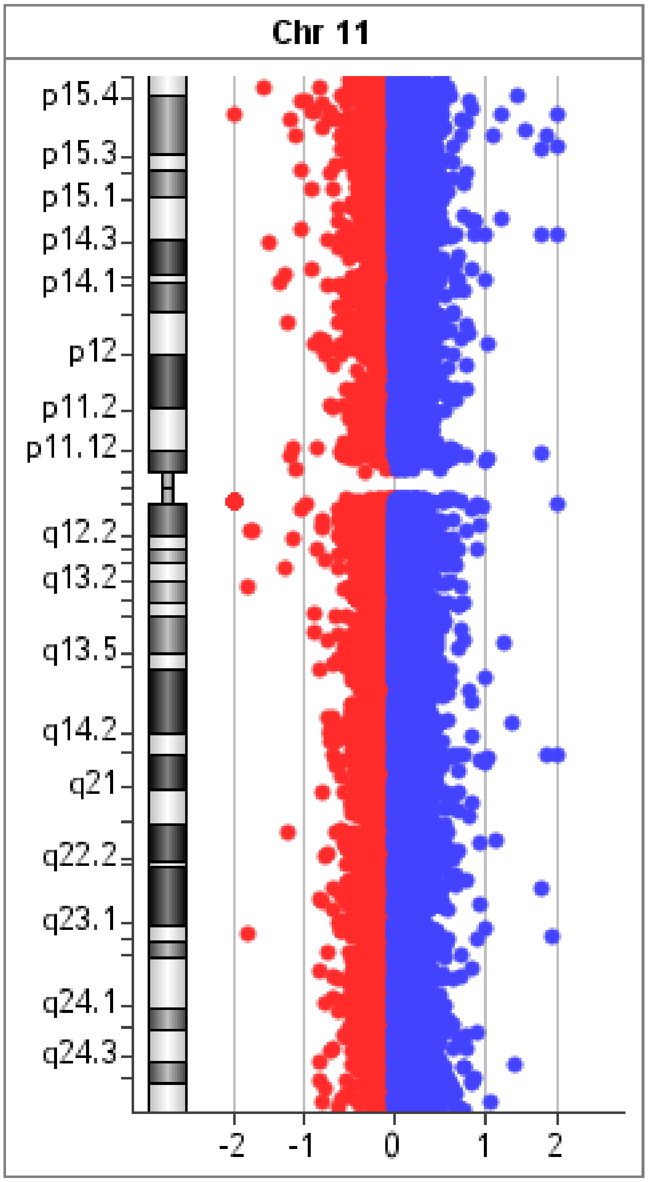

This is an intermediate report and not a final signed off report

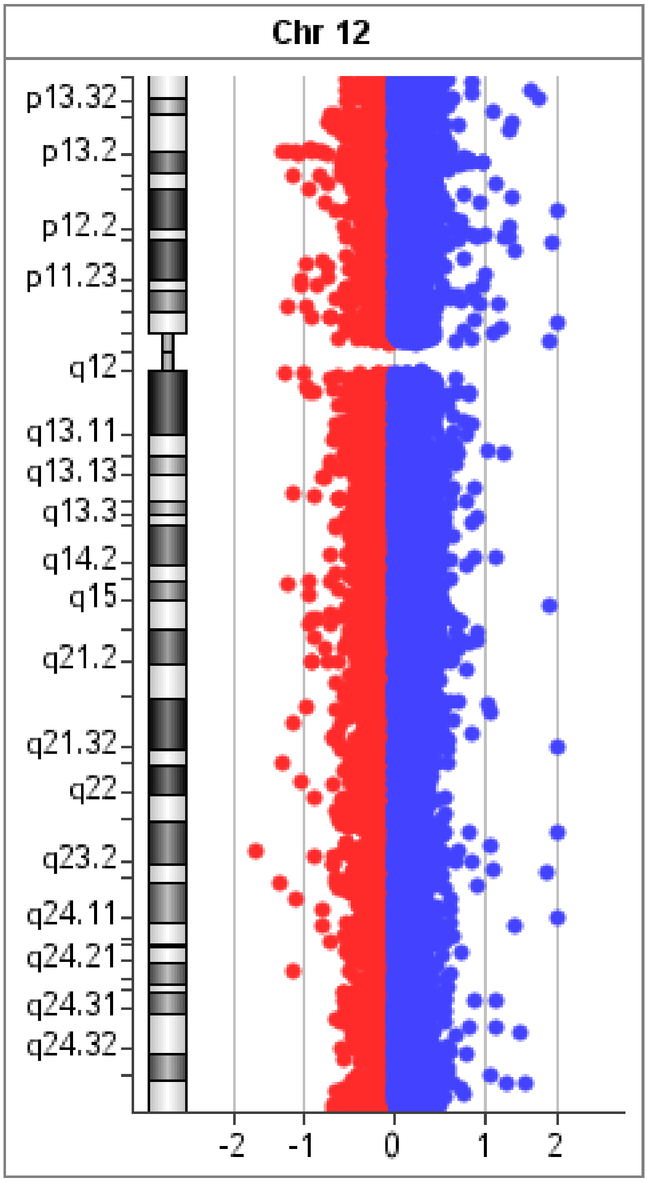

This is an intermediate report and not a final signed off report

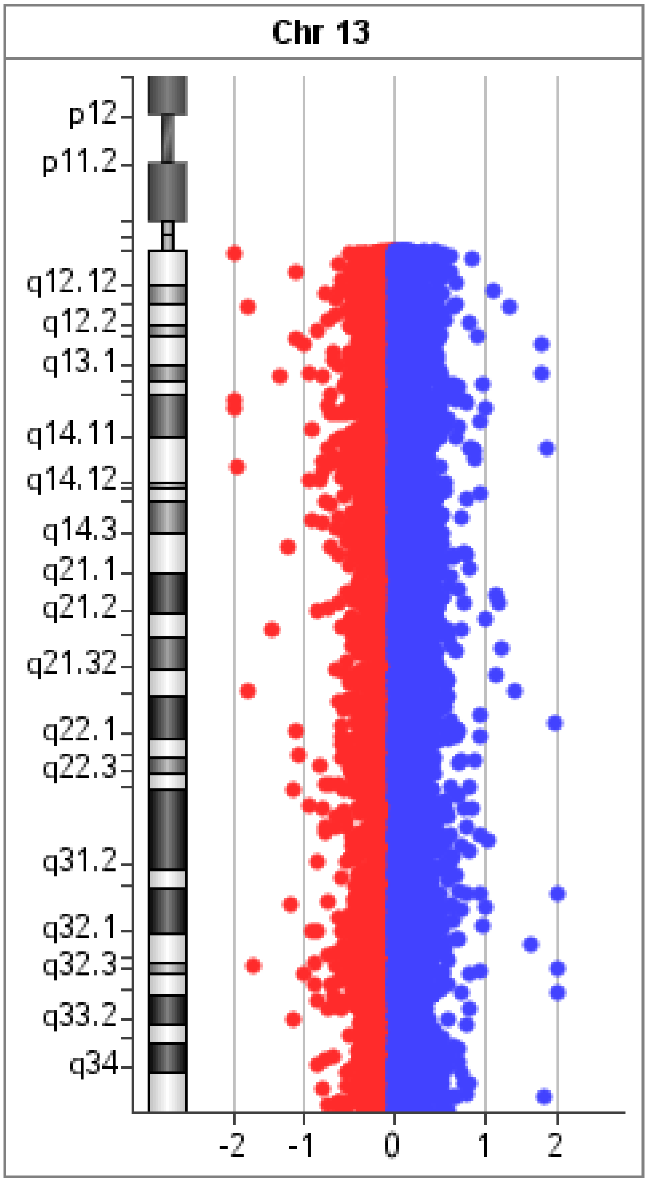

This is an intermediate report and not a final signed off report

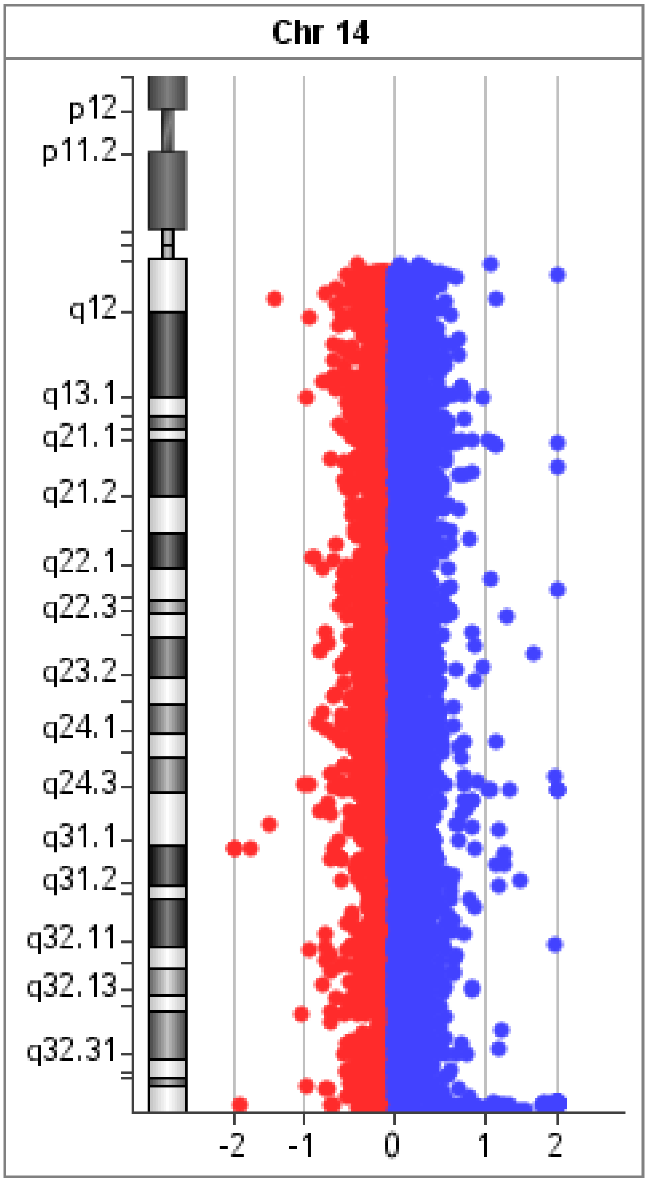

This is an intermediate report and not a final signed off report

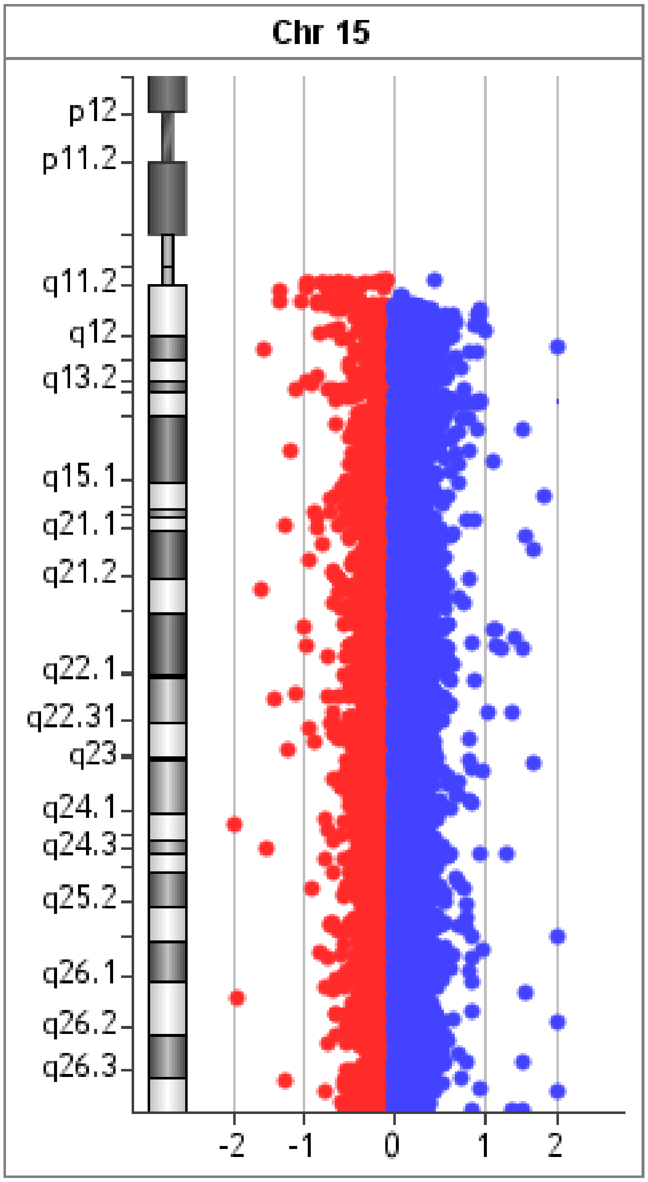

This is an intermediate report and not a final signed off report

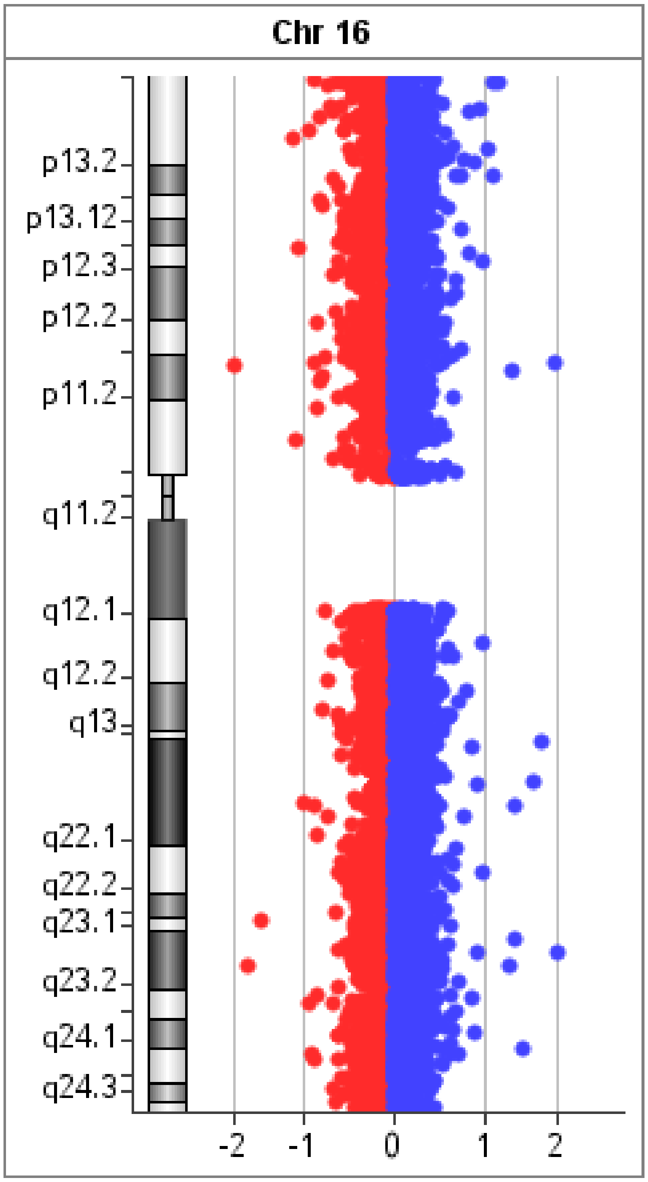

This is an intermediate report and not a final signed off report

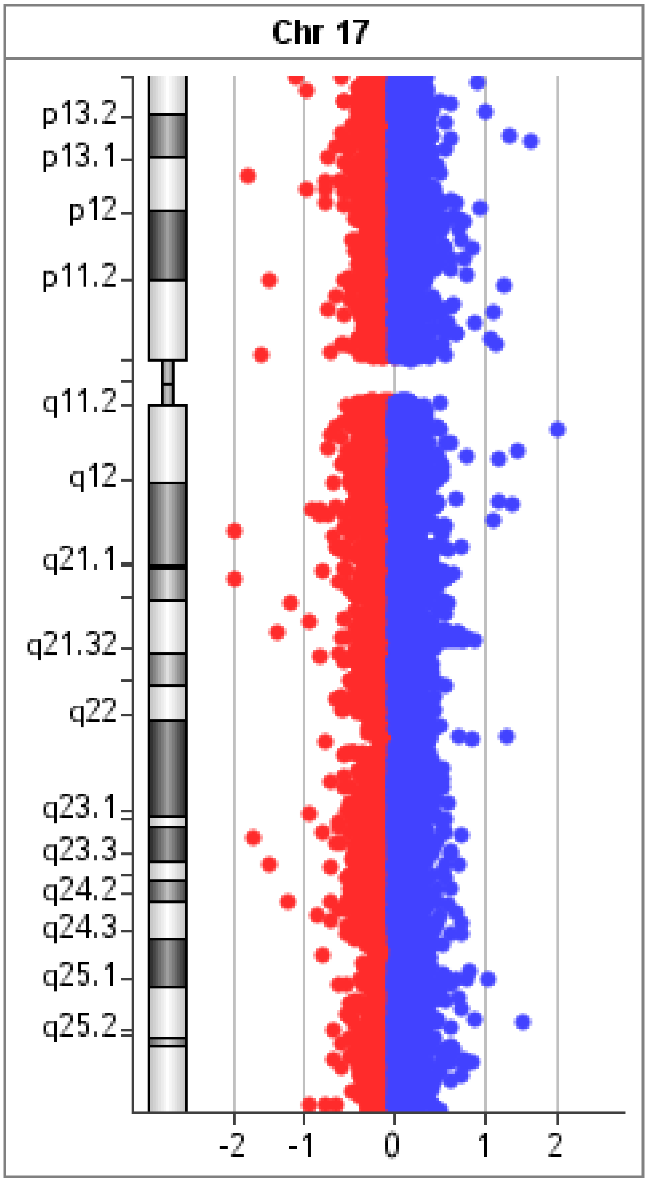

This is an intermediate report and not a final signed off report

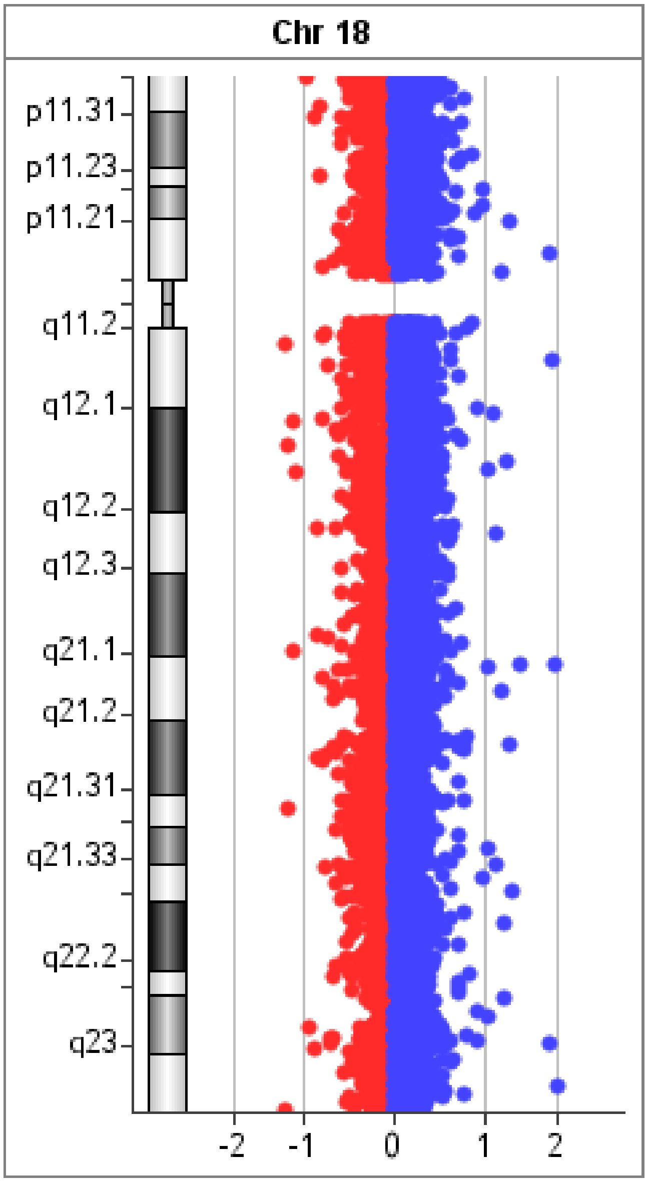

This is an intermediate report and not a final signed off report

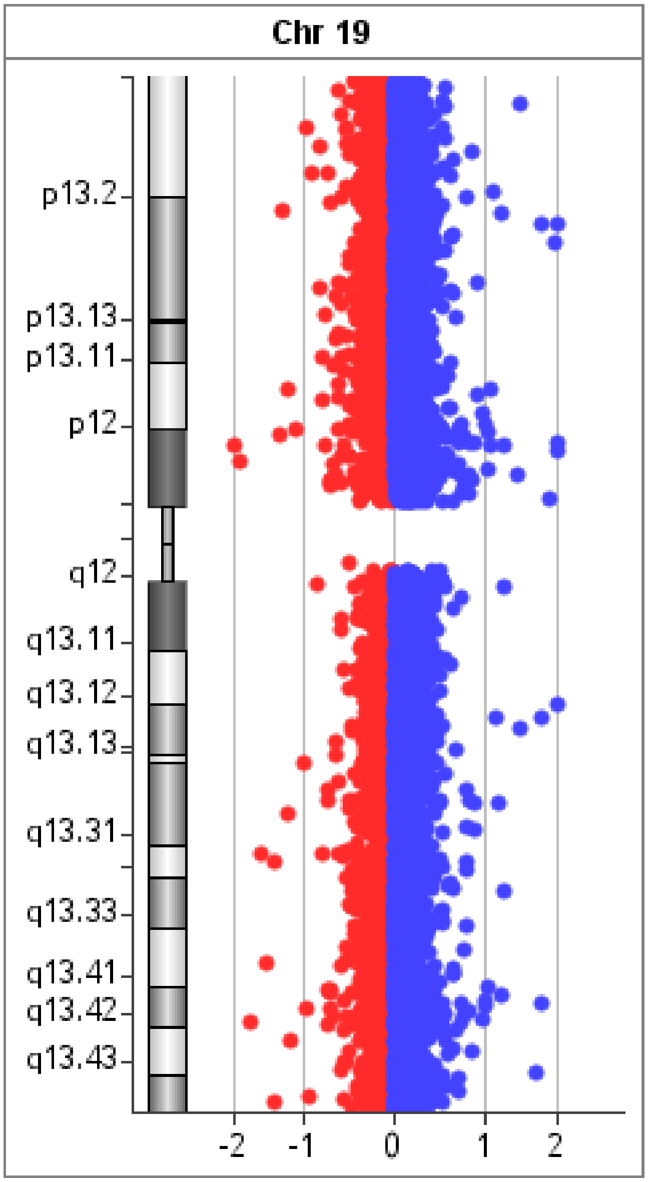

This is an intermediate report and not a final signed off report

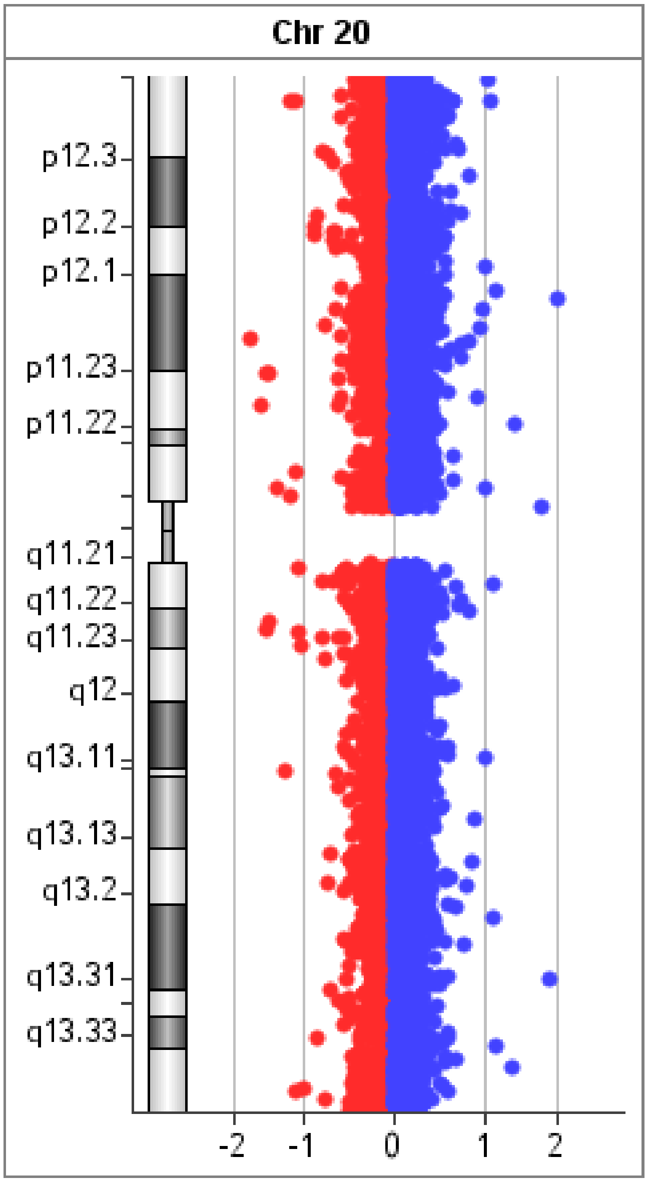

This is an intermediate report and not a final signed off report

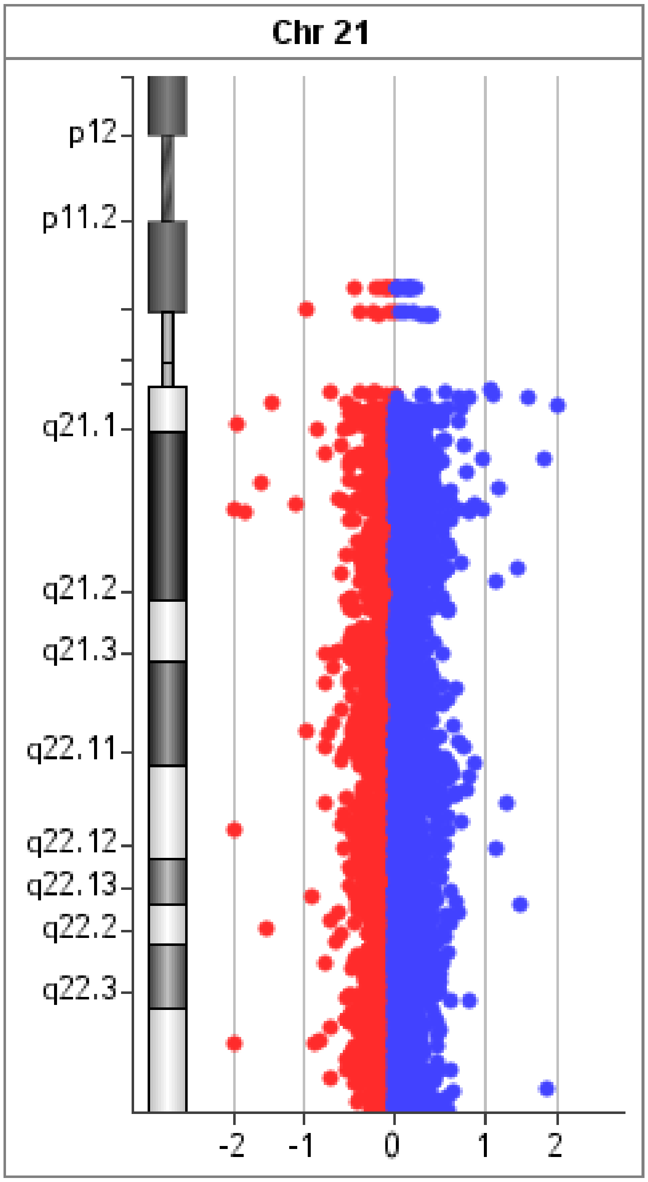

This is an intermediate report and not a final signed off report

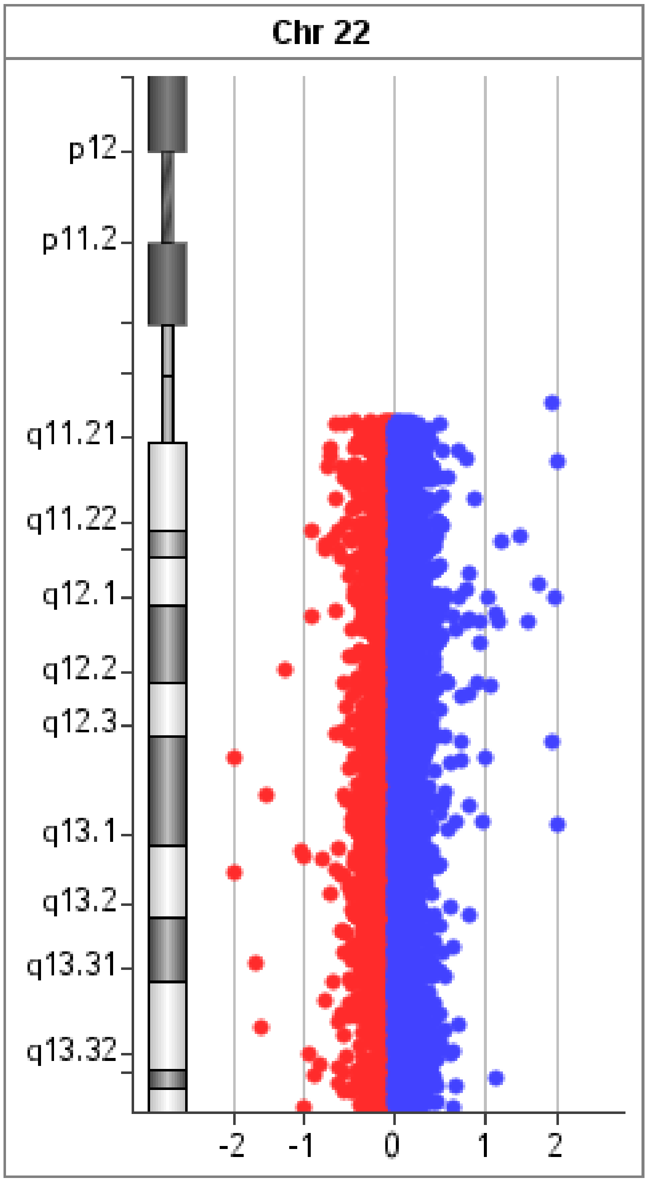

This is an intermediate report and not a final signed off report

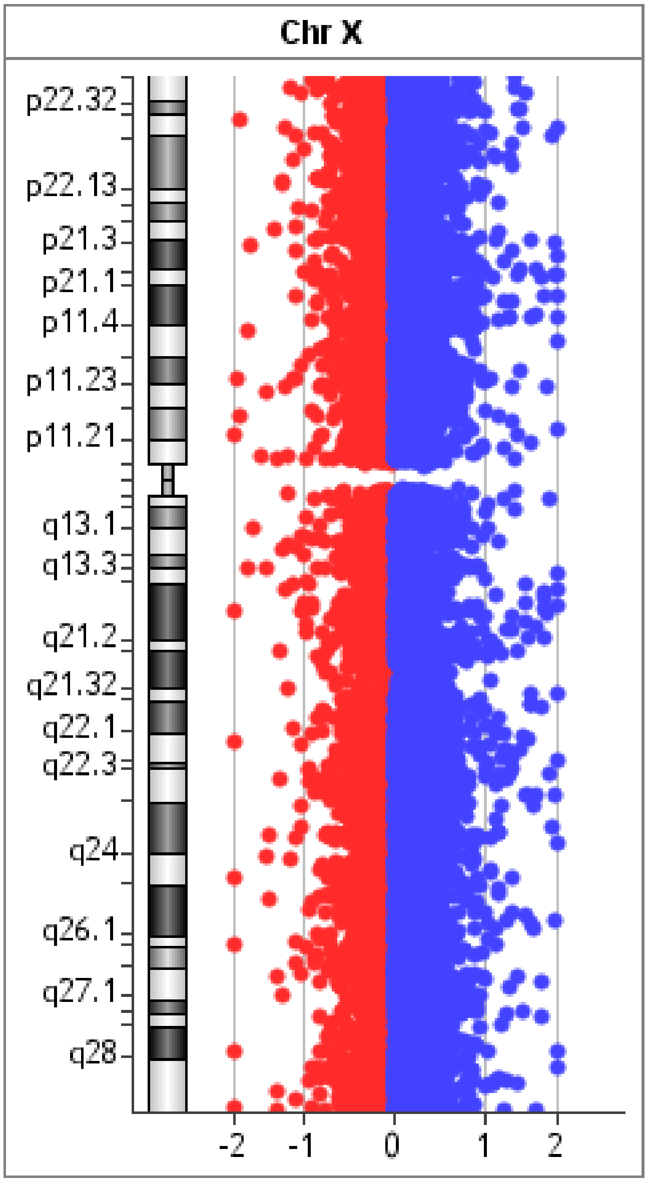

This is an intermediate report and not a final signed off report

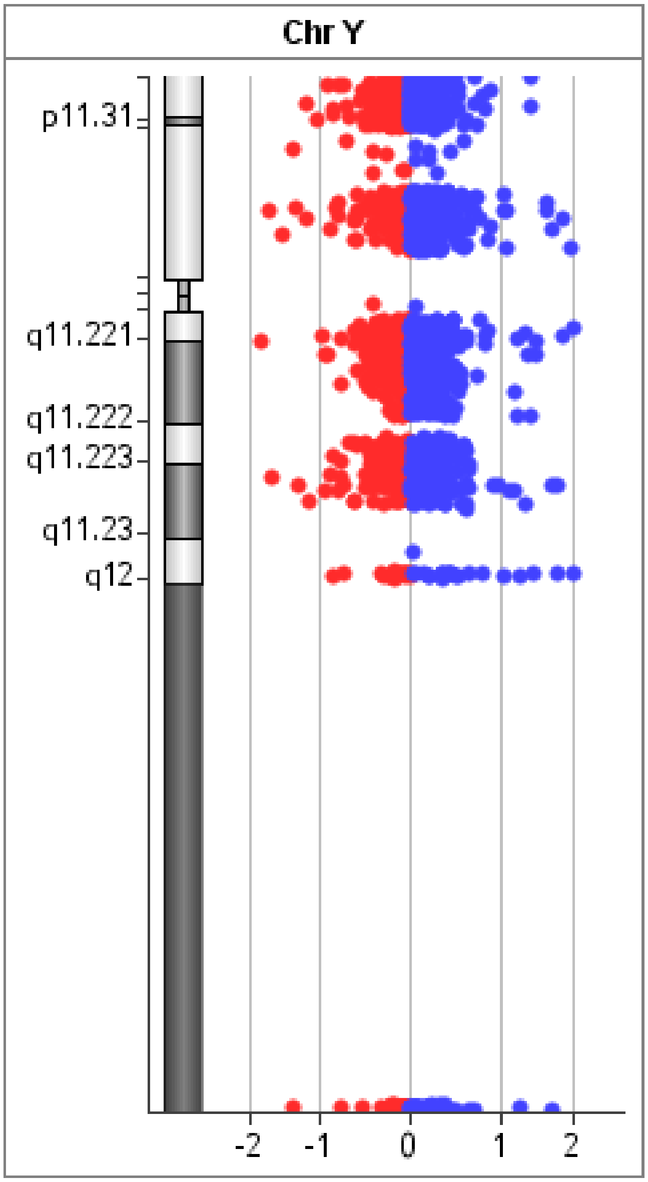

This is an intermediate report and not a final signed off report

Amp/Gain/Loss/Del Intervals Table

Chr	Start-Stop(bp)	Cytoband	Size(kb)	#Probes	Amp/Gain/ Loss/Del	Annotations	Classifications
chr3	100335969-100445465	q12.2	109.497	21	0.513	ADGRG7, TFG, nssv585039_unk...	
chr9	133576051-133681919	q34.12	105.869	21	-0.709	EXOSC2, ABL1, nsv831735...	
chr15	30652489-31004749	q13.2	352.261	7	-0.742	CHRFAM7A, GOLGA8R, ULK4P1...	
chr15	31985493-32510863	q13.3	525.371	68	0.540	CHRNA7, nssv585007_unk, esv33337...	

Amp=Amplification Del=Deletion

Total Amp/Gain/Loss/Del Intervals: 4

This is an intermediate report and not a final signed off report

ISCN Nomenclature

arr[GRCh37] 3q12.2(100335969_100445465)x3
arr[GRCh37] 9q34.12(133576051_133681919)x1
arr[GRCh37] 15q13.2(30652489_31004749)x1
arr[GRCh37] 15q13.3(31985493_32510863)x3

This is an intermediate report and not a final signed off report

Analysis Settings

Design	:	021850_20150623	Sample Name	:	10-0206-FM-25218502255 7_1_1
Genome	:	hg19	Aberration Algorithm	:	ADM-2
Threshold	:	6.0	Fuzzy Zero	:	OFF
GC Correction	:	ON	Window Size	:	2Kb
Centralization (legacy)	:	OFF	Diploid Peak Centralization	:	ON
SNP Copy Number	:	OFF	LOH	:	OFF
Combine Replicates (Intra Array)	:	ON	Array Level Filter	:	NONE
			Aberration Filter Name	:	Default Aberration Filter
Metric Set Filter	:	NONE	Feature Level Filter	:	gIsSaturated = true OR rIsSaturated = true OR gIsFeatNonUnifOL = true OR rIsFeatNonUnifOL = true OR LogRatio = 0; Include matching values=false
Aberration Filter	:	Minimum Number of Probes for Amplification >= 3 AND Nesting Level <= 100 AND Minimum Avg. Absolute Log Ratio for Amplification >= 0.25 AND Minimum Size (Kb) of Region for Amplification >= 0.0 AND Minimum Size (Kb) of Region for Deletion >= 0.0 AND Minimum Number of Probes for Deletion >= 3 AND Minimum Avg. Absolute Log Ratio for Deletion >= 0.25 AND Minimum Number of Probes for Gain >= 3 AND Minimum Number of Probes for Loss >= 3 AND Minimum Avg. Absolute Log Ratio for Gain >= 0.25 AND Minimum Avg. Absolute Log Ratio for Loss >= 0.25 AND Minimum Size (Kb) of Region for Gain >= 0.0 AND Minimum Size (Kb) of Region for Loss >= 0.0	LOH Filter	:	NONE
			Show Flat Intervals	:	false
Design Level Filter	:	Homology = 0 OR IsPseudoautosomal = 1			
Genomic Boundary	:	OFF			
Template Name	:	ouhsc-cgh			

Genomic region filter for Aberration: OFF

Genomic region filter for LOH: OFF

Notes

Sample Notes

No notes available.

This is an intermediate report and not a final signed off report

Amp/Gain/Loss/Del Interval Notes

No notes available.

This is an intermediate report and not a final signed off report
