# Supplementary material for: Genomic Copy Number Variants in CML Patients With the Philadelphia Chromosome (Ph+): An Update
Source: Front Genet. 2021 Aug 10;12:697009. doi: 10.3389/fgene.2021.697009 (PMC8383316; doi:10.3389/fgene.2021.697009)
Supplement: Supplementary file 8 [file Data_Sheet_8.PDF]

Sample Information

Array ID	:	252185024053_1_1
Global Display Name	:	15-0588-FM-0519D-Dav,C-252185024053_1_1
Green Sample	:	Agilent Euro Female
Red Sample	:	
Polarity	:	1
DerivativeOfLogRatioSD	:	0.167094
Intermediate Report by	:	OUHSC\xwang3

This is an intermediate report and not a final signed off report

Genome View (Amp/Del)

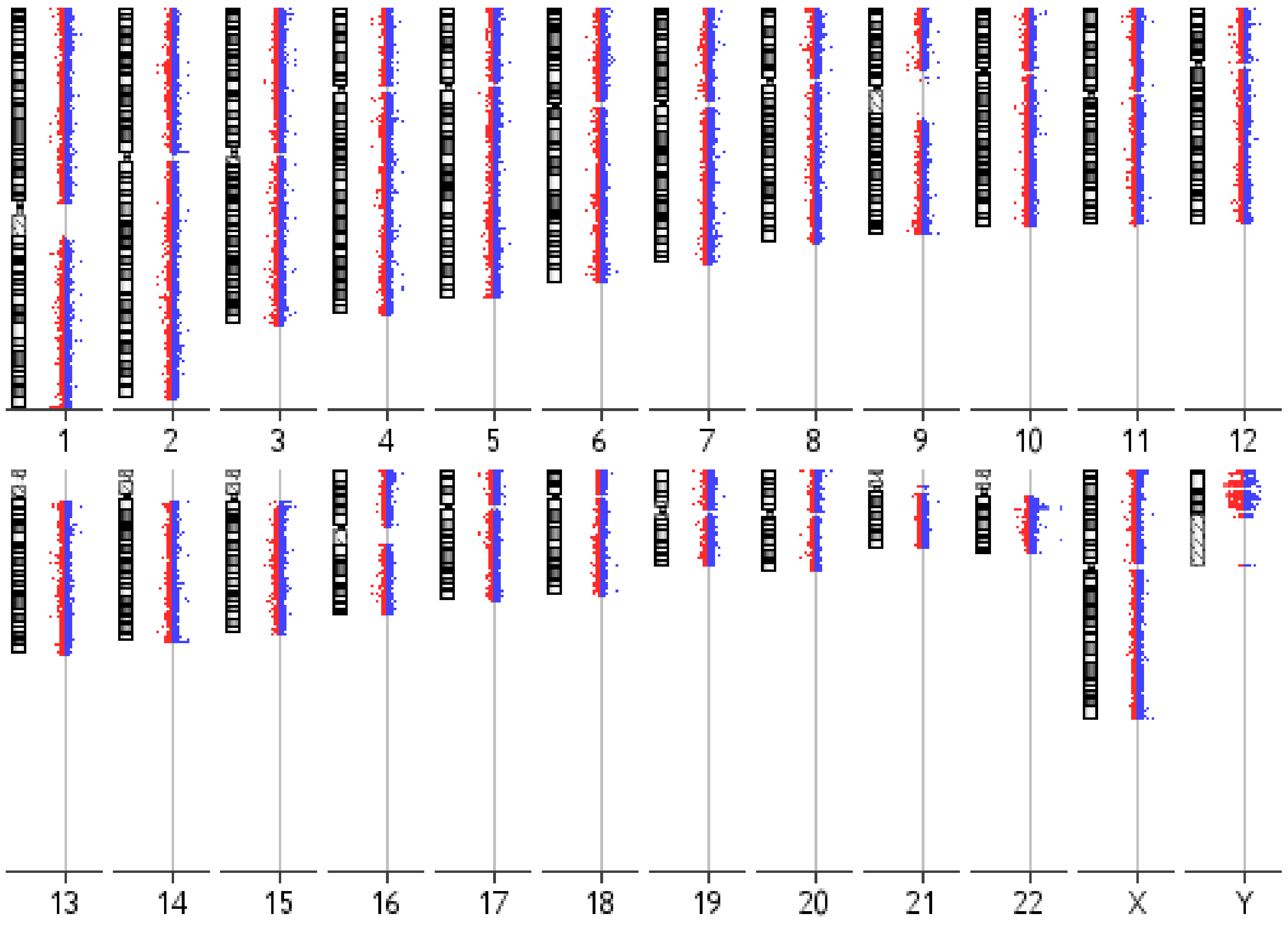

This is an intermediate report and not a final signed off report

Chromosome Views (Amp/Del)

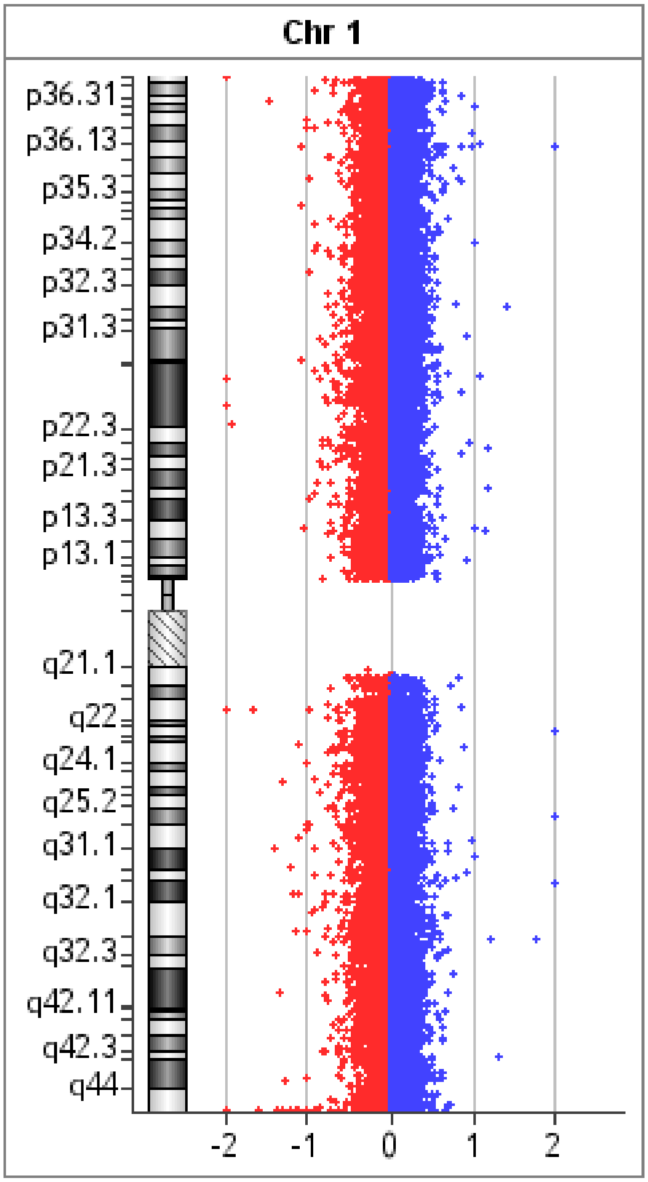

This is an intermediate report and not a final signed off report

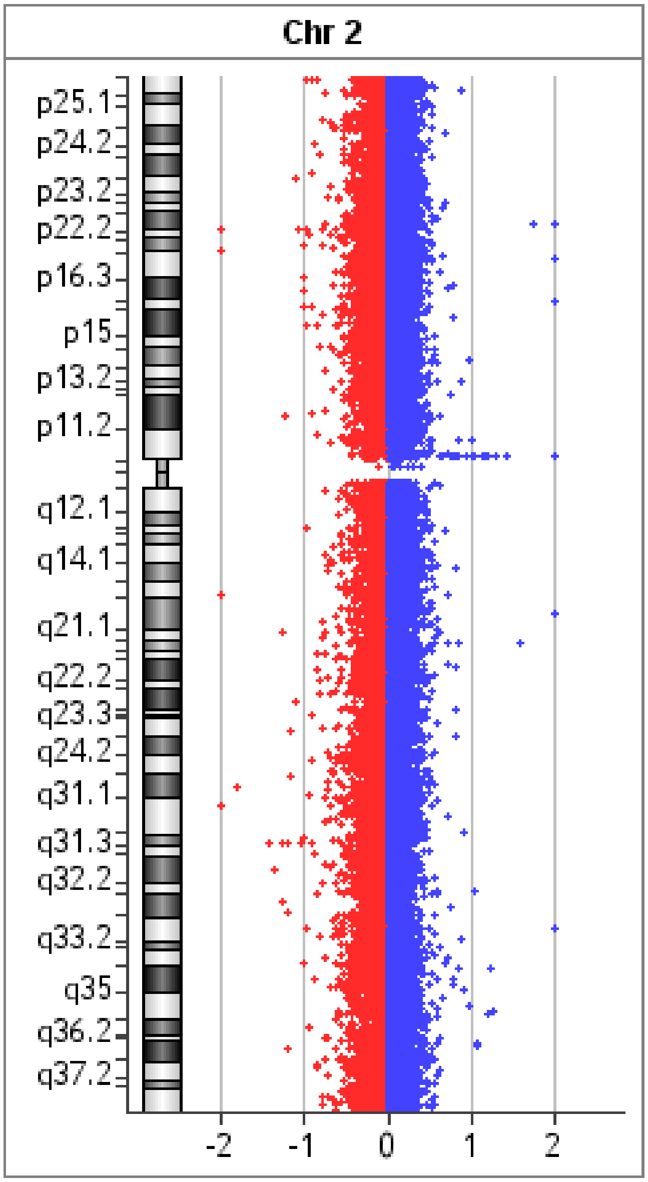

This is an intermediate report and not a final signed off report

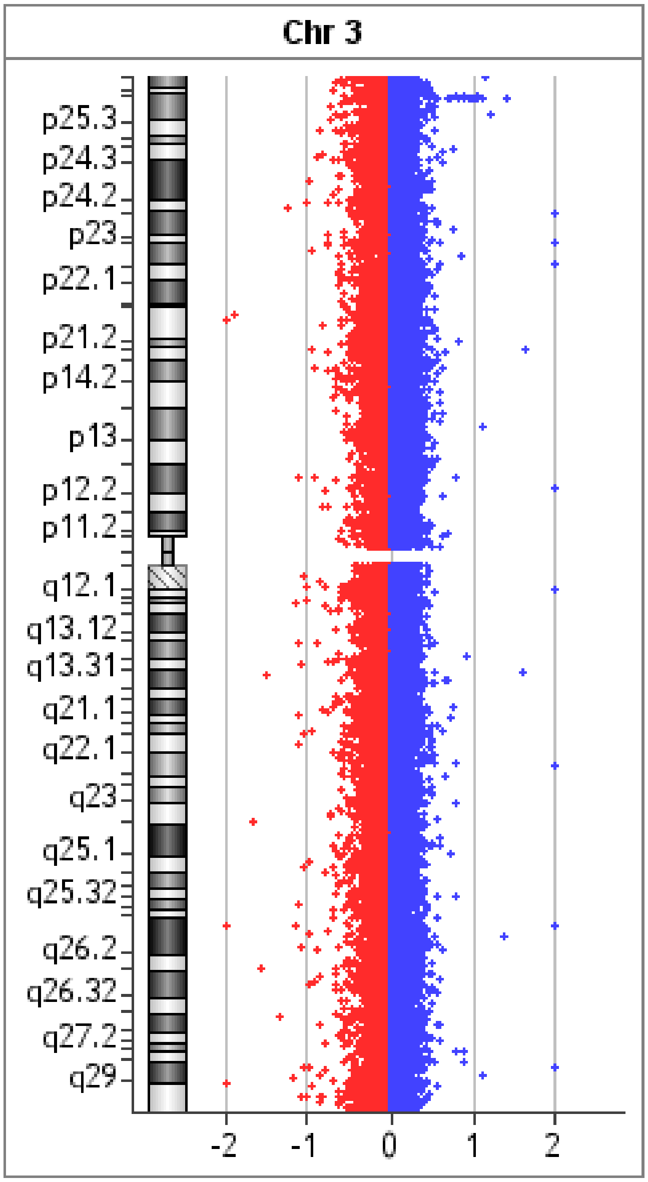

This is an intermediate report and not a final signed off report

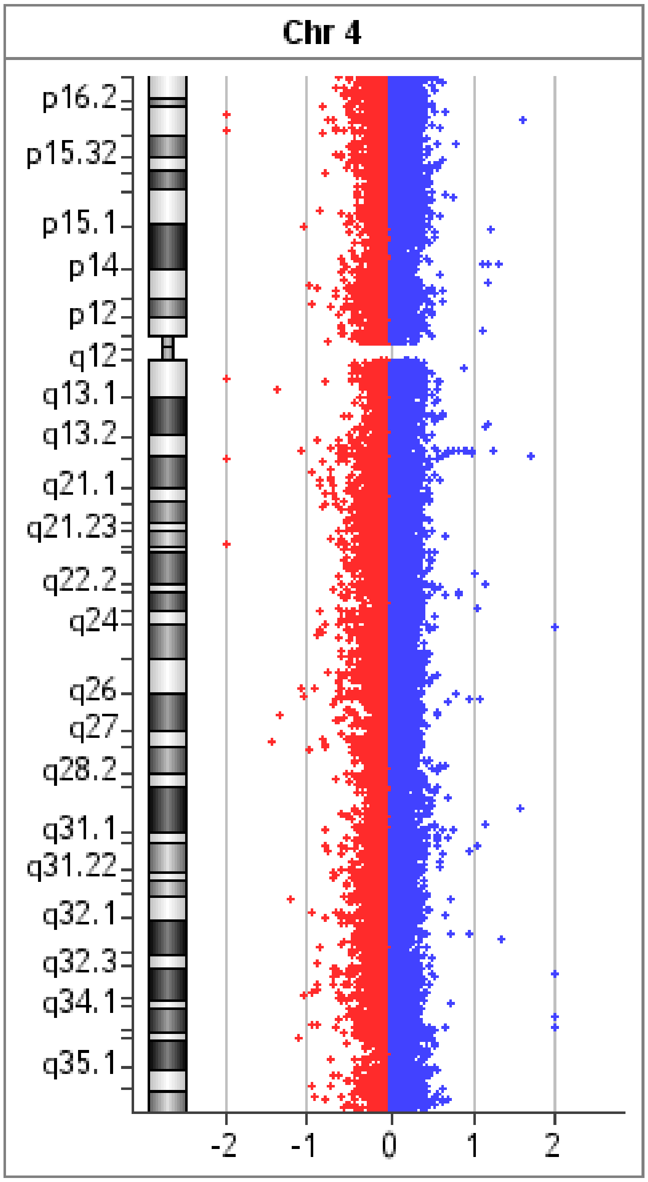

This is an intermediate report and not a final signed off report

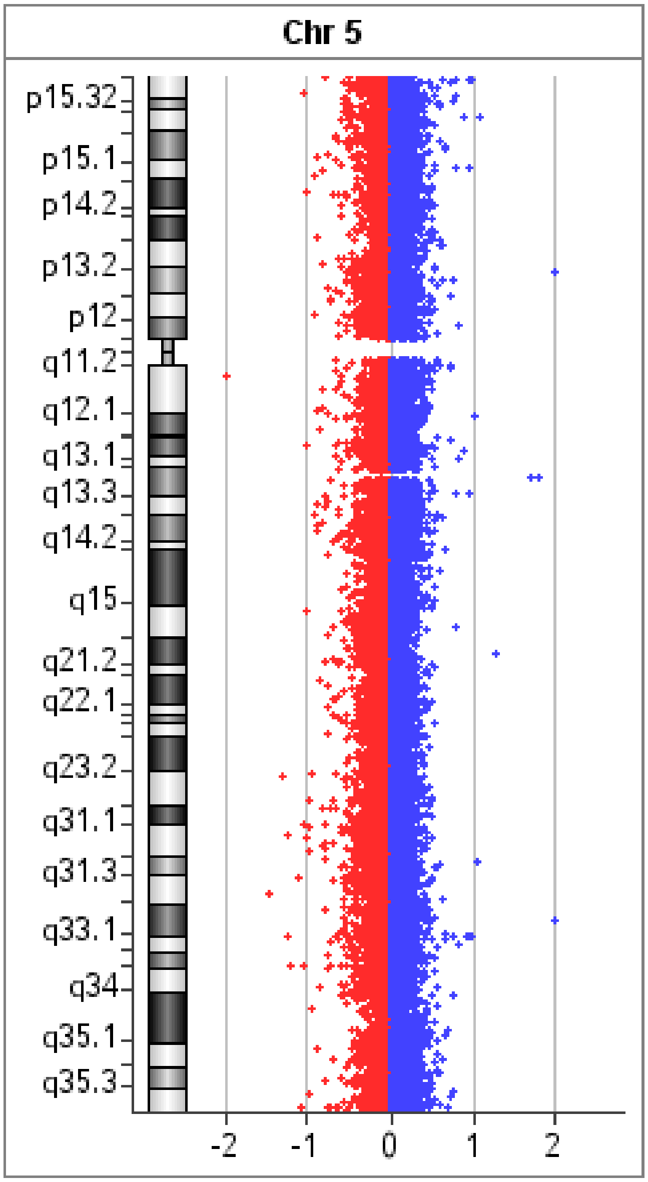

This is an intermediate report and not a final signed off report

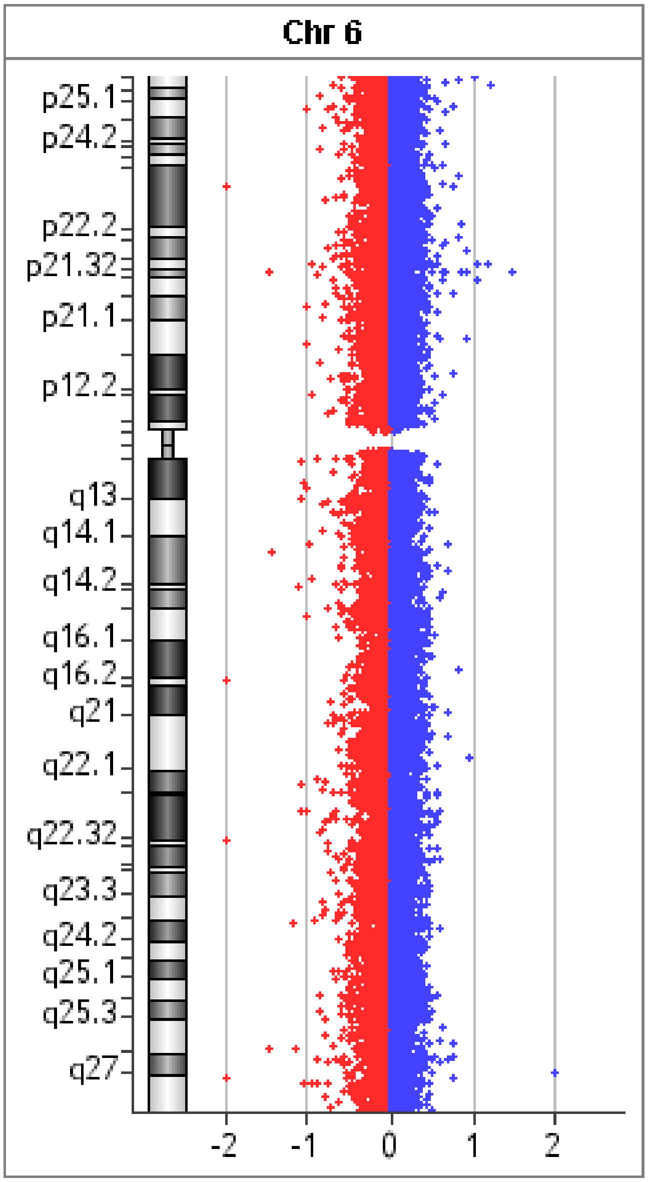

This is an intermediate report and not a final signed off report

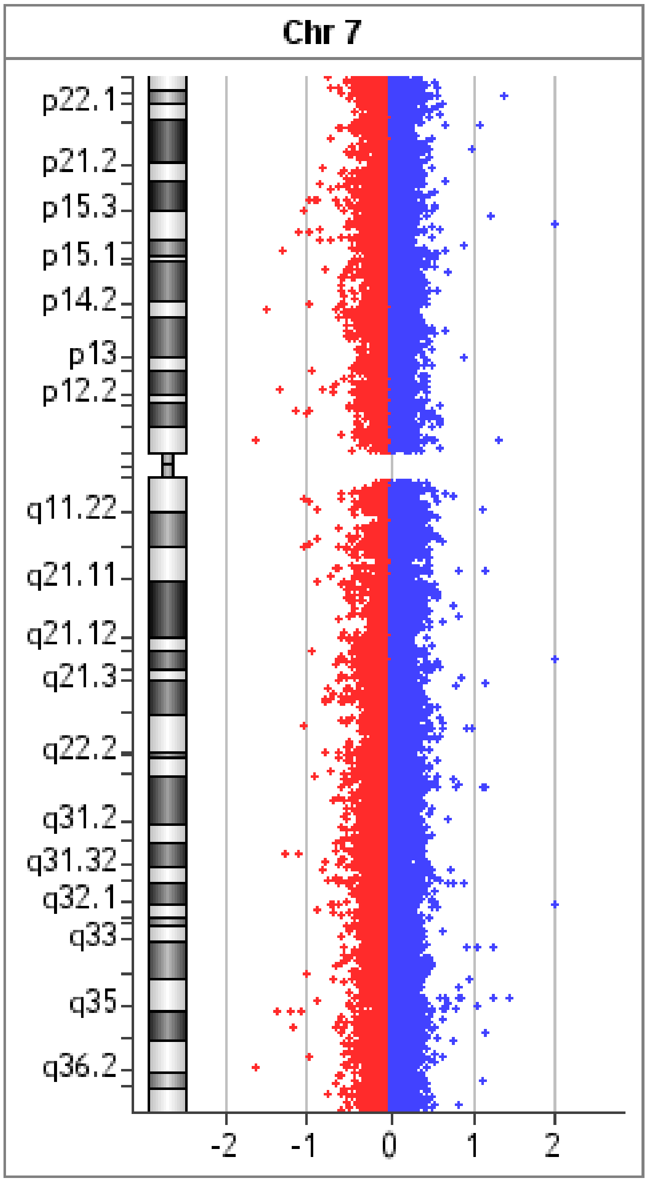

This is an intermediate report and not a final signed off report

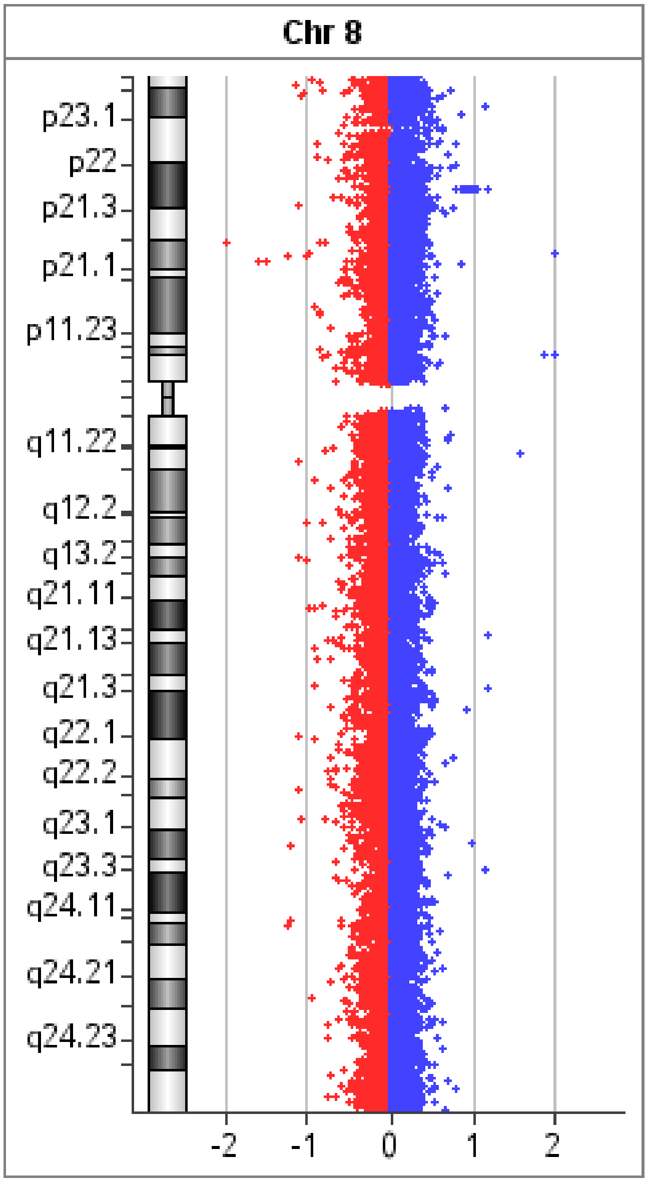

This is an intermediate report and not a final signed off report

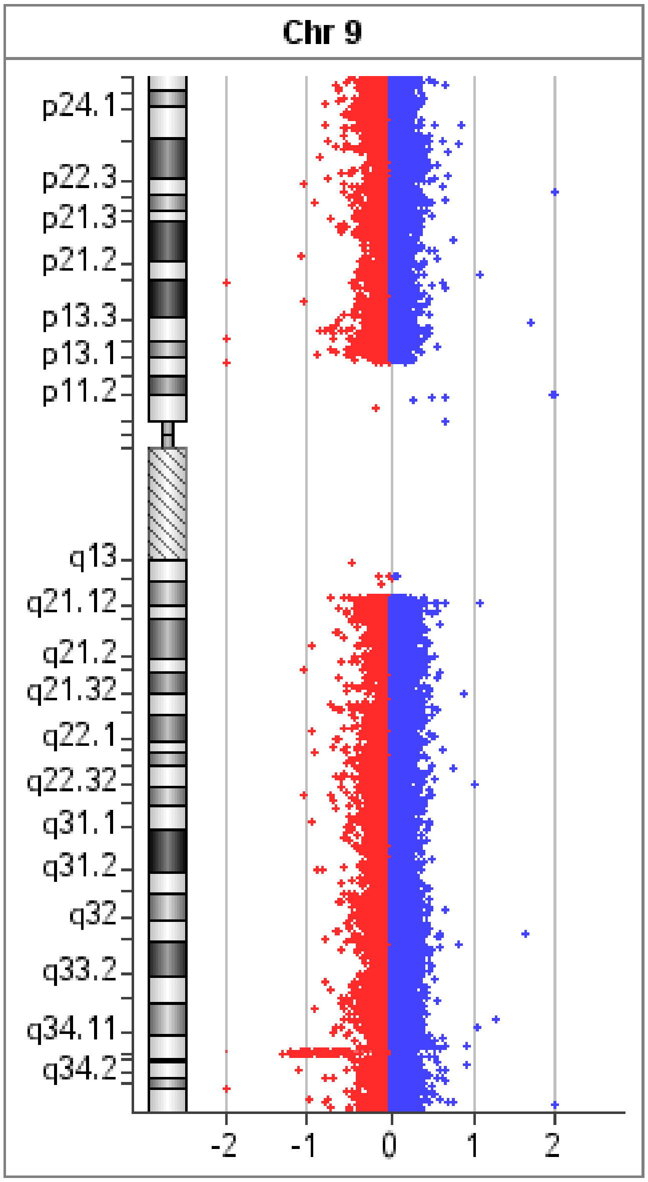

This is an intermediate report and not a final signed off report

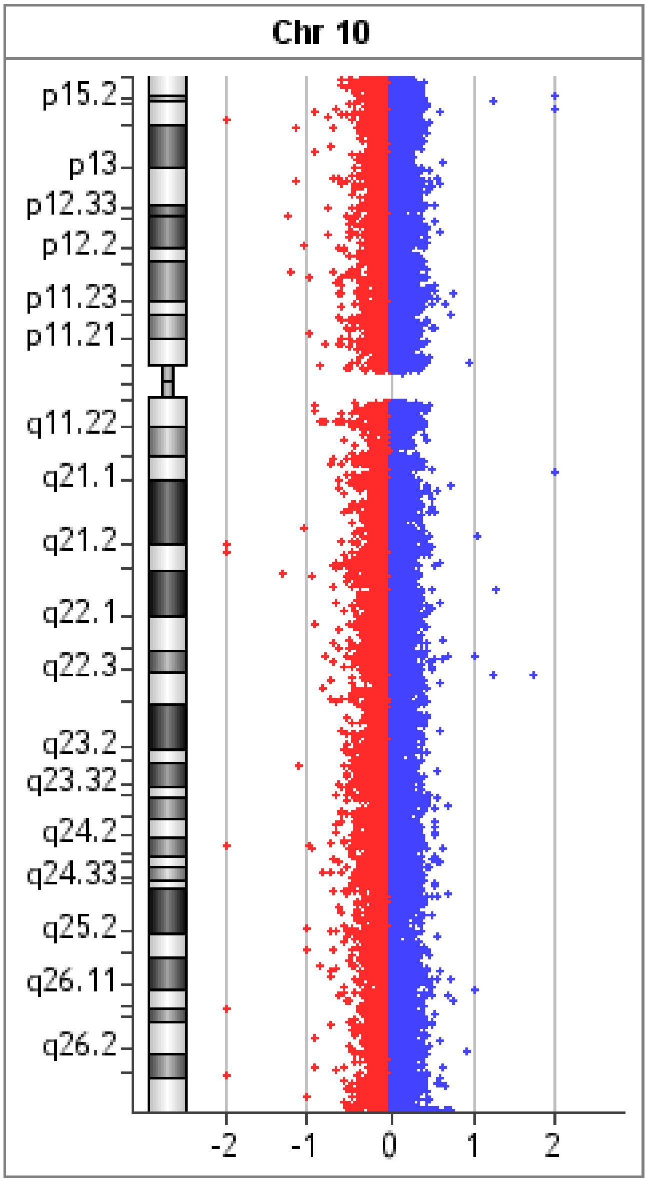

This is an intermediate report and not a final signed off report

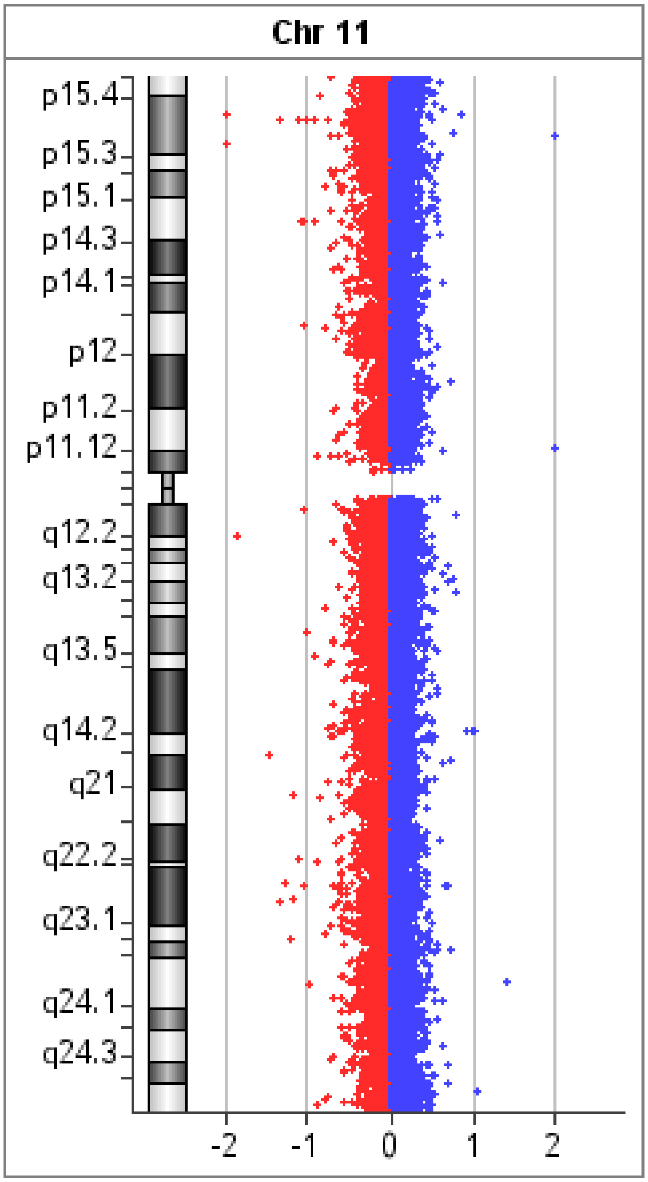

This is an intermediate report and not a final signed off report

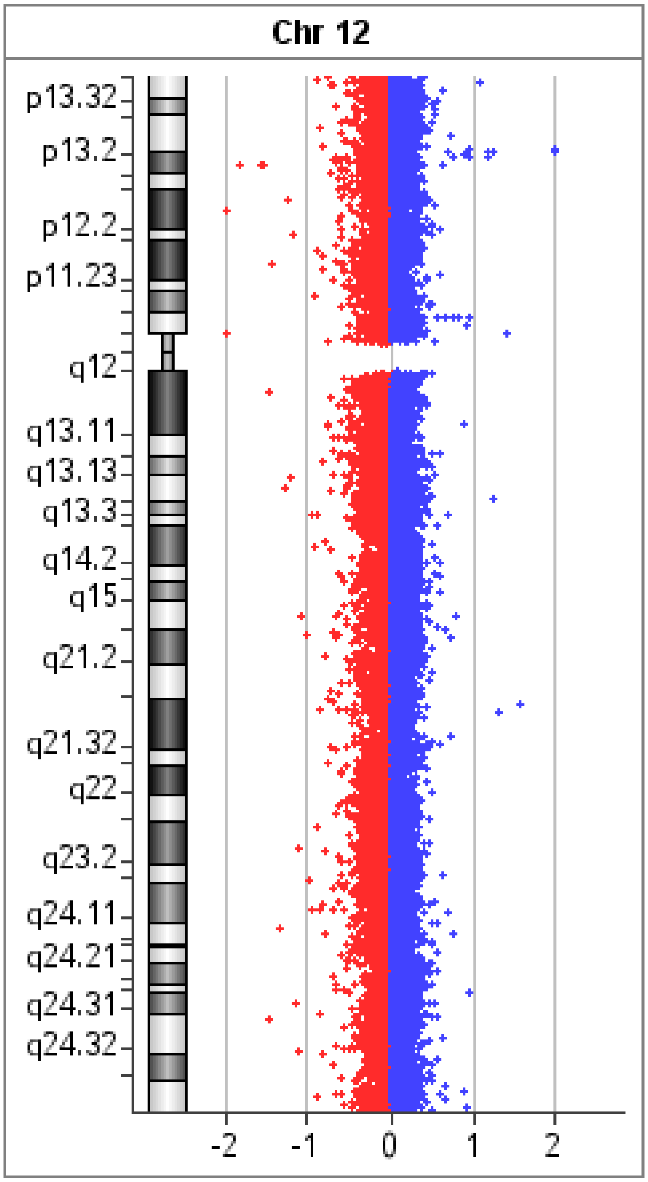

This is an intermediate report and not a final signed off report

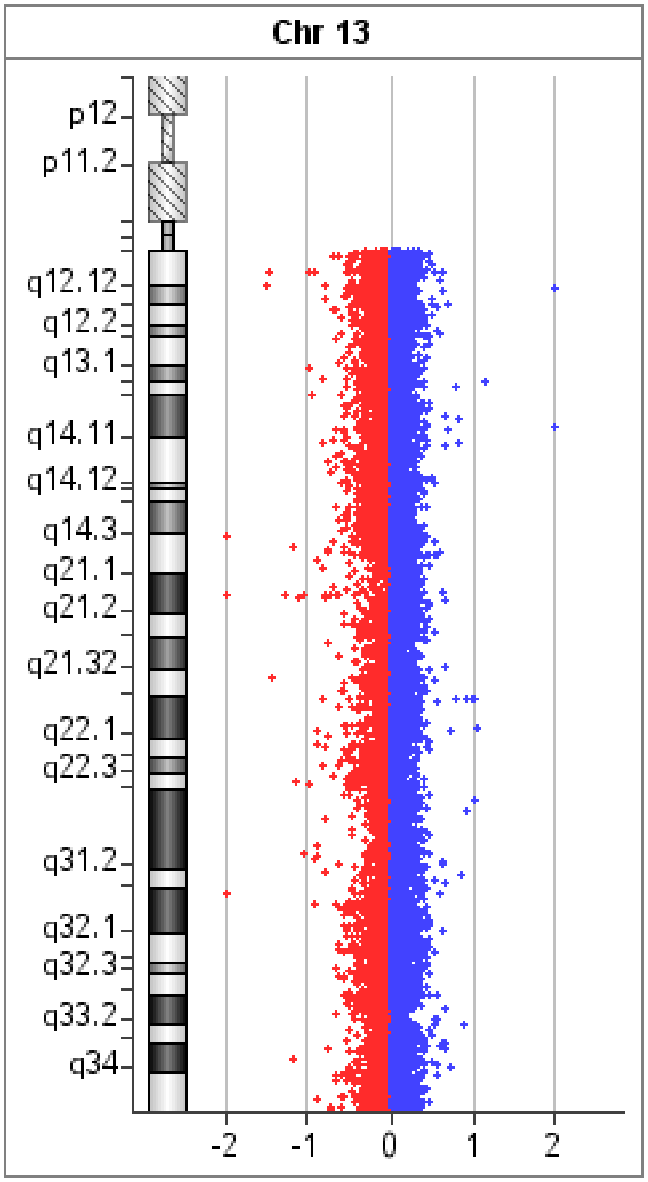

This is an intermediate report and not a final signed off report

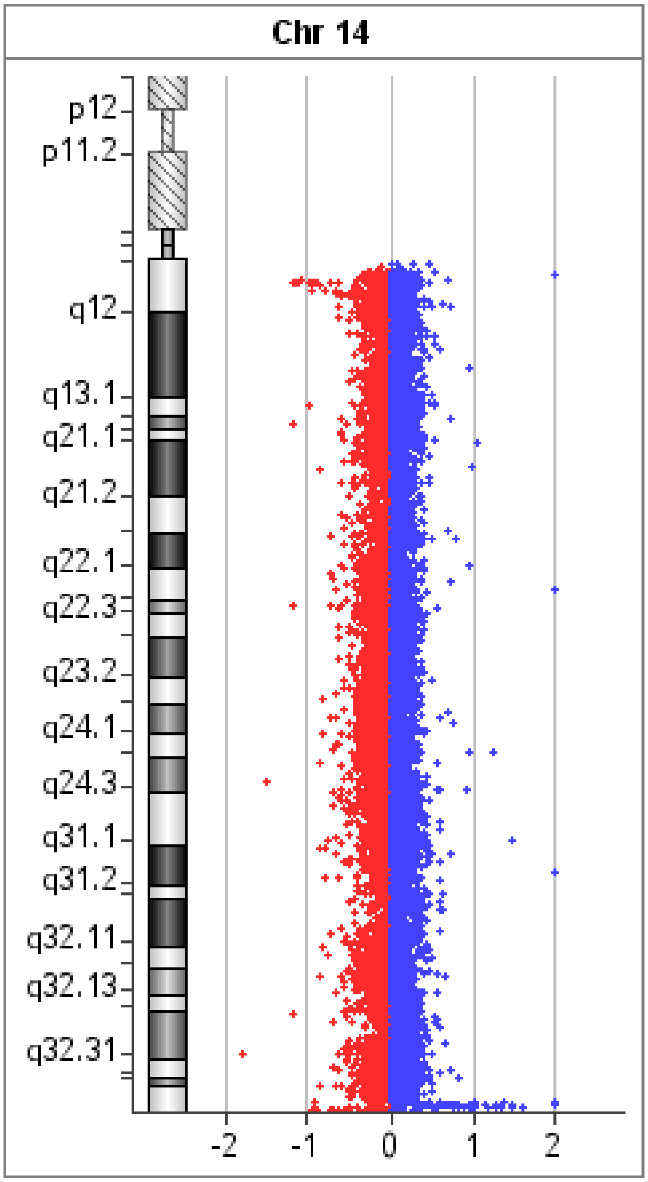

This is an intermediate report and not a final signed off report

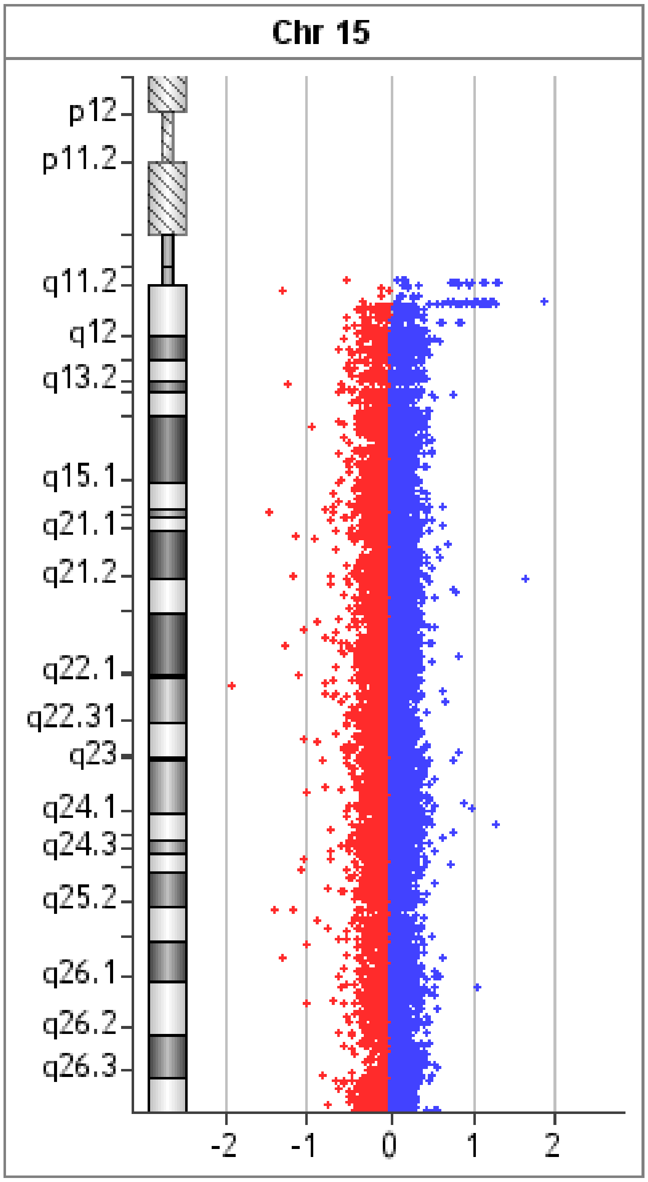

This is an intermediate report and not a final signed off report

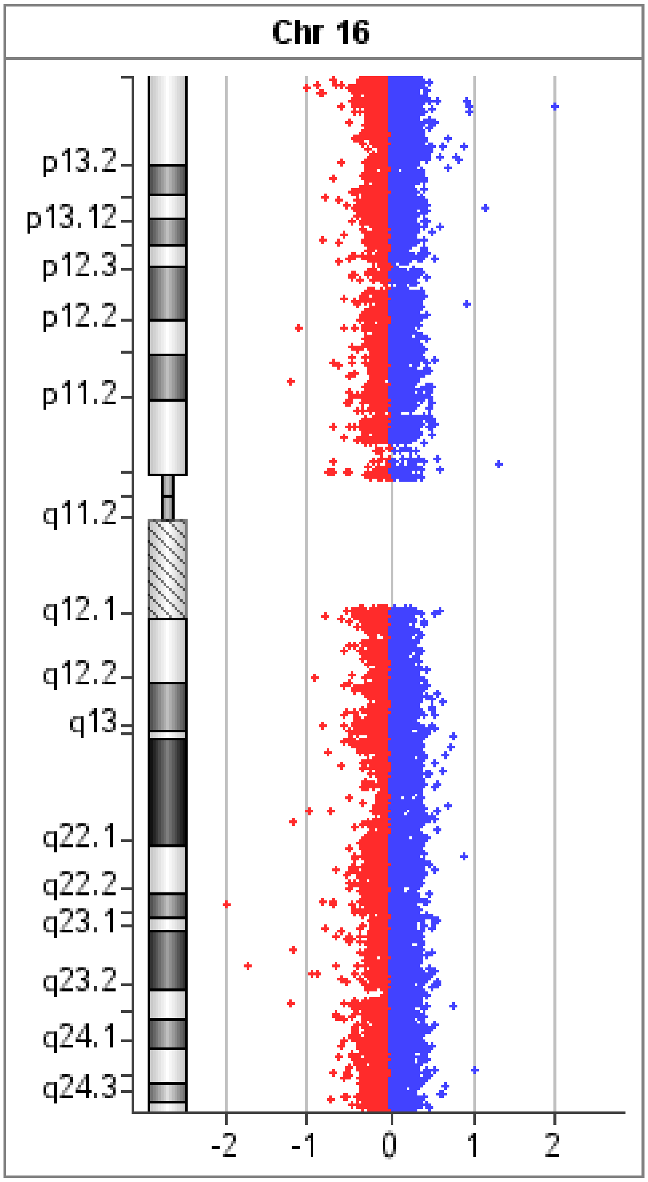

This is an intermediate report and not a final signed off report

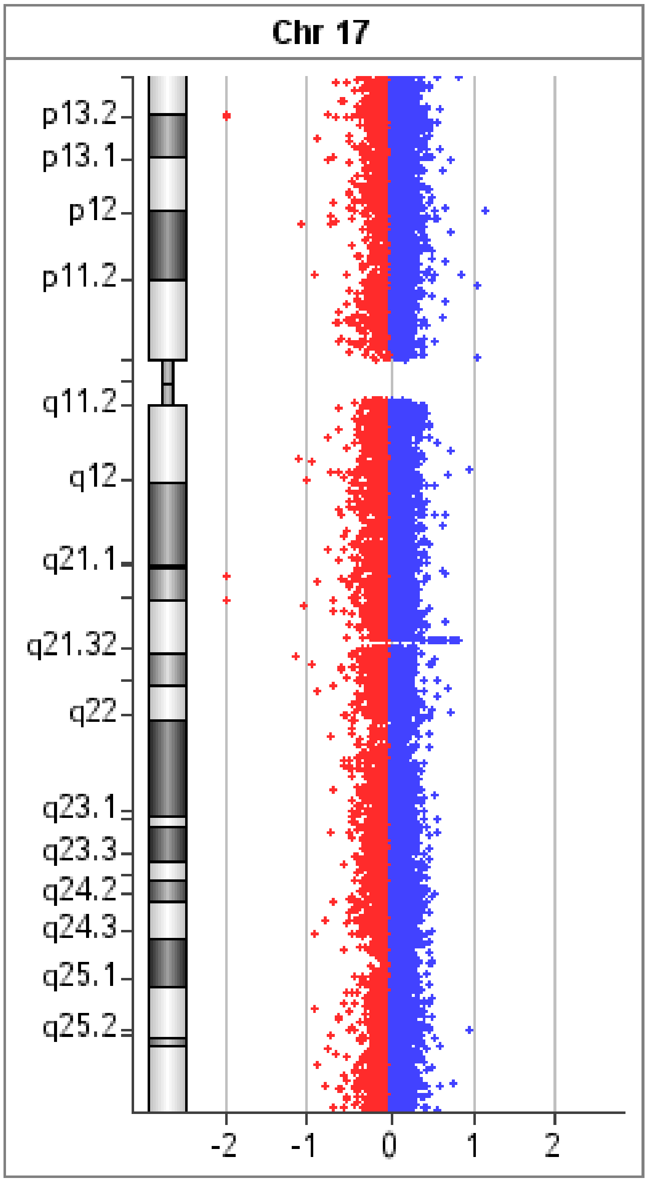

This is an intermediate report and not a final signed off report

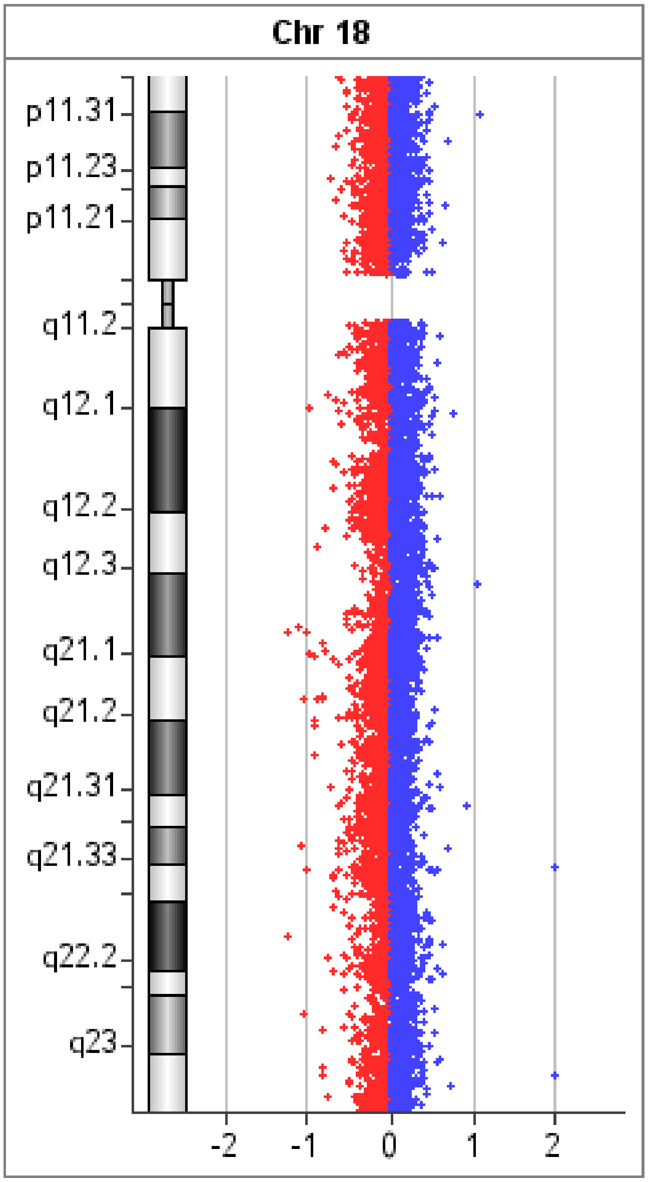

This is an intermediate report and not a final signed off report

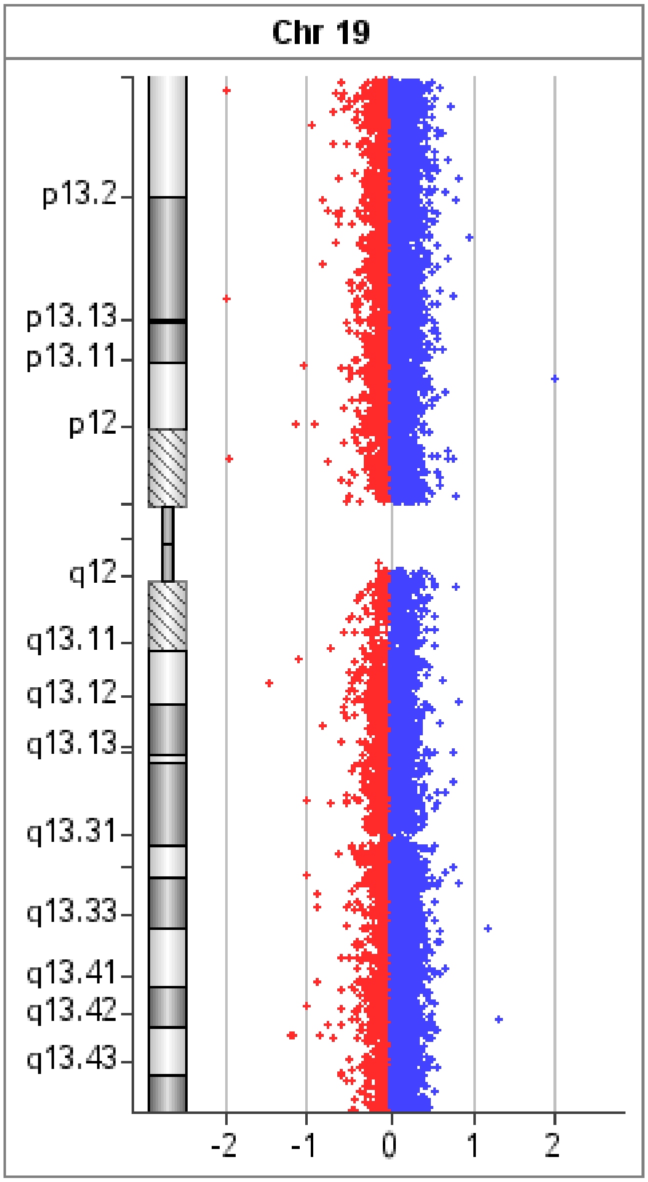

This is an intermediate report and not a final signed off report

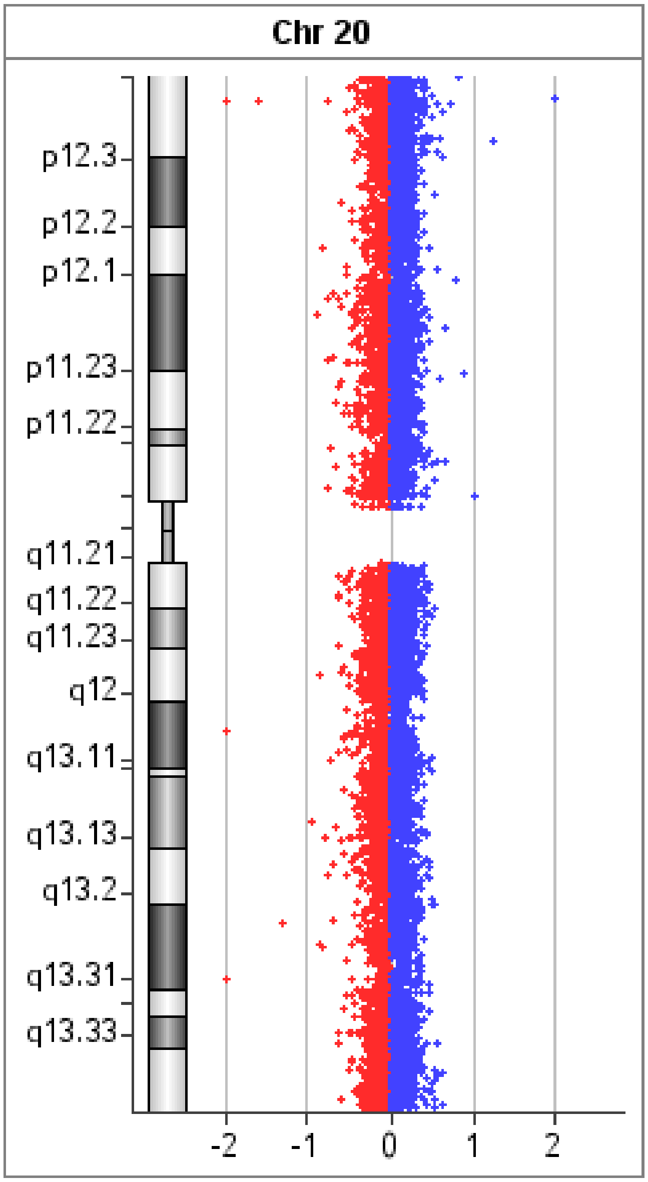

This is an intermediate report and not a final signed off report

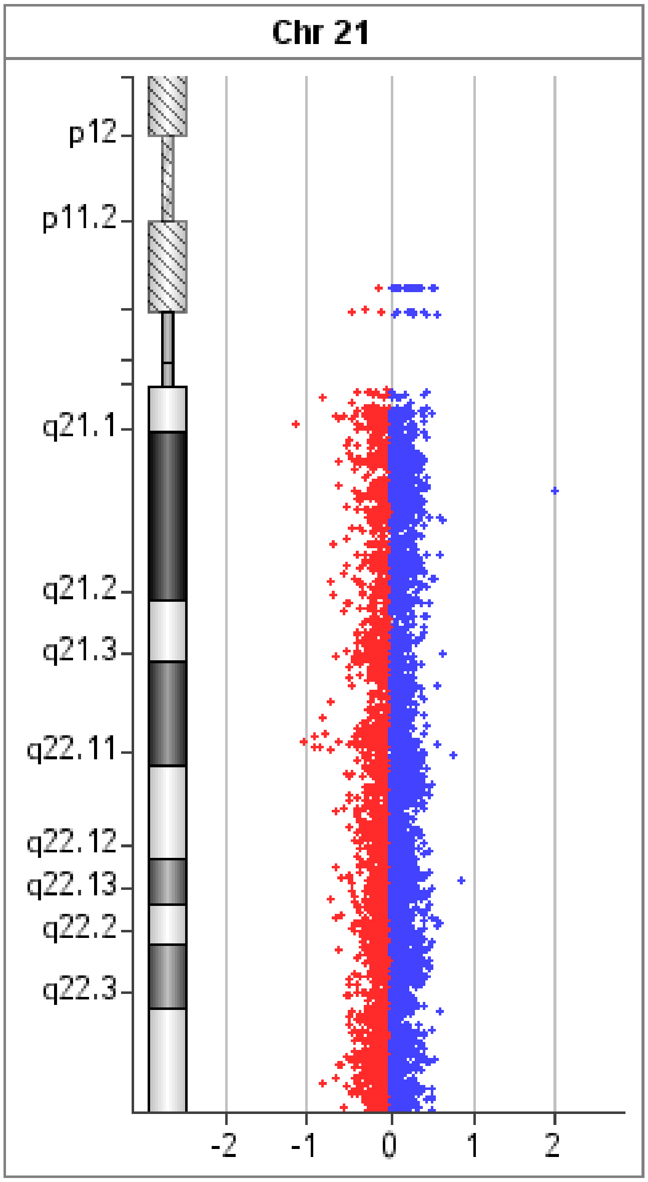

This is an intermediate report and not a final signed off report

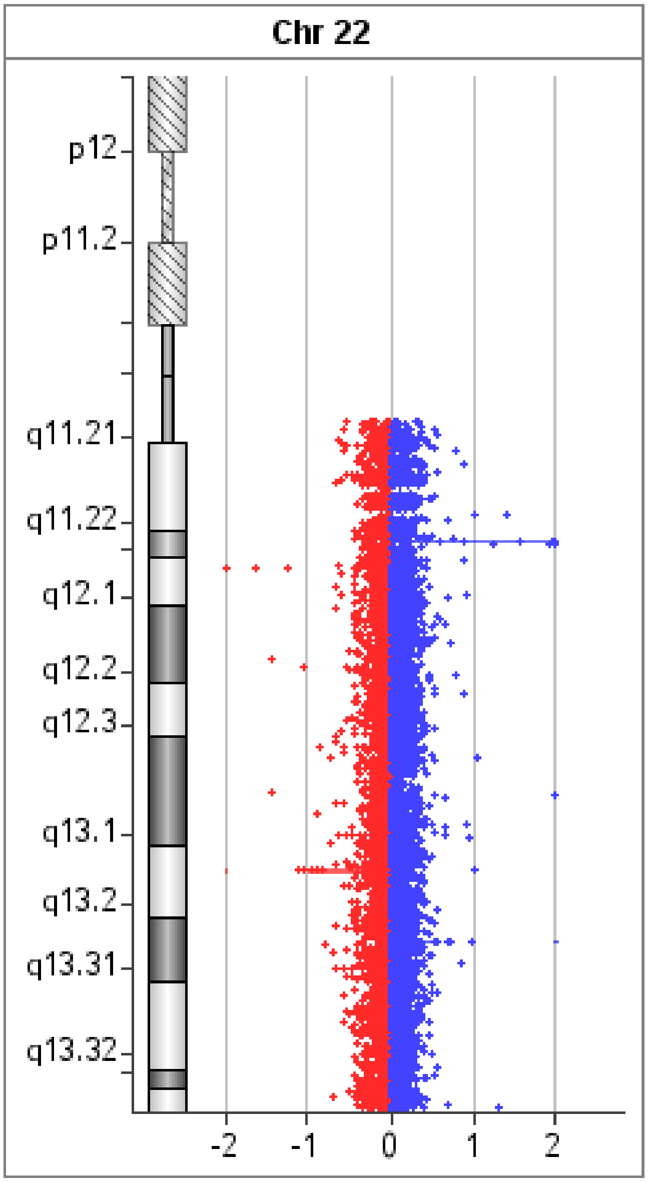

This is an intermediate report and not a final signed off report

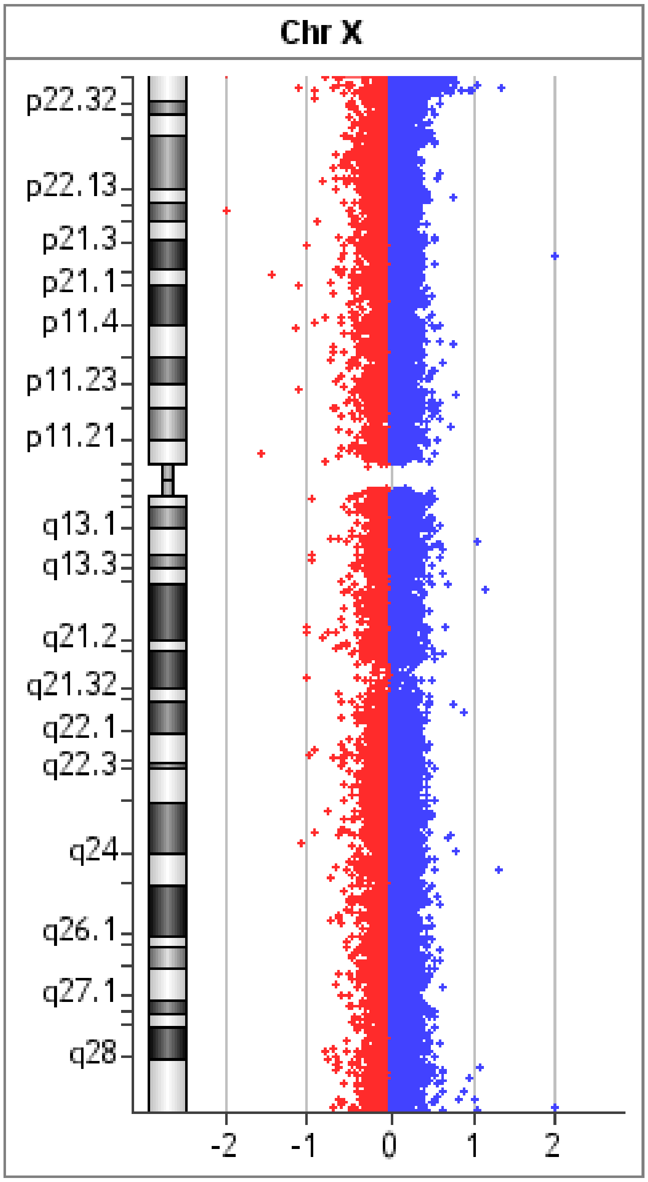

This is an intermediate report and not a final signed off report

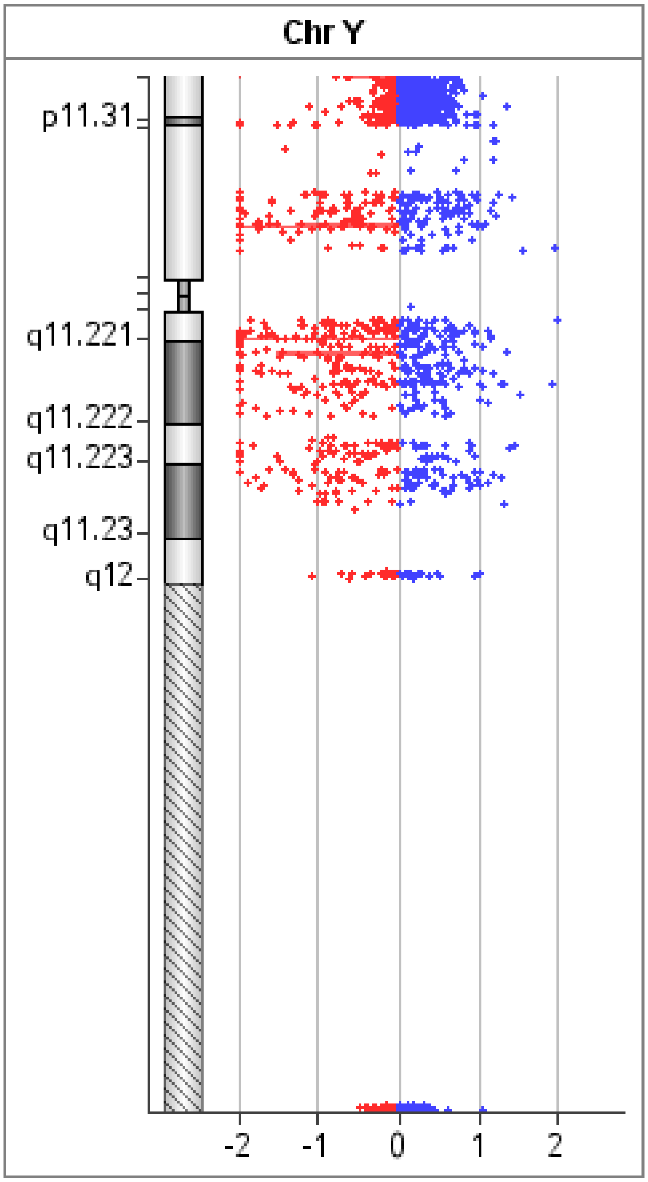

This is an intermediate report and not a final signed off report

Amp/Gain/Loss/Del Intervals Table

Chr	Start-Stop(bp)	Size(kb)	Cytoband	#Probes	Amp/Gain/Loss/Del	Annotations	Classifications
chr9	132870231-133562475	692	q34.11 - q34.12	91	-0.816928	GPR107, NCS1, ASS1...	pathogenic
chr22	23056562-23245888	189	q11.22	50	2.485444	MIR650, IGLL5, nssv576708_unk...	
chr22	39359112-39385485	26	q13.1	6	-0.976504	APOBEC3A, APOBEC3B, nsv915079...	
chr22	42918229-42949585	31	q13.2	5	0.614247	dgv1407e1, nsv428391, essv4940...	
chrX	81548-92387	11	p22.33	4	-0.675604	esv24825, nssv579164_unk, nssv579174_unk...	
chrY	31548-42387	11	p11.32	4	-0.675604	nssv706369_dn_M, nssv585216_unk, nssv1415445_unk...	
chrY	8441447-8581738	140	p11.2	15	-1.274365	TTTY18, TTTY19, nsv7473...	
chrY	8641183-8665425	24	p11.2	4	-2.315613	TTTY11, nsv7473, nssv706369_dn_M...	
chrY	15056647-15161456	105	q11.21 - q11.221	6	-1.976683	nssv706369_dn_M, nssv579297_unk, nssv579279_unk...	
chrY	15902588-15948750	46	q11.221	5	-1.506913	nssv579297_unk, nssv579279_unk, nssv579298_unk...	

Amp=Amplification Del=Deletion

Total Amp/Gain/Loss/Del Intervals: 10

This is an intermediate report and not a final signed off report

ISCN Nomenclature

arr 9q34.11q34.12(132,870,231-133,562,475)x1,22q11.22(23,056,562-23,245,888)x3,22q13.1(39,359,112-39,385,485)x1,22q13.2(42,918,229-42,949,585)x3

This is an intermediate report and not a final signed off report

Analysis Settings

Design	021850_20111015	Sample Name	15-0588-FM-0519D-Dav,C -252185024053_1_1
Genome	hg19	Aberration Algorithm	ADM-2
Threshold	6.0	Fuzzy Zero	OFF
GC Correction	ON	Window Size	2Kb
Centralization (legacy)	OFF	Diploid Peak Centralization	ON
SNP Copy Number	OFF	LOH	OFF
Combine Replicates (Intra Array)	ON	Array Level Filter	NONE
Metric Set Filter	NONE	Aberration Filter	Minimum Number of Probes for Amplification >= 3 AND Nesting Level <= 100 AND Minimum Avg. Absolute Log Ratio for Amplification >= 0.25 AND Minimum Size (Kb) of Region for Amplification >= 0.0 AND Minimum Size (Kb) of Region for Deletion >= 0.0 AND Minimum Number of Probes for Deletion >= 3 AND Minimum Avg. Absolute Log Ratio for Deletion >= 0.25 AND Minimum Number of Probes for Gain >= 3 AND Minimum Number of Probes for Loss >= 3 AND Minimum Avg. Absolute Log Ratio for Gain >= 0.25 AND Minimum Avg. Absolute Log Ratio for Loss >= 0.25 AND Minimum Size (Kb) of Region for Gain >= 0.0 AND Minimum Size (Kb) of Region for Loss >= 0.0
Feature Level Filter	gIsSaturated = true OR rIsSaturated = true OR gIsFeatNonUnifOL = true OR rIsFeatNonUnifOL = true OR LogRatio = 0; Include matching values=false		
LOH Filter	NONE		
Show Flat Intervals	false		
		Design Level Filter	Homology = 0 OR IsPseudoautosomal = 1
		Genomic Boundary	OFF
		Template Name	ouhsc-cgh

This is an intermediate report and not a final signed off report

Notes

Sample Notes

No notes available.

Amp/Gain/Loss/Del Interval Notes

No notes available.

This is an intermediate report and not a final signed off report
