# Supplementary material for: Genomic Copy Number Variants in CML Patients With the Philadelphia Chromosome (Ph+): An Update
Source: Front Genet. 2021 Aug 10;12:697009. doi: 10.3389/fgene.2021.697009 (PMC8383316; doi:10.3389/fgene.2021.697009)
Supplement: Supplementary file 9 [file Data_Sheet_9.PDF]

Sample Information

Array ID	:	252185022570_1_2
Global Display Name	:	14-1385-GM-252185022570_1_2
Green Sample	:	
Red Sample	:	
Polarity	:	1
DerivativeOfLogRatioSD	:	0.143059
Intermediate Report by	:	OUHSC\xwang3

This is an intermediate report and not a final signed off report

Genome View (Amp/Del)

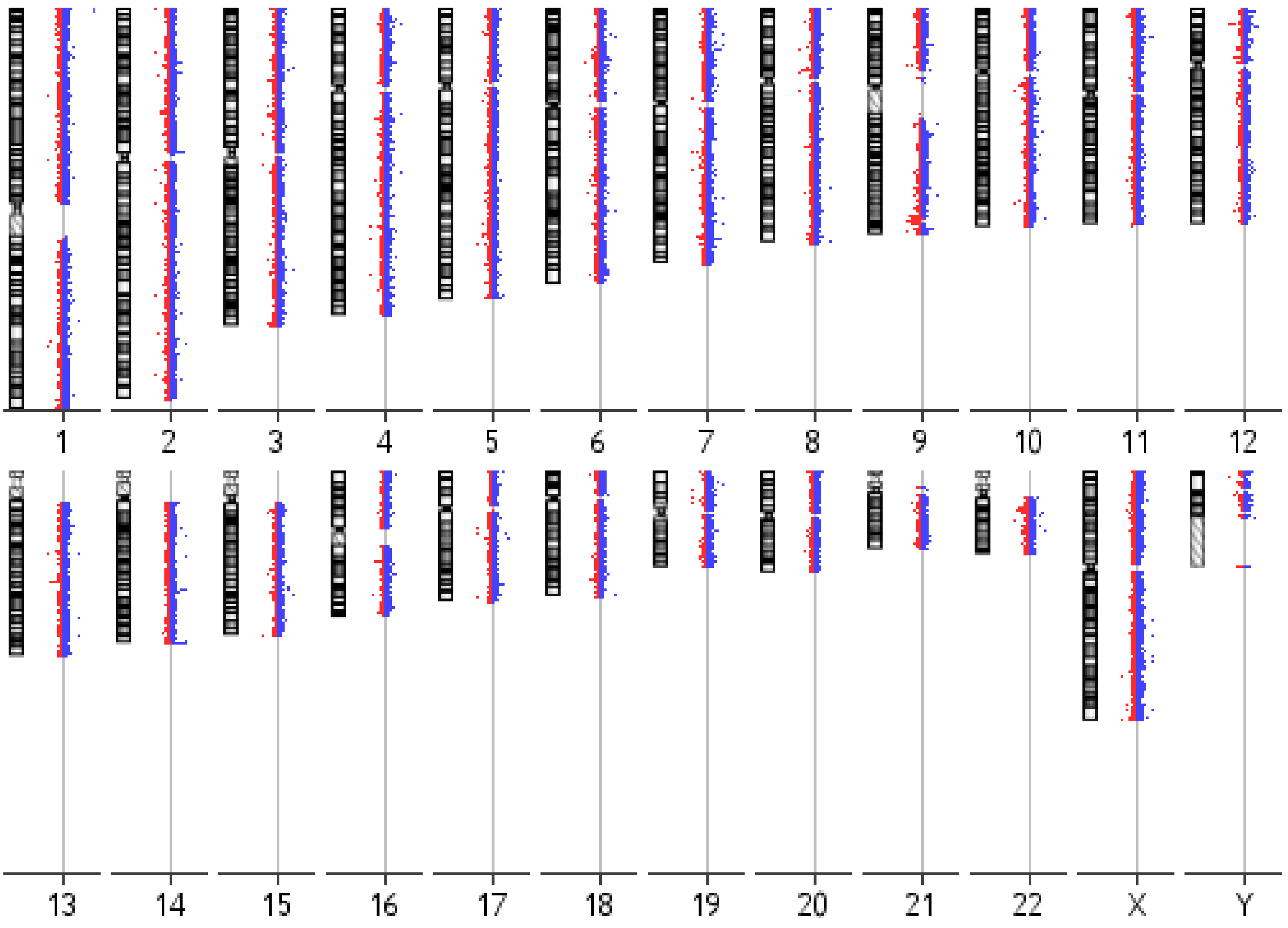

This is an intermediate report and not a final signed off report

Chromosome Views (Amp/Del)

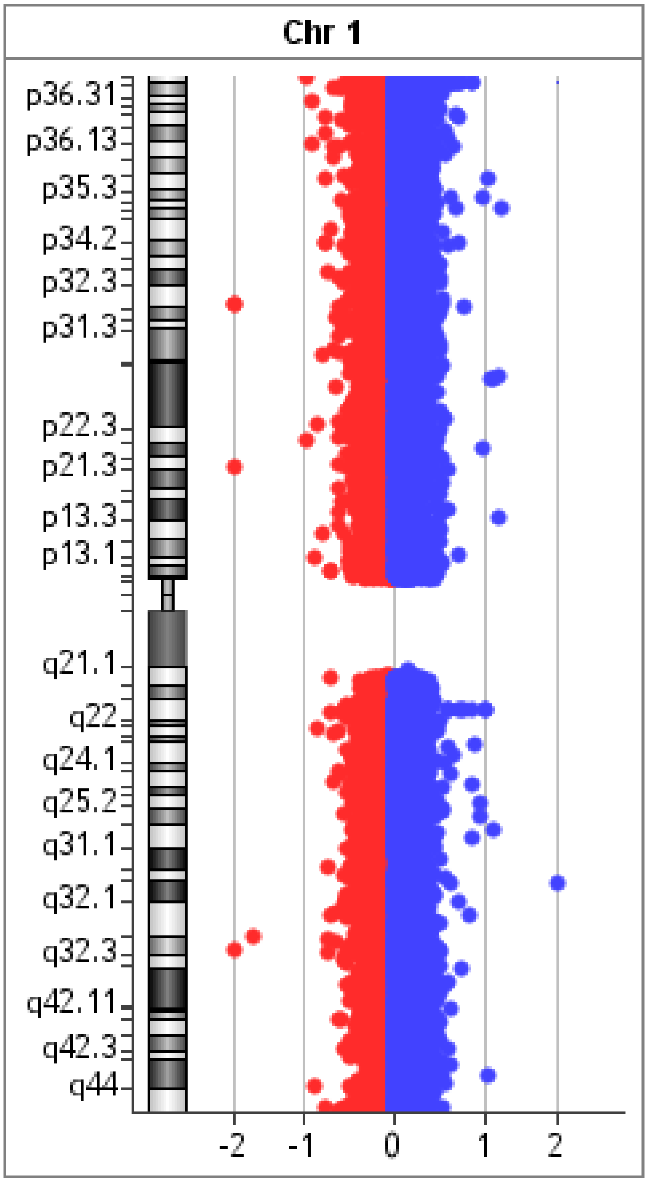

This is an intermediate report and not a final signed off report

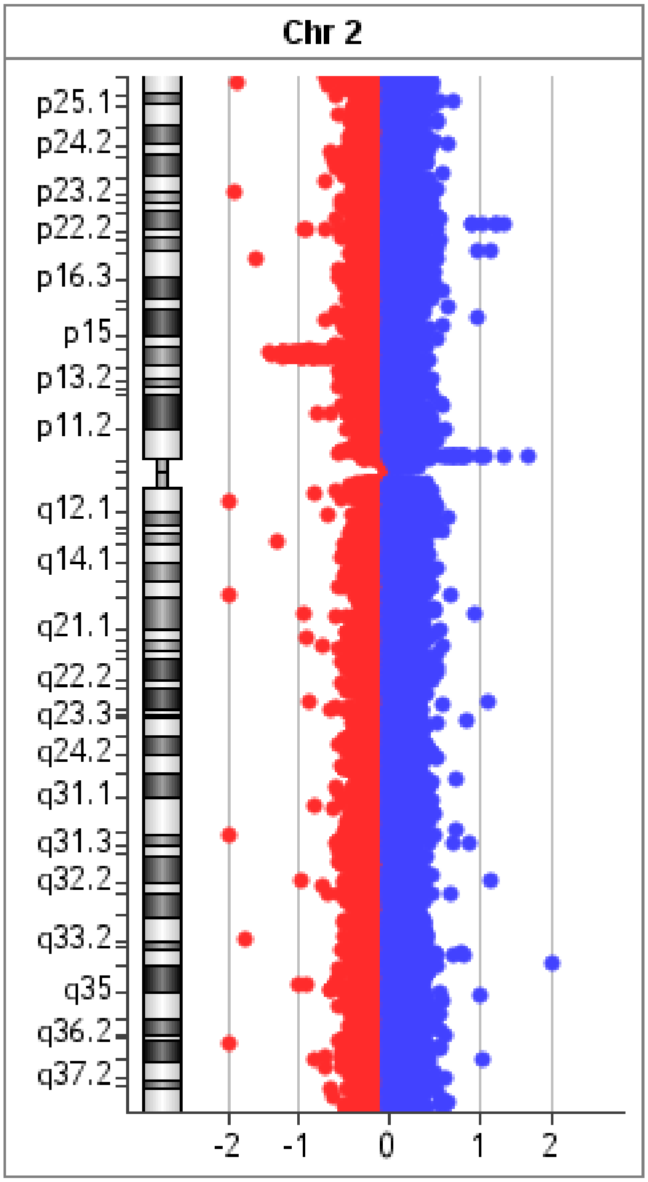

This is an intermediate report and not a final signed off report

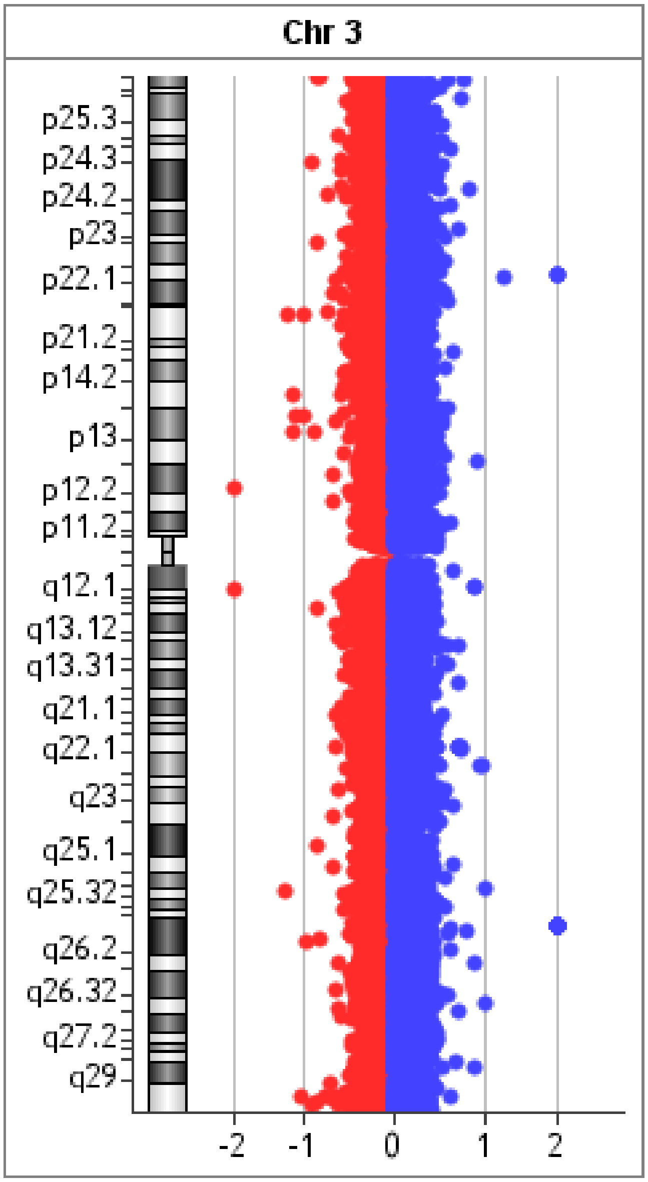

This is an intermediate report and not a final signed off report

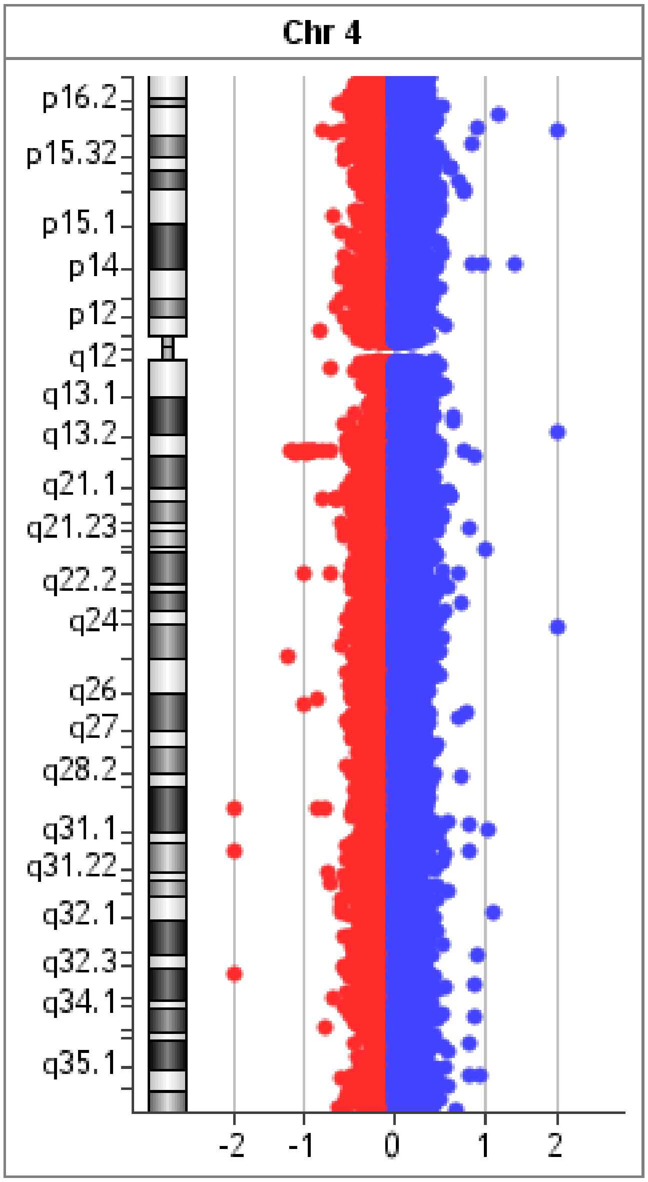

This is an intermediate report and not a final signed off report

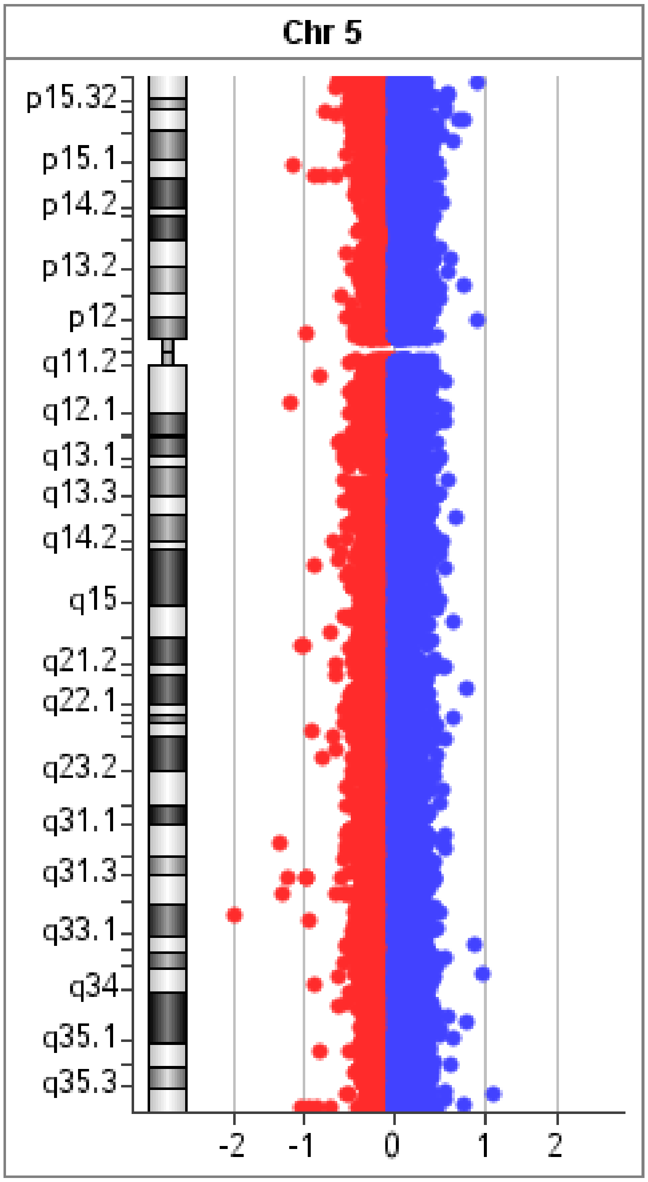

This is an intermediate report and not a final signed off report

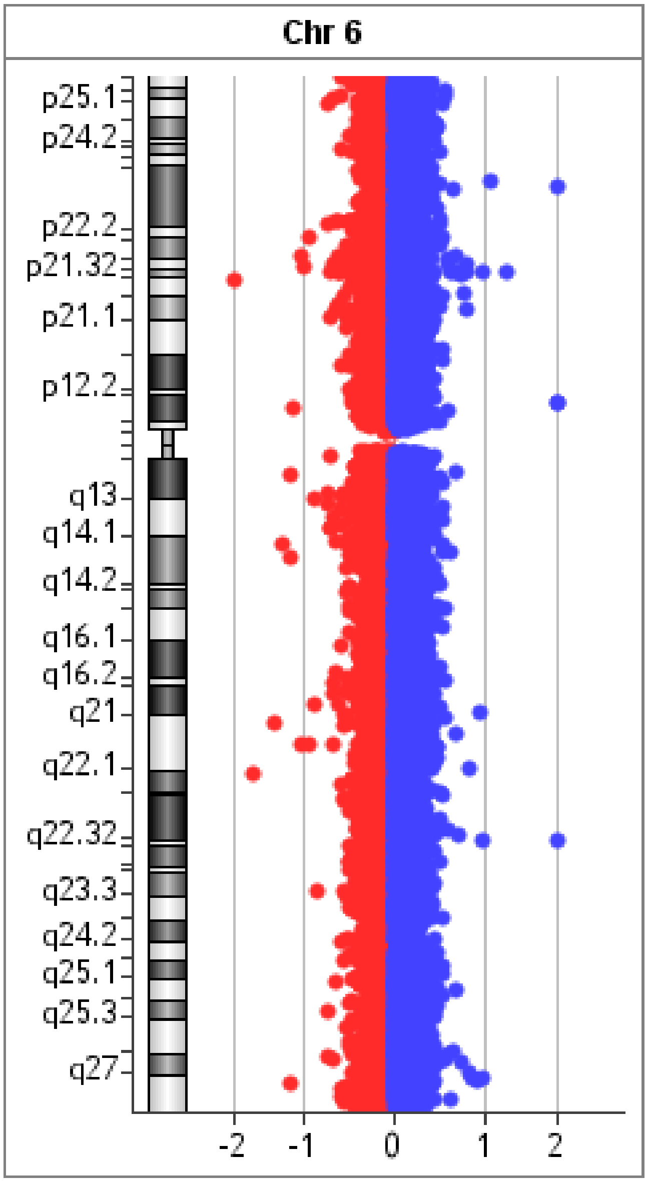

This is an intermediate report and not a final signed off report

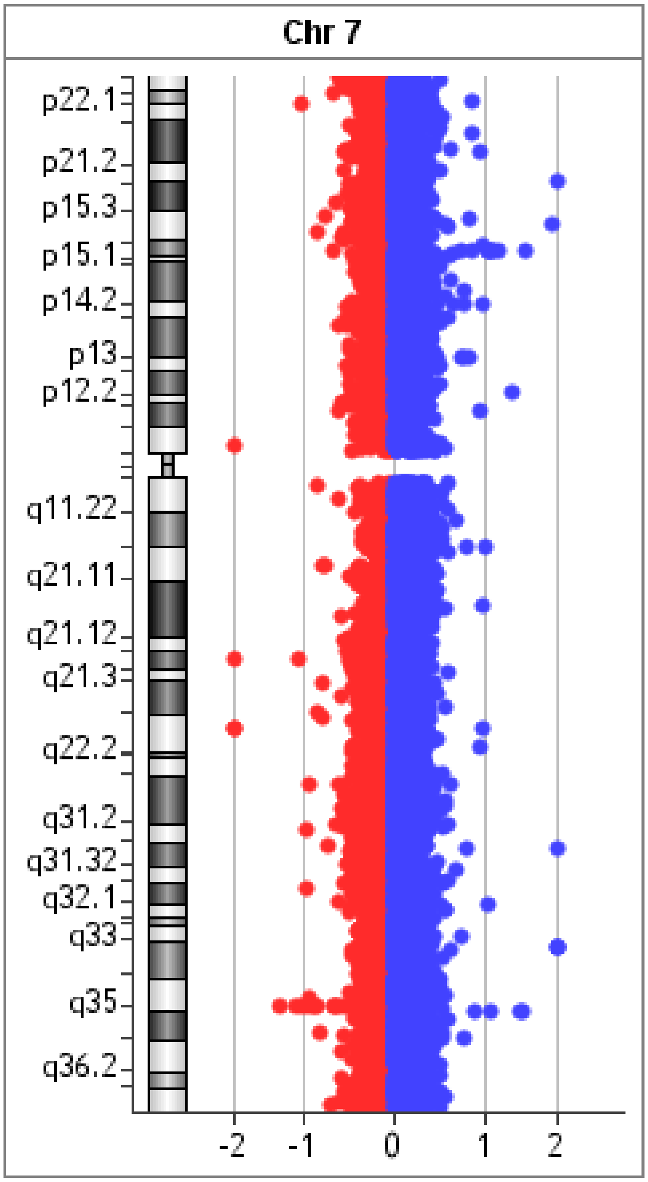

This is an intermediate report and not a final signed off report

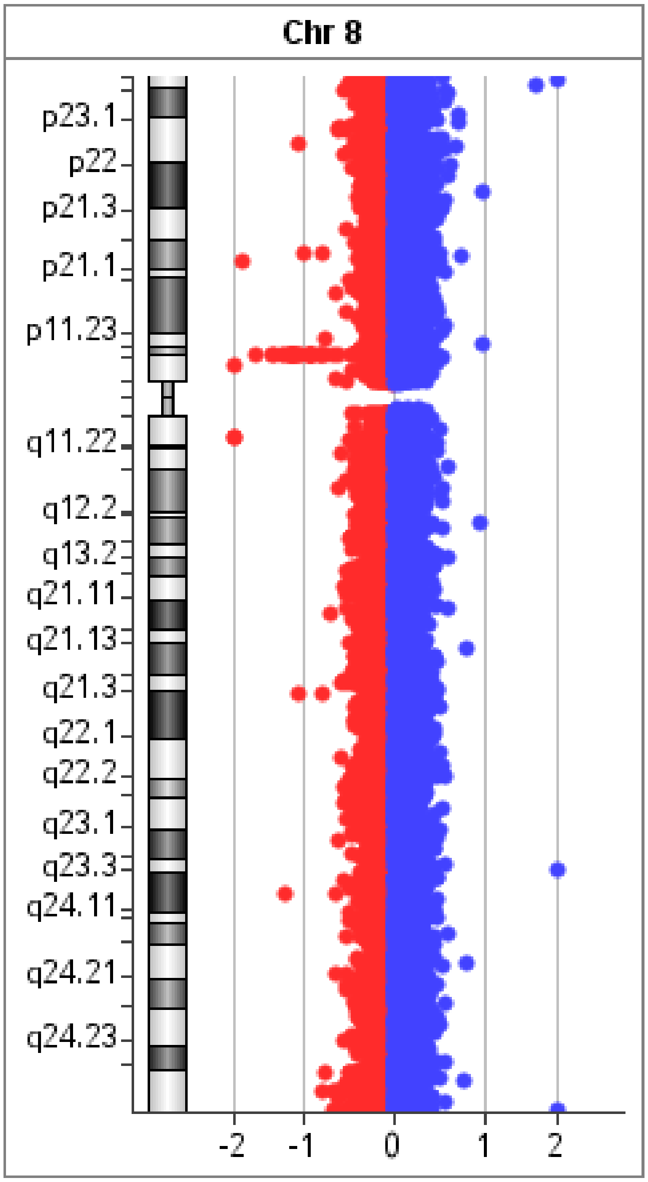

This is an intermediate report and not a final signed off report

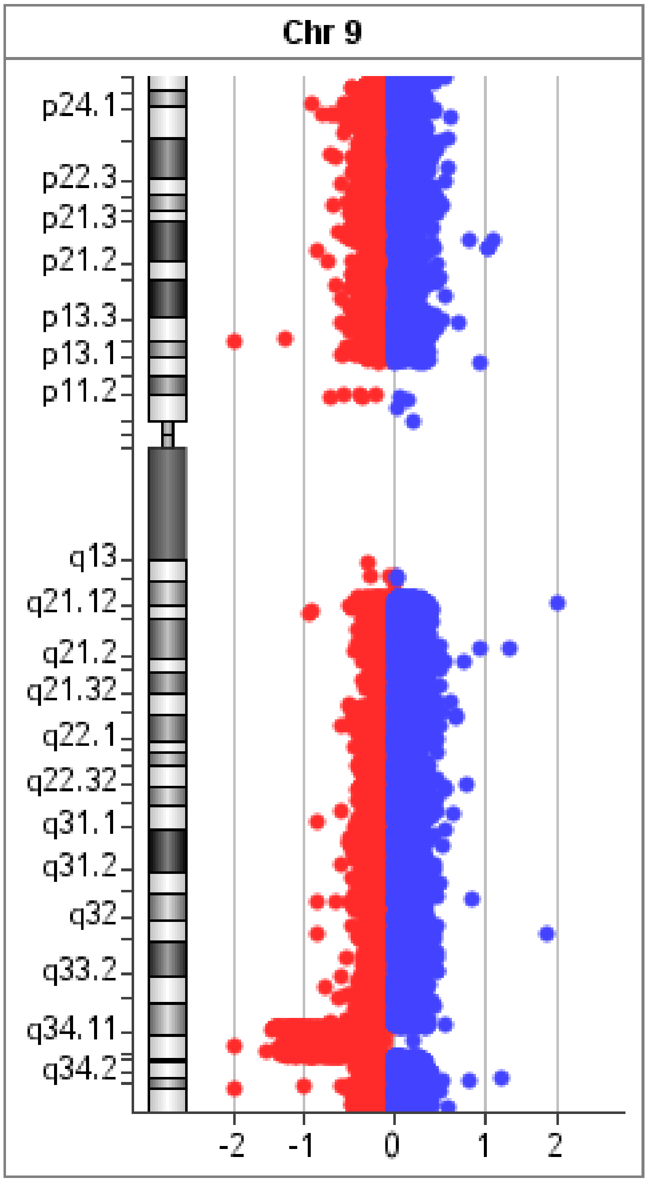

This is an intermediate report and not a final signed off report

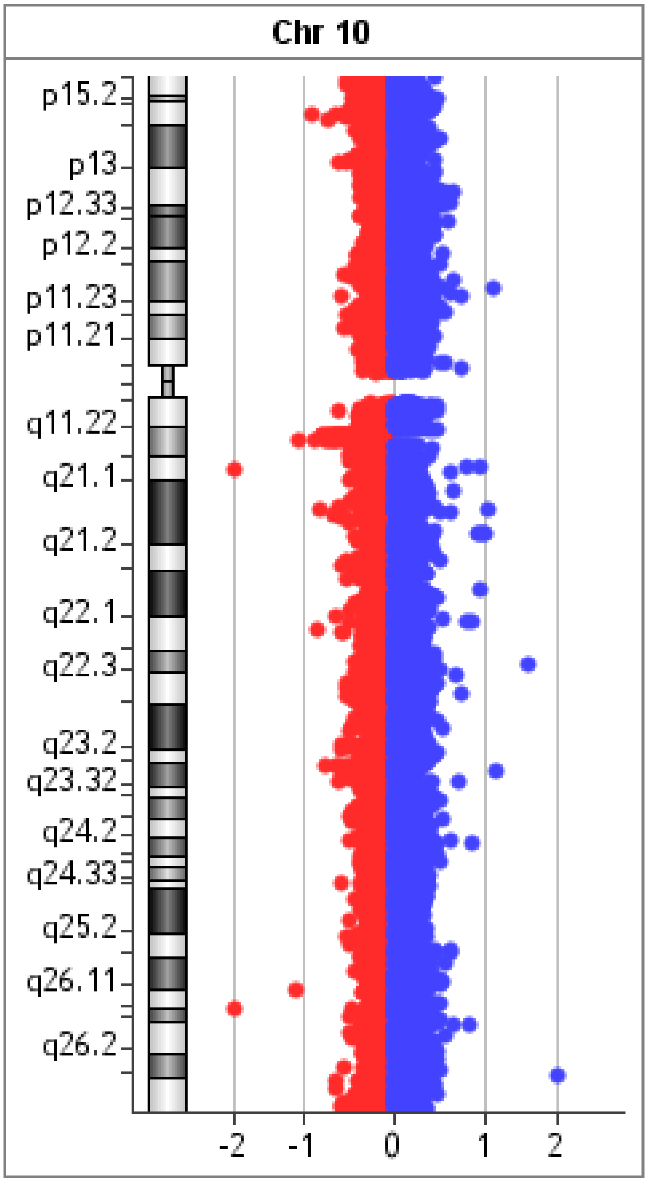

This is an intermediate report and not a final signed off report

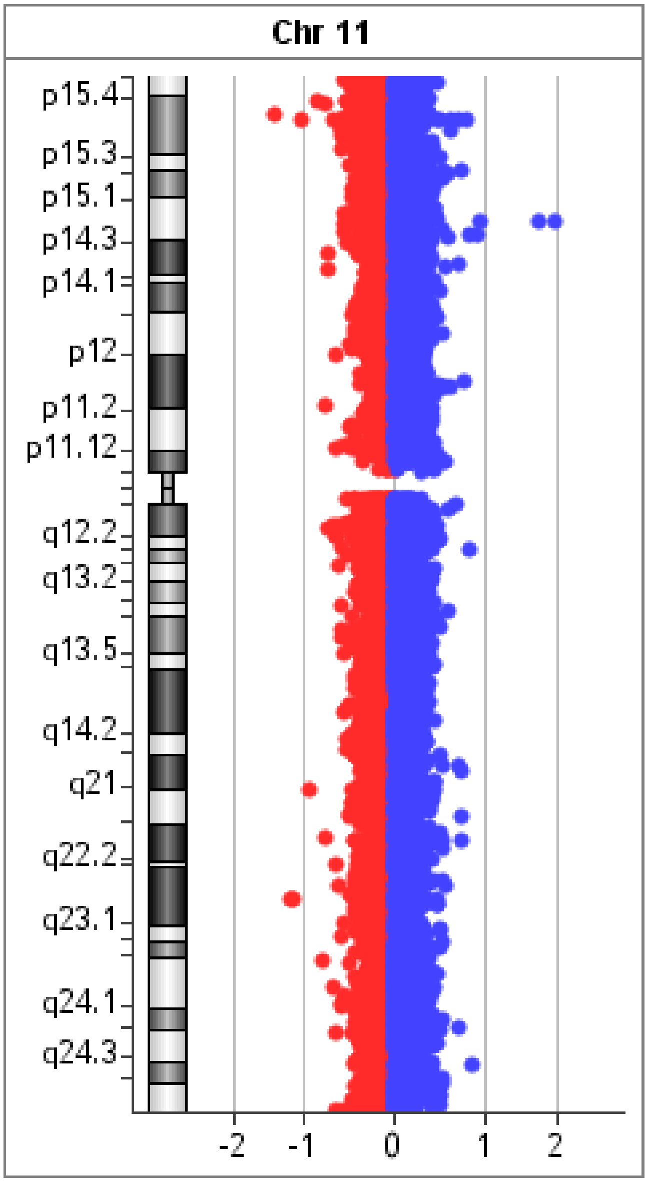

This is an intermediate report and not a final signed off report

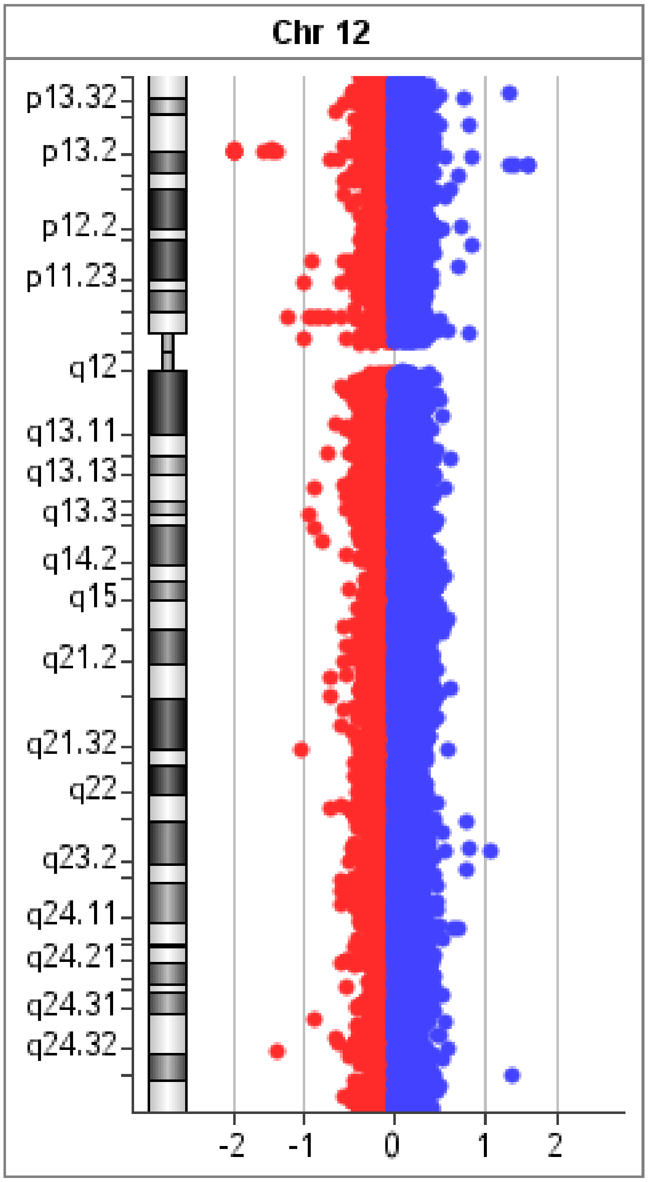

This is an intermediate report and not a final signed off report

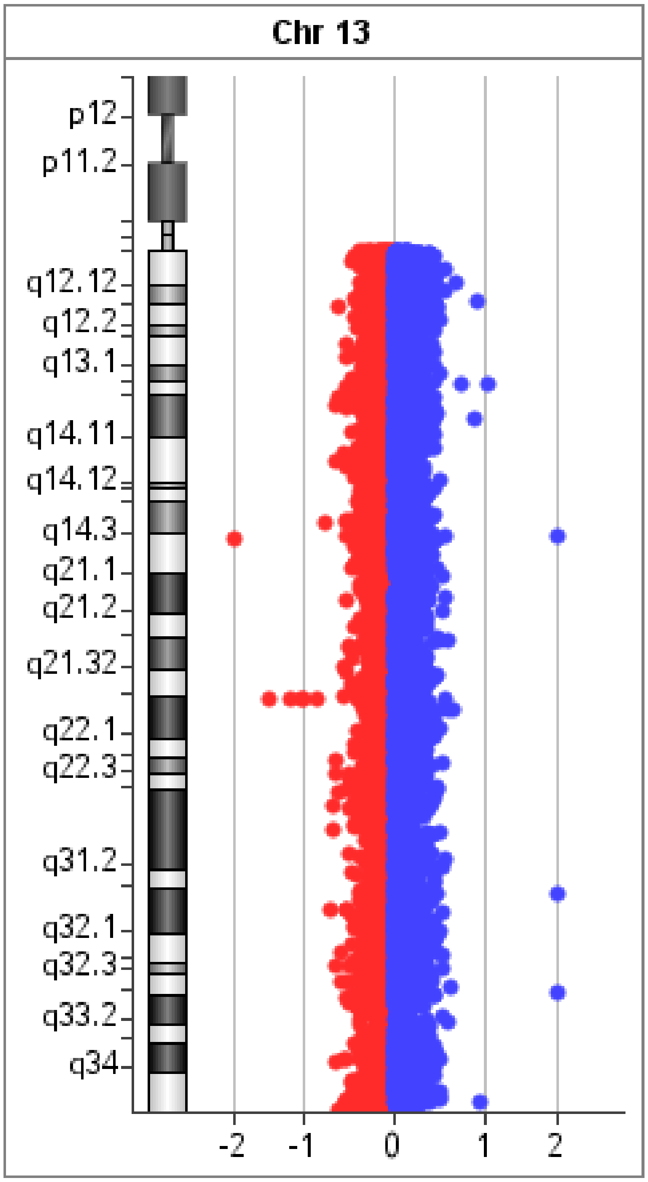

This is an intermediate report and not a final signed off report

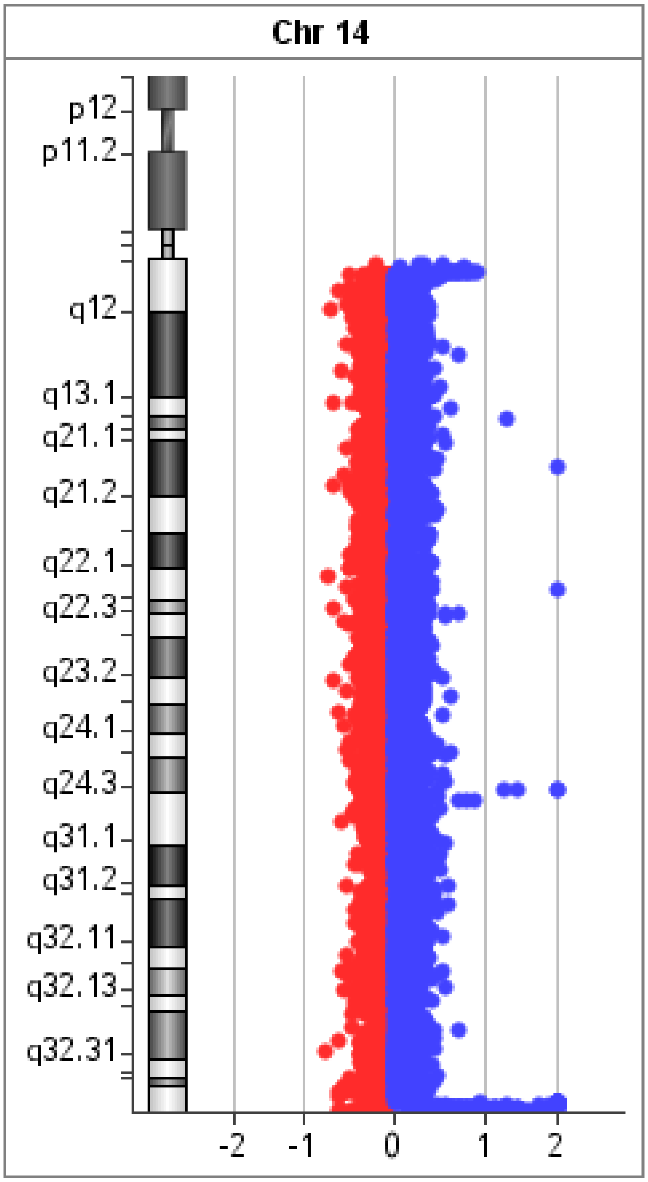

This is an intermediate report and not a final signed off report

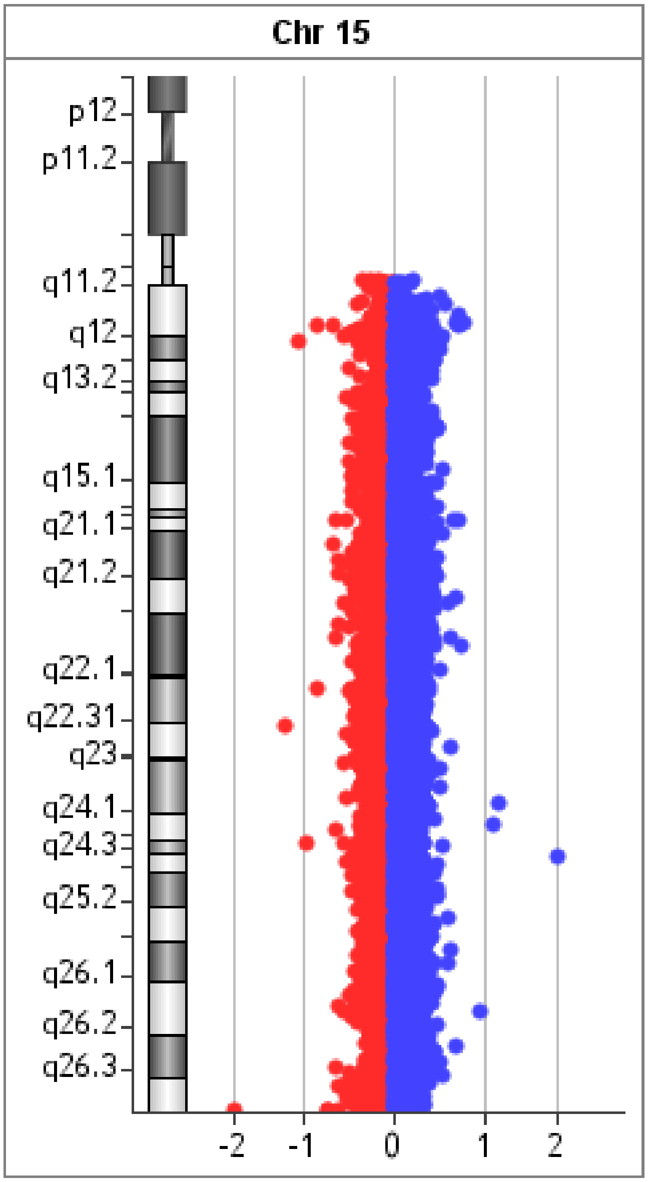

This is an intermediate report and not a final signed off report

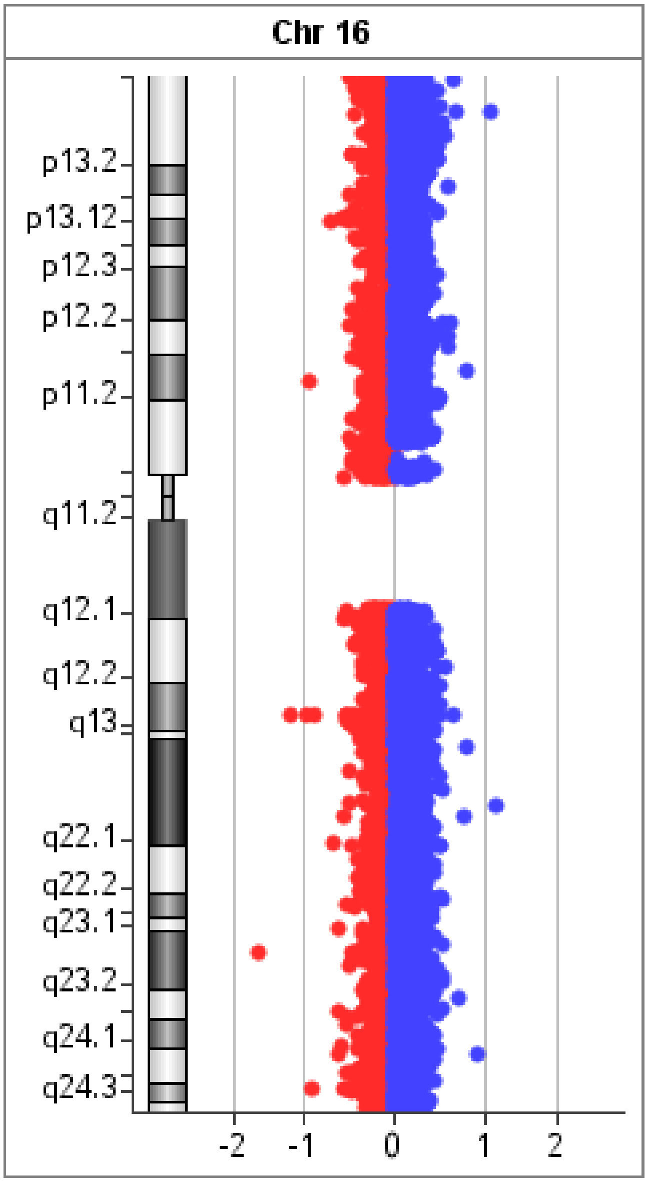

This is an intermediate report and not a final signed off report

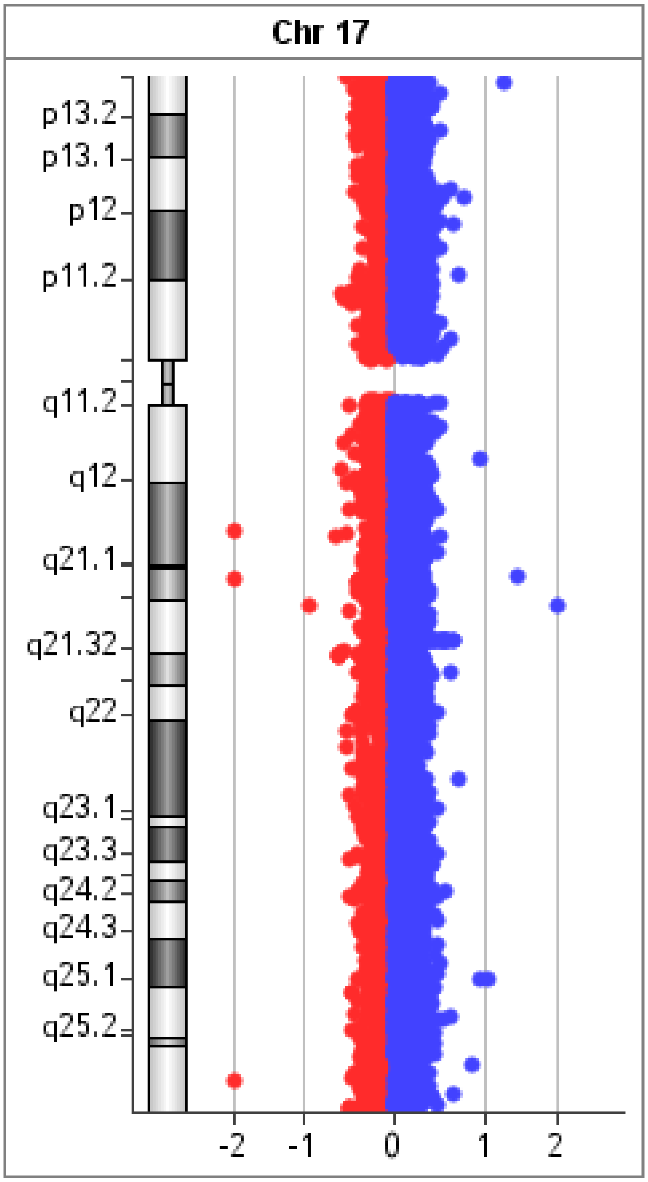

This is an intermediate report and not a final signed off report

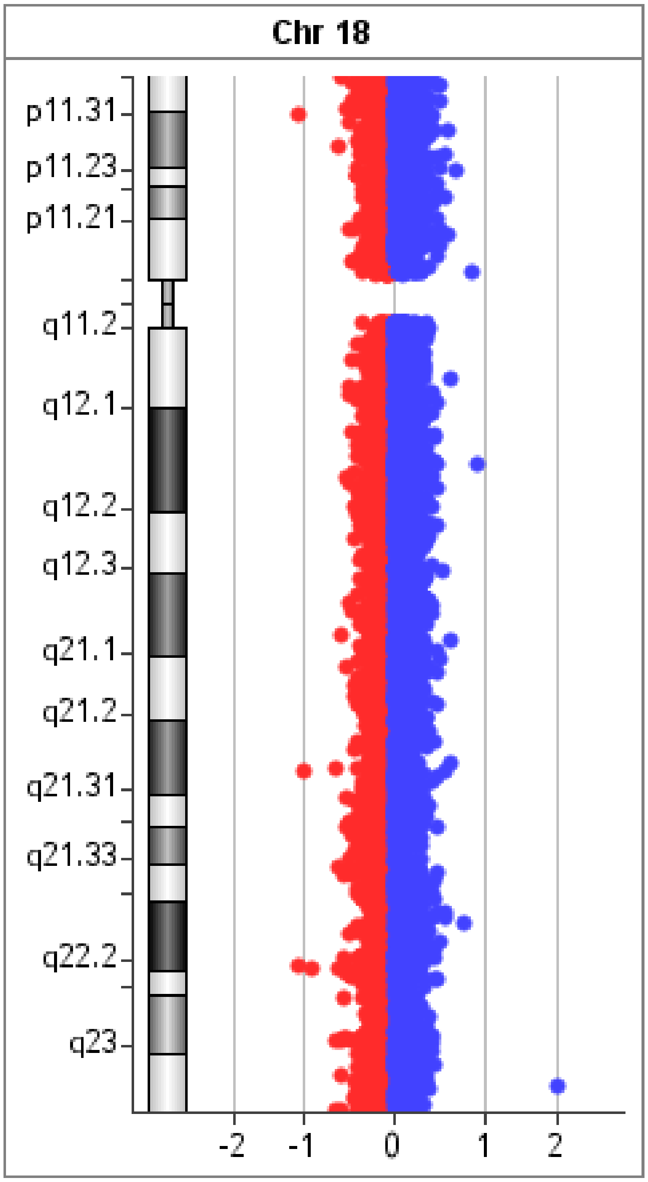

This is an intermediate report and not a final signed off report

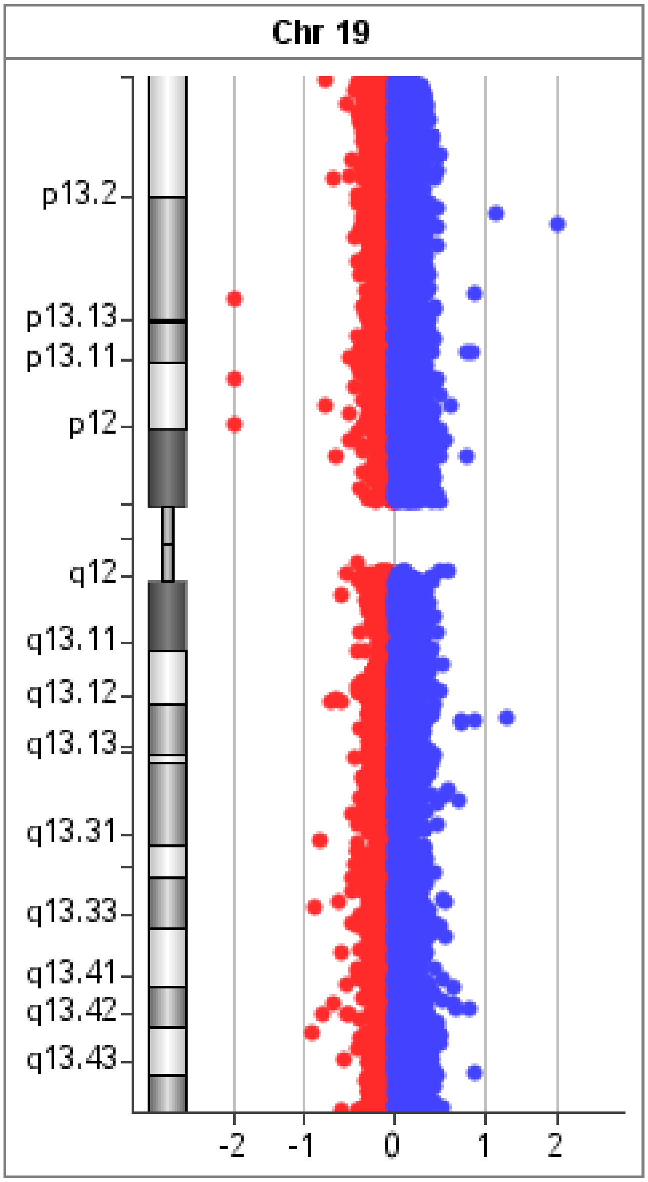

This is an intermediate report and not a final signed off report

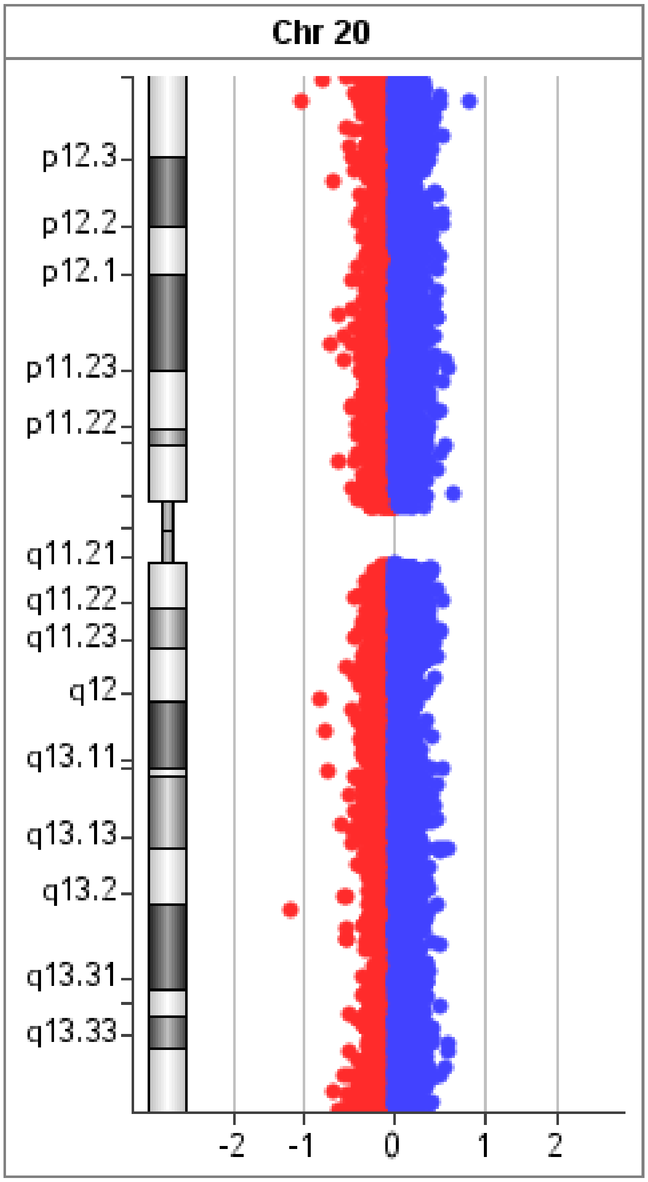

This is an intermediate report and not a final signed off report

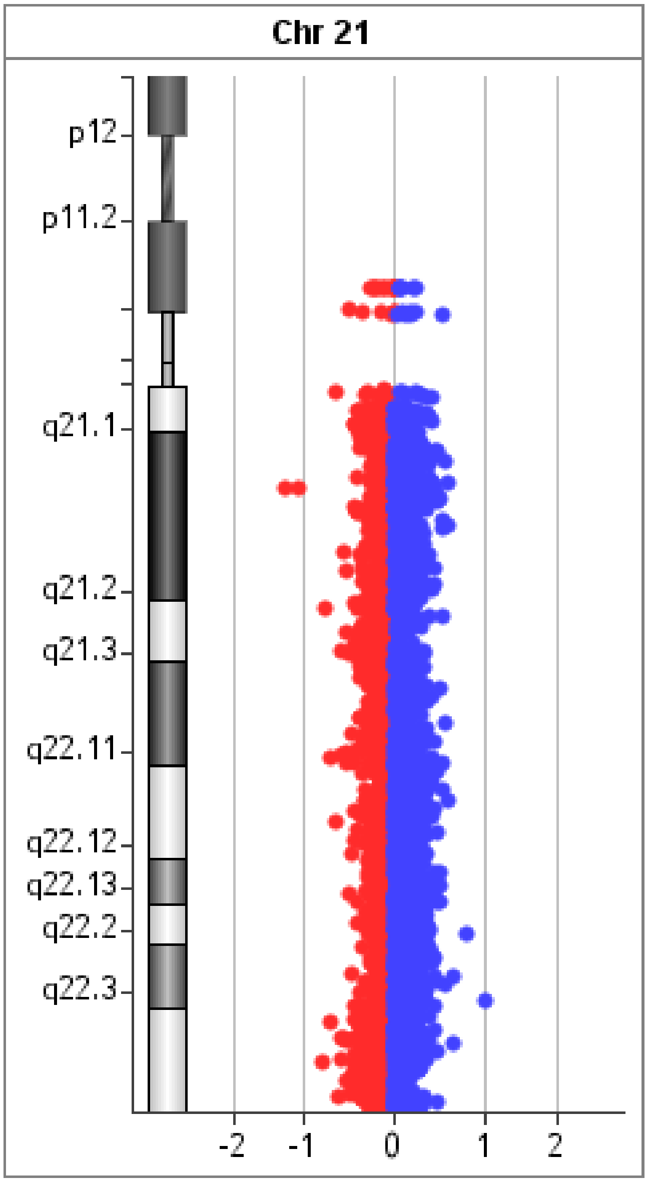

This is an intermediate report and not a final signed off report

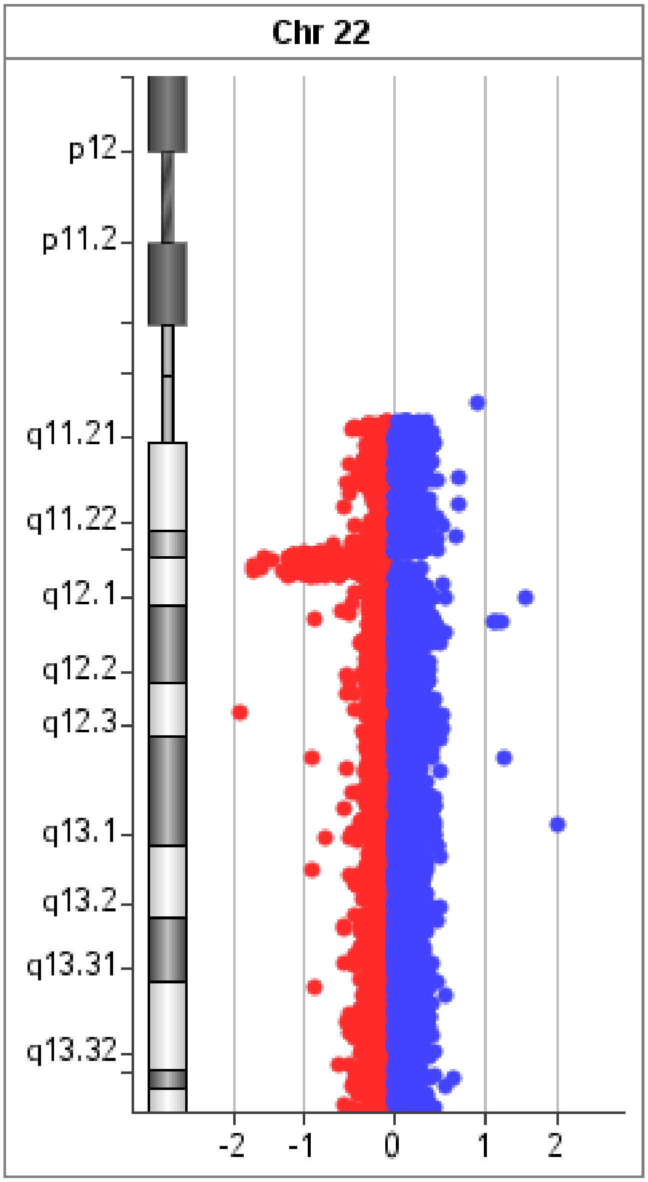

This is an intermediate report and not a final signed off report

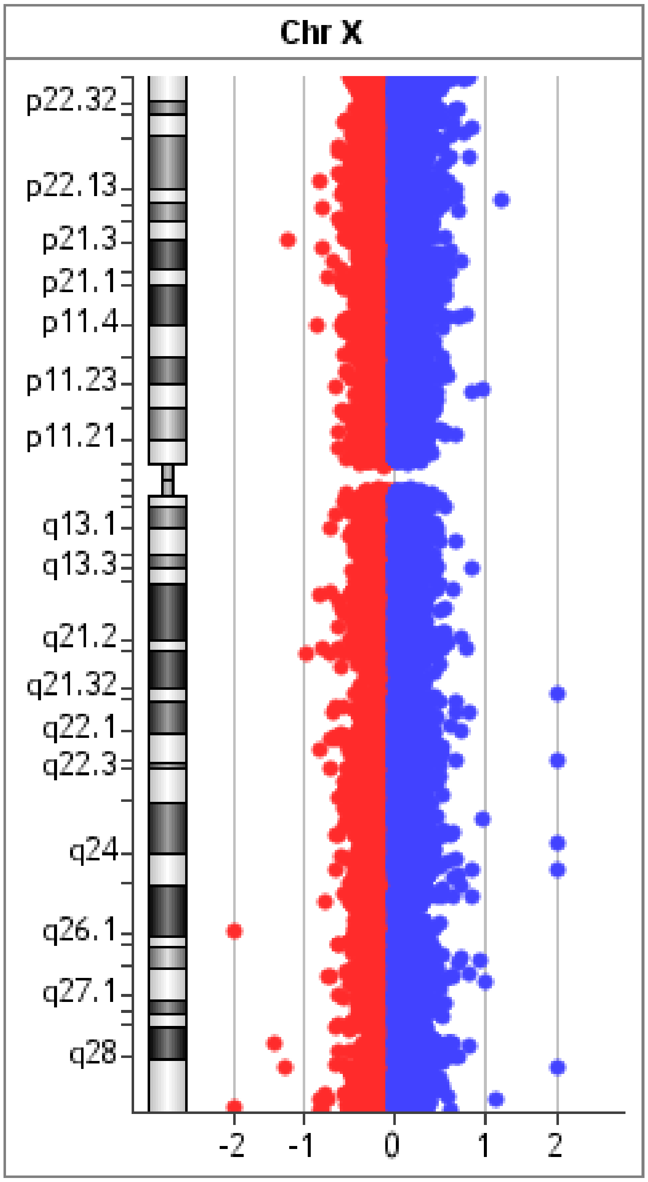

This is an intermediate report and not a final signed off report

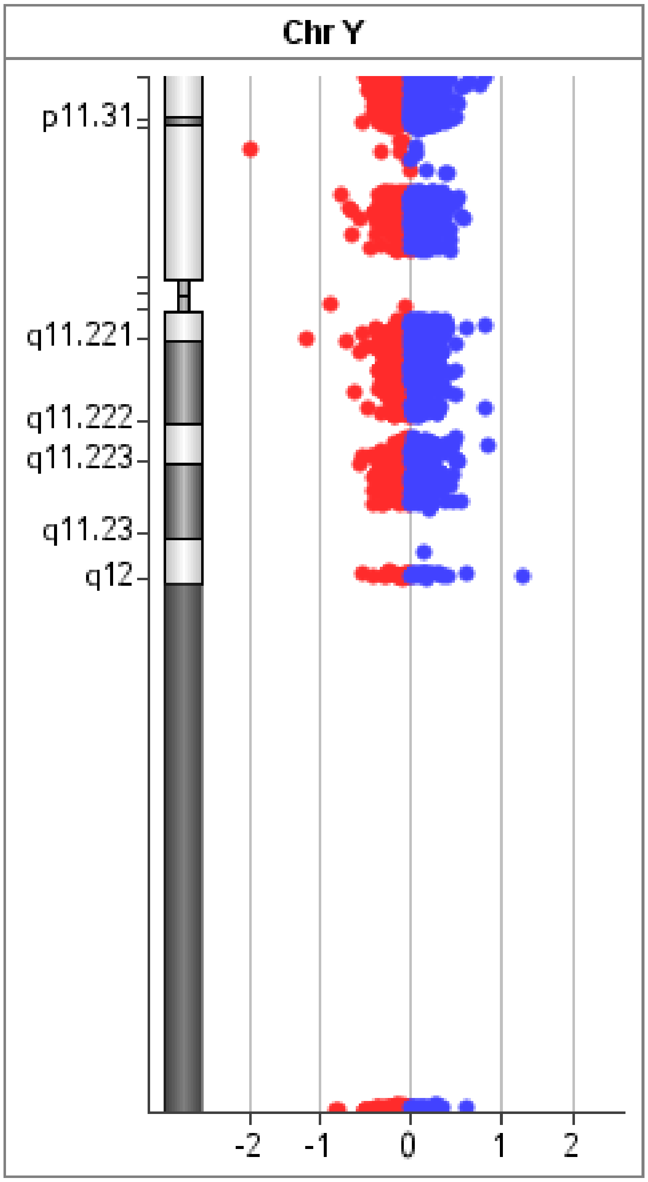

This is an intermediate report and not a final signed off report

Amp/Gain/Loss/Del Intervals Table

Chr	Start-Stop(bp)	Cytoband	Size(kb)	#Probes	Amp/Gain/Loss/Del	Annotations	Classifications
chr1	1509085-1723710	p36.33	214.626	35	0.439	SSU72, C1orf233, MIB2...	
chr2	65033335-65873808	p14	840.474	156	-0.842	LOC101927438, LOC400958, SLC1A4...	
chr9	129644449-133708614	q33.3 - q34.12	4,064.166	753	-0.815	ZBTB34, RALGPS1, ANGPTL2...	
chr22	23634972-24761440	q11.23	1,126.469	212	-0.764	BCR, CES5AP1, ZDHHC8P1...	

Amp=Amplification Del=Deletion

Total Amp/Gain/Loss/Del Intervals: 4

This is an intermediate report and not a final signed off report

ISCN Nomenclature

arr[GRCh37] 1p36.33(1509085_1723710)x3
arr[GRCh37] 2p14(65033335_65873808)x1
arr[GRCh37] 9q33.3q34.12(129644449_133708614)x1
arr[GRCh37] 22q11.23(23634972_24761440)x1

This is an intermediate report and not a final signed off report

Analysis Settings

Design	021850_20150623	Sample Name	14-1385-GM-25218502257 0_1_2
Genome	hg19	Aberration Algorithm	ADM-2
Threshold	6.0	Fuzzy Zero	OFF
GC Correction	ON	Window Size	2Kb
Centralization (legacy)	OFF	Diploid Peak Centralization	ON
SNP Copy Number	OFF	LOH	OFF
Combine Replicates (Intra Array)	ON	Array Level Filter	NONE
Metric Set Filter	NONE	Aberration Filter Name	Default Aberration Filter
Aberration Filter	Minimum Number of Probes for Amplification >= 3 AND Nesting Level <= 100 AND Minimum Avg. Absolute Log Ratio for Amplification >= 0.25 AND Minimum Size (Kb) of Region for Amplification >= 0.0 AND Minimum Size (Kb) of Region for Deletion >= 0.0 AND Minimum Number of Probes for Deletion >= 3 AND Minimum Avg. Absolute Log Ratio for Deletion >= 0.25 AND Minimum Number of Probes for Gain >= 3 AND Minimum Number of Probes for Loss >= 3 AND Minimum Avg. Absolute Log Ratio for Gain >= 0.25 AND Minimum Avg. Absolute Log Ratio for Loss >= 0.25 AND Minimum Size (Kb) of Region for Gain >= 0.0 AND Minimum Size (Kb) of Region for Loss >= 0.0	Feature Level Filter	gIsSaturated = true OR rIsSaturated = true OR gIsFeatNonUnifOL = true OR rIsFeatNonUnifOL = true OR LogRatio = 0; Include matching values=false
Design Level Filter	Homology = 0 OR IsPseudoautosomal = 1	LOH Filter	NONE
Genomic Boundary	OFF	Show Flat Intervals	false
Template Name	ouhsc-cgh		

Genomic region filter for Aberration: OFF

Genomic region filter for LOH: OFF

Notes

Sample Notes

No notes available.

This is an intermediate report and not a final signed off report

Amp/Gain/Loss/Del Interval Notes

No notes available.

This is an intermediate report and not a final signed off report
