# Supplementary material for: Genomic Copy Number Variants in CML Patients With the Philadelphia Chromosome (Ph+): An Update
Source: Front Genet. 2021 Aug 10;12:697009. doi: 10.3389/fgene.2021.697009 (PMC8383316; doi:10.3389/fgene.2021.697009)
Supplement: Supplementary file 10 [file Table_1.DOCX]

**Sample ID: 18-0364-D-M102-Halbrook,Alan**

**Subject information**

| DOB: |  |
| --- | --- |

| Sex: |  |
| --- | --- |

**Sample information**

| Type: |  |
| --- | --- |

| Date received: |  |
| --- | --- |

**Experimental Details**

| Microarray | Barcode: | 203723070079 |
| --- | --- | --- |

| Microarray type: | Illumina CytoSNP-850K v1.2 |
| --- | --- |

| GTC file: | 203723070079_R02C01.gtc |
| --- | --- |

| SNP manifest file: | CytoSNP-850Kv1-2_NS550_B3.bpm | SNP cluster file: | CytoSNP-850Kv1-2_NS550_B3_ClusterFile_GS2011 (2).egt |
| --- | --- | --- | --- |

| Annotation DB: | BG_Annotation_Ens74_20180801.db |
| --- | --- |

| Genome build name: | GRCh37 |
| --- | --- |

| Operator: |  |
| --- | --- |

**Algorithm Settings**

Created by BlueFuse Multi v4.5(32178)

Algorithm: BeadArray v2 - Standard
Smoothing: Backbone = 10

| CGH Reporting: | Minimum Del Size - backbone (Kb) = 0.000 |
| --- | --- |
|  | Minimum Dup Size - backbone (Kb) = 0.000 |
|  | Minimum LOH Region Size (Mb) = 1.0 |
|  | Sex Mismatched Calling = no |
| Significant Clones: | CGH Region = 10 |
|  | LOH Region = 500 |
| Recentering Chromosomes: |  |

**QC Measures**

| QC Status | Not set |
| --- | --- |
| SD Autosome/Robust | 0.15/0.11 |
| DLR Raw | 0.11 |
| Median Log R Deviation | 0.14 |
| Median BAF Deviation | 0.03 |
| Median Call Rate | 1.00 |

**Karyotype View**


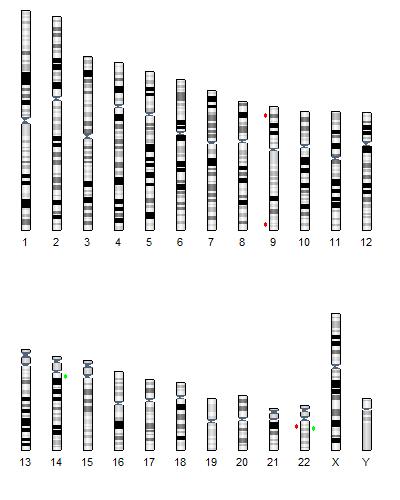


**Detailed Region Summary**

| Region 1 | Start Cyto | End Cyto | Assessment | Type | Copy # | Chromosome | Start | End | Size (bp) | Man Change | StdDev | Included | Excluded | %Included |
| --- | --- | --- | --- | --- | --- | --- | --- | --- | --- | --- | --- | --- | --- | --- |
|  | 9p23 | 9p23 | Unknown? | LOSS | -0.43 | 9 | 9,483,287 | 9,538,589 | 55,303 | no | 0.03 | 25 | 0 | 100.00 |

| Assessment | Region is of unknown significance? It is a Loss of 55Kb (<1Mb), and overlaps 1 HGNC and 1 OMIM gene(s). The maximum overlap between an ISCA pathogenic region of type Loss and this region is 1%. 0% of the region is covered by significant polymorphisms of type Loss (DGV: 0%, ISCA: 0%). |
| --- | --- |
| Region Comments | N/A |
| HGNC genes | PTPRD |
| OMIM genes | " PROTEIN-TYROSINE PHOSPHATASE, RECEPTOR-TYPE, DELTA; PTPRD (*601598)" |
| OMIM diseases |  |
| Diseases |  |
| DGV | \| Study \| Samples \| Regions \| Frequency Range \| Overlap Fraction \| Platform \| \| --- \| --- \| --- \| --- \| --- \| --- \| \| Coe et al. (2014) \| 145420 \| 4 \| 0.0 - 0.0% \| 100.0% \| Oligo aCGH, SNP array \| \| Cooper et al. (2011) \| 52263 \| 3 \| 0.0 - 0.0% \| 78.4% \| Oligo aCGH, SNP array \| \| Shaikh et al. (2009) \| 4052 \| 3 \| 0.0 - 0.1% \| 100.0% \| SNP array \| \| 1000 Genomes Consortium Phase 3 \| 2504 \| 2 \| 0.1 - 0.9% \| 6.1% \| Sequencing \| \| Itsara et al. (2009) \| 1557 \| 1 \| 0.1 - 0.1% \| 40.2% \| Oligo aCGH, SNP array \| \| Uddin et al. (2014) \| 873 \| 1 \| 0.1 - 0.1% \| 100.0% \| SNP array \| \| Conrad et al. (2009) \| 40 \| 1 \| 2.5 - 2.5% \| 1.3% \| Oligo aCGH \| \| Park et al. (2010) \| 31 \| 1 \| 6.5 - 6.5% \| 1.9% \| Oligo aCG \| |
| ISCA Gains | Pathogenic: nssv585239 [unkn orig] (0%), Pathogenic: nssv579110 [unkn orig] (0%), Pathogenic: nssv1415206 [maternal orig] (0%), Pathogenic: nssv1494930 [unkn orig] (0%), Pathogenic: nssv1494937 [unkn orig] (0%), Pathogenic: nssv1415412 [unkn orig] (0%), Pathogenic: nssv1494936 [unkn orig] (0%), Pathogenic: nssv579116 [unkn orig] (0%), Pathogenic: nssv579120 [paternal orig] (0%), Pathogenic: nssv579119 [unkn orig] (0%), Pathogenic: nssv579111 [de_novo orig] (0%), Pathogenic: nssv576748 [unkn orig] (0%), Pathogenic: nssv579122 [unkn orig] (0%), Pathogenic: nssv706201 [unkn orig] (0%), Pathogenic: nssv706521 [maternal orig] (0%), Pathogenic: nssv579121 [unkn orig] (0%), Pathogenic: nssv579147 [unkn orig] (0%), Pathogenic: nssv579118 [unkn orig] (0%), Pathogenic: nssv584344 [unkn orig] (0%), Pathogenic: nssv579112 [unkn orig] (0%), Pathogenic: nssv579123 [unkn orig] (0%), Pathogenic: nssv579149 [unkn orig] (0%), Pathogenic: nssv576650 [unkn orig] (0%), Pathogenic: nssv579124 [de_novo orig] (0%), Pathogenic: nssv579125 [unkn orig] (0%), Pathogenic: nssv707187 [unkn orig] (0%), Pathogenic: nssv576032 [unkn orig] (0%), Pathogenic: nssv579127 [unkn orig] (0%), Pathogenic: nssv579128 [unkn orig] (1%), Pathogenic: nssv583243 [unkn orig] (0%), Pathogenic: nssv1494925 [unkn orig] (0%), Unknown: nssv581357 [maternal orig] (4%), Unknown: nssv581358 [unkn orig] (4%), Unknown_Likely_Benign: nssv1495678 [unkn orig] (2%) |
| ISCA Losses | Pathogenic: nssv578298 [unkn orig] (0%), Pathogenic: nssv578299 [unkn orig] (1%), Pathogenic: nssv578301 [unkn orig] (0%), Pathogenic: nssv578300 [unkn orig] (0%), Pathogenic: nssv576252 [unkn orig] (0%), Pathogenic: nssv1494934 [unkn orig] (1%), Pathogenic: nssv706487 [M,unkn orig] (0%), Pathogenic: nssv1494923 [unkn orig] (0%), Pathogenic: nssv706454 [M,unkn orig] (0%), Pathogenic: nssv576435 [unkn orig] (1%), Pathogenic: nssv578305 [unkn orig] (1%), Pathogenic: nssv575789 [unkn orig] (1%), Pathogenic: nssv706259 [unkn orig] (1%), Pathogenic: nssv575847 [de_novo orig] (0%), Pathogenic: nssv582359 [de_novo orig] (0%), Pathogenic: nssv578306 [unkn orig] (0%), Pathogenic: nssv578307 [unkn orig] (0%), Pathogenic: nssv578309 [unkn orig] (0%), Pathogenic: nssv578304 [unkn orig] (0%), Pathogenic: nssv706596 [de_novo orig] (0%), Pathogenic: nssv576749 [unkn orig] (0%), Pathogenic: nssv578311 [de_novo orig] (0%), Pathogenic: nssv578312 [unkn orig] (0%), Pathogenic: nssv582199 [unkn orig] (0%), Pathogenic: nssv578317 [unkn orig] (0%), Pathogenic: nssv706405 [M,unkn orig] (0%), Pathogenic: nssv578320 [unkn orig] (0%), Unknown: nssv1495366 [unkn orig] (2%), Unknown: nssv584452 [unkn orig] (1%) |

| Region 2 | Start Cyto | End Cyto | Assessment | Type | Copy # | Chromosome | Start | End | Size (bp) | Man Change | StdDev | Included | Excluded | %Included |
| --- | --- | --- | --- | --- | --- | --- | --- | --- | --- | --- | --- | --- | --- | --- |
|  | 9q34.12 | 9q34.12 | Unknown? | LOSS | -0.34 | 9 | 133,595,219 | 133,714,305 | 119,087 | no | 0.08 | 50 | 0 | 100.00 |

| Assessment | Region is of unknown significance? It is a Loss of 119Kb (<1Mb), and overlaps 1 HGNC and 1 OMIM gene(s). The maximum overlap between an ISCA pathogenic region of type Loss and this region is 5%. 0% of the region is covered by significant polymorphisms of type Loss (DGV: 0%, ISCA: 0%). |
| --- | --- |
| Region Comments | N/A |
| HGNC genes | ABL1 |
| OMIM genes | " ABELSON MURINE LEUKEMIA VIRAL ONCOGENE HOMOLOG 1; ABL1 (*189980)" |
| OMIM diseases | LEUKEMIA, CHRONIC MYELOID; CML (#608232) |
| Diseases |  |
| DGV | \| Study \| Samples \| Regions \| Frequency Range \| Overlap Fraction \| Platform \| \| --- \| --- \| --- \| --- \| --- \| --- \| \| Coe et al. (2014) \| 29084 \| 3 \| 0.0 - 0.0% \| 39.8% \| Oligo aCGH, SNP array \| \| 1000 Genomes Consortium Phase 3 \| 2504 \| 2 \| 0.0 - 0.2% \| 10.3% \| Sequencing \| \| Uddin et al. (2014) \| 873 \| 2 \| 0.1 - 0.8% \| 10.5% \| SNP array \| \| Wong et al. (2007) \| 95 \| 1 \| 2.1 - 2.1% \| 60.3% \| BAC aCG \| |
| ISCA Gains | Pathogenic: nssv1494937 [unkn orig] (0%), Pathogenic: nssv1415412 [unkn orig] (0%), Pathogenic: nssv1494936 [unkn orig] (0%), Pathogenic: nssv579121 [unkn orig] (0%), Pathogenic: nssv579147 [unkn orig] (0%), Pathogenic: nssv579118 [unkn orig] (0%), Pathogenic: nssv584344 [unkn orig] (0%), Pathogenic: nssv579112 [unkn orig] (0%), Pathogenic: nssv579123 [unkn orig] (0%), Pathogenic: nssv579149 [unkn orig] (0%), Pathogenic: nssv576650 [unkn orig] (0%), Pathogenic: nssv579124 [de_novo orig] (0%), Pathogenic: nssv579127 [unkn orig] (0%), Pathogenic: nssv584434 [unkn orig] (1%), Pathogenic: nssv579136 [unkn orig] (1%), Pathogenic: nssv579138 [maternal orig] (1%), Pathogenic: nssv579139 [unkn orig] (1%), Unknown: nssv1495364 [unkn orig] (5%), Pathogenic: nssv579142 [unkn orig] (2%), Pathogenic: nssv584430 [unkn orig] (1%), Pathogenic: nssv576610 [unkn orig] (2%) |
| ISCA Losses | Pathogenic: nssv578349 [paternal orig] (5%) |

| Region 3 | Start Cyto | End Cyto | Assessment | Type | Copy # | Chromosome | Start | End | Size (bp) | Man Change | StdDev | Included | Excluded | %Included |
| --- | --- | --- | --- | --- | --- | --- | --- | --- | --- | --- | --- | --- | --- | --- |
|  | 14q11.2 | 14q11.2 | Unknown? | GAIN | 0.17 | 14 | 21,704,661 | 21,733,457 | 28,797 | no | 0.00 | 14 | 0 | 100.00 |

| Assessment | Region is of unknown significance? It is a Gain of 29Kb (<1Mb), and overlaps 1 HGNC and 1 OMIM gene(s). The maximum overlap between an ISCA pathogenic region of type Gain and this region is 2%. 0% of the region is covered by significant polymorphisms of type Gain (DGV: 0%, ISCA: 0%). |
| --- | --- |
| Region Comments | N/A |
| HGNC genes | HNRNPC |
| OMIM genes | " HETEROGENEOUS NUCLEAR RIBONUCLEOPROTEIN C; HNRNPC (*164020)" |
| OMIM diseases |  |
| Diseases |  |
| DGV |  |
| ISCA Gains | Pathogenic: nssv578665 [unkn orig] (0%), Pathogenic: nssv578666 [unkn orig] (2%), Pathogenic: nssv578667 [paternal orig] (0%), Pathogenic: nssv578668 [unkn orig] (1%), Pathogenic: nssv706219 [unkn orig] (0%), Unknown_Likely_Pathogenic: nssv1495740 [unkn orig] (1%), Unknown: nssv580837 [unkn orig] (4%), Unknown: nssv580838 [unkn orig] (9%) |
| ISCA Losses | Pathogenic: nssv577452 [unkn orig] (1%), Pathogenic: nssv577453 [unkn orig] (0%), Pathogenic: nssv577454 [unkn orig] (0%), Pathogenic: nssv577455 [unkn orig] (3%), Pathogenic: nssv577456 [de_novo orig] (4%), Pathogenic: nssv577457 [de_novo orig] (12%) |

| Region 4 | Start Cyto | End Cyto | Assessment | Type | Copy # | Chromosome | Start | End | Size (bp) | Man Change | StdDev | Included | Excluded | %Included |
| --- | --- | --- | --- | --- | --- | --- | --- | --- | --- | --- | --- | --- | --- | --- |
|  | 22q11.23 | 22q11.23 | Pathogenic? | LOSS | -0.26 | 22 | 23,633,252 | 24,707,112 | 1,073,861 | no | 0.04 | 284 | 0 | 100.00 |

| Assessment | Region is pathogenic? It is a Loss of 1074Kb (>=1Mb), and overlaps 34 HGNC and 17 OMIM gene(s). It overlaps the known disease region(s): OMIM disease: CAT EYE SYNDROME; CES (115470), OMIM disease: CHROMOSOME 22q11.2 DELETION SYNDROME, DISTAL (611867). The maximum overlap between an ISCA pathogenic region of type Loss and this region is 38%. 47% of the region is covered by significant polymorphisms of type Loss (DGV: 47%, ISCA: 5%). |
| --- | --- |
| Region Comments | N/A |
| HGNC genes | BCR, CES5AP1, ZDHHC8P1, IGLL1, C22orf43, GUSBP11, ASLP1, RGL4, ZNF70, VPREB3, C22orf15, CHCHD10, MMP11, SMARCB1, DERL3, SLC2A11, RN7SL268P, MIF, GSTT2B, DDTL, DDT, GSTT2, GSTTP1, MTND1P13, EIF4EBP1P2, GSTT1, GSTTP2, CABIN1, SUSD2, GGT5, POM121L9P, BCRP1, SPECC1L, SPECC1L-ADORA2A |
| OMIM genes | " BREAKPOINT CLUSTER REGION; BCR (*151410)", " IMMUNOGLOBULIN LAMBDA-LIKE POLYPEPTIDE 1; IGLL1 (*146770)", " RAL GUANINE NUCLEOTIDE DISSOCIATION STIMULATOR-LIKE 4; RGL4 (*612214)", " ZINC FINGER PROTEIN 70; ZNF70 (*194544)", " PRE-B-LYMPHOCYTE GENE 3; VPREB3 (*605017)", " MATRIX METALLOPROTEINASE 11; MMP11 (*185261)", " SWI/SNF-RELATED, MATRIX-ASSOCIATED, ACTIN-DEPENDENT REGULATOR OF CHROMATIN, (*601607)", " DER1-LIKE DOMAIN FAMILY, MEMBER 3; DERL3 (*610305)", " SOLUTE CARRIER FAMILY 2 (FACILITATED GLUCOSE TRANSPORTER), MEMBER (*610367)", " MACROPHAGE MIGRATION INHIBITORY FACTOR; MIF (*153620)", " GLUTATHIONE S-TRANSFERASE, THETA-2; GSTT2 (*600437)", " D-DOPACHROME TAUTOMERASE; DDT (*602750)", " GLUTATHIONE S-TRANSFERASE, THETA-2; GSTT2 (*600437)", " GLUTATHIONE S-TRANSFERASE, THETA-1; GSTT1 (*600436)", " CALCINEURIN-BINDING PROTEIN 1 (*604251)", " GAMMA-GLUTAMYLTRANSFERASE 5; GGT5 (*137168)", " SPERM ANTIGEN WITH CALPONIN HOMOLOGY AND COILED-COIL DOMAINS 1-LIKE; (*614140)" |
| OMIM diseases | LEUKEMIA, CHRONIC MYELOID; CML (#608232), AGAMMAGLOBULINEMIA 2, AUTOSOMAL RECESSIVE; AGM2 (#613500), SCHWANNOMATOSIS (#162091), RHABDOID TUMOR PREDISPOSITION SYNDROME 1; RTPS1 (#609322), MENTAL RETARDATION, AUTOSOMAL DOMINANT 15; MRD15 (#614608), RHEUMATOID ARTHRITIS, SYSTEMIC JUVENILE (#604302), FACIAL CLEFTING, OBLIQUE, 1; OBLFC1 (#600251) |
| Diseases | OMIM disease: CAT EYE SYNDROME; CES (115470), OMIM disease: CHROMOSOME 22q11.2 DELETION SYNDROME, DISTAL (611867) |
| DGV | \| Study \| Samples \| Regions \| Frequency Range \| Overlap Fraction \| Platform \| \| --- \| --- \| --- \| --- \| --- \| --- \| \| Coe et al. (2014) \| 203588 \| 15 \| 0.0 - 0.1% \| 22.9% \| Oligo aCGH, SNP array \| \| Cooper et al. (2011) \| 156789 \| 54 \| 0.0 - 3.0% \| 26.9% \| Oligo aCGH, SNP array \| \| Suktitipat et al. (2014) \| 9051 \| 5 \| 0.0 - 0.3% \| 9.2% \| SNP array \| \| Shaikh et al. (2009) \| 4052 \| 7 \| 0.0 - 0.2% \| 20.9% \| SNP array \| \| 1000 Genomes Consortium Phase 3 \| 2504 \| 18 \| 0.0 - 86.4% \| 16.3% \| Sequencing \| \| Campbell et al. (2011) \| 2366 \| 1 \| 5.6 - 5.6% \| 4.4% \| Oligo aCGH \| \| Vogler et al. (2010) \| 2218 \| 2 \| 9.2 - 9.4% \| 11.0% \| Merging, SNP array \| \| Uddin et al. (2014) \| 1746 \| 7 \| 0.1 - 3.3% \| 12.4% \| SNP array \| \| Itsara et al. (2009) \| 1557 \| 1 \| 0.1 - 0.1% \| 5.6% \| Oligo aCGH, SNP array \| \| Jakobsson et al. (2008) \| 443 \| 1 \| 0.2 - 0.2% \| 7.4% \| SNP array \| \| McCarroll et al. (2008) \| 270 \| 2 \| 76.3 - 76.7% \| 9.7% \| SNP array \| \| Redon et al. (2006) \| 270 \| 1 \| 1.5 - 1.5% \| 13.6% \| BAC aCGH, SNP array \| \| McCarroll et al. (2006) \| 269 \| 2 \| 0.7 - 5.6% \| 0.2% \| SNP array \| \| Locke et al. (2006) \| 265 \| 1 \| 0.4 - 0.4% \| 21.8% \| BAC aCGH \| \| Wong et al. (2007) \| 190 \| 3 \| 1.1 - 3.2% \| 33.7% \| BAC aCGH \| \| Park et al. (2010) \| 155 \| 31 \| 3.2 - 48.4% \| 12.3% \| Oligo aCGH \| \| Mokhtar et al. (2014) \| 68 \| 4 \| 2.9 - 14.7% \| 10.6% \| SNP array \| \| Conrad et al. (2006) \| 60 \| 1 \| 1.7 - 1.7% \| 2.5% \| Oligo aCGH, SNP array \| \| de Smith et al. (2007) \| 51 \| 2 \| 2.0 - 17.6% \| 6.0% \| Oligo aCGH \| \| Conrad et al. (2009) \| 40 \| 6 \| 2.5 - 75.0% \| 21.4% \| Oligo aCGH \| \| Iafrate et al. (2004) \| 39 \| 1 \| 7.7 - 7.7% \| 18.1% \| BAC aCGH, FISH \| \| Perry et al. (2008) \| 31 \| 5 \| 3.2 - 67.7% \| 43.6% \| Oligo aCGH \| \| Cooper et al. (2008) \| 9 \| 2 \| 11.1 - 11.1% \| 3.6% \| SNP array \| \| Kidd et al. (2010b) \| 9 \| 2 \| 11.1 - 22.2% \| 8.7% \| Sequencing \| \| Pang et al. (2010) \| 3 \| 1 \| 33.3 - 33.3% \| 0.1% \| Oligo aCGH, Sequencing, SNP array \| \| Arlt et al. (2011) \| 2 \| 11 \| 100.0 - 100.0% \| 7.3% \| Sequencing, SNP array \| \| Kim et al. (2009) \| 2 \| 2 \| 50.0 - 50.0% \| 0.1% \| Oligo aCGH, Sequencing, SNP array \| \| McKernan et al. (2009) \| 1 \| 1 \| 100.0 - 100.0% \| 11.2% \| Sequencing \| \| Schuster et al. (2010) \| 1 \| 1 \| 100.0 - 100.0% \| 0.8% \| Oligo aCGH, Sequencin \| |
| ISCA Gains | Pathogenic: nssv578919 [maternal orig] (15%), Pathogenic: nssv575290 [unkn orig] (3%), Pathogenic: nssv706258 [unkn orig] (3%), Pathogenic: nssv583831 [unkn orig] (3%), Pathogenic: nssv578922 [unkn orig] (8%), Pathogenic: nssv1495084 [unkn orig] (7%), Unknown: nssv580278 [unkn orig] (1%), Pathogenic: nssv582213 [maternal orig] (6%), Unknown: nssv580279 [maternal orig] (6%), Unknown: nssv1495586 [unkn orig] (54%), Unknown: nssv1495576 [unkn orig] (54%), Unknown: nssv1415248 [unkn orig] (53%), Pathogenic: nssv585232 [unkn orig] (2%), Unknown: nssv582975 [unkn orig] (2%), Unknown_Likely_Pathogenic: nssv575342 [maternal orig] (55%), Unknown: nssv580282 [maternal orig] (55%), Unknown: nssv582968 [maternal orig] (55%), Unknown: nssv706420 [M,unkn orig] (55%), Unknown: nssv580281 [maternal orig] (55%), Unknown: nssv706908 [F,unkn orig] (52%), Unknown: nssv580283 [paternal orig] (55%), Unknown: nssv580284 [unkn orig] (55%), Unknown: nssv580285 [maternal orig] (53%), Unknown: nssv581067 [unkn orig] (90%), Pathogenic: nssv1495103 [unkn orig] (8%), Unknown: nssv580286 [unkn orig] (72%), Unknown: nssv1495580 [unkn orig] (78%), Unknown: nssv1495574 [unkn orig] (76%), Unknown: nssv580288 [unkn orig] (79%), Unknown: nssv580287 [unkn orig] (77%), Unknown: nssv580289 [unkn orig] (81%), Unknown: nssv580290 [unkn orig] (74%), Unknown: nssv580291 [unkn orig] (80%), Unknown_Likely_Pathogenic: nssv575844 [unkn orig] (80%), Unknown: nssv584380 [unkn orig] (80%), Unknown: nssv580293 [paternal orig] (80%), Unknown: nssv580292 [unkn orig] (80%), Unknown: nssv582818 [de_novo orig] (80%), Unknown: nssv576328 [unkn orig] (80%), Unknown: nssv580294 [paternal orig] (73%), Unknown: nssv580295 [unkn orig] (73%), Unknown: nssv580296 [unkn orig] (79%), Unknown: nssv1415411 [unkn orig] (100%), Unknown: nssv1415506 [unkn orig] (100%), Unknown: nssv583232 [unkn orig] (61%), Unknown_Likely_Benign: nssv585029 [unkn orig] (100%) |
| ISCA Losses | Pathogenic: nssv582219 [unkn orig] (27%), Pathogenic: nssv580073 [maternal orig] (5%), Pathogenic: nssv580072 [de_novo orig] (35%), Pathogenic: nssv580074 [unkn orig] (36%), Pathogenic: nssv580079 [de_novo orig] (1%), Pathogenic: nssv1495077 [unkn orig] (1%), Pathogenic: nssv584439 [unkn orig] (6%), Pathogenic: nssv580078 [unkn orig] (34%), Pathogenic: nssv1495070 [unkn orig] (36%), Pathogenic: nssv580076 [unkn orig] (37%), Pathogenic: nssv580080 [de_novo orig] (35%), Pathogenic: nssv580081 [de_novo orig] (38%), Pathogenic: nssv580085 [unkn orig] (1%), Pathogenic: nssv580087 [unkn orig] (1%), Pathogenic: nssv580089 [unkn orig] (2%), Pathogenic: nssv580088 [unkn orig] (2%), Unknown_Likely_Pathogenic: nssv576708 [unkn orig] (2%), Pathogenic: nssv580090 [unkn orig] (4%), Pathogenic: nssv580091 [unkn orig] (4%), Benign: nssv707475 [unkn orig] (100%), Benign: nssv707444 [unkn orig] (100%), Benign: nssv707371 [unkn orig] (100%), Benign: nssv707555 [unkn orig] (100%), Benign: nssv707285 [unkn orig] (100%), Benign: nssv707386 [unkn orig] (100%), Benign: nssv707427 [unkn orig] (100%), Benign: nssv707346 [unkn orig] (100%), Benign: nssv707395 [unkn orig] (100%), Benign: nssv707275 [unkn orig] (100%), Benign: nssv707308 [unkn orig] (100%), Benign: nssv581968 [unkn orig] (100%), Benign: nssv581969 [unkn orig] (100%), Benign: nssv581962 [unkn orig] (100%), Benign: nssv581964 [unkn orig] (100%), Benign: nssv581961 [unkn orig] (100%) |

| Region 5 | Start Cyto | End Cyto | Assessment | Type | Copy # | Chromosome | Start | End | Size (bp) | Man Change | StdDev | Included | Excluded | %Included |
| --- | --- | --- | --- | --- | --- | --- | --- | --- | --- | --- | --- | --- | --- | --- |
|  | 22q11.23 | 22q12.1 | Unknown? | GAIN | 0.06 | 22 | 25,809,371 | 25,910,667 | 101,297 | no | 0.04 | 16 | 0 | 100.00 |

| Assessment | Region is of unknown significance? It is a Gain of 101Kb (<1Mb), and overlaps 2 HGNC and 0 OMIM gene(s). It overlaps the known disease region(s): OMIM disease: CAT EYE SYNDROME; CES (115470), OMIM disease: CHROMOSOME 22q11.2 DELETION SYNDROME, DISTAL (611867). The maximum overlap between an ISCA pathogenic region of type Gain and this region is 1%. 100% of the region is covered by significant polymorphisms of type Gain (DGV: 100%, ISCA: 82%). |
| --- | --- |
| Region Comments | N/A |
| HGNC genes | IGLVIVOR22-1, CRYBB2P1 |
| OMIM genes |  |
| OMIM diseases |  |
| Diseases | OMIM disease: CAT EYE SYNDROME; CES (115470), OMIM disease: CHROMOSOME 22q11.2 DELETION SYNDROME, DISTAL (611867) |
| DGV | \| Study \| Samples \| Regions \| Frequency Range \| Overlap Fraction \| Platform \| \| --- \| --- \| --- \| --- \| --- \| --- \| \| Coe et al. (2014) \| 232672 \| 2 \| 0.2 - 0.3% \| 100.0% \| Oligo aCGH, SNP array \| \| Cooper et al. (2011) \| 121947 \| 6 \| 0.1 - 0.2% \| 100.0% \| Oligo aCGH, SNP array \| \| Suktitipat et al. (2014) \| 9051 \| 5 \| 0.8 - 1.5% \| 100.0% \| SNP array \| \| 1000 Genomes Consortium Phase 3 \| 7512 \| 5 \| 5.0 - 5.7% \| 100.0% \| Sequencing \| \| Itsara et al. (2009) \| 6228 \| 3 \| 1.2 - 2.3% \| 100.0% \| Oligo aCGH, SNP array \| \| Campbell et al. (2011) \| 2366 \| 1 \| 1.1 - 1.1% \| 100.0% \| Oligo aCGH \| \| Shaikh et al. (2009) \| 2026 \| 1 \| 2.3 - 2.3% \| 100.0% \| SNP array \| \| Uddin et al. (2014) \| 1746 \| 1 \| 2.3 - 2.3% \| 100.0% \| SNP array \| \| Pinto et al. (2007) \| 1542 \| 1 \| 9.5 - 9.5% \| 100.0% \| SNP array \| \| Vogler et al. (2010) \| 1109 \| 1 \| 4.8 - 4.8% \| 100.0% \| Merging, SNP array \| \| Locke et al. (2006) \| 530 \| 3 \| 1.5 - 2.3% \| 100.0% \| BAC aCGH \| \| Jakobsson et al. (2008) \| 443 \| 1 \| 4.7 - 4.7% \| 100.0% \| SNP array \| \| McCarroll et al. (2008) \| 270 \| 1 \| 6.7 - 6.7% \| 100.0% \| SNP array \| \| Redon et al. (2006) \| 270 \| 1 \| 6.7 - 6.7% \| 100.0% \| BAC aCGH, SNP array \| \| Wang et al. (2007) \| 224 \| 1 \| 2.7 - 2.7% \| 100.0% \| SNP array \| \| Wong et al. (2007) \| 95 \| 1 \| 1.1 - 1.1% \| 64.7% \| BAC aCGH \| \| Park et al. (2010) \| 93 \| 4 \| 4.3 - 6.5% \| 100.0% \| Oligo aCGH \| \| de Smith et al. (2007) \| 51 \| 1 \| 41.2 - 41.2% \| 100.0% \| Oligo aCGH \| \| Sharp et al. (2005) \| 48 \| 1 \| 8.3 - 8.3% \| 56.4% \| BAC aCGH, FISH \| \| Conrad et al. (2009) \| 40 \| 1 \| 10.0 - 10.0% \| 100.0% \| Oligo aCGH \| \| Mokhtar et al. (2014) \| 34 \| 1 \| 32.4 - 32.4% \| 100.0% \| SNP array \| \| Perry et al. (2008) \| 31 \| 1 \| 9.7 - 9.7% \| 100.0% \| Oligo aCGH \| \| Sebat et al. (2004) \| 31 \| 1 \| 3.2 - 3.2% \| 100.0% \| ROMA \| \| Cooper et al. (2008) \| 9 \| 1 \| 11.1 - 11.1% \| 100.0% \| SNP array \| \| McKernan et al. (2009) \| 1 \| 1 \| 100.0 - 100.0% \| 100.0% \| Sequencin \| |
| ISCA Gains | Pathogenic: nssv575290 [unkn orig] (0%), Pathogenic: nssv706258 [unkn orig] (0%), Pathogenic: nssv583831 [unkn orig] (0%), Pathogenic: nssv578922 [unkn orig] (1%), Pathogenic: nssv1495084 [unkn orig] (1%), Pathogenic: nssv1495103 [unkn orig] (1%), Unknown: nssv1495575 [unkn orig] (9%), Benign: nssv581758 [unkn orig] (36%), Benign: nssv581761 [unkn orig] (36%) |
| ISCA Losses | Unknown_Likely_Pathogenic: nssv584491 [de_novo orig] (3%), Benign: nssv581592 [unkn orig] (34%), Benign: nssv707454 [unkn orig] (39%), Benign: nssv581805 [unkn orig] (45%), Benign: nssv578714 [unkn orig] (45%), Benign: nssv581955 [unkn orig] (45%), Benign: nssv581860 [unkn orig] (45%), Benign: nssv581874 [unkn orig] (45%), Benign: nssv581855 [unkn orig] (45%), Unknown: nssv1495578 [unkn orig] (45%) |

**ISCN**

Warning: The sample sex was not specified, assuming Male

| ISCN | Size (bp) |
| --- | --- |
| arr[GRCh37] 9p23(9483287_9538589)x1 | 55,303 |
| arr[GRCh37] 9q34.12(133595219_133714305)x1 | 119,087 |
| arr[GRCh37] 14q11.2(21704661_21733457)x3 | 28,797 |
| arr[GRCh37] 22q11.23(23633252_24707112)x1 | 1,073,861 |
| arr[GRCh37] 22q11.23q12.1(25809371_25910667)x3 | 101,297 |

**LOH Region Tabular View**

| Number of Regions | 0 |
| --- | --- |

|  |
| --- |

| Total Autosomal LOH: | 0.16% (4.35Mb) |
| --- | --- |

|  |
| --- |

**Warning: All results should be confirmed using an alternative technique.**
